# FluoroFusion: NHC-Catalyzed Nucleophilic Aromatic Substitution Reaction Unveils Functional Perfluorinated Diarylmethanones

**DOI:** 10.1021/acs.orglett.4c00677

**Published:** 2024-03-08

**Authors:** Cheng-Lin Chan, Shao-Chi Lee, Pei-Shan Lin, Radyn Vanessa Phaz P. Tapales, Jia-Syuan Li, Chun-An Lai, Jyh-Tsung Lee, Chien-Hung Li, Hsuan-Hung Liao

**Affiliations:** † Department of Chemistry, National Sun Yat-sen University, Kaohsiung 804201, Taiwan (R.O.C.); ‡ Department of International Ph.D. Program for Science, National Sun Yat-sen University, Kaohsiung 804201, Taiwan (R.O.C.); § Department of Chemistry, National Chung Hsing University, Taichung 402202, Taiwan (R.O.C.); ∥ KAUST Catalysis Center (KCC), King Abdullah University of Science and Technology (KAUST), Thuwal 23955, Saudi Arabia; ⊥ Department of Applied and Medicinal Chemistry, Kaohsiung Medical University, Kaohsiung 807378, Taiwan (R.O.C.)

## Abstract

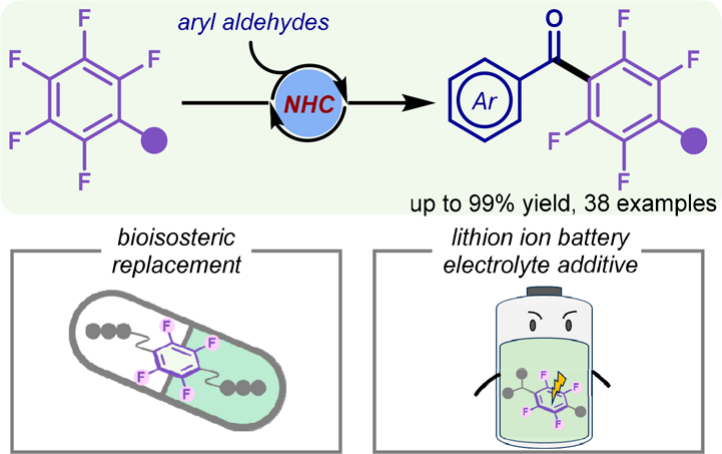

A mild, facile, and metal-free approach via the *N*-heterocyclic carbene-catalyzed S_N_Ar reaction between aryl aldehydes with perfluoroarenes to obtain the coveted functional perfluorinated diarylmethanones is disclosed. This method accommodates a diverse substrate range and exhibits notable tolerance toward various functional groups. Our success in modifying biologically relevant molecules, crafting a fully fluorinated bioisosteric analogue of drug candidate **D1**, and highlighting the potential of these ketones as valuable electrolyte additives for lithium-ion batteries (LIBs) underscores the versatility of our methodology.

Fluorine, renowned for its distinctive properties, has catalyzed the advancement of a wide range of fluorinated molecules, now integral to various scientific domains.^1^ Perfluorinated arenes, notably, have garnered significant attention in recent years due to their unique attributes,^2^ particularly in modifying molecular properties through hydrogen substitution with fluorine in aromatic compounds, thereby enhancing performance across pharmaceuticals, agrochemicals, and materials science applications.^3−5^ Within this realm, perfluorinated diarylmethanones emerge as the quintessential structural motif. Their significance is underlined by several applications (Scheme 1a), for instance, in vemurafenib, known as a potent therapeutic agent for melanoma skin cancer.^6^ Moreover, the utility of these compounds as synthetic handles is undeniable, as they adeptly transform carbonyl groups into a suite of functional entities. Illustratively, when perfluoroaryl diarylmethanones undergo reduction, they yield perfluoroarene-containing diarylmethanes, which form the backbone of entrectinib,^7^ a renowned kinase inhibitor. On another front, these ketones, when subjected to condensation reactions, birth highly conjugated structures, with perfluoroarene-additive doping material (**PFA-ADM1**) being a prime example.^8^ Given the promising outcomes and potential applications, the need for efficient synthetic methods to access perfluorinated diarylmethanones is evident. However, challenges persist, making their functionalization a critical and complex scientific endeavor.

**Scheme 1:**
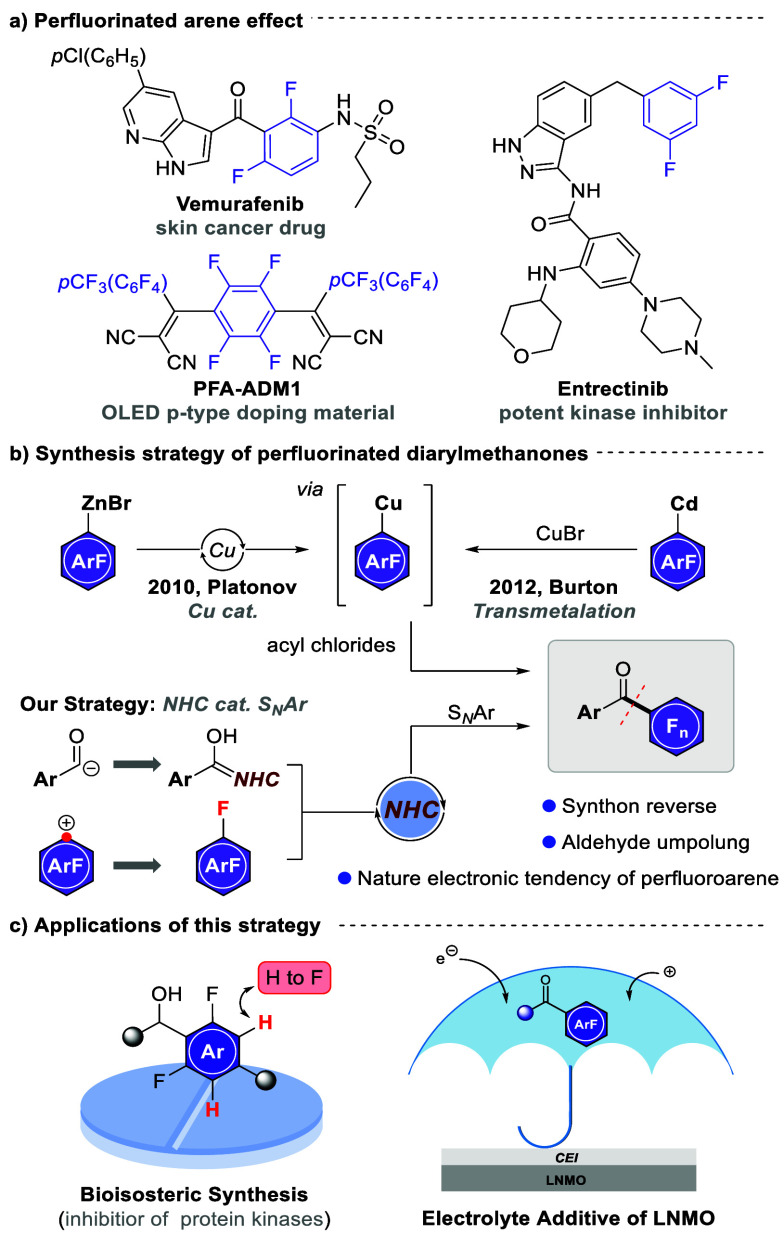
Synthesis and Application of Perfluorinated Arene (a) Relevance of perfluorinated diarylmethanones. (b) Approaches to the synthesis of perfluorinated diarylmethanones. (c) Applications of our strategy.

Traditional approaches to the synthesis of perfluorinated diarylmethanones typically involve transmetalation processes to generate stoichiometric quantities of sensitive perfluoroaryl metallic reagents (Scheme 1b).^9^ For example, Platonov’s group reported a copper-catalyzed approach using perfluoroarylzinc^9a^ as a starting material, while Burton’s team described an alternative method utilizing sodium iodide to iodize perfluoroarenes, followed by two sequential transmetalation steps employing cadmium and copper, that ultimately culminated in the coupling with acyl chlorides.^9b^ However, both of these methods employed sensitive metallic reagents and treated perfluoroarenes as donor synthons, which contradicts their inherently electron-deficient nature. This observation brings to light an exciting realization: perfluoroarenes, given their electron-withdrawing prowess, are primed to act as stellar acceptors in nucleophilic aromatic substitution (S_N_Ar) reactions.^10^

Suzuki’s group^11^ demonstrated the role of *N*-heterocyclic carbenes (NHCs) in catalyzing the nucleophilic acylation of 4-nitroarylfluorides to produce the desired nitrobenzophenone derivatives. Building on this and driven by our enduring interest in S_N_Ar reactions,^10b,10c^ we envisaged the possibility of achieving a mild, facile, and metal-free methodology for synthesizing the coveted functional perfluorinated diarylmethanones.

Leveraging NHCs to effect polarity inversion on aldehydes,^12^ we could generate Breslow intermediates, serving as nucleophiles to engage electron-deficient perfluoroarenes. Consequently, this approach would enable the direct synthesis of perfluorinated diarylmethanones without the need for heavy metals or harsh reaction conditions. Furthermore, we explored the potential applications of this developed strategy in the synthesis of bioisosteric analogs of anticancer drug candidates and the utilization of these compounds as additives for the electrolytes in lithium-ion batteries (LIBs)(Scheme 1c).

We initiated our investigation with benzaldehyde **1a** and pentafluoropyridine **2a** as standard substrates for the NHC-catalyzed S_N_Ar reaction. After systematic optimization of the NHC precatalyst, base, solvent, and temperature, we successfully achieved the optimized conditions (details in Supporting Information Table S2.1–S2.4). The desired product **3a** was efficiently obtained with a 95% NMR yield and further purified by column chromatography to yield **3a** with a 92% isolated yield (Scheme 2).

**Scheme 2:**
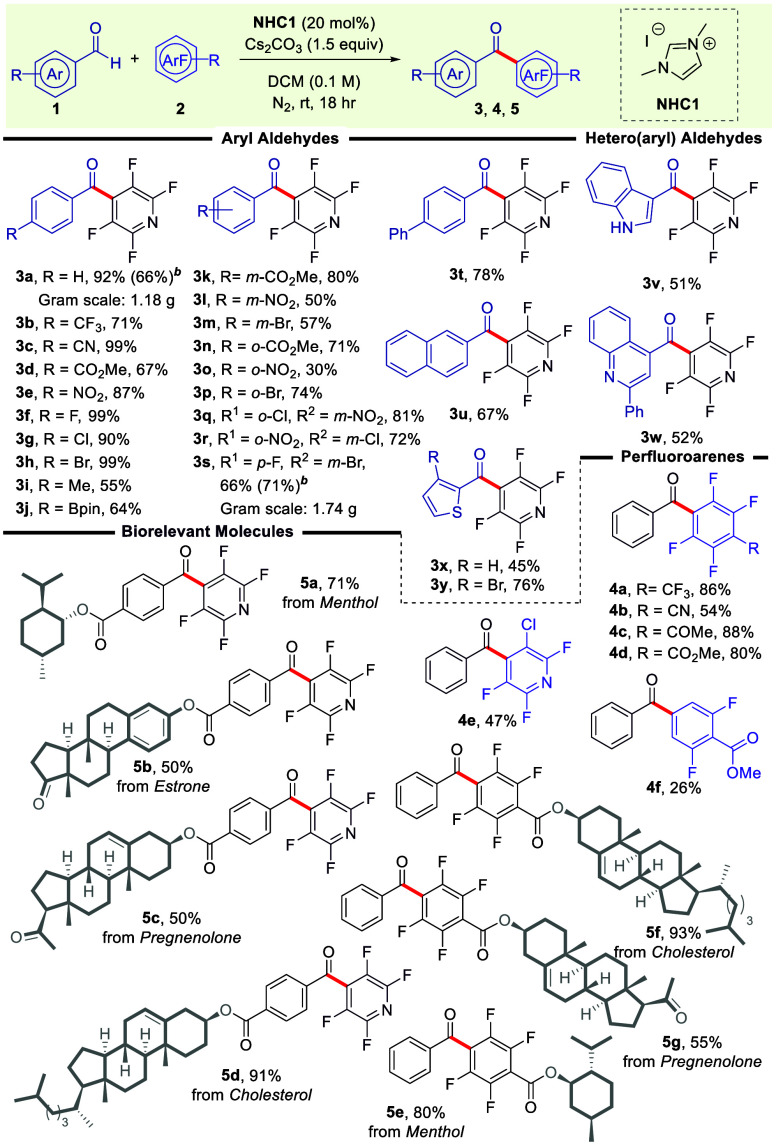
Scope Evaluation of the NHC-Catalyzed Synthesis of Perfluorinated Diarylmethanones Unless otherwise stated, the reaction was conducted with **1** (1.0 equiv, 0.1 mmol), **2** (1.0 equiv, 0.1 mmol), **NHC1** precatalyst (0.2 equiv, 0.02 mmol), and Cs_2_CO_3_ (1.5 equiv, 0.15 mmol) in anhydrous dichloromethane (1 mL, 0.1 M) under a N_2_ environment and stirred at room temperature for 18 h. All yields correspond to purified products. Gram-scale synthesis was conducted using **1** (1.0 equiv, 7.0 mmol), **2** (1.1 equiv, 7.7 mmol), **NHC1** precatalyst (0.2 equiv, 1.4 mmol), and Cs_2_CO_3_ (1.5 equiv, 10.5 mmol) in anhydrous dichloromethane (70 mL, 0.1M) under a N_2_ environment and stirred at room temperature for 60 h.

Having established the optimal reaction conditions, we then explored the generality of the methodology (Scheme 2). Our initial focus was to probe the influence of different substituent patterns on aromatic aldehydes **1** and their subsequent impact on product formation. To our delight, the results demonstrated that regardless of the positional orientation of various functional groups—be it *ortho*, *meta*, or *para*—on the aromatic ring, the outcomes were consistently favorable, yielding products **3a**–**3p** with good to excellent yields. In particular, the integration of electron-withdrawing moieties (**1b**–**1e**) and halogen groups (**1f**–**1h**) in the *para*-position of the aryl aldehyde was well tolerated, and the substrates were seamlessly converted to the desired products **3b**–**3h** in 67–99% yields with notable regioselectivity. The addition of inductively electron-donating groups, such as methyl at the *para*-position of the aryl aldehyde, also led to the desired perfluorinated diarylmethanone (**3i**) in a good yield. Boron-substituted derivatives (**3j**, 64%), acknowledged as versatile synthons in catalytic transformations, exhibited noteworthy compatibility within our established reaction milieu. Notably, *para*-substituted aromatic aldehydes exhibited remarkable versatility in the reaction outcome when subjected to different substituents. Building on these promising outcomes, we directed our attention to assessing the steric effects by relocating the ester, nitro, or bromo functionalities to the *meta* (**1k**–**1m**) and *ortho* (**1n**–**1p**) positions of the aromatic aldehyde. Results unveiled that benzaldehydes hosting these different functionalities displayed relatively weak steric effects, resulting in the desired products in moderate to very good yields, along with commendable regioselectivity. The use of substrates with functional groups has proven efficient in introducing diverse functionalities into the target product, with halogen atoms being notably promising. Our evaluation included assessing the reactivity of disubstituted benzaldehyde precursors containing halogen functionalities (**1q-1s**). Encouragingly, these substrates exhibited remarkable resilience under our reaction conditions, and their interaction with pentafluoropyridine (**2a**) culminated in the generation of target products **3q**–**3s** in commendable yields. Extended conjugated systems, such as biphenyl (**3t**, 78%) and naphthalene (**3u**, 67%), were also amiable. Introducing heterocycles, exemplified by indole **3v** and quinoline derivative **3w**, led to 51% and 52% yields, respectively, while thiophene incorporation (**3x**) showed a modestly lower yield of 45%. However, upon introducing bromine to thiophene (**3y**), the yield was significantly enhanced to 76%. Moreover, the synthetic utility of the protocol was demonstrated by the successful gram-scale synthesis of **3a** (66%, 1.18 g) and **3s** (71%, 1.74 g).

Next, the scope of perfluoroarene (**2**) was investigated. All evaluated substrates participated effectively in the reaction, providing products **4a**–**4f** in 26–88% yields. The chemoselectivity of the developed methodology was further probed upon replacing the pyridine scaffold in the perfluoro arene with a benzene ring. *para*-Substituted perfluorobenzenes featuring electron-withdrawing groups (**2b**–**2e**) were all found to perform well under our optimized conditions, providing **4a**–**4d**, respectively, in yields of 54–88%. We then explored the influence of replacing one fluorine entity with a chlorine atom at the 3-position to assess functional group effects on perfluoroarenes. 3-Chloro-2,4,5,6-tetra fluoropyridine (**2f**) was chosen as the representative substrate for this case. Our findings reveal that, despite chloride being a better leaving group, the addition reactions predominantly take place at the most electron-deficient carbon in the structure (4-position), yielding the major product **4e** (47%). Subsequently, we successfully conducted the reaction using 2,4,6-trifluorobenzoic acid methyl ester (**2g**), forming the desired product **4f**. However, the yield fell short of our expectations; the discrepancy is likely attributed to an enhanced electron density encompassing the ring, which subsequently resulted in a lower reaction rate.

Motivated by the broad functional group tolerance observed with smaller molecules, we have made noteworthy progress in extending this methodology to assess tolerance toward complex natural products and bioactive compounds (Scheme 2). Aryl aldehydes derived from menthol and steroidal hormones (estrone, pregnenolone, and cholesterol) were effectively transformed into the corresponding products **5a**–**5d** with favorable yields. Furthermore, we successfully synthesized the derivatives **5e**–**5g**, containing perfluorocarbons derived from the natural product menthol (**5e**, 80%) and steroid hormones, specifically cholesterol (**5f**, 93%) and pregnenolone (**5g**, 55%). These examples highlight the method’s ability to handle intricate molecules with minimal side reactions, showcasing its promise in tailoring perfluorinated diarylmethanones for enhancing drug performance and therapeutic applications.

Intending to expand the applicability of our established protocol, we embarked on a twofold investigation. First, we endeavored to synthesize bioisosteric analogues of an established drug candidate, coined compound **D1**, which can be used for the inhibition of serine-threonine protein kinase and the sensitization of cancer cells to anticancer agents.^13^ The strategic replacement of hydrogen moieties with fluorine atoms has emerged as a viable approach to bolster a drug’s stability and biological activities.^4,14^ In pursuit of this strategy, we systematically substituted the remaining hydrogen atoms residing in the *ortho*-pyridine ring of **D1** with fluorine atoms, resulting in the synthesis of a fully fluorinated pyridine compound denoted as **PF-D1**, (Scheme 3a). Based on the retrosynthetic analysis, we proposed that **PF-D1** could be accessed from the stitching of the northern aromatic fragment and the southern quinazoline fragment through a Suzuki coupling reaction. The key intermediate **3s** was synthesized from aldehyde **1s** (western fragment) and pentafluoropyridine **2a** (eastern fragment) via our developed NHC-catalyzed S_N_Ar methodology. Utilizing our previously established large-scale NHC-catalyzed S_N_Ar conditions yielded the desired product **3s** (71%, 1.74 g). Subsequently, we conducted a Miyaura borylation reaction by employing dichlorobis(triphenylphosphine)palladium(II) as a catalyst under alkaline conditions. The reaction further progressed through a Suzuki reaction employing bis(benzonitrile)dichloro palladium(II) accompanied by tricyclohexylphosphine as the ligand. The synthesis sequence culminated with the utilization of sodium borohydride to reduce the ketone moiety to the corresponding alcohol, completing the four-step linear sequence synthesis of compound **PF-D1** in an overall yield of 24%. Compared to existing literature methods,^13^ our protocol offers several advantages, including cost-effectiveness and shorter reaction times (for details, see Supporting Information section 3.2). While the pharmacological attributes of **PF-D1** await comprehensive exploration, our study unequivocally demonstrates the versatility and utility of our methodology in constructing perfluorinated diarylmethanones for drug design.

**Scheme 3:**
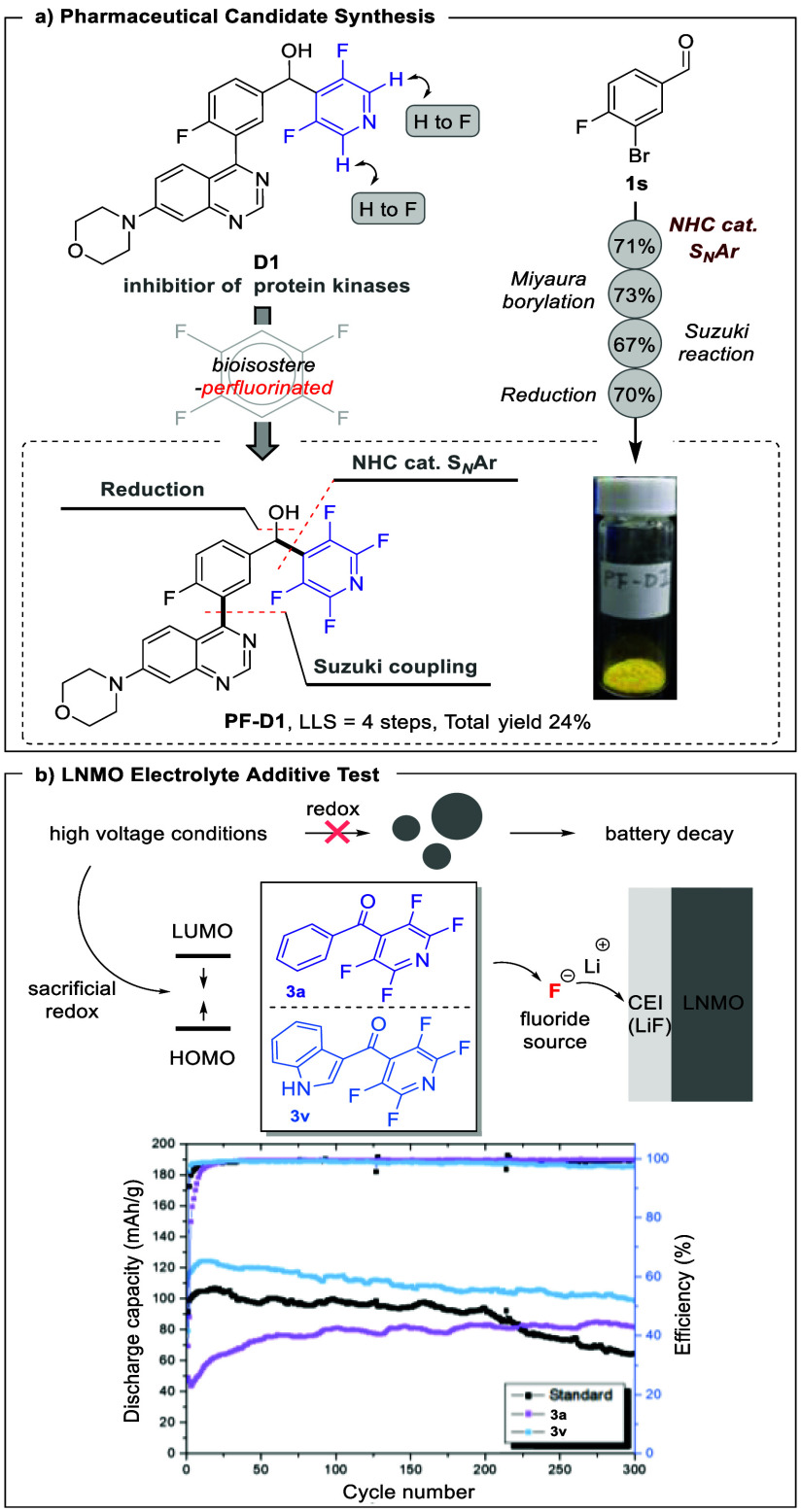
Application of Our Developed Strategy (a) Synthesis of a bioisosteric analogue. (b) Evaluation of perfluorinated diarylmethanones as electrolyte additives for Li-ion battery.

In addition to the successful synthesis of **PF-D1**, we explored the potential of perfluorinated diarylmethanone compounds as additives in LIB electrolytes. LIBs are promising energy storage systems with diverse applications,^5^ with lithium nickel manganese oxide (LNMO) being a standout cathode material due to its high energy density and operating voltage. However, the prevalent electrolyte salt of LNMO, LiPF_6_, faces stability issues at higher voltages, leading to the formation of harmful HF and lithium-ion consumption.^5b^ Previous studies suggest that perfluorinated arenes can act as effective additives in LIB electrolytes, mitigating battery performance degradation by generating fluoride ions and forming a protective layer alongside lithium ions to prevent electrode oxidation and degradation.^15^ Consequently, we conducted a systematic evaluation of the efficiency and discharge capacity of LIBs (Scheme 3b). The (LNMO)//Li half-cells were tested both in the absence and presence of 0.05 wt % additive compound **3a** or **3v**, selected from our substrate scope. Without the additive (standard), our findings show a noticeable decrease in discharge capacity (64.8 mAh/g at 1C after 300 cycles). Conversely, incorporating these additives (**3a** or **3v**) led to a significant improvement in discharge capacity, resulting in values of 81.6 mAh/g for **3a** and 97.2 mAh/g for **3v** at 1C after 300 cycles. Additionally, the Coulombic efficiency (CE) remained nearly unchanged after 300 cycles. Both of these results indicate the preservation of LIB efficiency and the effective mitigation of performance degradation, potentially stemming from the formation of the solid electrolyte interface (SEI) layer on the cathode.^15a^ We infer from this that perfluorinated diarylmethanone additives might act as sacrificial redox agents, bolstering the overall performance of the LIBs. Our research confirms the potential of perfluorinated diarylmethones as viable electrolyte additives for LNMO-based LIBs.

In conclusion, we have successfully devised a mild, facile, metal-free, and highly regio- and chemoselective umpolung S_N_Ar reaction utilizing *N*-heterocyclic carbenes (NHCs) as catalysts, enabling the synthesis of perfluorinated diarylmethanones. This approach allows direct conversion of aromatic aldehydes and perfluoroarenes into the target compounds without the need for sensitive metallic reagents. Notably, this reaction exhibits broad substrate compatibility, excellent functional group tolerance, and mild reaction conditions. Practical applications include modifying pharmaceutical compounds, as illustrated by transforming a drug candidate into the perfluorinated bioisostere, **PF-D1**. Additionally, we investigated the use of perfluorinated diarylmethanones **3a** and **3v** as electrolyte additives in lithium-ion batteries (LIBs), leading to enhanced cycling stability and maintenance of excellent efficiency. We posit that our method will unlock possibilities in the realm of perfluoroarene chemistry, extending its applicability in diverse fields of application.

## Data Availability

The data underlying this study are available in the published article and its Supporting Information

## Supporting Information Available

Experimental procedure, bioisotere synthesis, experimental procedure for the LIB test, proposed mechanism, reference and NMR spectra (PDF)
FAIR data, including the primary NMR FID files, for compounds **2c′**, **2j**, **3a**–**3y**, **4a**–**4f**, **5a**–**5g**, **6**, **7**, **NCH1**, **NCH4**, and **PF**-**D1** (ZIP)
