# Supplementary material for: FluoroFusion: NHC-Catalyzed Nucleophilic Aromatic Substitution Reaction Unveils Functional Perfluorinated Diarylmethanones
Source: Org Lett. 2024 Mar 8;26(11):2338–42. doi: 10.1021/acs.orglett.4c00677 (PMC10964231; doi:10.1021/acs.orglett.4c00677)
Supplement: Supplementary file 1 — ol4c00677_si_001.zip [file ol4c00677_si_001.zip › NHC SNAr OL/NHC SnAr Supporting Information OL.docx]

Supplementary Information

**FluoroFusion: NHC-Catalyzed Nucleophilic Aromatic Substitution Reaction Unveils Functional Polyfluorinated Diarylmethanones**

Cheng-Lin Chan^1,2^, Shao-Chi Lee^3^, Pei-Shan Lin^1^, Radyn Vanessa Phaz P. Tapales^1,2^, Jia-Syuan Li^1^, Chun-An Lai^1^, Jyh-Tsung Lee^1^, Chien-Hung Li^4^ and Hsuan-Hung Liao*^,1,4,^

^1^*Department of Chemistry, National Sun Yat-sen University, Kaohsiung, Taiwan (R.O.C.)*

^2^*Department of International PhD Program for Science, National Sun Yat-sen University, Kaohsiung, Taiwan (R.O.C.)*

^3^*KAUST Catalysis Center (KCC), King Abdullah University of Science and Technology (KAUST), Thuwal, Saudi Arabia*

^4^*Department of Applied and Medicinal Chemistry, Kaohsiung Medical University, Kaohsiung, Taiwan (R.O.C.)*

**Corresponding author. Email:* [*hsuan-hung.liao@mail.nsysu.edu.tw*](mailto:hsuan-hung.liao@mail.nsysu.edu.tw)

**Contents**

[1. General information 2](#_Toc159337613)

[1.1 Pre-treatment of experiments, solvents, and reagents 2](#_Toc159337614)

[1.2 Chromatography, data analysis, and collection 2](#_Toc159337615)

[1.3 Instrument catalogue 2](#_Toc159337616)

[1.4 Purchasing reagent 3](#_Toc159337617)

[1.5 Synthesized compound 10](#_Toc159337618)

[2. Experimental procedure 13](#_Toc159337619)

[2.1. Preparation of substrates 13](#_Toc159337620)

[2.2. Preparation of *N*-heterocyclic carbene (NHCs) 15](#_Toc159337621)

[2.3. Standard condition of NHC-catalyzed S*_N_*Ar reaction 17](#_Toc159337622)

[2.4 General procedures of NHC-Catalyzed S*_N_*Ar reaction 19](#_Toc159337623)

[General procedure A 19](#_Toc159337624)

[General procedure B 19](#_Toc159337625)

[2.5 Substrate scopes overview of NHC-Catalyzed S*_N_*Ar reaction 20](#_Toc159337626)

[2.6 Substrate scopes data of NHC-Catalyzed S*_N_*Ar reaction 23](#_Toc159337627)

[2.7 Large-scale syntheses of NHC-Catalyzed S*_N_*Ar reaction 61](#_Toc159337628)

[3. Synthesis of bioisostere 62](#_Toc159337629)

[3.1 Procedure of synthesis 62](#_Toc159337630)

[3.2 Procedure comparison 65](#_Toc159337631)

[4. Experimental procedure of LIBs test 66](#_Toc159337632)

[4.1 Preparation of LNiMnO cathode material pole piece and electrolyte 66](#_Toc159337633)

[4.2 Cycle Life Test 67](#_Toc159337634)

[5. Proposed mechanism 67](#_Toc159337635)

[6. Reference 68](#_Toc159337636)

[7. NMR spectra 69](#_Toc159337637)

1. General information

1.1 Pre-treatment of experiments, solvents, and reagents

**Glassware** All glassware has been oven-dried after cleaning with a cleaning machine, Steelco LAB 500CL.

**Solvents** All solvents were purchased from suppliers. Unless otherwise stated, the following ACS grade solvents (Acetonitrile, 1,4-Dioxane, Diethyl Ether, DCM, DMF, THF, Toluene) were stored over microwave-activated 3Å molecular sieves for at least one night and transferred into anhydrous engineering alumina column drying system (Vigor Gas Purification Technologies Co., Ltf, VSPS-7) before use.

**Chemicals** To maintain the activity, sensitive compounds such as NHC catalysts and aldehyde were stored in the glovebox. Other chemicals were directly used as received and were always filled with nitrogen before storage and twined with parafilm carefully. See section **S1.4** for the complete supplier list of each chemical.

1.2 Chromatography, data analysis, and collection

**Thin-layer Chromatography (TLC)** Merck aluminium-backed sheets coated with 60F_254_ silica gel. The silica plate was visualized using a UV lamp (λ_max_ = 254 nm).

**Column chromatography** Column chromatography was carried out using KM3 scientific silica gel (45 – 75 µm) purchased from KM3 scientific.

1.3 Instrument catalogue

**Nuclear Magnetic Resonance (NMR)** ^1^H-, ^13^C- and ^19^F- Nuclear Magnetic Resonance (NMR) spectra were used to identify the structure of starting materials and products by using Bruker Avance 300 MHz, Jeol ECZS 400 MHz, Bruker Avance 500 MHz and Jeol ECZR 600 MHz. Coupling constants are abridged as follows: s = singlet, d = doublet, t = triplet, q = quartet, quin = quintet, sext = sextet, sept = septet, m = multiplet, dd = doublet of doublet.

**Gas Chromatography–Mass Spectrometry (GC-MS) The operation method was set as follows**: 1.0 mL sample was injected by auto-sampler in a split mode (100:1) with 0.5 mL air gap into the GC-MS system consisting of an Agilent 8860 gas chromatograph, an Agilent 5977B mass selective detector, and Agilent 7693A autoinjector. Gas chromatography was performed on a 30 m HP-5MS with 0.25 mm inner diameter (I.D.) and 0.25 mm film thickness with an initial injection temperature of 50 ºC to 300 ºC, MSD transfer line of 280 ºC, and the ion source adjusted to 230 ºC. The helium carrier gas was set at a constant flow rate of 1.197 ml min-1. The mass spectrometer was operated in positive electron impact mode (EI), with ionization energy in the m/z 50 – 550 scan range. The spectra of all chromatogram peaks were evaluated using the MSD Chemstation.

**High-Resolution Mass Spectra (HRMS)** Jeol AccuTOF GCx-plus / Shimadzu QP2020

**Table S1.1** Supplier of equipment and instruments.

| Equipment | Supplier |
| --- | --- |
| Electronic balance | *Shimadzu* UW2200H/ ATX224 |
| Hot plate stirrer | *Corning* PC-420D |
| Immersion cooler | *Panchum* IC-9090 |
| Pump of rotary evaporator | *KNF Laboport* N820.3FT.18 |
| Rotary evaporator | *Heidolph* Hei-Vap Core HL G3 |
| Refrigerated circulator bath | *Panchum* CC-300 |
| Ultra-low temp. reaction bath | *Panchum* UR-8500 |
| Vacuum pump | *Edwards* RV5 |
| Instruments | Supplier |
| Glovebox | *Vigor* SG1200/750TS-F |
| GC-MS  (Gas Chromatograph Mass Spectrometer) | *Agilent* 5977B |

1.4 Purchasing reagent

**Table S1.2** Supplier of solvents.

| Solvent | Supplier | Solvent | Supplier |
| --- | --- | --- | --- |
| ACS Acetone | Duksan | HPLC Acetonitrile | J.T. Baker |
| ACS Acetonitrile | J.T. Baker | HPLC DMF | Macron |
| ACS Benzene | Echo | HPLC Ethyl acetate | Merck |
| ACS Chloroform | Acros | HPLC Hexane | Echo |
| ACS Dimethyl sulfoxide | UR | HPLC Isopropanol | Echo |
| ACS Diethyl ether | Duksan | ACS Hexane | Duksan |
| ACS Dichloromethane | Macron /Duksan | ACS Methanol | Macron |
| ACS Ethanol | J.T. Baker | ACS THF | Macron |
| ACS Ethyl acetate | Macron | ACS Toluene | Echo |

**Table S.1.3** Supplier of solvents in solvent purification systems.

| Solvent | Supplier | Solvent | Supplier |
| --- | --- | --- | --- |
| Acetonitrile | J.T. Baker | Dichloromethane | Macron |
| DMF | Macron | THF | Macron |
| 1,4-Dioxane | J.T. Baker | Toluene | J.T. Baker |
| Diethyl ether | Echo |  |  |

**Table S1.4** Supplier of deuterated solvents.

| Solvent | Supplier | Solvent | Supplier |
| --- | --- | --- | --- |
| Acetonitrile-d_3_ | Sigma-Aldrich | DMSO-d_6_ | Sigma-Aldrich |
| Benzene-d_6_ | Sigma-Aldrich | Dichloromethane-d_2_ | Sigma-Aldrich |
| Chloroform-d_1_ | Merck | Methanol-d_4_ | Sigma-Aldrich |

**Table S1.5** Supplier of commercially available *N*-heterocyclic carbenes (NHCs).

| Structure | Name | CAS# | Supplier |
| --- | --- | --- | --- |
|  | 1,3-Bis(2,6-diisopropylphenyl)-1*H*-imidazol-3-ium chloride | 250285-32-6 | BLD |
|  | 1,3-Bis(2,6-diisopropylphenyl)-4,5-dihydro-1*H*-imidazol-3-ium chloride | 258278-25-0 | BLD |
|  | 1,3-Diisopropyl-1*H*-imidazol-3-ium tetrafluoroborate | 286014-34-4 | BLD |
|  | 1,3-Di-tert-butyl-1*H*-imidazol-3-ium tetrafluoroborate | 263163-17-3 | BLD |

**Table S1.6** Supplier of commercially available aldehydes.

| No. | Structure | Name | CAS Number | Supplier |
| --- | --- | --- | --- | --- |
| **1a** |  | Benzaldehyde | 100-52-7 | Nova |
| **1b** |  | 4-(Trifluoromethyl)benzaldehyde | 455-19-6 | Combi-Blocks |
| **1c** |  | 4-Formylbenzonitrile | 105-07-7 | Merck |
| **1e** |  | 4-Nitrobenzaldehyde | 555-16-8 | BLD Pharm |
| **1f** |  | 4-Fluorobenzaldehyde | 459-57-4 | Aldrich |
| **1g** |  | 4-Chlorobenzaldehyde | 104-88-1 | Acros |
| **1h** |  | 4-Bromobenzaldehyde | 1122-91-4 | vetec |
| **1i** |  | 4-Methylbenzaldehyde | 104-87-0 | BLD Pharm |
| **1l** |  | 3-Nitrobenzaldehyde | 99-61-6 | Acros |
| **1m** |  | 3-Bromobenzaldehyde | 3132-99-8 | Nova |
| **1o** |  | 2-Nitrobenzaldehyde | 552-89-6 | Alfa Aesar |
| **1p** |  | 2-Bromobenzaldehyde | 6630-33-7 | Alfa Aesar |
| **1q** |  | 2-Chloro-5-nitrobenzaldehyde | 6361-21-3 | Alfa Aesar |
| **1r** |  | 5-Chloro-2-nitrobenzaldehyde | 6628-86-0 | BLD Pharm |
| **1s** |  | 3-Bromo-4-fluorobenzaldehyde | 77771-02-9 | BLD Pharm |
| **1t** |  | [1,1'-Biphenyl]-4-carbaldehyde | 3218-36-8 | BLD Pharm |
| **1v** |  | 1*H*-Indole-3-carbaldehyde | 487-89-8 | Alfa Aesar |
| **1x** |  | Thiophene-2-carbaldehyde | 98-03-3 | Alfa Aesar |
| **1y** |  | 3-Bromothiophene-2-carbaldehyde | 930-96-1 | BLD Pharm |
| **1a’** |  | 4-Methoxybenzaldehyde | 123-11-5 | Alfa Aesar |
| **1b’** |  | 3-Nitrothiophene-2-carbaldehyde | 58963-75-0 | BLD Pharm |
| **1c’** |  | 6,6-Dimethylbicyclo[3.1.1]hept-2-ene-2-carbaldehyde | 564-94-3 | BLD Pharm |
| **1d’** |  | 2-Phenylacetaldehyde | 122-78-1 | Alfa Aesar |

**Table S1.7** Supplier of commercially available polyfluoroarenes.

| No. | Structure | Name | CAS Number | Supplier |
| --- | --- | --- | --- | --- |
| **2a** |  | Pentafluoropyridine | 700-16-3 | BLD Pharm |
| **2b** |  | 1,2,3,4,5-Pentafluoro-6-(trifluoromethyl)benzene | 434-64-0 | BLD Pharm |
| **2c** |  | Pentafluorobenzonitrile | 773-82-0 | BLD Pharm |
| **2d** |  | 2,3,4,5,6-Pentafluoroacetophenone | 652-29-9 | BLD Pharm |
| **2e** |  | Methyl-pentafluorobenzoate | 36629-42-2 | TCI |
| **2f** |  | 3-Chloro-2,4,5,6-tetrafluoropyridine | 1735-84-8 | BLD Pharm |
| **2a’** |  | 1,2,3,4,5-Pentafluorobenzene | 363-72-4 | BLD Pharm |
| **2b’** |  | 1,3,5-Trifluorobenzene | 372-38-3 | BLD Pharm |

**Table S1.8** Supplier of commercially available reagent.

| Name | CAS Number | Supplier |
| --- | --- | --- |
| Bis(triphenylphosphine)-palladium (II) dichloride | 13965-03-2 | Nova |
| Bis(pinacol)diborane | 73183-34-3 | Matrix Scientific |
| Caesium carbonate | 534-17-8 | Alfa Aesar |
| Caesium fluoride | 13400-13-0 | Nova |
| 4-(4-Chloroquinazolin-7-yl) morpholine | 1334602-74-2 | BLD Pharm |
| 4-Dimethylaminopyridine | 1122-58-3 | Merck |
| Methyl iodide | 74-88-4 | Jassen |
| 1-Methyl-1*H*-imidazole | 616-47-7 | ACROS |
| *N*, *N*'-Dicyclohexylcarbodiimide | 538-75-0 | Merck |
| Palladium (II) chloride | 7647-10-1 | TCI |
| Potassium acetate | 127-08-2 | Sigma-Aldrich |
| Potassium carbonate | 584-08-7 | Duksan |
| Sodium borohydride | 16940-66-2 | TCI |
| Sodium carbonate | 497-19-8 | Shimakyus |
| Tricyclohexylphosphine | 2622-14-2 | Nova |
| Triethylamine | 121-44-8 | Alfa Aesar |

1.5 Synthesized compound

**Table S1.9** Synthesized aryl aldehydes and polyfluoroarenes.

| No. | Structure | Name | Reference |
| --- | --- | --- | --- |
| **1d** |  | Methyl-4-formylbenzoate | [Ref](https://chemistry-europe.onlinelibrary.wiley.com/doi/abs/10.1002/1099-0690(200006)2000:11%3C2095::AID-EJOC2095%3E3.0.CO;2-J) |
| **1j** |  | 4-(4,4,5,5-Tetramethyl-1,3,2-dioxaborolan-2-yl)-benzaldehyde | [Ref](https://pubs.acs.org/doi/full/10.1021/jo070084v) |
| **1k** |  | Methyl-3-formylbenzoate | [Ref](https://onlinelibrary.wiley.com/doi/full/10.1002/anie.202004747) |
| **1n** |  | Methyl-2-formylbenzoate | [Ref](https://www.sciencedirect.com/science/article/pii/S022352341500197X) |
| **1u** |  | 2-Naphthaldehyde | [Ref](https://www.tandfonline.com/doi/full/10.1080/00397911.2022.2134799?casa_token=BThdCzfU1zMAAAAA:udtHhm56zDOD-3sBRQ41zKO7DKV69iIx8PlAzrQvpOGcy-mQs_eyuEBHUQfWTm69fsI62u3NjiwNEQ) |
| **1w** |  | 2-Phenylquinoline-4-carbaldehyde | [Ref](https://pubs.acs.org/doi/full/10.1021/acs.joc.9b00035) |
| **2g** |  | Methyl-2,4,6-trifluorobenzoate | [Ref](https://www.sciencedirect.com/science/article/pii/S0223523411000493) |
| **2c’** |  | 4-Bromophenyl 2,3,4,5,6-pentafluorobenzoate | [link](#P2c) |

**Table S1.10** Synthesized bioactive analogues.

| No. | Structure | Reference |
| --- | --- | --- |
| **1z** |   from *menthol* | [Ref](https://pubs.acs.org/doi/full/10.1021/acs.orglett.9b04495) |
| **1aa** |   from *estrone* | [Ref](https://onlinelibrary.wiley.com/doi/full/10.1002/anie.202117843) |
| **1ab** |   from *pregnenolone* | [Ref](https://pubs.acs.org/doi/full/10.1021/acs.orglett.9b04495) |
| **1ac** |   from *cholesterol* | [Ref](https://pubs.acs.org/doi/full/10.1021/acs.orglett.2c01648) |
| **2h** |   from *menthol* | [Ref](https://onlinelibrary.wiley.com/doi/full/10.1002/anie.202010492) |
| **2i** |   from *cholesterol* | [Ref](https://onlinelibrary.wiley.com/doi/full/10.1002/anie.202010492) |
| **2j** |   from *pregnenolone* | [link](#P6c) |

2. Experimental procedure

2.1. Preparation of substrates

Methylation

To a solution of benzoic acid (6.0 mmol, 1.0 equiv) in anhydrous DMF (24 mL, 0.25 M) was added finely ground potassium carbonate (1.7 g, 12 mmol, 2.0 equiv) and iodoethane (0.9 mL, 15 mmol, 2.5 equiv) under nitrogen atmosphere. The reaction stirred at rt until completion was indicated by TLC before being diluted with water and extracted with anhydrous ether. The combined organic layers were then washed with brine, dried over MgSO_4_, filtered, and dried under high vacuum. The crude residue was purified by flash column chromatography (height of packing silica gel: 7.0 cm, Hexane/ethyl acetate: 6/1) to afford desire product.

All characterization data for each compound **1d**, **1k**, **1n** and **2g** are consistent with reported literatures.

Boronic esterification

To the solution of 4-Formylbenzeneboronic acid (1.5 g, 10.0 mmol) in anhydrous ether (100 mL) was added pinacol (1.2 g, 10.0 mmol) and MgSO_4_ (150 mg) under nitrogen atmosphere. The mixture was stirred for overnight at rt. After monitoring by TLC, the MgSO_4_ was filtered. The solution was washed with water and dried over MgSO_4_. The crude residue was purified by flash column chromatography (height of packing silica gel: 7.0 cm, Hexane/ethyl acetate: 6/1) to afford desire product.

The characterization data for compound **1j** is consistent with reported literature.

Reduction and oxidation

To the solution of lithium aluminium hydride (759.0 mg, 20.0 mmol, 4.0 equiv) in anhydrous ether (10.0 mL) was added aryl carboxylic acid solution (5.0 mmol in 10.0 mL of anhydrous THF) under nitrogen atmosphere. The reaction mixture was stirred at 80 ℃ for overnight. The reaction was cooled to 0 ℃ and quenched with water. The mixture was extracted with 1.0 M HCl_(aq)_. The organic layer was dried over MgSO_4_ and evaporated to afford desire alcohol. The residue was further dissolved in anhydrous DCM. The solution was added to a flask which is equipped with MnO_2_ (4.3 g, 50 mmol, 10.0 equiv). The reaction was stirred for overnight at room temperature. After monitoring by TLC, the reaction was filtered, extracted with DCM, and purified by column chromatography to afford desire product.

The characterization data for each compound **1u**, **1w** is consistent with reported literature.

Steglich esterification

Thionyl chloride (1.8 mL, 25 mmol, 5.0 equiv) was added to the solution of benzoic acid (5.0 mmol, 1.0 equiv) in anhydrous DCM (20 mL) under nitrogen atmosphere. The reaction mixture was stirred under reflux for 5 h. After completion of the reaction, the excess of thionyl chloride and DCM were removed by high vacuum to give crude acyl chloride. Subsequently, the crude acyl chloride was resolved in 10 mL anhydrous DCM and was slowly added to the solution of triethylamine (2.0 mL, 15.0 mmol, 3.0 equiv) and R^2^OH (5.0 mmol, 1.0 equiv) with 10 mL anhydrous DCM at 0 ºC under nitrogen atmosphere. Then the reaction mixture was stirred for 2 - 5 h at room temperature. After monitoring by TLC, the reaction was quenched with water, extracted with DCM and water. The organic layer was dried over anhydrous MgSO_4_ and evaporated in high vacuum. The residue was purified by column chromatography to provide the desired product.

The characterization data for each compound **1z**, **1aa**, **1ab**, **1ac**, **2h**, and **2i** is consistent with reported literature.

**Perfluorophenyl 4-bromobenzoate**, **2c’**

**^1^H NMR** (300 MHz, CDCl_3_) *δ* 8.06 (d, *J* = 8.7 Hz, 1H), 7.70 (d, *J* = 8.7 Hz, 1H) ppm.

**^13^C NMR** (101 MHz, CDCl_3_) *δ* 162.0, 142.6 – 140.0 (m), 141.0 – 139.1 (m), 138.6 – 136.7 (m), 132.4, 132.1, 130.3, 125.8. ppm.

**^19^F NMR** (282 MHz, CDCl_3_) *δ* -151.82 – -153.38 (m), -157.54 (t, *J* = 21.6 Hz), -162.09 (dd, *J* = 21.8, 17.2 Hz) ppm.

[*See NMR spectra*](#S2c)

**(8*S*,9*S*,10*R*,13*S*,14*S*,17*S*)-17-Acetyl-10,13-dimethyl-2,3,4,7,8,9,10,11,12,13,14,15,16,17-tetradecahydro-1H-cyclopenta[*a*]phenanthren-3-yl 2,3,4,5,6-pentafluorobenzoate**, **2j**


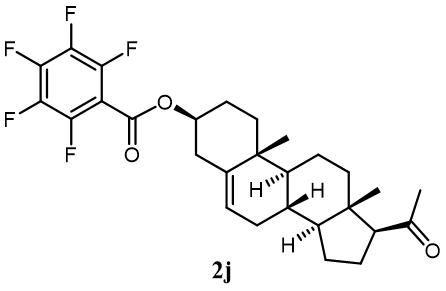


**^1^H NMR** (300 MHz, CDCl_3_) *δ* 5.45 – 5.43 (m, 1H), 4.97 – 4.86 (m, 1H), 2.57 – 2.45 (m, 3H), 2.20 – 1.90 (m, 8H), 1.80 – 1.41(m, 8H), 1.25 – 1.17 (m, 3H), 1.05 (s, 4H), 0.64 (s, 3H) ppm.

**^13^C NMR** (101 MHz, CDCl_3_) *δ* 209.3, 158.2, 146.5 – 143.7 (m), 144.3 – 141.4 (m), 138.9, 138.8 – 136.1 (m), 122.9, 108.9 – 108.6 (m), 63.5, 56.7, 49.8, 43.8, 38.7, 37.7, 36.8, 36.5, 31.69, 31.67, 31.4, 27.5, 24.4, 22.7, 20.9, 19.1, 13.1. ppm.

**^19^F NMR** (282 MHz, CDCl_3_) *δ* -138.6 – -138.8 (m), -149.2 (tt), -160.4 – -160.6 (m) ppm.

*[See NMR spectra](#S6c)*

2.2. Preparation of *N*-heterocyclic carbene (NHCs)

1,3-Dimethyl-1H-imidazol-3-ium iodide, NHC1

According to a literature procedure^[[1]](#footnote-1)^, an oven-dried flask containing a stirring bar was cooled to room temperature under high vacuum and refilled with nitrogen. Methyl iodide (851.6 mg, 6.0 mmol, 1.2 equiv) was added in portions to a DCM (1.0 mL) solution of the 1-methyl-1*H*-imidazole (410 mg, 5.0 mmol, 1.0 equiv) over the course of 30 min (**Caution**: exothermic reaction) at 0 ℃. The reaction mixture was stirred for further 1 hr, after which the mixture was concentrated and dried under high vacuum to afford carbene **NHC1** (1030.4 mg, 4.6 mmol, 92%) as a white solid.

**^1^H NMR** (300 MHz, MeOD) *δ* 9.24 (s, 1H), 7.87 (s, 2H), 4.20 (s, 6H) ppm.

**^13^C NMR** (101 MHz, CDCl_3_) *δ* 137.7, 123.4, 37.1 ppm.

*[See NMR spectra](#SNHC1)*

3-(2,6-Diisopropylphenyl)-5,6,7,8-tetrahydro-4*H*-cyclohepta[*d*]thiazol-3-ium perchlorate, NHC4

According to the literature^[[2]](#footnote-2)^, NaOH (0.4 g, 11 mmol) was dissolved to a solution of 2,6-diisopropylamine (1.5 mL, 11 mmol) in DMSO (5 mL). CS_2_ was added dropwise at 0 ℃ and the media was allowed to stir at room temperature for 1 hr. 2-Bromocycloheptan-1-one was then added by portion, and the solution was stirred overnight at room temperature. The precipitate formed by the addition of water (10 mL) was filtrated, washed with water, and then suspended in absolute ethanol (13 mL). Concentrated HCl (0.5 mL) was then added, and the solution was heated to reflux for 1 hr. After cooling and the addition of water (10 mL), a brown solid was filtrated. The dithiocarbamate was obtained as a beige solid, which was then dissolved in acetic acid (25 mL) and treated with 30% H_2_O_2_ (1.6 mL, 19.1 mmol) for 1 hr at room temperature. Remove most of the solvents, and the residue was dissolved in methanol (5 mL). To this solution, a solution of NaClO_4_ (3.0 g, MeOH 140 mL, and water 70 mL) was added at 0 °C and stirred for 1 hr at the same temperature. After evaporation of solvents and trituration with Et_2_O, the product **SN_4_** was obtained as a beige solid (1.7 g, 4.1 mmol, 40%).

An oven-dried flask containing a stirring bar was cooled to room temperature under high vacuum and refilled with nitrogen. A solution of the **SN_4_** (2.2 g, 6.5 mmol, 1.0 equiv) in glacial acetic acid (27 mL, 0.25 M) was treated under a water bath cooling dropwise with H_2_O_2_ (30%, 2.0 mL, 2.1 mmol, 3.3 equiv) and was stirred for 1 hr at that temperature. After 1 hr, the volatiles were removed, the residue was dissolved in MeOH (5.0 mL), and a solution of sodium perchlorate monohydrate (3.7 g, 26.6 mmol, 4.1 eq) in MeOH/H2O = 2:1 (23.0 mL) was added at room temperature. After stirring for 10 min, the volume of the solvent was reduced to half. The solid was collected by suction filtration and was washed with H_2_O (40.0 mL). The following recrystallization with MeOH afforded the product **NHC4** as a colorless solid.

**^1^H NMR** (400 MHz, CDCl_3_) *δ* 9.7 (s, 1H), 7.61 (t, *J* = 7.8 Hz, 1H), 7.37 (d, *J* = 7.8 Hz, 2H), 3.19 − 3.15 (m, 2H), 2.57 − 2.53 (m, 2H), 2.06 (sept, *J* = 6.9 Hz, 2H), 1.97 − 1.91 (m, 4H), 1.19 (d, *J* = 6.8 Hz, 6H), 1.17 (d, *J* = 6.8 Hz, 6H) ppm.

**^13^C NMR** (400 MHz, CDCl_3_) *δ* 155.7, 148.5, 144.9, 141.0, 141.0, 132.5, 132.1, 125.2, 30.8, 28.8, 28.2, 27.3, 26.6, 25.5, 24.8, 23.2 ppm.

[*See NMR spectra*](#SNHC4)

2.3. Standard condition of NHC-catalyzed S*_N_*Ar reaction

Prepare two over-dried tubes (5.0 mL) with a screw cap containing a stirring bar that was charged into the nitrogen-filled glovebox. A tube was added **1aa** (10.6 mg, 0.1 mmol, 1.0 equiv), **2a** (16.9 mg, 0.1 mmol, 1.0 equiv), and anhydrous solvent (1.0 mL, 0.1 M). The solution was added into the other tube, which contained an **NHC** catalyst (0.02 mmol, 20 mol%) and base (0.15 mmol, 1.5 equiv). The sealed tube was taken out of the glove box and stirred for 18 hr at room temperature. After the reaction was completed, the crude was filtered by flash column chromatography with ethyl acetate (the height of packing silica gel: 3.0 – 4.0 cm). After removing the solvent by suction filtration, 14.6 mg trifluoromethylbenzene was added to configure the NMR sample to calculate the spectrum yield.

Table S2.1 Screening of NHC-catalyst

^[a]^The spectrum yield was determined by ^19^F NMR using trifluoromethylbenzene as an internal standard. ^[b]^Isolated yield.

Table S2.2 Screening of solvent

^[a]^The spectrum yield was determined by ^19^F NMR using trifluoromethylbenzene as an internal standard. ^[b]^Isolated yield.

Table S2.3 Screening of base

^[a]^The spectrum yield was determined by ^19^F NMR using trifluoromethylbenzene as an internal standard. ^[b]^Isolated yield.

Table S2.4 Background condition test

^[a]^The spectrum yield was determined by ^19^F NMR using trifluoromethylbenzene as an internal standard. ^[b]^Isolated yield.

2.4 General procedures of NHC-Catalyzed S*_N_*Ar reaction

General procedure A

Prepare two over-dried tubes (5.0 mL) with a screw cap containing a stirring bar that was charged into the nitrogen-filled glovebox. A tube was added aromatic aldehyde (0.1 mmol, 1.0 equiv), perfluoroarene (0.1 mmol, 1.0 equiv), and anhydrous DCM (1.0 mL, 0.1 M). The DCM solution was added into the other tube, which contained **NHC1** precatalyst (4.5 mg, 0.02 mmol, 20 mol%) and Cs_2_CO_3_ (48.8 mg, 0.15 mmol, 1.5 equiv). The sealed tube was taken out of the glovebox and stirred for a further 18 hr. After the reaction was completed, the crude was purified by silica gel column chromatography (the height of packing silica gel: 6.0 – 8.0 cm, appropriate eluent) or preparative TLC on silica gel to afford the products.

General procedure B

In the nitrogen-filled glovebox, an oven-dried pear-shaped flask (10.0 mL) with a septum and 250 ml reaction round bottom bottle equipped with a stir bar was prepared. Aryl aldehyde (7.0 mmol, 1.0 equiv) and perfluoroarene (7.7 mmol, 1.1 equiv) were added into the pear-shaped flask followed by septum covering. The round bottom bottle was sealed by septum after the addition of **NHC1** catalyst (313.6 mg, 1.4 mmol, 20 mol%) and Cs_2_CO_3_ (3423.0 mg, 10.5 mmol, 1.5 equiv). Both containers were taken out of the glovebox. Under nitrogen gas, 50 mL anhydrous DCM was added into the reaction round bottom bottle as well as 5 mL into the heart-shaped bottle. The mixture of **1** and **2** in DCM was injected into the reaction round bottom bottle. Wash the residue three times (5 mL DCM per time). The reaction was stirred for a further 60 hr. After the reaction is completed, wash the reaction with DCM and water, dry it with MgSO_4_ then remove DCM. Purified by silica gel column chromatography (the height of packing silica gel: 8.0 – 10.0 cm) followed by removing the solvent in a vacuum to afford the products.

2.5 Substrate scopes overview of NHC-Catalyzed S*_N_*Ar reaction

Table S2.5 Substrate scopes of compound 3

|   [3a](#P3a) |   [3b](#P3b) |   [3c](#P3c) |   [3d](#P3d) |
| --- | --- | --- | --- |
|   [3e](#P3e) |   [3f](#P3f) |   [3g](#P3g) |   [3h](#P3h) |
|   [3i](#P3i) |   [3j](#P3j) |   [3k](#P3k) |   [3l](#P3l) |
|   [3m](#P3m) |   [3n](#P3n) |   [3o](#P3o) |   [3p](#P3p) |
|   [3q](#P3q) |   [3r](#P3r) |   [3s](#P3s) |   [3t](#P3t) |
|   [3u](#P3u) |  |  |  |
| Heteroaryl aldehyde | | | |
|   [3v](#P3v) |   [3w](#P3w) |   [3x](#P3x) |   [3y](#P3y) |

Table S2.6 Substrate scopes of compound 4

| ****  [**4a**](#P4a) | ****  [**4b**](#P4b) | **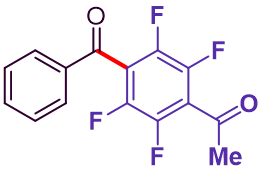**  [**4c**](#P4c) | ****  [**4d**](#P4d) |
| --- | --- | --- | --- |
| ****  [**4e**](#P4e) | ****  [**4f**](#P4f) |  |  |

Table S2.7 Biorelevant molecules syntheses of compound 7

|   [**5a**](#P5a) |   [**5b**](#P5b) |
| --- | --- |
|   [**5c**](#P5c) |   [**5d**](#P5d) |
|   [**5e**](#P5e) |   [**5f**](#P5f) |
|   [**5g**](#P5g) |  |

Table S2.8 Substrate scopes limitations

| Started material | Result | Started material | Result |
| --- | --- | --- | --- |
| **1a’** | No Reaction  (electron donating group) | **1b’** | No reaction  (low solubility) |
| **1c’** | Trace  (low conversion) | **1d’** | Messy reaction  (aldol condensation side reaction) |
| **1e’** | Messy reaction  (aldol condensation side reaction) | **2a’** | No reaction  (without electron-withdrawing group) |
| **2b’** | No reaction  (without electron-withdrawing group) | **2c’** | No desired product  (carbonyl addition) |

2.6 Substrate scopes data of NHC-Catalyzed S*_N_*Ar reaction

(Perfluoropyridin-4-yl)(phenyl)methanone, 3a

According to **General Procedure A**, benzaldehyde **1a** (10.6 mg, 0.1 mmol, 1.0 equiv) and pentafluoropyridine **2a** (16.9 mg, 0.1 mmol, 1.0 equiv) were used to obtain crude residue, which was purified by silica gel column chromatography (Hexane) to afford the **3a** as pale-yellow liquid (23.5 mg, 0.092 mmol, 92%­).

**^1^H NMR** (300 MHz, CDCl_3_) *δ* 7.87 (d, *J* = 7.7 Hz, 2H), 7.74 (t, *J* = 7.4 Hz, 1H), 7.57 (t, *J* = 7.7 Hz, 2H) ppm.

**^13^C{^1^H} NMR** (101 MHz, CDCl_3_) *δ* 184.4, 144.9 – 142.1 (m), 140.0 – 137.0 (m), 135.7, 134.6, 131.5 (t, *J* = 18.7 Hz) 129.7, 129.4 ppm.

**^19^F NMR** (376 MHz, CDCl_3_) *δ* -87.9 – -88.0 (m), -141.4 – -141.5 (m) ppm.

*[See NMR spectra](#S3a)*

**HRMS** (*m/z*): (EI) calc’d for C_12_H_5_F_4_NO [M]^+^: 255.0299, found 255.0302

**IR** (neat) ν_max_: 2920, 2850, 1460, 1287, 965, 832, 672 cm^-1^

**TLC**: R*_f_* = 0.46 (Hexane/ethyl acetate 9:1)


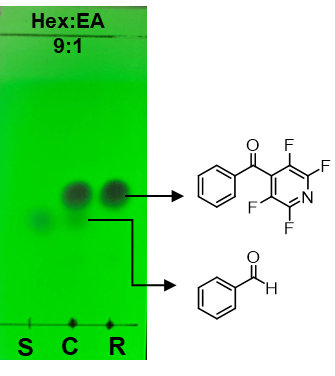


(Perfluoropyridin-4-yl)(4-(trifluoromethyl)phenyl)methanone, 3b

According to **General Procedure A**, 4-(trifluoromethyl)benzaldehyde **1b** (17.4 mg, 0.1 mmol, 1.0 equiv) and pentafluoropyridine **2a** (16.9 mg, 0.1 mmol, 1.0 equiv) were used to obtain crude residue, which was purified by silica gel column chromatography (Hexane/ethyl acetate 10:1) to afford the **3b** as colorless oil (22.9 mg, 0.071mmol, 71%).

**^1^H NMR** (300 MHz, CDCl_3_) *δ* 7.99 (d, *J* = 8.1, 2H), 7.84 (d, *J* = 8.1, 2H) ppm.

**^13^C{^1^H} NMR** (101 MHz, CDCl_3_) *δ* 183.6, 145.0 – 142.2 (m), 140.0 – 137.1 (m), 137.2, 136.9, 136.6, 130.7 – 130.3 (m), 130.02, 126.5 (q, *J* = 3.72 Hz), 123.1 (q, *J* = 273.04 Hz) ppm.

**^19^F NMR** (282 MHz, CDCl_3_) *δ* -63.5 (s, 1F), -87.3 (ddd, *J* = 26.7, 26.6, 12.1 Hz), -141.2 – -141.4 (m) ppm.

*[See NMR spectra](#S3b)*

**HRMS** (*m/z*): (EI) calc’d for C_13_H_4_NOF_7_ [M] ^+^: 323.0176, found 323.0174

**IR** (neat) ν_max_: 2919, 2850, 1689, 1484, 1321, 1133, 992 cm^-1^

**TLC**: R*_f_* = 0.58 (Hexane/ethyl acetate 9:1)


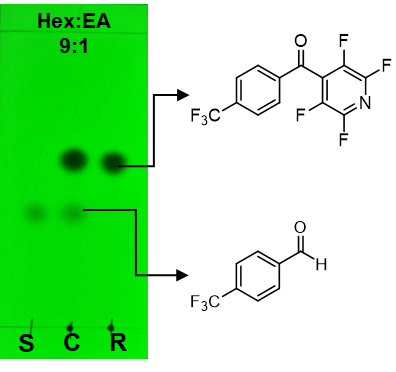


4-(Perfluoroisonicotinoyl)benzonitrile, 3c

According to **General Procedure A**, 4-formylbenzonitrile **1c** (13.1 mg, 0.1 mmol, 1.0 equiv) and pentafluoropyridine **2a** (16.9 mg, 0.1 mmol, 1.0 equiv) were used to obtain crude residue, which was purified by silica gel column chromatography (Hexane/ethyl acetate 10:1) to afford the **3c** as white solid (27.8 mg, 0.099 mmol, 99%).

**^1^H NMR** (300 MHz, CDCl_3_) *δ* 7.98 (d, *J* = 8.5 Hz, 2H), 7.87 (d, *J* = 8.7 Hz, 2H) ppm.

**^13^C{^1^H}** **NMR** (101 MHz, CDCl_3_) *δ* 183.3, 145.0 – 142.2 (m), 140.0 – 137.0 (m), 137.3, 133.1, 129.9, 118.8, 117.2 ppm.

**^19^F NMR** (282 MHz, CDCl_3_) *δ* -86.9 (ddd, *J* = 27.0, 26.5, 9.4 Hz), -141.9 – -141.1 (m) ppm.

*[See NMR spectra](#S3c)*

**HRMS** (*m/z*): (EI) calc’d for C_13_H_4_N_2_OF_4_ [M] ^+^: 280.0254, found 280.0257

**IR** (neat) ν_max_: 2926, 2854, 2232, 1689, 1459, 1284 cm^-1^

**M.P.**: 130 ℃

**TLC**: R*_f_* = 0.31 (Hexane/ethyl acetate 9:1)


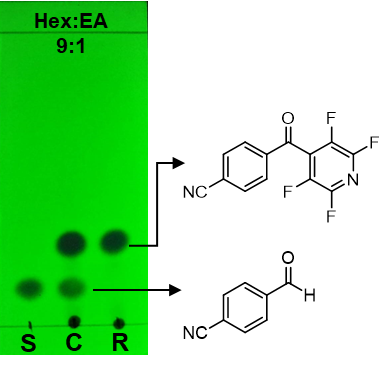


Methyl 4-(perfluoroisonicotinoyl)benzoate, 3d

According to **General Procedure A**, methyl 4-formylbenzoate **1d** (16.4 mg, 0.1 mmol, 1.0 equiv) and pentafluoropyridine **2a** (16.9 mg, 0.1 mmol, 1.0 equiv) were used to obtain crude residue, which was purified by silica gel column chromatography (Hexane/ethyl acetate 15:1) to afford the **3d** as white solid (20.9 mg, 0.067 mmol, 67%).

**^1^H NMR** (300 MHz, CDCl_3_) *δ* 8.20 (d, *J* = 8.1 Hz, 2H), 7.92 (d, *J* = 8.2 Hz, 2H), 3,97 (s, 3H) ppm.

**^13^C{^1^H}** **NMR** (101 MHz, CDCl_3_) *δ* 184.0, 165.6, 144.9 – 142.2 (m), 140.0 – 137.0 (m), 137.5, 136.1, 130.8 (t, *J* = 19.0 Hz), 130.4, 129.6, 52.7 ppm.

**^19^F NMR** (282 MHz, CDCl_3_) *δ* -87.5 – -87.7 (m), -141.3 – -141.5 ppm.

*[See NMR spectra](#S3d)*

**HRMS** (*m/z*): (EI) calc’d for C_14_H_7_NO_3_F_4_ [M] ^+^: 313.0357, found 313.0353

**IR** (neat) ν_max_: 2961, 2923, 2851, 1723, 1683, 1462, 1281, 964 cm^-1^

**M.P.**: 128 ℃

**TLC**: R*_f_* = 0.45 (Hexane/ethyl acetate 9:1)

**
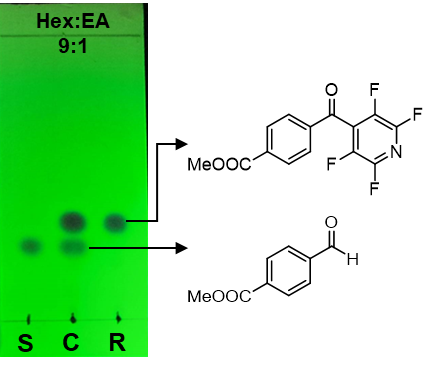
**

(4-Nitrophenyl)(perfluoropyridin-4-yl)methanone, 3e

According to **General Procedure A**, 4-nitrobenzaldehyde **1e** (15.1 mg, 0.1 mmol, 1.0 equiv) and pentafluoropyridine **2a** (16.9 mg, 0.1 mmol, 1.0 equiv) were used to obtain crude residue, which was purified by silica gel column chromatography (Hexane/ethyl acetate 10:1) to afford the **3e** as pale-yellow solid (22.9 mg, 0.071 mmol, 87%).

**^1^H NMR** (300 MHz, CDCl_3_) *δ* 8.42 – 8.39 (m, 2H), 8.08 – 8.05 (m, 2H) ppm.

**^13^C{^1^H} NMR** (101 MHz, CDCl_3_) *δ* 183.1, 151.6, 145.1 – 142.3 (m), 140.0 – 137.1 (m), 138.7, 130.7, 129.9 (t, *J* = 18.7 Hz), 124.5 ppm.

**^19^F NMR** (282 MHz, CDCl_3_) *δ* -86.8 – -87.0 (m), -140.8 – -141.1 (m) ppm.

*[See NMR spectra](#S3e)*

**HRMS** (*m/z*): (EI) calc’d for C_12_H_4_F_4_N_2_O_3_ [M] ^+^: 300.0153, found 300.0151

**IR** (neat) ν_max_: 1691, 1525, 1460, 1348, 1284, 962, 823 cm^-1^

**M.P.**: 98 ℃

**TLC**: R*_f_* = 0.26 (Hexane/ethyl acetate 9:1)


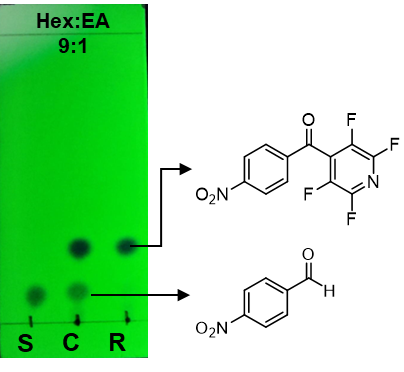


(4-Fluorophenyl)(perfluoropyridin-4-yl)methanone, 3f

According to **General Procedure A**, 4-fluorobenzaldehyde **1f** (12.4 mg, 0.1 mmol, 1.0 equiv) and pentafluoropyridine **2a** (16.9 mg, 0.1 mmol, 1.0 equiv) were used to obtain crude residue, which was purified by silica gel column chromatography (Hexane/ethyl acetate 9:1) to afford the **3f** as colorless oil (27.1 mg, 0.099 mmol, 99%).

**^1^H NMR** (300 MHz, CDCl_3_) *δ* 7.93 – 7.88 (m, 2H), 7.27 – 7.22 (m, 2H) ppm.

**^13^C{^1^H} NMR** (101 MHz, CDCl_3_) *δ* 182.7, 167.3 (d, *J* = 260.1 Hz), 144.8 – 142.3 (m), 139.6 – 137.3 (m), 132.6 (d, *J* = 10.1 Hz), 131.3 (d, *J* = 3.0 Hz), 116.9 (d, *J* = 22.3 Hz) ppm.

**^19^F NMR** (376 MHz, CDCl_3_) *δ* -88.2 – -88.4 (m), -100.0, -142.0 – -142.2 (m) ppm.

*[See NMR spectra](#S3f)*

**HRMS** (*m/z*): (EI) calc’d for C_12_H_4_NOF_5_ [M] ^+^: 273.0208, found 273.0210

**IR** (neat) ν_max_: 2922, 2851, 1680, 1595, 1455, 1287, 850 cm^-1^

**TLC**: R*_f_* = 0.50 (Hexane/ethyl acetate 9:1)


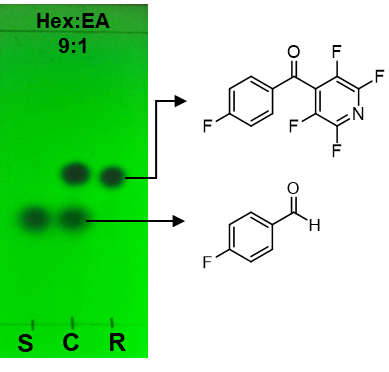


(4-Chlorophenyl)(perfluoropyridin-4-yl)methanone, 3g

According to **General Procedure A**, 4-chlorobenzaldehyde **1g** (14.0 mg, 0.1 mmol, 1.0 equiv) and pentafluoropyridine **2a** (16.9 mg, 0.1 mmol, 1.0 equiv) were used to obtain crude residue, which was purified by silica gel column chromatography (Hexane/ethyl acetate 10:1) to afford the **3g** as pale-yellow oil (26.1 mg, 0.090 mmol, 90%­).

**^1^H NMR** (300 MHz, CDCl_3_) *δ* 7.80 (d, *J* = 8.5 Hz, 2H), 7.55 – 7.52 (d, *J* = 8.6 Hz, 2H) ppm.

**^13^C{^1^H} NMR** (101 MHz, CDCl_3_) *δ* 183.2, 144.8 – 142.3 (m), 140.0 – 143.7 (m), 133.0 (m), 130.1, 129.8 ppm.

**^19^F NMR** (282 MHz, CDCl_3_) *δ* -87.7 (ddd, *J* = 26.7, 26.7, 9.7 Hz), -141.4 – -141.6 (m) ppm.

*[See NMR spectra](#S3g)*

**HRMS** (*m/z*): (EI) calc’d for C_12_H_4_ClF_4_NO [M]^+^: 288.9912, found 288.9910

**IR** (neat) ν_max_: 2922, 2851, 1680, 1448, 1376, 1266, 831 cm^-1^

**TLC**: R*_f_* = 0.53 (Hexane/ethyl acetate 9:1)


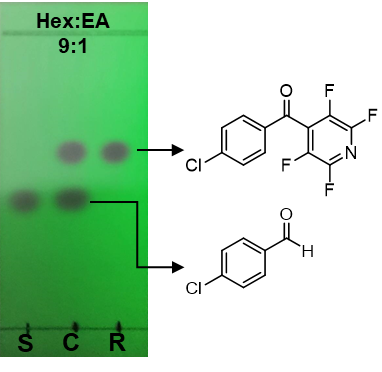


(4-Bromophenyl)(perfluoropyridin-4-yl)methanone, 3h

According to [**General Procedure**](#_2.2._General_Procedure) **A**, 4-bromobenzaldehyde **3h** (18.5 mg, 0.1 mmol, 1.0 equiv) and pentafluoropyridine **2a** (16.9 mg, 0.1 mmol, 1.0 equiv) were used to obtain crude residue, which was purified by silica gel column chromatography (Hexane/ethyl acetate 9:1) to afford the **3h** as white solid (33.1 mg, 0.099 mmol, 99%­).

**^1^H NMR** (300 MHz, CDCl_3_) *δ* 7.71 (s, 4H) ppm.

**^13^C{^1^H} NMR** (101 MHz, CDCl_3_) *δ* 183.4, 144.9 – 142.1 (m), 139.9 – 137.0 (m), 133.4, 132.8, 131.5, 130.9, 130.7 ppm.

**^19^F NMR** (282 MHz, CDCl_3_) *δ* -87.7 (ddd, *J* = 26.8, 26.6, 9.6 Hz), -141.4 – -141.6 (m) ppm.

*[See NMR spectra](#S3h)*

**HRMS** (*m/z*): (EI) calc’d for C_12_H_4_NOF_4_Br [M]^+^: 332.9407, found 332.9410

**IR** (neat) ν_max_: 1682, 1587, 1458, 1410, 1284, 968 cm^-1^

**M.P.**: 82 ℃

**TLC**: R*_f_* = 0.62 (Hexane/ethyl acetate 9:1)


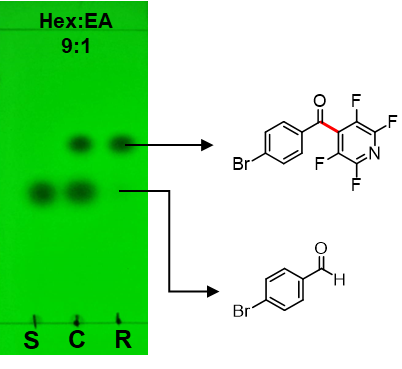


(Perfluoropyridin-4-yl)(p-tolyl)methanone, 3i

According to **General Procedure A**, 4-methylbenzaldehyde **1i** (12.0 mg, 0.1 mmol, 1.0 equiv) and pentafluoropyridine **2a** (16.9 mg, 0.1 mmol, 1.0 equiv) were used to obtain crude residue, which was purified by preparative TLC plate (Hexane/ethyl acetate 15:1) to afford the **3i** as colorless oil (14.8 mg, 0.055 mmol, 55%).

**^1^H NMR** (300 MHz, CDCl_3_) *δ* 7.75 (d, 2H, *J* = 8.02 Hz), 7.36 (d, 2H, *J* = 7.97 Hz), 2.47 (s, 3H) ppm.

**^13^C NMR** (400 MHz, CDCl_3_) *δ* 183.8, 147.3, 145.3 – 141.9 (m), 139.6 – 137.4 (m), 132.3, 130.1, 129.9, 22.0 ppm.

**^19^F NMR** (282 MHz, CDCl_3_) *δ* -88.3 (ddd, *J* = 26.9, 26.8, 9.4 Hz), -141.7 – -141.9 (m) ppm.

*[See NMR spectra](#S3i)*

**HRMS** (*m/z*): (EI) calc’d for C_13_H_7_NOF_4_ [M] ^+^: 269.0458, found 269.0456

**IR** (neat) ν_max_: 2923, 2851, 1676, 1604, 1458, 1287, 969, 842

**TLC**: R*_f_* = 0.55 (Hexane/ethyl acetate 9:1)


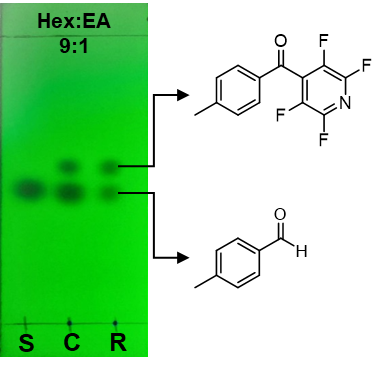


**(Perfluoropyridin-4-yl)(4-(4,4,5,5-tetramethyl-1,3,2-dioxaborolan-2-yl)phenyl)methanone,** **3j**

According to **General Procedure A**, 4-(4,4,5,5-tetramethyl-1,3,2-dioxaborolan-2-yl)benzaldehyde **1j** (23.2 mg, 0.1 mmol, 1.0 equiv) and pentafluoropyridine **2a** (16.9 mg, 0.1 mmol, 1.0 equiv) were used to obtain a crude residue, which was purified by silica gel column chromatography (Hexane/ethyl acetate 15:1) to afford the **3j** as white solid (24.1 mg, 0.064 mmol, 64%­).

**^1^H NMR** (300 MHz, CDCl_3_) *δ* 7.98 (d, *J* = 8.4 Hz, 2H), 7.82 (d, *J* = 7.9 Hz, 2H), 1.36 (s, 12H) ppm.

**^13^C{^1^H} NMR** (101 MHz, CDCl_3_) *δ* 184.7, 144.8 – 142.3 (m), 140.0 – 137.4 (m), 136.3, 135.5, 128.6, 84.5, 24.8 ppm.

**^19^F NMR** (376 MHz, CDCl_3_) *δ* -88.0 (ddd, *J* = 26.6, 26.5, 9.8 Hz), -141.4 – -141.9 (m) ppm.

*[See NMR spectra](#S3j)*

**HRMS** (*m/z*): (EI) calc’d for C_18_H_16_BNO_3_F_4_ [M]^+^: 381.1154, found 381.1157

**IR** (neat) ν_max_: 2981, 2930, 1683, 1459, 1359, 1289, 1142, 1090, 961, 809 cm^-1^

**M.P.**: 84 ℃

**TLC:** R*_f_* = 0.73 (Hexane/ethyl acetate 7:3)


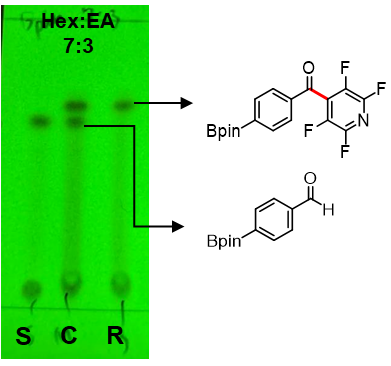


Methyl 3-(perfluoroisonicotinoyl)benzoate, 3k

According to **General Procedure A**, methyl 3-formylbenzoate **1k** (16.4 mg, 0.1 mmol, 1.0 equiv) and pentafluoropyridine **2a** (16.9 mg, 0.1 mmol, 1.0 equiv) were used to obtain crude residue, which was purified by silica gel column chromatography (Hexane/ethyl acetate 15:1) to afford the **3k** as colorless oil (25.1 mg, 0.080 mmol, 80%).

**^1^H NMR** (300 MHz, CDCl_3_) *δ* 8.42 (s, 1H), 8.40 – 8.37 (m, 1H), 8.11 – 8.08 (m, 1H), 7.68 (t, *J* = 7.8 Hz, 1H), 3.96 (s, 3H) ppm.

**^13^C{^1^H} NMR** (101 MHz, CDCl_3_) *δ* 183.8, 165.5, 145.0 – 142.2 (m), 140.0 – 137.4 (m), 137.1, 136.3, 135.0, 133.3, 131.6, 130.7, 129.7, 52.7 ppm.

**^19^F NMR** (282 MHz, CDCl3) *δ* -87.4 – -87.7 (m), -141.3 – -141.5 (m) ppm.

*[See NMR spectra](#S3k)*

**HRMS** (*m/z*): (ESI) calc’d for C_14_H_8_O_3_NF_4_ [M+H]^+^: 314.0435, found: 314.0434

**IR** (neat) ν_max_: 2957, 1726, 1461, 1268, 1209, 969, 744 cm^-1^

**TLC**: R*_f_* = 0.35 (Hexane/ethyl acetate 9:1)


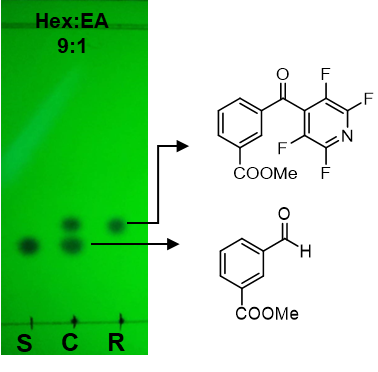


(3-Nitrophenyl)(perfluoropyridin-4-yl)methanone, 3l

According to **General Procedure A**, 3-nitrobenzaldehyde **1l** (15.1 mg, 0.1 mmol, 1.0 equiv) and pentafluoropyridine **2a** (16.9 mg, 0.1 mmol, 1.0 equiv) were used to obtain crude residue, which was purified by silica gel column chromatography (Hexane/ethyl acetate 25:1→15:1) to afford the **3l** as pale-yellow oil (14.9 mg, 0.050 mmol, 50%).

**^1^H NMR** (300 MHz, CDCl_3_) *δ* 8.68 – 8.66 (m, 1H), 8.61 – 8.57 (m, 1H), 8.22 – 8.19 (m, 1H), 7.82 (t, *J* = 8.0 Hz, 1H) ppm.

**^13^C{^1^H} NMR** (101 MHz, CDCl_3_) *δ* 182.6, 148.8, 145.0 – 142.3 (m), 140.1 – 137.2 (m), 135.9, 134.8, 130.8, 130.0 – 129.8 (m), 129.7, 124.3 ppm.

**^19^F NMR** (282 MHz, CDCl_3_) *δ* -86.6 – -86.8 (m), -140.8 – -141.1 (m) ppm.

[*See NMR spectra*](#S3l)

**HRMS** (*m/z*): (EI) calc’d for C_12_H_4_N_2_O_3_F_4_ [M]^+^: 300.0153, found 300.0149

**IR** (neat) ν_max_: 3088, 2923, 2851, 1533, 1461, 1351, 969 cm^-1^

**TLC**: R*_f_* = 0.20 (Hexane/ethyl acetate 9:1)


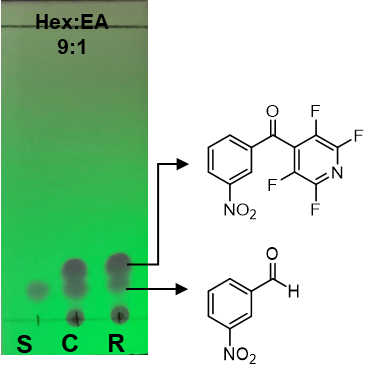


(3-Bromophenyl)(perfluoropyridin-4-yl)methanone, 3m

According to **General Procedure A**, 3-bromobenzaldehyde **1m** (18.5 mg, 0.1 mmol, 1.0 equiv) and pentafluoropyridine **2a** (16.9 mg, 0.1 mmol, 1.0 equiv) were used to obtain crude residue, which was purified by silica gel column chromatography (Hexane/ethyl acetate 20:1) to afford the **3m** as colorless oil (19.1 mg, 0.057 mmol, 57%).

**^1^H NMR** (400 MHz, CDCl_3_) *δ* 8.010 – 8.006 (m, 1H), 7.86 – 7.74 (m, 2H), 7.47 – 7.43 (m, 1H) ppm.

**^13^C{^1^H} NMR** (101 MHz, CDCl_3_) *δ* 183.2, 144.9 – 142.1 (m), 140.0 – 137.0 (m), 138.6, 136.3, 132.3, 130.9, 130.7 – 130.3 (m), 128.3, 123.7 ppm.

**^19^F NMR** (282 MHz, CDCl_3_) *δ* -87.5 (ddd, 28.59, 28.39, 12.55 Hz), -141.2 – -142.4 (m) ppm.

*[See NMR spectra](#S3m)*

**HRMS** (*m/z*): (EI) calc’d for C_12_H_4_NOF_4_Br [M^+^]: 332.9407, found 332.9404

**IR** (neat) ν_max_: 2917, 2851, 1683, 1462, 1288, 968, 679 cm^-1^

**TLC**: R*_f_* = 0.42 (Hexane/ethyl acetate 9:1)


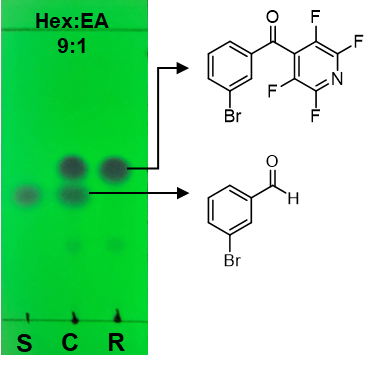


Methyl 2-(perfluoroisonicotinoyl)benzoate, 3n

According to **General Procedure A**, methyl 2-formylbenzoate **1n** (16.4 mg, 0.1 mmol, 1.0 equiv) and pentafluoropyridine **2a** (16.9 mg, 0.1 mmol, 1.0 equiv) were used to obtain crude residue, which was purified by silica gel column chromatography (Hexane/ethyl acetate 30:1→20:1) to afford the **3n** as a colorless oil (22.2 mg, 0.071 mmol, 71%).

**^1^H NMR** (300 MHz, CDCl_3_) *δ* 7.99 – 7.96 (m, 1H), 7.74 – 7.65 (m, 2H), 7.58 – 7.55 (m, 1H), 3.81 (s, 3H) ppm.

**^13^C{^1^H} NMR** (101 MHz, CDCl_3_) *δ* 186.5, 166.5, 145.1 – 142.3 (m), 140.8 – 137.8 (m), 140.1, 132.8, 132.2, 130.7 – 130.4 (m), 130.2, 129.3, 128.8, 52.9 ppm.

**^19^F NMR** (282 MHz, CDCl3) *δ* -88.8 – -89.0 (m), -141.0 – -141.3 (m) ppm.

*[See NMR spectra](#S3n)*

**HRMS** (*m/z*): (ESI) calc’d for C_14_H_7_O_3_NF_4_^23^Na [M+Na]^+^: 336.025 , found 336.02542

**IR** (neat) ν_max_: 2919, 2852, 1696, 1463, 1284, 961, 708 cm^-1^

**TLC**: R*_f_* = 0.22 (Hexane/ethyl acetate 9:1)


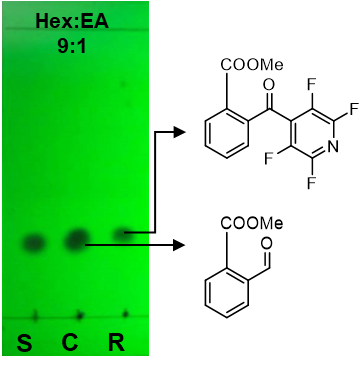


(2-Nitrophenyl)(perfluoropyridin-4-yl)methanone, 3o

According to **General Procedure A**, 2-nitrobenzaldehyde **1o** (15.1 mg, 0.1 mmol, 1.0 equiv) and pentafluoropyridine **2a** (16.9 mg, 0.1 mmol, 1.0 equiv) were used to obtain crude residue, which was purified by preparative TLC plate (Hexane/ethyl acetate 25:1) to afford the **3o** as pale-yellow solid (9.1 mg, 0.030 mmol, 30%).

**^1^H NMR** (300 MHz, CDCl_3_) *δ* 8.23 (d, *J* = 8.1 Hz, 1H), 7.93 – 7.76 (m, 2H), 7.67 (d, J = 7.4, 1.6 Hz, 1H) ppm.

**^13^C{^1^H} NMR** (101 MHz, CDCl_3_) *δ* 183.3, 146.1, 145.4 – 142.4 (m), 140.9 – 137.9 (m), 135.0, 132.8, 129.6, 124.7 ppm.

**^19^F NMR** (282 MHz, CDCl_3_) *δ* -87.7 – -88.0 (m), -141.1 – -141.3 (m) ppm.

*[See NMR spectra](#S3o)*

**HRMS** (*m/z*): (EI) calc’d for C_12_H_4_F_4_N_2_O_3_ [M^+^]: 300.0153, found 300.0150

**IR** (neat) ν_max_: 2922, 2854, 1699, 1530, 1461, 1345, 1294, 969, 828 cm^-1^

**M.P.**: 99 ℃

**TLC**: R*_f_* = 0.20 (Hexane/ethyl acetate 9:1)


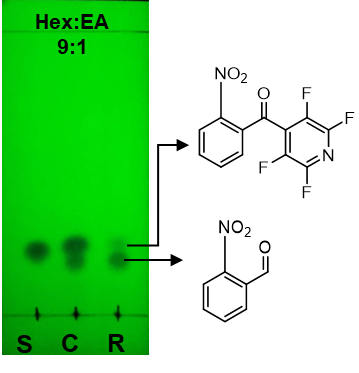


(2-Bromophenyl)(perfluoropyridin-4-yl)methanone, 3p

According to **General Procedure A**, 2-bromobenzaldehyde **1p** (18.5 mg, 0.1 mmol, 1.0 equiv) and pentafluoropyridine **2a** (16.9 mg, 0.1 mmol, 1.0 equiv) were used to obtain crude residue, which was purified by silica gel column chromatography (Hexane/ethyl acetate 20:1) to afford the **3p** as colorless oil (24.7 mg, 0.074 mmol, 74%).

**^1^H NMR** (400 MHz, CDCl_3_) *δ* 7.72 – 7.64 (m, 2H), 7.52 – 7.46 (m, 2H) ppm.

**^13^C{^1^H} NMR** (101 MHz, CDCl_3_) *δ* 184.8, 144.9 – 142.1 (m), 140.4 – 137.4 (m), 137.1, 134.7, 131.8, 131.7 – 131.3 (m), 128.1, 121.1 ppm.

**^19^F NMR** (282 MHz, CDCl_3_) *δ* -88.1 – -88.4 (m), -141.6 – -141.8 (m) ppm.

*[See NMR spectra](#S3p)*

**HRMS** (*m/z*): (EI) calc’d for C_12_H_4_NOF_4_Br [M^+^]: 332.9407, found 332.9405

**IR** (neat) ν_max_: 1684, 1586, 1459, 1406, 1294, 968, 832, 744 cm^-1^

**TLC**: R*_f_* = 0.65 (Hexane/ethyl acetate 9:1)


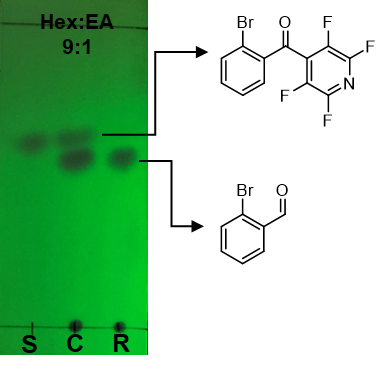


(2-Chloro-5-nitrophenyl)(perfluoropyridin-4-yl)methanone, 3q

According to **General Procedure A**, 2-chloro-5-nitrobenzaldehyde **1q** (18.5 mg, 0.1 mmol, 1.0 equiv) and pentafluoropyridine **2a** (16.9 mg, 0.1 mmol, 1.0 equiv) were used to obtain crude residue, which was purified by preparative TLC plate (Hexane/ethyl acetate 25:1→15:1) to afford the **3q** as pale-yellow solid (27.1 mg, 0.081 mmol, 81%).

**^1^H NMR** (300 MHz, CDCl_3_) *δ* 8.57 (d, *J* = 2.7 Hz, 1H), 8.42 (dd, *J* = 8.8, 2.7 Hz, 1H), 7.72 (d, *J* = 8.8 Hz, 1H) ppm.

**^13^C{^1^H} NMR** (101 MHz, CDCl_3_) *δ* 182.3, 146.9, 145.1 – 142.3 (m), 140.4 – 137.4 (m), 139.2, 136.3, 132.5, 130.5 – 130.1 (m), 128.6, 126.2 ppm.

**^19^F NMR** (282 MHz, CDCl_3_) *δ* -86.9 – -87.1 (m), -141.5 – -141.7 (m) ppm.

*[See NMR spectra](#S3q)*

**HRMS** (*m/z*): (EI) calc’d for C_12_H_3_N_2_O_3_F_4_Cl [M^+^]: 333.9763, found 333.9760

**IR** (neat) ν_max_: 2923, 2854, 1608, 1530, 1461, 1348, 739 cm^-1^

**M.P.**: 102 ℃

**TLC**: R*_f_* = 0.36 (Hexane/ethyl acetate 9:1)


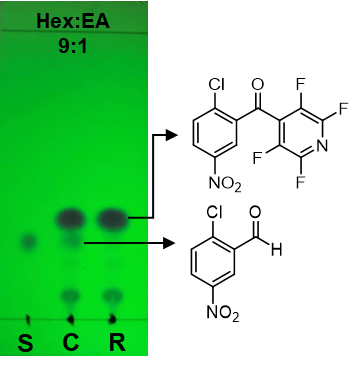


(5-Chloro-2-nitrophenyl)(perfluoropyridin-4-yl)methanone, 3r

According to **General Procedure A**, 5-chloro-2-nitrobenzaldehyde **1r** (18.5 mg, 0.1 mmol, 1.0 equiv) and pentafluoropyridine **2a** (16.9 mg, 0.1 mmol, 1.0 equiv) were used to obtain crude residue, which was purified by silica gel column chromatography (Hexane/ethyl acetate 25:1→15:1) to afford the **3r** as pale-yellow solid (24.0 mg, 0.072 mmol, 72%).

**^1^H NMR** (300 MHz, CDCl_3_) *δ* 8.20 (d, *J* = 8.8 Hz, 1H), 7.74 (dd, *J* = 8.8, 2.2 Hz, 1H), 7.62 (d, *J* = 2.2 Hz, 1H) ppm.

**^13^C{^1^H} NMR** (101 MHz, CDCl_3_) *δ* 181.8, 145.2 – 142.5 (m), 144.1, 142.2, 140.7 – 138.0 (m), 136.5, 132.5, 129.5, 126.1 ppm.

**^19^F NMR** (282 MHz, CDCl_3_) *δ* -87.3 – -87.5 (m), -140.8 – -141.1 (m) ppm.

*[See NMR spectra](#S3r)*

**HRMS** (*m/z*): (EI) calc’d for C_12_H_3_N_2_O_3_F_4_Cl [M^+^]: 333.9763, found 333.9766

**IR** (neat) ν_max_: 2919, 2852, 1696, 1463, 1284, 961, 708 cm^-1^

**TLC**: R*_f_* = 0.38 (Hexane/ethyl acetate 9:1)


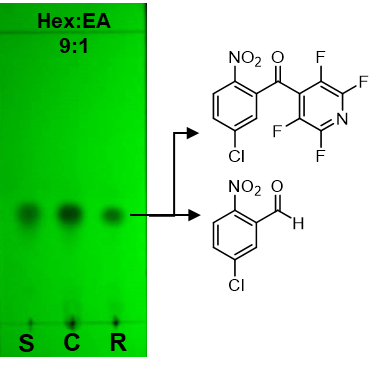


(3-Bromo-4-fluorophenyl)(perfluoropyridin-4-yl)methanone, 3s

According to **General Procedure A**, 3-bromo-4-fluorobenzaldehyde **1s** (20.3 mg, 0.1 mmol, 1.0 equiv) and pentafluoropyridine **2a** (16.9 mg, 0.1 mmol, 1.0 equiv) were used to obtain crude residue, which was purified by silica gel column chromatography (Hexane/ethyl acetate 20:1) to afford the **3s** as pale-yellow liquid (23.2 mg, 0.066 mmol, 66%).

**^1^H NMR** (400 MHz, CDCl_3_) *δ* 8.12 (dd, *J* = 6.4, 2.2 Hz, 1H), 7.82 – 7.77 (m, 1H), 7.30 (dd, *J* = 8.6, 7.8 Hz, 1H) ppm.

**^13^C{^1^H} NMR** (101 MHz, CDCl_3_) *δ* 181.8, 163.6 (d, *J* = 260.0 Hz), 143.5 (dt, *J* = 248.9, 14.0 Hz), 149.9 – 137.0 (m), 135.4, 132.2 (d, *J* = 3.5 Hz), 131.1 (d, *J* = 9.3 Hz), 130.4 (t, *J* = 19.3 Hz), 117.6 (d, *J* = 23.4 Hz), 111.0 (d, *J* = 22.2 Hz) ppm.

**^19^F NMR** (282 MHz, CDCl_3_) *δ* -87.1 – -87.3 (m), -93.8, -141.2 – -141.4 (m) ppm.

*[See NMR spectra](#S3s)*

**HRMS** (*m/z*): (EI) calc’d for C_12_H_3_NOF_5_Br [M]^+^: 350.9313, found 350.9313

**IR** (neat) ν_max_: 1686, 1459, 1289, 1264, 1048, 969, 777 cm^-1^

**TLC**: R*_f_* = 0.62 (Hexane/ethyl acetate 9:1)


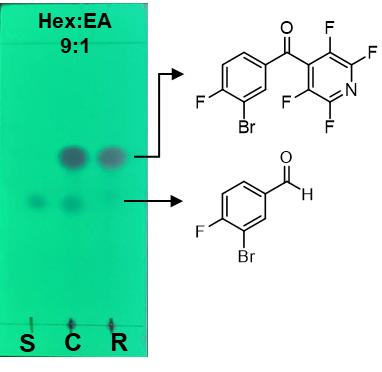


[1,1'-Biphenyl]-4-yl(perfluoropyridin-4-yl)methanone, 3t

According to **General Procedure A**, [1,1'-biphenyl]-4-carbaldehyde **1t** (18.2 mg, 0.1 mmol, 1.0 equiv) and pentafluoropyridine **2a** (16.9 mg, 0.1 mmol, 1.0 equiv) were used to obtain crude residue, which was purified by silica gel column chromatography (Hexane/ethyl acetate 9:1) to afford the **3t** as pale-yellow solid (25.8 mg, 0.078 mmol, 78%­).

**^1^H NMR** (300 MHz, CDCl_3_) *δ* 7.94 (d, *J* = 8.5 Hz, 2H), 7.78 (d, *J* = 8.6 Hz, 2H), 7.66 – 7.63 (m, 2H), 7.53 – 7.44 (m, 3H) ppm.

**^13^C{^1^H} NMR** (101 MHz, CDCl_3_) *δ* 183.8, 148.5, 144.9 – 142.1 (m), 140.0 – 137.1 (m), 139.1, 133.3, 131.8 – 131.4 (m), 130.4, 129.1, 128.9, 127.9, 127.4 ppm.

**^19^F NMR** (282 MHz, CDCl_3_) *δ* -88.0 (ddd, *J* = 28.8, 28.5, 12.6 Hz), -141.4 – -141.7 (m) ppm.

*[See NMR spectra](#S3t)*

**HRMS** (*m/z*): (EI) calc’d for C_18_H_9_NOF_4_ [M]^+^: 331.0615, found 331.0617

**IR** (neat) ν_max_: 2923, 2851, 1683, 1459, 1273, 967, 695 cm^-1^

**M.P.**: 105 ℃

**TLC**: R*_f_* = 0.51(Hexane/ethyl acetate 9:1)


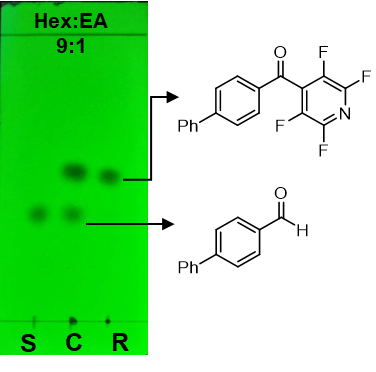


Naphthalen-2-yl(perfluoropyridin-4-yl)methanone, 3u

According to **General Procedure A**, 2-naphthaldehyde **1u** (15.6 mg, 0.1 mmol, 1.0 equiv) and pentafluoropyridine **2a** (16.9 mg, 0.1 mmol, 1.0 equiv) were used to obtain crude residue, which was purified by silica gel column chromatography (Hexane/ethyl acetate 25:1) to afford the **3u** as white solid (20.4 mg, 0.067 mmol, 67%­).

**^1^H NMR** (300 MHz, CDCl_3_) *δ* 8.23 (s, 1H), 8.02 – 7.92 (m, 4H), 7.73 – 7.58 (m, 2H) ppm.

**^13^C{^1^H} NMR** (101 MHz, CDCl_3_) *δ* 184.2, 144.9 – 142.2 (m), 140.1 – 137.2 (m), 136.7, 133.4, 132.3, 132.2, 131.8 – 131.6 (m), 130.1, 130.0, 129.6, 128.1, 127.6, 123.4 ppm.

**^19^F NMR** (282 MHz, CDCl_3_) *δ* -87.9 – -88.1 (m), -141.4 – -141.6 (m) ppm.

*[See NMR spectra](#S3u)*

**HRMS** (*m/z*): (EI) calc’d for C_16_H_7_NOF_4_ [M]^+^: 305.0458, found 305.0460

**IR** (neat) ν_max_: 2925, 2856, 1676, 1451, 1294, 962, 740 cm^-1^

**M.P.**: 92 ℃

**TLC**: R*_f_* = 0.54 (Hexane/ethyl acetate 9:1)


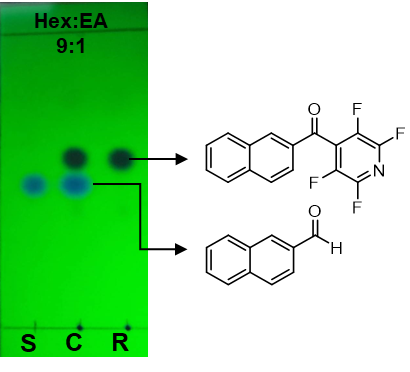


(1*H*-Indol-3-yl)(perfluoropyridin-4-yl)methanone, 3v

According to **General Procedure A**, 1*H*-indole-3-carbaldehyde **1v** (14.5 mg, 0.1 mmol, 1.0 equiv) and pentafluoropyridine **2a** (16.9 mg, 0.1 mmol, 1.0 equiv) were used to obtain crude residue, which was purified by silica gel column chromatography (Hexane/ethyl acetate 9:1) to afford the **3v** as pale brown solid (15.0 mg, 0.051 mmol, 51%­).

**^1^H NMR** (300 MHz, CDCl_3_) *δ* 10.18 (s, 1H), 8.42 – 8.39 (m, 1H), 7.89 – 7.87 (m, 1H), 7.47 – 7.44 (m, 2H), 7.24 – 7.21 (m, 1H) ppm.

**^13^C{^1^H} NMR** (101 MHz, CDCl_3_) *δ* 184.9, 145.5 – 143.1 (m), 138.9 – 135.8 (m), 137.0, 136.5, 128.7 – 128.5 (m), 125.9, 125.0, 124.6, 122.7, 122.5, 111.02 – 110.97 (m) ppm.

**^19^F NMR** (282 MHz, CDCl_3_) *δ* -86.0 – -86.2 (m), -145.2 – -145.5 (m) ppm.

*[See NMR spectra](#S3v)*

**HRMS** (*m/z*): (EI) calc’d for C_14_H_6_N_2_OF_4_ [M]^+^: 294.0411, found 294.0412

**IR** (neat) ν_max_: 3118, 3069, 1672, 1550, 1472, 1181, 1043 cm^-1^

**M.P.**: 169 ℃

**TLC**: R*_f_* = 0.51(Hexane/ethyl acetate 9:1)


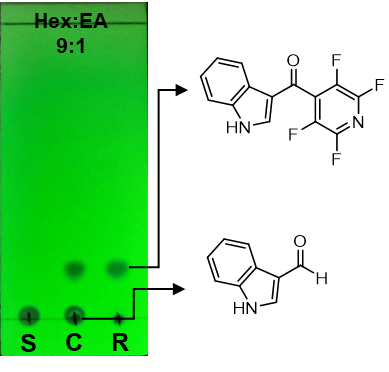


(Perfluoropyridin-4-yl)(2-phenylquinolin-4-yl)methanone, 3w

According to **General Procedure A**, 2-phenylquinoline-4-carbaldehyde **1v** (23.3 mg, 0.1 mmol, 1.0 equiv) and pentafluoropyridine **2a** (16.9 mg, 0.1 mmol, 1.0 equiv) were used to obtain crude residue, which was purified by silica gel column chromatography (Hexane/ethyl acetate 15:1) to afford the **3w** yellow solid (19.9 mg, 0.052 mmol, 52%­).

**^1^H NMR** (300 MHz, CDCl_3_) *δ* 8.76 (d, *J* = 8.5 Hz, 1H), 8.32 (d, *J* = 8.4 Hz, 1H), 8.07 – 8.05 (m, 2H), 7.91 – 7.85 (m, 2H), 7.75 (t, *J* = 7.8 Hz, 1H), 7.55 – 7.53 (m, 3H) ppm.

**^13^C{^1^H} NMR** (101 MHz, CDCl_3_) *δ* 186.0, 157.1, 149.6, 145.3 – 142.3 (m), 140.0 – 137.3 (m), 138.9, 138.2, 131.6 – 131.3 (m), 130.9, 130.7, 130.2, 129.5, 129.1, 127.5, 124.7, 122.1, 121.2 ppm.

**^19^F NMR** (282 MHz, CDCl_3_) *δ* -87.0 (ddd, *J* = 27.0, 26.9, 9.9 Hz), -141.2 (ddd, *J* = 26.9, 26.7, 10.0 Hz) ppm.

*[See NMR spectra](#S3w)*

**HRMS** (*m/z*): (ESI) calc’d for C_21_H_11_ON_2_F_4_ [M+H]^+^: 383.0802, found: 383.0801

**IR** (neat) ν_max_: 2925, 2854, 1689, 1460, 1264, 966, 770 cm^-1^

**M.P.**: 132 ℃

**TLC**: R*_f_* = 0.51 (Hexane/ethyl acetate 9:1)


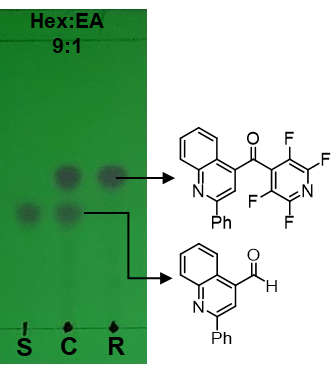


(Perfluoropyridin-4-yl)(thiophen-2-yl)methanone, 3x

According to **General Procedure A**, thiophene-2-carbaldehyde **1x** (11.2 mg, 0.1 mmol, 1.0 equiv) and pentafluoropyridine **2a** (16.9 mg, 0.1 mmol, 1.0 equiv) were used to obtain crude residue, which was purified by preparative TLC plate (Hexane/ethyl acetate 25:1) to afford the **3x** (11.8 mg, 0.045 mmol, 45%­).

**^1^H NMR** (300 MHz, CDCl_3_) *δ* 7.94 (dd, *J* = 4.9, 1.0 Hz, 1H), 7.56 (d, *J* = 3.9 Hz, 1H), 7.24 – 7.21 (m, 1H) ppm.

**^13^C{^1^H} NMR** (101 MHz, CDCl_3_) *δ* 175.5, 138.3, 136.6, 129.0 ppm.

**^19^F NMR** (282 MHz, CDCl_3_) *δ* -87.6 – -87.9 (m), -141.3 – -141.5 (m) ppm.

*[See NMR spectra](#S3x)*

**HRMS** (*m/z*): (EI) calc’d for C_10_H_3_F_4_NOS [M]^+^: 260.9868, found 260.9866

**IR** (neat) ν_max_: 2956, 2917, 2849, 1662, 1463, 1410, 1300, 797 cm^-1^

**M.P.**: 54 ℃

**TLC**: R*_f_* = 0.35 (Hexane/ethyl acetate 9:1)


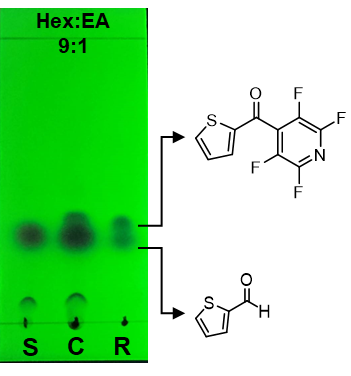


(3-Bromothiophen-2-yl)(perfluoropyridin-4-yl)methanone, 3y

According to **General Procedure A**, 3-bromothiophene-2-carbaldehyde **1y** (19.1 mg, 0.1 mmol, 1.0 equiv) and pentafluoropyridine **2a** (16.9 mg, 0.1 mmol, 1.0 equiv) were used to obtain crude residue, which was purified by silica gel column chromatography (Hexane/ethyl acetate 9:1) to afford the **3y** white solid (25.8 mg, 0.076 mmol, 76%­).

**^1^H NMR** (300 MHz, CDCl_3_) *δ* 7.81 (d, *J* = 1.4 Hz, 1H), 7.45 (d, *J* = 1.2 Hz, 1H) ppm.

**^13^C{^1^H} NMR** (101 MHz, CDCl_3_) *δ* 174.8, 145.0 – 142.2 (m), 141.8, 139.9 – 136.9 (m), 138.1, 135.2, 130.2 (t, *J* = 18.8 Hz), 112.2 ppm.

**^19^F NMR** (282 MHz, CDCl_3_) *δ* -87.1 (ddd, *J* = 27.7, 27.6, 12.0 Hz), -141.0 – -141.2 (m) ppm.

*[See NMR spectra](#S3y)*

**HRMS** (*m/z*): (EI) calc’d for C_10_H_2_NOF_4_SBr [M]^+^: 338.8971, found 338.8971

**IR** (neat) ν_max_: 3106, 1660, 1458, 1388, 1296, 1188, 966, 781, 694 cm^-1^

**M.P.**: 84 ℃

**TLC**: R*_f_* = 0.61 (Hexane/ethyl acetate 9:1)


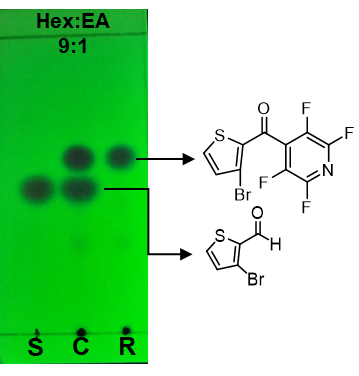


Phenyl(2,3,5,6-tetrafluoro-4-(trifluoromethyl)phenyl)methanone, 4a

According to **General Procedure A**, benzaldehyde **1a** (10.6 mg, 0.1 mmol, 1.0 equiv) and 1,2,3,4,5-pentafluoro-6-(trifluoromethyl)benzene **2b** (23.6 mg, 0.1 mmol, 1.0 equiv) were used to obtain crude residue, which was purified by silica gel column chromatography (Hexane) to afford the **4a** as colorless liquid (27.6 mg, 0.086 mmol, 86%­).

**^1^H NMR** (400 MHz, CDCl_3_) *δ* 7.86 (d, *J* = 7.8 Hz, 2H), 7.72 (t, *J* = 7.3 Hz, 1H), 7.55 (t, *J* = 7.4 Hz, 2H) ppm.

**^13^C{^1^H} NMR** (101 MHz, CDCl_3_) *δ* 184.7, 145.6 – 142.2 (m), 135.5, 135.2, 129.7, 129.3, 122.7 – 121.9 (m) ppm.

**^19^F NMR** (376 MHz, CDCl_3_) *δ* -56.3 – -56.4 (m), -138.0 – -138.1 (m), -138.27 – -138.30 (m) ppm.

*[See NMR spectra](#S4a)*

**HRMS** (*m/z*): (EI) calc’d for C_14_H_5_F_7_O [M]^+^: 322.0220, found 322.0223

**IR** (neat) ν_max_: 2925, 2853, 1996, 1680, 1482, 1333, 1150, 990, 715 cm^-1^

**TLC**: R*_f_* = 0.56 (Hexane/ethyl acetate 9:1)


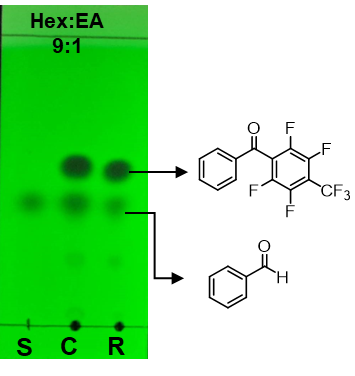


4-Benzoyl-2,3,5,6-tetrafluorobenzonitrile, 4b

According to **General Procedure A**, benzaldehyde **1a** (10.6 mg, 0.1 mmol, 1.0 equiv) and 2,3,4,5,6-pentafluorobenzonitrile **2c** (19.3 mg, 0.1 mmol, 1.0 equiv) were used to obtain crude residue, which was purified by silica gel column chromatography (Hexane) to afford the **4b** as colorless oil (15.1 mg, 0.054 mmol, 54%­).

**^1^H NMR** (300 MHz, CDCl_3_) *δ* 7.85 – 7.82 (m, 2H), 7.75 – 7.70 (m, 1H), 7.56 (t, *J* = 7.8 Hz, 2H) ppm.

**^13^C{^1^H} NMR** (101 MHz, CDCl_3_) *δ* 184.1, 148.4 – 145.8 (m), 144.5 – 141.9 (m), 135.7, 135.0, 129.7, 129.3, 124.9 – 124.5 (m), 106.9 ppm.

**^19^F NMR** (282 MHz, CDCl_3_) *δ* -129.9 – -130.1 (m), -137.2 – -137.3 (m) ppm.

*[See NMR spectra](#S4b)*

**HRMS** (*m/z*): (EI) calc’d for C_14_H_5_NOF_4_ [M]^+^: 279.0302, found 279.0299

**IR** (neat) ν_max_**:** 3055, 1684, 1490, 1323, 1265, 992, 737 cm^-1^

**TLC**: R*_f_* = 0.36 (Hexane/ethyl acetate 9:1)


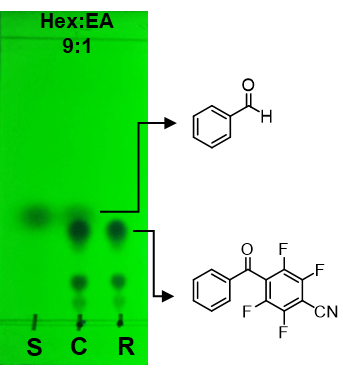


1-(4-Benzoyl-2,3,5,6-tetrafluorophenyl)ethan-1-one, 4c

According to **General Procedure A**, benzaldehyde **1a** (10.6 mg, 0.1 mmol, 1.0 equiv) and 1-(perfluorophenyl)ethan-1-one **2d** (21.0 mg, 0.1 mmol, 1.0 equiv) were used to obtain crude residue, which was purified by silica gel column chromatography (Hexane) to afford the **4c** as colorless oil (26.0 mg, 0.088 mmol, 88%).

**^1^H NMR** (400 MHz, TMS) *δ* 7.86 (d, *J* = 7.2 Hz, 2H), 7.72 – 7.68 (m, 1H), 7.54 (t, *J* = 7.8 Hz, 2H), 2.68 (s, 3H).

**^13^C{^1^H} NMR** (101 MHz, CDCl_3_) *δ* 191.6, 185.4, 144.7 (ddt, *J* = 37.5, 15.0, 5.0), 142.2 (ddt, *J* = 34.7, 15.2, 5.1), 135.5, 135.2, 129.7, 129.1, 121.3 – 120.8 (m), 32.4 ppm.

**^19^F NMR** (376 MHz, CDCl_3_) *δ* -139.0 – -139.1 (m), -140.0 – -140.1 (m) ppm.

*[See NMR spectra](#S4c)*

**HRMS** (*m/z*): (EI) calc’d for C_15_H_8_F_4_O_2_ [M]^+^: 296.0455, found 296.0455

**IR** (neat) ν_max_**:** 2925, 1715, 1678, 1471, 1303, 1172, 962, 667 cm^-1^

**TLC**: R*_f_* = 0.32 (Hexane/ethyl acetate 9:1)


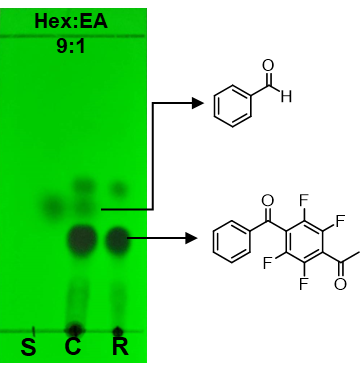


Methyl 4-benzoyl-2,3,5,6-tetrafluorobenzoate, 4d

According to **General Procedure A**, benzaldehyde **1a** (10.6 mg, 0.1 mmol, 1.0 equiv) and methyl 2,3,4,5,6-pentafluorobenzoate **2e** (22.6 mg, 0.1 mmol, 1.0 equiv) were used to obtain crude residue, which was purified by silica gel column chromatography (Hexane/ethyl acetate 9:1) to afford the **4d** as yellow oil (24.9 mg, 0.080 mmol, 80%­).

**^1^H NMR** (300 MHz, CDCl_3_) *δ* 7.87 – 7.84 (m, 2H), 7.72 – 7.66 (m, 1H), 7.56 – 7.51 (m, 2H), 4.02 (s, 3H) ppm.

**^13^C{^1^H} NMR** (101 MHz, CDCl_3_) *δ* 185.3, 159.6, 145.8 – 143.2 (m), 144.5 – 141.9 (m), 135.5, 135.2, 129.7, 129.1, 121.6 – 121.2 (m), 114.4 – 114.0 (m), 53.5 ppm.

**^19^F NMR** (282 MHz, CDCl_3_) *δ* -137.5 – -137.7 (m), -139.4 – -139.6 (m) ppm.

*[See NMR spectra](#S4d)*

**HRMS** (*m/z*): (EI) calc’d for C_15_H_8_O_3_F_4_ [M]^+^: 312.0404, found 312.0404

**IR** (neat) ν_max_: 2956, 1741, 1683, 1474, 1321, 1213, 993, 668 cm^-1^

**TLC**: R*_f_* = 0.32 (Hexane/ethyl acetate 9:1)


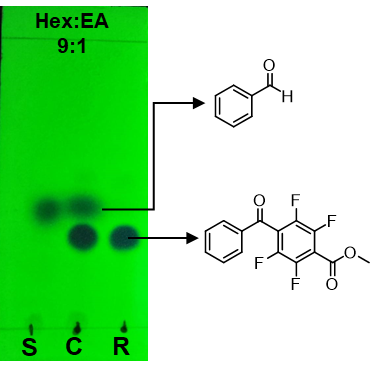


(3-Chloro-2,5,6-trifluoropyridin-4-yl)(phenyl)methanone, 4e

According to **General Procedure A**, benzaldehyde **1a** (10.6 mg, 0.1 mmol, 1.0 equiv) and 3-chloro-2,4,5,6-tetrafluoropyridine **2f** (18.6 mg, 0.1 mmol, 1.0 equiv) were used to obtain crude residue, which was purified by silica gel column chromatography (Hexane) to afford the **4e** as colorless oil (12.8 mg, 0.047 mmol, 47%­).

**^1^H NMR** (300 MHz, CDCl_3_) *δ* 7.84 (d, *J* = 7.0 Hz, 2H), 7.73 (t, *J* = 7.5 Hz, 1H,), 7.56 (t, *J* = 7.9 Hz, 2H) ppm.

**^13^C{^1^H} NMR** (101 MHz, CDCl_3_) *δ* 186.4, 152.4 – 150.0 (m), 148.4 – 146.1 (m), 142.2, 142.0, 140.7 – 137.8 (m), 135.7, 134.1, 129.7, 129.4, 110.8 – 109.9 (m) ppm.

**^19^F NMR** (282 MHz, CDCl_3_) *δ* -72.8 (dd, *J* = 29.2, 11.7 Hz), -86.0 (dd, *J* = 22.0, 11.7 Hz), -143.5 (dd, *J* = 29.1, 22.1 Hz) ppm.

*[See NMR spectra](#S4e)*

**HRMS** (*m/z*): (EI) calc’d for C_12_H_5_NOF_3_Cl [M]^+^: 271.0006, found 271.0008

**IR** (neat) ν_max_: 1451, 1378, 1262, 890, 830, 737 cm^-1^

**TLC:** R*_f_* = 0.49 (Hexane/ethyl acetate 9:1)


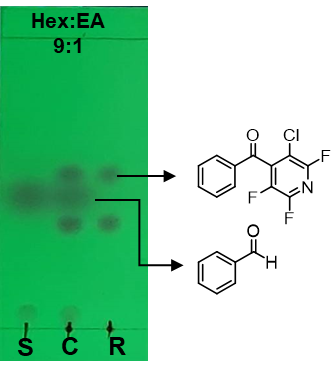


Methyl 4-benzoyl-2,6-difluorobenzoate, 4f

According to **General Procedure A**, benzaldehyde **1a** (10.6 mg, 0.1 mmol, 1.0 equiv) and methyl-2,4,6-trifluorobenzoate **2g** (19.0 mg, 0.1 mmol, 1.0 equiv) were used to obtain crude residue, which was purified by silica TLC plate (Hexane) to afford the **4f** as white solid (7.2 mg, 0.026 mmol, 26%).

**^1^H NMR** (400 MHz, CDCl_3_) *δ* 7.78 (dt, *J* = 7.0, 1.4 Hz, 2H), 7.67 – 7.63 (m, 1H), 7.54 – 7.50 (m, 2H), 7.39 – 7.35 (m, 2H), 3.99 (s, 3H) ppm.

**^13^C{^1^H} NMR** (101 MHz, CDCl_3_) *δ* 193.1, 161.5 (d, *J* = 6.1 Hz), 160.1 (d, *J* = 242.9 Hz), 141.8 – 141.7 (m), 135.8, 133.5, 130.0, 128.7, 114.1, 113.3 (d, *J* = 24.2 Hz), 53.1 ppm.

**^19^F NMR** (282 MHz, CDCl_3_) *δ* -108.5 ppm.

*[See NMR spectra](#S4f)*

**HRMS** (*m/z*): (EI) calc’d for C_15_H_10_F_2_O_3_ [M]^+^: 276.0593, found 276.0596

**IR** (neat) ν_max_: 2955, 2924, 2851, 1739, 1421, 1334, 1240, 1045, 725 cm^-1^

**TLC**: R*_f_* = 0.18 (Hexane/ethyl acetate 9:1)


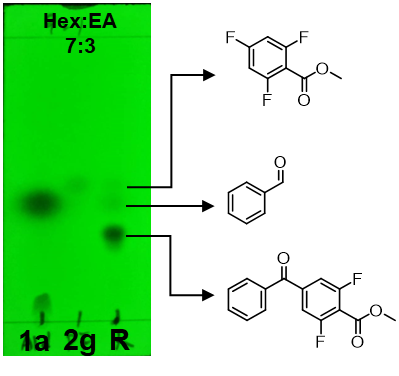


(1*R*,2*S*,5*R*)-2-Isopropyl-5-methylcyclohexyl 4-(perfluoroisonicotinoyl)benzoate, 5a

According to **General Procedure A**, (1*R*,2*S*,5*R*)-2-isopropyl-5-methylcyclohexyl 4-formylbenzoate **1z** (28.8 mg, 0.1 mmol, 1.0 equiv) and pentafluoropyridine **2a** (16.9 mg, 0.1 mmol, 1.0 equiv) were used to obtain crude residue, which was purified by silica gel column chromatography (Hexane) to afford the **5a** as colorless liquid (31.0 mg, 0.071 mmol, 71%­).

**^1^H NMR** (300 MHz, CDCl_3_) *δ* 8.20 (d, *J* = 8.4 Hz, 2H), 7.92 (d, *J* = 8.2 Hz, 2H), 4.96 (td, *J* = 10.9, 4.4 Hz, 1H), 2.12 (d, *J* = 11.8 Hz, 1H), 1.92 (pd, *J* = 9.1, 2.7 Hz, 1H), 1.77 – 1.70 (m, 2H), 1.63 – 1.53 (m, 2H), 1.25 – 1.06 (m, 2H), 0.99 – 0.90 (m, 7H), 0.79 (d, *J* = 7.0 Hz, 3H). ppm.

**^13^C{^1^H} NMR** (101 MHz, CDCl_3_) *δ* 184.0, 164.5, 144.9 – 142.2 (m), 140.0 – 137.1 (m), 137.4, 136.9, 131.1 – 130.7 (m), 130.3, 129.6, 76.0, 47.2, 40.8, 34.2, 31.4, 26.5, 23.6, 21.9, 20.7, 16.4 ppm.

**^19^F NMR** (282 MHz, CDCl_3_) *δ* -87.6 (ddd, *J* = 26.5, 26.4, 9.8 Hz), -141.3 – -141.5 (m) ppm.

[*See NMR spectra*](#S7a)

**HRMS** (*m/z*): (ESI) calc’d for C_23_H_23_O_3_NF_4_^23^Na [M+Na]^+^: 460.1506, found: 460.1503

**IR** (neat) ν_max_: 2959, 2928, 1714, 1688, 1461, 1270, 1109, 961, 826 cm^-1^

**TLC**: R*_f_* = 0.58 (Hexane/ethyl acetate 9:1)


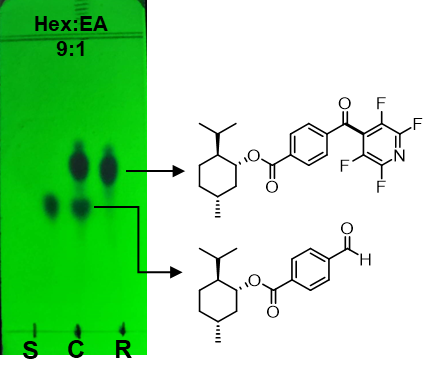


(8*R*,9*S*,13*S*,14*S*)-8,13-Dimethyl-17-oxo-7,8,9,11,12,13,14,15,16,17-decahydro-6*H*-cyclopenta[*a*]phenanthren-3-yl 4-(perfluoroisonicotinoyl)benzoate, 5b

According to **General Procedure A**, (8*R*,9*S*,13*S*,14*S*)-8,13-Dimethyl-17-oxo-7,8,9,11,12,13,14,15,16,17-decahydro-6*H*-cyclopenta[*a*]phenanthren-3-yl 4-formylbenzoate **1aa** (41.7 mg, 0.1 mmol, 1.0 equiv) and pentafluoropyridine **2a** (16.9 mg, 0.1 mmol, 1.0 equiv) were used to obtain crude residue, which was purified by silica gel column chromatography (Hexane) to afford the **5b** as white solid (28.3 mg, 0.050 mmol, 50%­).

**^1^H NMR** (300 MHz, CDCl_3_) *δ* 8.36 (d, *J* = 8.5 Hz, 2H), 7.99 (d, *J* = 8.3 Hz, 2H), 7.36 (d, *J* = 8.5 Hz, 1H), 7.01 – 6.95 (m, 2H), 2.95 (dd, *J* = 9.0, 4.2 Hz, 2H), 2.56 – 1.96 (m, 7H), 1.69 – 1.44 (m, 6H), 0.93 (s, 3H). ppm.

**^13^C{^1^H} NMR** (101 MHz, CDCl_3_) *δ* 220.7, 183.9, 163.9, 148.4, 144.9 – 142.3 (m), 140.0 – 137.0 (m), 138.3, 137.9, 137.9, 135.6, 130.9, 130.7 – 130.5 (m), 129.7, 126.6, 121.4, 118.5, 50.4, 47.9, 44.1, 37.9, 35.8, 34.1, 31.5, 29.4, 26.3, 25.7, 21.5, 13.8 ppm.

**^19^F NMR** (282 MHz, CDCl_3_) *δ* -87.3 – -87.5 (m), -141.1 – -141.3 (m) ppm.

[*See NMR spectra*](#S7b)

**HRMS** (*m/z*): (EI) calc’d for: C_32_H_27_NO_4_F_4_ [M]^+^: 565.1871, found 565.1869

**IR** (neat) ν_max_: 2932, 2863, 1735, 1687, 1462, 1260, 1075, 962, 908, 731 cm^-1^

**M.P.:** 183 ℃

**TLC**: R*_f_* = 0.58 (Hexane/ethyl acetate 9:1)


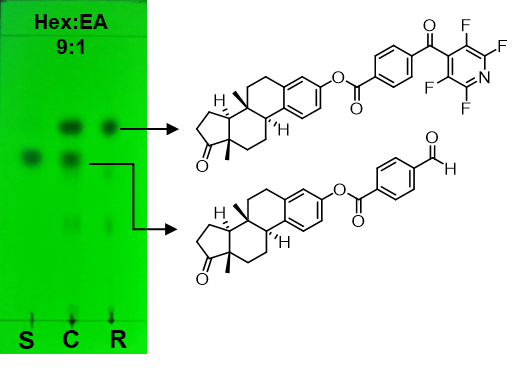


(3*S*,8*S*,9*S*,10*R*,13*S*,14*S*,17*S*)-17-Acetyl-8,10,13-trimethyl-2,3,4,7,8,9,10,11,12,13,14,15,16,17-tetradecahydro-1*H*-cyclopenta[*a*]phenanthren-3-yl 4-(perfluoroisonicotinoyl)benzoate, 5c

According to **General Procedure A**, (3*S*,8*S*,9*S*,10*R*,13*S*,14*S*,17*S*)-17-acetyl-8,10,13-trimethyl-2,3,4,7,8,9,10,11,12,13,14,15,16,17-tetradecahydro-1*H*-cyclopenta[*a*]phenanthren-3-yl 4-formylbenzoate **1ab** (46.2 mg, 0.1 mmol, 1.0 equiv) and pentafluoropyridine **2a** (16.9 mg, 0.1 mmol, 1.0 equiv) were used to obtain crude residue, which was purified by silica gel column chromatography (Hexane/ethyl acetate 15:1) to afford the **5c** as white solid (30.6 mg, 0.050 mmol, 50%­).

**^1^H NMR** (400 MHz, CDCl_3_) *δ* 8.20 (d, *J* = 8.6 Hz, 2H), 7.91 (d, *J* = 8.4 Hz, 2H), 5.43 (dd, *J* = 5.2, 1.9 Hz, 1H), 4.93 – 4.85 (m, 1H), 2.54 (t, *J* = 8.9 Hz, 1H), 2.48 (d, *J* = 7.4 Hz, 2H), 2.22 – 2.16 (m, 1H), 2.12 (s, 3H), 2.05 – 2.00 (m, 2H), 1.94 (dt, *J* = 13.5, 3.4 Hz, 1H), 1.78 – 1.60 (m, 5H), 1.57 – 1.46 (m, 4H), 1.27 – 1.19 (m, 3H), 1.07 (s, 3H), 1.05 – 1.02 (m, 1H), 0.64 (s, 3H) ppm.

**^13^C{^1^H} NMR** (101 MHz, CDCl_3_) *δ* 209.5, 184.0, 164.4, 144.8 – 142.3 (m), 140.0 – 137.0 (m), 139.3, 137.4, 136.8, 130.8 (t, *J* = 53.2 Hz), 130.4, 129.5, 122.8, 122.8, 75.5, 63.6, 56.8, 49.8, 43.9, 38.7, 38.0, 36.9, 36.6, 31.8, 31.8, 31.5, 27.7, 24.5, 22.8, 21.0, 19.3, 13.2 ppm.

**^19^F NMR** (282 MHz, CDCl_3_) *δ* -87.3 – -87.5 (m), -141.1 – -141.3 (m) ppm.

[*See NMR spectra*](#S7c)

**HRMS** (*m/z*): (EI) calc’d for C_34_H_35_NO_4_F_4_ [M]^+^: 597.2497, found: 597.2494

**IR** (neat) ν_max_: 2939, 2889, 1721, 1690, 1458, 1280, 1116, 964, 828, 728 cm^-1^

**M.P.:** 193 ℃

**TLC**: R*_f_* = 0.24 (Hexane/ethyl acetate 9:1)


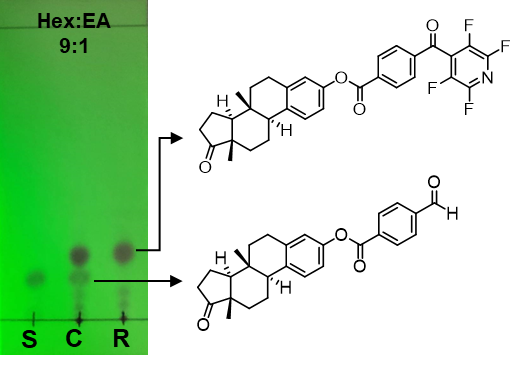


(3*S*,8*S*,9*S*,10*R*,13*R*,14*S*,17*R*)-10,13-Dimethyl-17-((*R*)-6-methylheptan-2-yl) 2,3,4,7,8,9,10,11,12,13,14,15,16,17-tetradecahydro-1*H*-cyclopenta[*a*]phenanthren-3-yl 4-(perfluoroisonicotinoyl)benzoate, 5d

According to **General Procedure A**, (3*S*,8*S*,9*S*,10*R*,13*R*,14*S*,17*R*)-10,13-dimethyl-17-((*R*)-6-methylheptan-2-yl)-2,3,4,7,8,9,10,11,12,13,14,15,16,17-tetradecahydro-1*H*-cyclopenta[*a*]phenanthren-3-yl 4-formylbenzoate **1ac** (51.8 mg, 0.1 mmol, 1.0 equiv) and pentafluoropyridine **2a** (16.9 mg, 0.1 mmol, 1.0 equiv) were used to obtain crude residue, which was purified by silica gel column chromatography (Hexane) to afford the **5d** as white solid (60.7 mg, 0.091 mmol, 91%­).

**^1^H NMR** (300 MHz, CDCl_3_) *δ* 8.20 (d, *J* = 8.5 Hz, 2H), 7.91 (d, *J* = 8.2 Hz, 2H), 5.43 (d, *J* = 4.3 Hz, 1H), 4.94 – 4.84 (m, 1H), 2.47 (d, *J* = 7.5 Hz, 2H), 2.05 – 1.13 (m, 25H), 1.07 (s, 3H), 1.04 – 0.96 (m, 3H), 0.92 (d, *J* = 6.4 Hz, 2H), 0.86 (dd, *J* = 6.6, 1.3 Hz, 6H), 0.69 (s, 3H) ppm.

**^13^C{^1^H} NMR** (101 MHz, CDCl_3_) *δ* 184.0, 164.5, 144.9 – 142.2 (m), 139.7 – 137.0 (m), 139.3, 137.4, 136.9, 131.1 – 130.7 (m), 130.4, 129.5, 123.2, 75.7, 56.7, 56.1, 50.0, 42.3, 39.7, 39.5, 38.1, 37.0, 36.6, 36.2, 35.8, 31.9, 31.9, 28.2, 28.0, 27.8, 24.3, 23.8, 22.8, 22.5, 21.0, 19.3, 18.7, 11.9 ppm.

**^19^F NMR** (282 MHz, CDCl_3_) *δ* -87.4 – -87.7 (m), -141.2 – -141.4 (m) ppm.

*[See NMR spectra](#S7d)*

**HRMS** (*m/z*): (EI) calc’d for C_40_H_49_NO_3_F_4_ [M]^+^: 667.3643, found: 667.3642

**IR** (neat) ν_max_: 2935, 2867, 1721, 1458, 1279, 1108, 964, 829, 750 cm^-1^

**M.P.**: 182 ℃


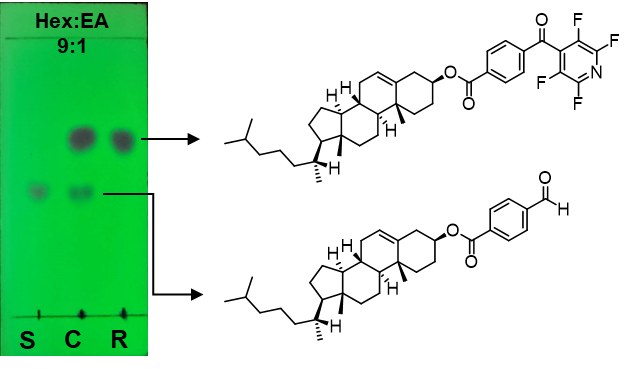
**TLC**: R*_f_* = 0.63 (Hexane/ethyl acetate 9:1)

(1*R*,2*S*,5*R*)-2-Isopropyl-5-methylcyclohexyl-4-benzoyl-2,3,5,6-tetrafluorobenzoate, 5e

According to **General Procedure A**, benzaldehyde **1a** (10.6 mg, 0.1 mmol, 1.0 equiv) and (1*R*,2*S*,5*R*)-2-isopropyl-5-methylcyclohexyl-2,3,4,5,6-pentafluorobenzoate **2h** (35.0 mg, 0.1 mmol, 1.0 equiv) were used to obtain crude residue, which was purified by silica gel column chromatography (Hexane/ethyl acetate 9:1) to afford the **5e** as pale-yellow oil (34.9 mg, 0.080 mmol, 80 %­).

**^1^H NMR** (300 MHz, CDCl_3_) *δ* 7.90 – 7.82 (m, 2H), 7.72 – 7.65 (m, 1H), 7.57 – 7.48 (m, 2H), 5.03 (td, *J* = 10.9, 4.4 Hz, 1H), 2.19 (dd, *J* = 11.8, 3.0 Hz, 1H), 2.08 – 1.95 (m, 1H), 1.80 – 1.68 (m, 2H), 1.59 – 1.45 (m, 1H), 1.30 – 1.03 (m, 3H), 0.99 – 0.89 (m, 6H), 0.87 – 0.79 (m, 3H) ppm.

**^13^C{^1^H} NMR** (101 MHz, CDCl_3_) *δ* 185.4, 158.8, 145.4 – 144.3 (m), 142.9 – 141.8 (m), 135.6, 135.1, 129.7, 129.1, 120.8 (t, *J* = 57.5), 115.3 (t, *J* = 49.2), 78.0, 46.9, 40.6, 34.0, 31.5, 25.9, 23.1, 21.9, 20.8, 15.9 ppm.

**^19^F NMR** (282 MHz, CDCl_3_) *δ* -138.3 – -138.5 (m), -138.5 – -139.7 (m) ppm.

*[See NMR spectra](#S7e)*

**HRMS** (*m/z*): (EI) calc’d for C_24_H_24_F_4_O_3_: 436.1656, found 436.1653

**IR** (neat) ν_max_: 2957, 2927, 2871, 1731, 1475, 1309, 1208, 990 cm^-1^

**TLC**: R*_f_* = 0.56 (Hexane/ethyl acetate 9:1)


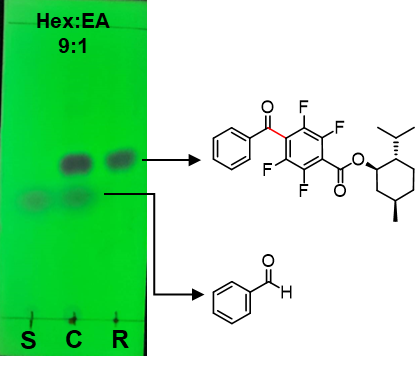


(3*S*,8*S*,9*S*,10*R*,13*R*,14*S*)-10,13-Dimethyl-17-((*R*)-6-methylheptan-2-yl)-2,3,4,7,8,9,10,11,12,13,14,15,16,17-tetradecahydro-1*H*-cyclopenta[*a*]phenanthren-3-yl 4-benzoyl-2,3,5,6-tetrafluorobenzoate, 5f

According to **General Procedure A**, benzaldehyde **1a** (10.6 mg, 0.1 mmol, 1.0 equiv) and (3*S*,8*S*,9*S*,10*R*,13*R*,14*S*)-10,13-Dimethyl-17-((*R*)-6-methylheptan-2-yl)-2,3,4,7,8,9,10,11,12,13,14,15,16,17-tetradecahydro-1*H*-cyclopenta[*a*]phenanthren-3-yl 2,3,4,5,6-pentafluorobenzoate **2i** compound with methane (1:1) (59.7 mg, 0.1 mmol, 1.0 equiv) were used to obtain crude residue, which was purified by silica gel column chromatography (Hexane/ethyl acetate 9:1) to afford the **5f** as white solid (61.9 mg, 0.093 mmol, 93%­).

**^1^H NMR** (300 MHz, CDCl_3_) *δ* 7.86 (d, *J* = 7.7 Hz, 2H), 7.69 (t, *J* = 7.4 Hz, 1H), 7.53 (t, *J* = 7.6 Hz, 2H), 5.46 (d, *J* = 5.0 Hz, 1H), 5.04 – 4.88 (m, 1H), 2.50 (d, *J* = 8.0 Hz, 2H), 2.06 – 1.69 (m, 9H), 1.61 – 1.45 (m, 12H), 1.40 – 1.10 (m, 4H), 1.06 (s, 3H), 1.02 – 0.97 (m, 3H), 0.92 (d, *J* = 6.5 Hz, 2H), 0.87 (dd, *J* = 6.6, 1.4 Hz, 6H), 0.69 (s, 3H) ppm

**^13^C{^1^H}** NMR (101 MHz, CDCl_3_) *δ* 185.4, 158.5, 158.4, 145.4 – 142.9 (m), 144.5 – 141.6 (m), 144.2, 139.0, 135.6, 135.2, 129.7, 129.1, 123.4, 123.4, 121.2 – 121.0 (m), 115.1 – 115.0 (m), 56.7, 56.1, 50.0, 42.3, 39.7, 39.5, 37.9, 36.9, 36.6, 36.2, 35.8, 31.9, 31.8, 28.2, 28.0, 27.6, 24.3, 23.8, 22.8, 22.5, 21.0, 19.3, 18.7, 11.8 ppm.

**^19^F NMR** (282 MHz, CDCl_3_) *δ* -138.2 – -138.30, -139.6 – -139.7 ppm.

*[See NMR spectra](#S7f)*

**HRMS** (*m/z*): (EI) calc’d for C_41_H_50_F_4_O_3_ [M]^+^: 666.3691, found 666.3691

**IR** (neat) ν_max_: 2935, 2869, 1733, 1475, 1323, 1232, 1208, 992, 733 cm^-1^

**M.P.**: 116 ℃


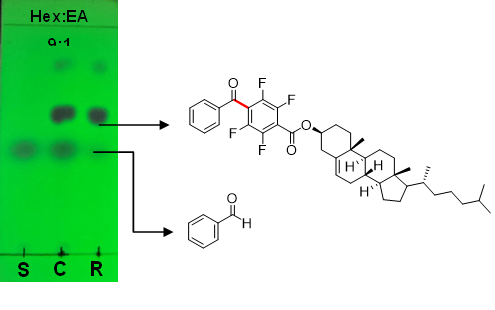
**TLC**: R*_f_* = 0.61 (Hexane/ethyl acetate 9:1)

(3*S*,8*S*,9*S*,10*R*,13*S*,14*S*,17*S*)-17-Acetyl-8,10,13-trimethyl-2,3,4,7,8,9,10,11,12,13,14,15,16,17-tetradecahydro-1*H*-cyclopenta[*a*]phenanthren-3-yl 4-benzoyl-2,3,5,6-tetrafluorobenzoate, 5g

According to **General Procedure A**, benzaldehyde **1a** (10.6 mg, 0.1 mmol, 1.0 equiv) and (3*S*,8*S*,9*S*,10*R*,13*S*,14*S*,17*S*)-17-Acetyl-8,10,13-trimethyl-2,3,4,7,8,9,10,11,12,13,14,15,16,17-tetradecahydro-1*H*-cyclopenta[*a*]phenanthren-3-yl 2,3,4,5,6-pentafluorobenzoate **2j** (52.4 mg, 0.1 mmol, 1.0 equiv) were used to obtain crude residue, which was purified by silica gel column chromatography (Hexane/ethyl acetate 9:1) to afford the **5g** as pale-yellow oil (32.9 mg, 0.054 mmol, 55 %­).

**^1^H NMR** (300 MHz, CDCl_3_) *δ* 7.86 – 7.83 (m, 2H), 7.71 – 7.65 (m, 1H), 7.55 – 7.50 (m, 2H), 5.46 – 5.44 (m, 1H), 5.01 – 4.90 (m, 1H), 2.57 – 2.47, (m, 3H), 2.20 – 1.91 (m, 8H), 1.83 – 1.41 (m, 8H), 1.26 – 1.16 (m, 3H), 1.12 – 1.05 (m, 4H), 0.64 (s, 3H) ppm.

**^13^C{^1^H} NMR** (101 MHz, CDCl_3_) *δ* 209.5, 185.4, 158.5, 145.6 – 142.9 (m), 141.8 – 138.0 (m), 139.0, 135.6, 135.2, 129.7, 129.1, 123.1, 121.2 – 120.8 (m), 115.4 – 114.9 (m), 63.6, 56.8, 49.8, 43.9, 38.7, 37.8, 36.9, 36.6, 31.8, 31.5, 27.6, 24.4, 22.8, 21.0, 19.3, 13.2 ppm.

**^19^F NMR** (282 MHz, CDCl_3_) *δ* -138.1 – -138.3 (m), -139.5 – -139.7 (m) ppm.

[*See NMR spectra*](#S7g)

**HRMS** (*m/z*): (EI) calc’d for C_35_H_36_O_4_F_4_ [M]^+^: 596.2544, found 596.2546

**IR** (neat) ν_max_: 2939, 2853, 2252, 1597, 1475, 1322, 1208, 1051, 993 cm^-1^

**M.P.**: 138 ℃

**TLC**: R*_f_* = 0.20 (Hexane/ethyl acetate 9:1)


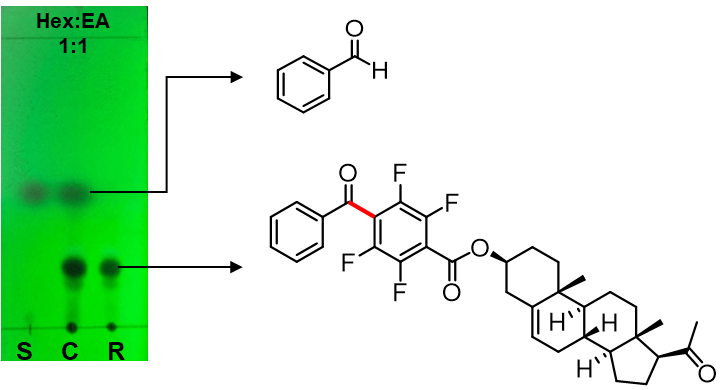


2.7 Large-scale syntheses of NHC-Catalyzed S*_N_*Ar reaction

Gram-scale synthesis of 4a

According to **General Procedure B**, benzaldehyde **1a** (0.74 g, 7 mmol, 1.0 equiv) and pentafluoropyridine **2a** (1.30 g, 7.7 mmol, 1.1 equiv) were used to obtain crude residue, which was purified by silica gel column chromatography (Hexane) to afford the **3a** as pale-yellow liquid (1.1810 g, 4.63 mmol, 66%­).

**
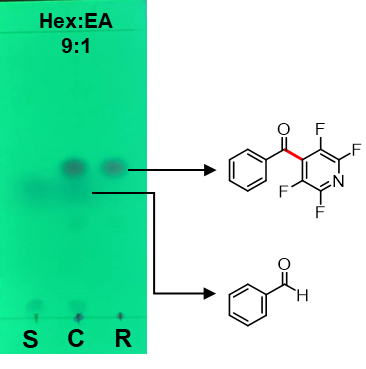

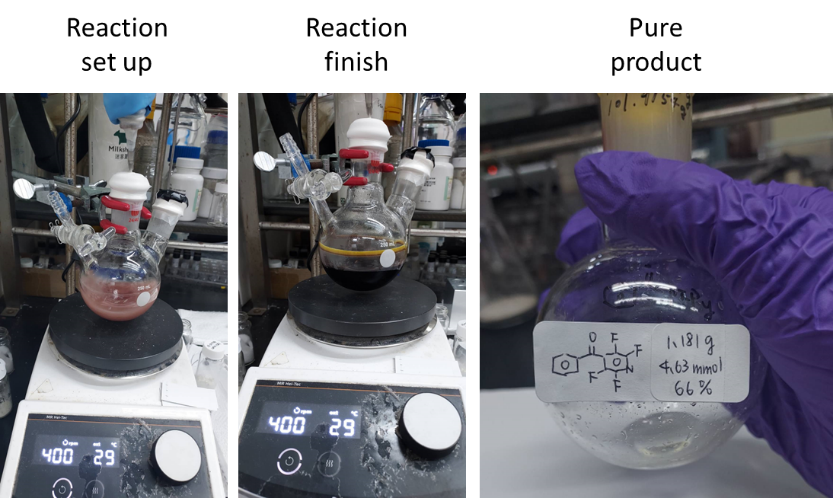
**

Gram-scale synthesis of 3r

According to **General Procedure B**, 3-bromo-4-fluorobenzaldehyde **1s** (1.41 g, 7 mmol, 1.0 equiv) and pentafluoropyridine **2a** (1.3 g, 7.7 mmol, 1.1 equiv) were used to obtain crude residue, which was purified by silica gel column chromatography (Hexane) to afford the **3s** as pale-yellow liquid (1.7438 g, 4.95 mmol, 71%­).

**
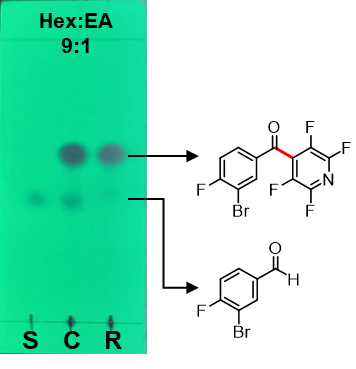

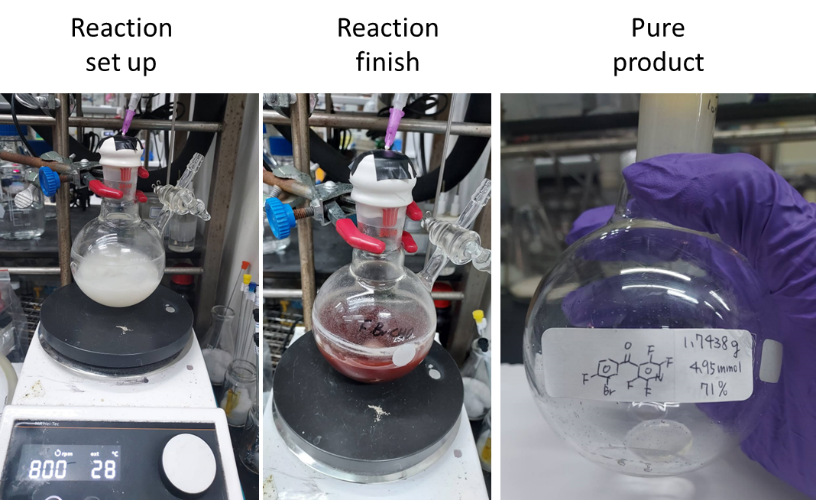
**

3. Synthesis of bioisostere

3.1 Procedure of synthesis

Synthesis of compound 6

In the nitrogen-filled glovebox, an oven-dried tube equipped with a stirring bar was added bis(triphenylphosphine)palladium chloride (4.2 mg, 0.006 mmol, 3.0 mol%), potassium acetate (58.9 mg, 0.6 mmol, 3.0 equiv) and bis(pinacolato)diboron (101.6 mg, 0.4 mmol, 2.0 equiv). The tube was added 0.5 mL preparative 0.2 M solution **3r** (70.4 mg, 0.2 mmol, 1.0 equiv) in dioxane followed by additional 0.5 mL dioxane. Then the tube was sealed and moved out, stirred at 130 ℃ for further 1.5 hr. After the reaction completed, the crude was purified by silica gel column chromatography (the height of packing silica gel: 8.0 cm, Hexane/ethyl acetate 25:1) afford **6** (56.7 mg, 0.142 mmol, 71%).

**^1^H NMR** (300 MHz, CDCl_3_) *δ* 8.24 (dd, *J* = 5.4, 2.5 Hz, 1H), 7.95 (ddd, *J* = 8.1, 5.0, 2.5 Hz, 1H), 7.20 (t, *J* = 8.6 Hz, 1H), 1.37 (s, 12H) ppm.

**^13^C{^1^H} NMR** (101 MHz, CDCl_3_) *δ* 182.9, 171.4 (d, *J* = 264.8 Hz), 144.9 – 142.1 (m), 140.0 – 137.0 (m), 139.5 (d, *J* = 10.5 Hz), 135.1 (d, *J* = 11.1 Hz), 131.2 (t, *J* = 18.1 Hz), 130.8 (d, *J* = 2.6 Hz), 116.9 (d, *J* = 25.3 Hz), 84.6, 24.8 ppm.

**^19^F NMR** (282 MHz, CDCl_3_) *δ* -87.8 – -88.0 (m), -89.1, -141.4 – -141.6 (m) ppm.

[*See NMR spectra*](#S8)

**HRMS** (*m/z*): (EI) calc’d for C_18_H_15_BNO_3_F_5_ [M]^+^: 399.1060, found: 399.1062

**IR** (neat) ν_max_: 2980, 2957, 1607, 1464, 1352, 1286, 1143, 853 cm^-1^

**TLC**: R*_f_* = 0.25 (Hexane/ethyl acetate 9:1)


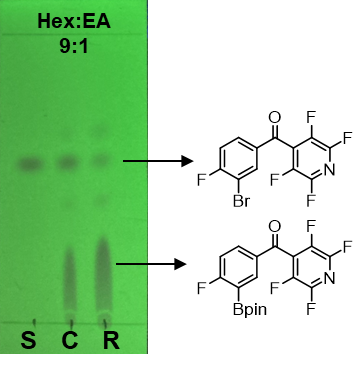


Synthesis of compound 7

In the nitrogen-filled glovebox, an oven-dried tube (5.0 mL) equipped with a stirring bar was added bis(benzonitrile)palladium dichloride (1.92 mg, 0.005 mmol, 5.0 mol%), tricyclohexylphosphine (2.8 mg, 0.01 mmol, 10.0 mol%), sodium carbonate (21.2 mg, 0.2 mmol, 2.0 equiv) and **S1** (25.0 mg, 0.1 mmol, 1.0 equiv). The tube was added 0.5 mL preparative 0.2 M solution **6** (0.1 mmol, 1.0 equiv) in dioxane followed by additional 0.5 mL dioxane. Then the tube was sealed, moved out and added 0.01 mL degassed water then stirred at 100 ℃ for further 2.5 hr. After the reaction completed, the crude was washed by ethyl acetate and water. Organic mixture was purified by preparative TLC plate (Hexane/ethyl acetate 7:3) to afford **7** (32.6 mg, 0.067 mmol, 67%).

**^1^H** NMR (300 MHz, DMSO-*d*_6_) *δ* 9.11 (s, 1H), 8.37 – 8.32 (m, 2H), 7.74 (t, *J* = 9.0 Hz, 1H), 7.53 (s, 2H), 7.21 (s, 1H), 3.77 (t, *J* = 4.7 Hz, 4H), 3.44 (t, *J* = 4.9 Hz, 4H) ppm.

**^13^C{^1^H} NMR** (101 MHz, DMSO-*d*_6_) *δ* 183.3, 163.6 (d, *J* = 259.6 Hz), 159.7, 154.8, 154.7, 152.1, 144.4 – 141.7 (m), 140.1 – 137.2 (m), 134.2 (d, *J* = 5.2 Hz), 133.9 (d, *J* = 10.2 Hz), 131.4, 129.9 (t, *J* = 18.8 Hz), 127.1, 126.3 (d, *J* = 16.9 Hz), 119.3, 118.0 (d, *J* = 23.0 Hz), 116.8, 106.1, 65.8, 46.9 ppm.

**^19^F NMR** (282 MHz, DMSO-*d*_6_) *δ* -90.0 – -90.3 (m), -102.8, -142.7 – -143.0 (m) ppm.

[*See NMR spectra*](#S9)

**HRMS** (*m/z*): (ESI) calc’d for C_24_H_16_O_2_N_4_F_5_ [M+H]^+^: 487.1188, found 487.1186

**IR** (neat) ν_max_: 2969, 2955, 1613, 1464, 1269, 968 cm^-1^

**TLC**: R*_f_* = 0.50 (Hexane/ethyl acetate 1:1)


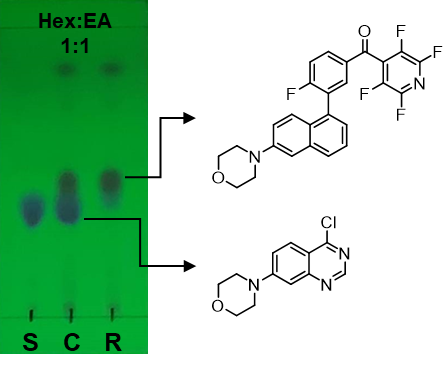


Synthesis of PF-D1

Under the nitrogen gas, an oven-dried tube equipped with a stirring bar was added sodium borohydride (5.7 mg, 0.15 mmol, 1.5 equiv). At 0 ℃, the tube was added 0.5 mL preparative 0.2 M solution **7** (48.6 mg, 0.1 mmol, 0.1 equiv) in methanol followed by additional 0.5 mL methanol. The mixture was stirred at room temperature for 2 hr. After the reaction completed, the crude was washed by ethyl acetate and water. Organic mixture was purified by preparative TLC plate (Hexane/ethyl acetate 7:3) to afford **PF-D1** as a yellow solid (34.0 mg, 0.070 mmol, 70%).

**^1^H NMR** (300 MHz, DMSO-*d*_6_) *δ* 9.10 (s, 1H), 7.77 – 7.67 (m, 2H), 7.52 – 7.42 (m, 4H), 7.20 (s, 1H), 6.92 (s, 1H), 6.28 (s, 1H), 3.77 (t, *J* = 4.7 Hz, 4H), 3.44 (t, *J* = 4.8 Hz, 4H) ppm.

**^13^C{^1^H} NMR** (101 MHz, DMSO-*d*_6_) *δ* 161.1, 158.5 (d, *J* = 247.5 Hz), 155.0, 154.5, 152.1, 144.3 – 141.6 (m), 141.0 – 138.1 (m), 137.1 (d, *J* = 2.1 Hz), 136.4 (t, *J* = 13.6 Hz), 129.2 (d, *J* = 8.7 Hz), 128.8, 127.2 (d, *J* = 1.6 Hz), 124.6 (d, *J* = 15.9 Hz), 119.0, 116.7, 116.1 (d, *J* = 22.3 Hz), 106.3, 65.8, 64.9, 46.9 ppm.

**^19^F NMR** (282 MHz, CDCl_3_) *δ* -92.2 – -92.5 (m), -116.2, -143.7, – -143.9 (m) ppm.

[*See NMR spectra*](#SPFD1)

**HRMS** (*m/z*): (ESI) calc’d for C_24_H_18_O_2_N_4_F_5_ [M+H]^+^: 489.1344, found 489.1341

**IR** (neat) ν_max_: 3222, 2956, 2922, 2852, 1612, 1468, 1235, 1124, 948, 824 cm^-1^

**M.P.**: 128 ℃

**TLC**: R*_f_* = 0.31 (Hexane/ethyl acetate 9:1)


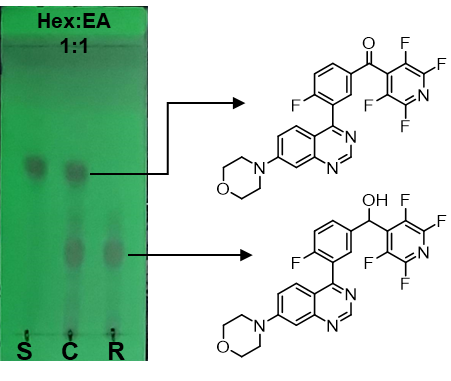

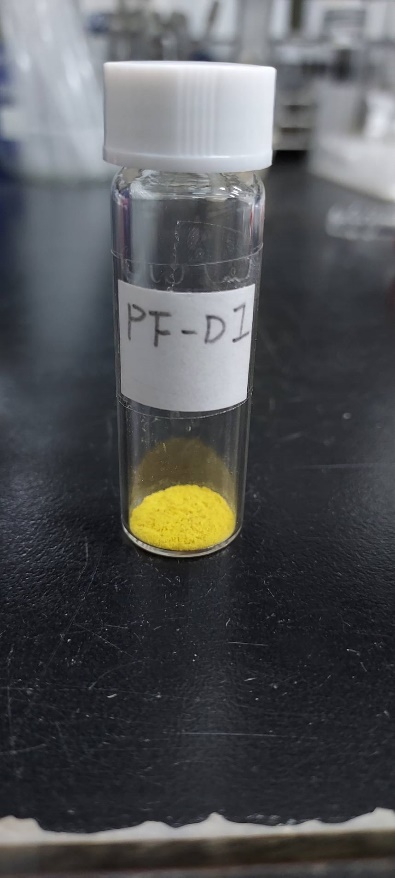


3.2 Procedure comparison

Compared to the existing literature methods^[[3]](#footnote-3)^, our protocol offers several advantages, such as lower cost (Price information comes from the official selling price of BLD pharm on September 27, 2023), shorter reaction time, higher yield (Only some steps have documented yields in the literature, and 29% of by-product **R5’** will be obtained in the harsh S*_N_*Ar reaction.), and broader substrate scope. Although the pharmacological properties of compound **PF-D1** remain to be elucidated, we have demonstrated the utility and versatility of our methodology for constructing perfluorinated aryl ketones in drug design, as well as the modifiability of the ketone group for further functionalization. Our synthesis method opens up new possibilities for the discovery and optimization of novel drug candidates based on perfluorinated aryl ketones.

4. Experimental procedure of LIBs test

4.1 Preparation of LNiMnO cathode material pole piece and electrolyte

Preparation of pole pieces

1. First, mix LiNi_0.5_Mn_1.5_O_4_, PVDF, and Super P in a weight ratio of 70:10:20. Then, add a specified amount of *N*-methyl-2-pyrrolidone (NMP) as a solvent and stir the mixture for approximately 20 hr to create a thick slurry. Prior to using a coating machine, ensure the slurry is homogenized using a homogenizer for even dispersion.

2. Utilize a coating machine to apply the slurry evenly onto battery-grade aluminum foil while maintaining a thickness of 200 μm. Solidify the electrode surface at 110 ℃ using a heater, and then subject it to a 12-hour heat treatment at 80 ℃ in a vacuum oven to remove the solvent.

3. Using an automatic rolling machine, roll the dried electrode sheet with a 59% rolling rate.

4. Cut the electrode sheets into circular pieces with a 1.3 cm diameter by an electrode sheet cutter, and then transfer them into an argon-filled glovebox to assemble coin cell batteries.

Preparation of electrolyte

Inside the glovebox, thoroughly mix ethylene carbonate (EC) and diethyl carbonate (DEC) in a 1:1 (v/v) ratio. Then, add 1 M lithium hexafluorophosphate (LiPF_6_) until it completely dissolves, creating the standard electrolyte. The additives are dissolved in the standard electrolyte at a weight concentration of 0.05 wt%.

Preparation of half-Cell

The processed LiNi_0.5_Mn_1.5_O_4_ cathode material electrode is placed into an argon gas-filled glovebox. The battery being prepared in this experiment is a CR2032 half-cell with the following assembly steps:

1. Weigh the electrode, position it centrally in the lower cover, ensuring that the coated side faces upward.
2. Submerge the PP separator in the electrolyte to ensure full saturation and place it on top of the electrode. This ensures effective isolation of lithium metal from direct contact with the cathode material to prevent short circuits.
3. Attach lithium metal, facing downward, to the spacer on the spring tab, directly onto the separator.
4. Seal the cell by affixing the upper cover and using a battery sealing machine to ensure a secure closure.

4.2 Cycle Life Test

In this study, constant current charge-discharge tests are performed. The voltage is charged from the initial voltage to the termination voltage over time and then discharged from the termination voltage back to the initial voltage, completing one cycle. The purpose is to observe voltage variations during this cycle and obtain electrochemical data such as charge-discharge capacity for each cycle and efficiency per cycle. This data is crucial for assessing capacitance retention and the stability of battery lifespan.

5. Proposed mechanism

The potential mechanism for the NHC-catalyzed S*_N_*Ar reaction may commence with the deprotonation of **NHC1** salt, activating the NHC carbene. After activation, the Breslow intermediate form through nucleophilic addition to the aldehyde. Under basic conditions, the Breslow intermediate attacks the 4-position of the perfluoroarene, leading to defluorination and yielding the desired polyfluorinated diarylmethanones.

6. Reference

1. Gardner, S.; Kawamoto, T.; Curran, D. P. Synthesis of 1, 3-dialkylimidazol-2-ylidene boranes from 1, 3-dialkylimidazolium iodides and sodium borohydride. *J. Org. Chem.* **2015**, *80*, 19, 9794–9797

2. Lebeuf, R.; Hirano, K.; Glorius, F. Palladium-catalyzed C-allylation of benzoins and an NHC-catalyzed three component coupling derived thereof: compatibility of NHC-and Pd-catalysts. *Organic Letters* **2008**, *10*, 19, 4243–4246.

3. Fuchss, T.; Emde, U., Buchstaller, H-P.; Mederski, W. Arylquinazolines. U.S. Patent US 2022/0117970 A1, April 21, 2022

7. NMR spectra

^1^H NMR (300 MHz, CDCl_3_) of 2c’, *[See procedure](#P2c)
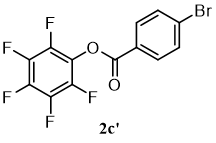
*

^13^C NMR (101 MHz, CDCl_3_) of 2c’ *
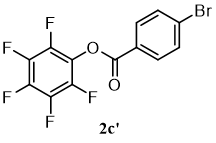
*

^19^F NMR (282 MHz, CDCl_3_) of 2c’ *
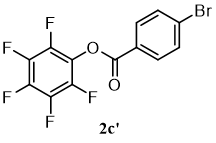
*

^1^H NMR (300 MHz, CDCl_3_) of 2j, *[See procedure](#P2j)*


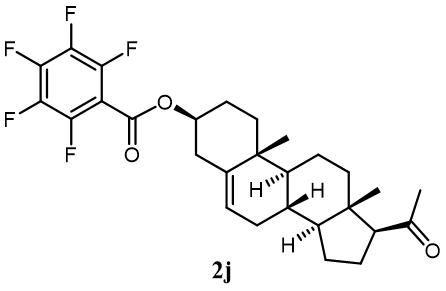


**^13^C NMR** (101 MHz, CDCl_3_) of **2j**


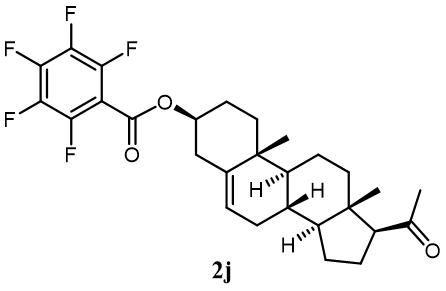


**^19^F NMR** (282 MHz, CDCl_3_) of **2j**
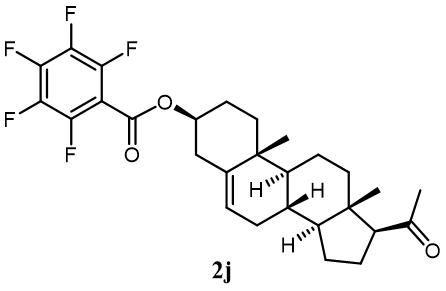


^1^H NMR (300 MHz, *d*-methanol) of NHC1, *[See procedure](#NHC1)*

**^13^C NMR** (101 MHz, CDCl_3_) of **NHC1**

^1^H NMR (300 MHz, CDCl_3_) of NHC4, *[See procedure](#NHC4)*

**^13^C NMR** (101 MHz, CDCl_3_) of **NHC4**

Spectrum of substrate scope

**^1^H NMR** (400 MHz, CDCl_3_) of **3a**, [*See procedure*](#P3a)

**^13^C NMR** (101 MHz, CDCl_3_) of **3a**

**^19^F NMR** (282 MHz, CDCl_3_) of **3a**

**^1^H NMR** (300 MHz, CDCl_3_) of **3****b**, [*See procedure*](#P3b)

**^13^C NMR** (101 MHz, CDCl_3_) of **3****b**

**^19^F NMR** (282 MHz, CDCl_3_) of **3****b**

**^1^H NMR** (300 MHz, CDCl_3_) of **3c**, [*See procedure*](#P3c)

**^13^C NMR** (101 MHz, CDCl_3_) of **3****c**

**^19^F NMR** (282 MHz, CDCl_3_) of **3****c**

**^1^H NMR** (300 MHz, CDCl_3_) of **3d**, [*See procedure*](#P3d)

**^13^C NMR** (101 MHz, CDCl_3_) of **3****d**

**^19^F NMR** (282 MHz, CDCl_3_) of **3****d**

**^1^H NMR** (300 MHz, CDCl_3_) of **3e**, [*See procedure*](#P3e)

**^13^C NMR** (101 MHz, CDCl_3_) of **3****e**

**^19^F NMR** (282 MHz, CDCl_3_) of **3****e**

**^1^H NMR** (300 MHz, CDCl_3_) of **3f**, [*See procedure*](#P3f)

**^13^C NMR** (101 MHz, CDCl_3_) of **3****f**

**^19^F NMR** (376 MHz, CDCl_3_) of **3****f**

**^1^H NMR** (300 MHz, CDCl_3_) of **3g**, [*See procedure*](#P3g)

**^13^C NMR** (101 MHz, CDCl_3_) of **3****g**

**^19^F NMR** (282 MHz, CDCl_3_) of **3****g**

**^1^H NMR** (300 MHz, CDCl_3_) of **3h**, [*See procedure*](#P3h)

**^13^C NMR** (101 MHz, CDCl_3_) of **3****h**

**^19^F NMR** (282 MHz, CDCl_3_) of **3****h**

**^1^H NMR** (300 MHz, CDCl_3_) of **3i**, [*See procedure*](#P3i)

**^13^C NMR** (101 MHz, CDCl_3_) of **3****i**

**^19^F NMR** (282 MHz, CDCl_3_) of **3****i**

**^1^H NMR** (400 MHz, CDCl_3_) of **3j**, [*See procedure*](#P3j)

**^13^C NMR** (101 MHz, CDCl_3_) of **3****j**

**^19^F NMR** (282 MHz, CDCl_3_) of **3****j**

**^1^H NMR** (300 MHz, CDCl_3_) of **3k**, [*See procedure*](#P3k)

**^13^C NMR** (101 MHz, CDCl_3_) of **3****k**

**^19^F NMR** (282 MHz, CDCl_3_) of **3****k**

**^1^H NMR** (300 MHz, CDCl_3_) of **3l**, [*See procedure*](#P3l)

**^13^C NMR** (101 MHz, CDCl_3_) of **3****l**

**^19^F NMR** (282 MHz, CDCl_3_) of **3****l**

**^1^H NMR** (300 MHz, CDCl_3_) of **3m**, [*See procedure*](#P3m)

**^13^C NMR** (101 MHz, CDCl_3_) of **3****m**

**^19^F NMR** (282 MHz, CDCl_3_) of **3****m**

**^1^H NMR** (300 MHz, CDCl_3_) of **3n**, [*See procedure*](#P3n)

**^13^C NMR** (101 MHz, CDCl_3_) of **3****n**

**^19^F NMR** (282 MHz, CDCl_3_) of **3****n**

**^1^H NMR** (300 MHz, CDCl_3_) of **3o**, [*See procedure*](#P3o)

**^13^C NMR** (101 MHz, CDCl_3_) of **3****o**

**^19^F NMR** (282 MHz, CDCl_3_) of **3****o**

**^1^H NMR** (300 MHz, CDCl_3_) of **3p**, [*See procedure*](#P3p)

**^13^C NMR** (101 MHz, CDCl_3_) of **3****p**

**^19^F NMR** (282 MHz, CDCl_3_) of **3****p**

**^1^H NMR** (300 MHz, CDCl_3_) of **3q**, [*See procedure*](#P3q)

**^13^C NMR** (101 MHz, CDCl_3_) of **3****q**

**^19^F NMR** (282 MHz, CDCl_3_) of **3****q**

**^1^H NMR** (300 MHz, CDCl_3_) of **3r**, [*See procedure*](#P3r)

**^13^C NMR** (101 MHz, CDCl_3_) of **3****r**

**^19^F NMR** (282 MHz, CDCl_3_) of **3****r**

**^1^H NMR** (300 MHz, CDCl_3_) of **3s**, [*See procedure*](#P3s)

**^13^C NMR** (101 MHz, CDCl_3_) of **3****s**

**^19^F NMR** (282 MHz, CDCl_3_) of **3****s**

**^1^H NMR** (300 MHz, CDCl_3_) of **3t**, [*See procedure*](#P3t)

**^13^C NMR** (101 MHz, CDCl_3_) of **3****t**

**^19^F NMR** (282 MHz, CDCl_3_) of **3s**

**^1^H NMR** (300 MHz, CDCl_3_) of **3u**, [*See procedure*](#P3u)

**^13^C NMR** (101 MHz, CDCl_3_) of **3****u**

**^19^F NMR** (282 MHz, CDCl_3_) of **3****u**

**^1^H NMR** (300 MHz, CDCl_3_) of **3v**, [*See procedure*](#P3v)

**^13^C NMR** (101 MHz, CDCl_3_) of **3****v**

**^19^F NMR** (282 MHz, CDCl_3_) of **3****v**

**^1^H NMR** (300 MHz, CDCl_3_) of **3w**, [*See procedure*](#P3w)

**^13^C NMR** (101 MHz, CDCl_3_) of **3****w**

**^19^F NMR** (282 MHz, CDCl_3_) of **3****w**

**^1^H NMR** (300 MHz, CDCl_3_) of **3x**, [*See procedure*](#P3x)

**^13^C NMR** (101 MHz, CDCl_3_) of **3****x**

**^19^F NMR** (282 MHz, CDCl_3_) of **3****x**

**^1^H NMR** (300 MHz, CDCl_3_) of **3y**, [*See procedure*](#P3y)

**^13^C NMR** (101 MHz, CDCl_3_) of **3****y**

**^19^F NMR** (282 MHz, CDCl_3_) of **3****y**

**^1^H NMR** (400 MHz, CDCl_3_) of **4a**, [*See procedure*](#P4a)

**^13^C NMR** (101 MHz, CDCl_3_) of **4****a**

**^19^F NMR** (376 MHz, CDCl_3_) of **4****a**

**^1^H NMR** (300 MHz, CDCl_3_) of **4b**, [*See procedure*](#P4b)

**^13^C NMR** (101 MHz, CDCl_3_) of **4****b**

**^19^F NMR** (376 MHz, CDCl_3_) of **4****b**

**^1^H NMR** (400 MHz, CDCl_3_) of **4c**, [*See procedure*](#P4c)

**^13^C NMR** (101 MHz, CDCl_3_) of **4****c**

**^19^F NMR** (376 MHz, CDCl_3_) of **4****c**

**^1^H NMR** (300 MHz, CDCl_3_) of **4d**, [*See procedure*](#P4d)

**^13^C NMR** (101 MHz, CDCl_3_) of **4****d**

**^19^F NMR** (282 MHz, CDCl_3_) of **4****d**

**^1^H NMR** (300 MHz, CDCl_3_) of **4e**, [*See procedure*](#P4e)

**^13^C NMR** (101 MHz, CDCl_3_) of **4****e**

**^19^F NMR** (282 MHz, CDCl_3_) of **4****e**

**^1^H NMR** (300 MHz, CDCl_3_) of **4f**, *[See procedure](#P4f)*

**^13^C NMR** (101 MHz, CDCl_3_) of **4****f**

**^19^F NMR** (282 MHz, CDCl_3_) of **4****f**

**Substrate scopes of biorelevant molecules**

**^1^H NMR** (300 MHz, CDCl_3_) of **5a**, *[See procedure](#P5a)*

**^13^C NMR** (101 MHz, CDCl_3_) of **5a**

**^19^F NMR** (282 MHz, CDCl_3_) of **5a**

**^1^H NMR** (300 MHz, CDCl_3_) of **5b**, *[See procedure](#P5b)*

**^13^C NMR** (101 MHz, CDCl_3_) of **5b**

**^19^F NMR** (282 MHz, CDCl_3_) of **5b**

**^1^H NMR** (400 MHz, CDCl_3_) of **5c**, *[See procedure](#P5c)*

**^13^C NMR** (101 MHz, CDCl_3_) of **5c**

**^19^F NMR** (282 MHz, CDCl_3_) of **5c**

**^1^H NMR** (300 MHz, CDCl_3_) of **5d**, *[See procedure](#P5d)*

**^13^C NMR** (101 MHz, CDCl_3_) of **5d**

**^19^F NMR** (282 MHz, CDCl_3_) of **5d**

**^1^H NMR** (300 MHz, CDCl_3_) of **5e**, *[See procedure](#P5e)*

**^13^C NMR** (101 MHz, CDCl_3_) of **5e**

**^19^F NMR** (282 MHz, CDCl_3_) of **5e**

**^1^H NMR** (300 MHz, CDCl_3_) of **5f**, *[See procedure](#P5f)*

**^13^C NMR** (101 MHz, CDCl_3_) of **5f**

**^19^F NMR** (282 MHz, CDCl_3_) of **5f**

**^1^H NMR** (300 MHz, CDCl_3_) of **5g**, *[See procedure](#P5g)*

**^13^C NMR** (101 MHz, CDCl_3_) of **5g**

**^19^F NMR** (282 MHz, CDCl_3_) of **5g**

**^1^H NMR** (300 MHz, CDCl_3_) of **6**, *[See procedure](#P6)*

**^13^C NMR** (101 MHz, CDCl_3_) of **6**

**^19^F NMR** (282 MHz, CDCl_3_) of **6**

**^1^H NMR** (300 MHz, DMSO-*d*_6_) of **7**, *[See procedure](#P7)*

**^13^C NMR** (101 MHz, DMSO-*d*_6_) of **7**

**^19^F NMR** (282 MHz, DMSO-*d*_6_) of **7**

**^1^H NMR** (300 MHz, DMSO-*d*_6_) of **PF-D1**, *[See procedure](#PPFD1)*

**^13^C NMR** (101 MHz, DMSO-*d*_6_) of **PF-D1**

**^19^F NMR** (282 MHz, DMSO-*d*_6_) of **PF-D1**

1. [↑](#footnote-ref-1)
2. [↑](#footnote-ref-2)
3. [↑](#footnote-ref-3)
