# Supplementary figures and images for: FluoroFusion: NHC-Catalyzed Nucleophilic Aromatic Substitution Reaction Unveils Functional Perfluorinated Diarylmethanones
Source: Org Lett. 2024 Mar 8;26(11):2338–42. doi: 10.1021/acs.orglett.4c00677 (PMC10964231; doi:10.1021/acs.orglett.4c00677)

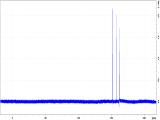

Supplement: Supplementary file 1 — ol4c00677_si_001.zip [file ol4c00677_si_001.zip › NHC SNAr OL/NMR/2c'/19F/pdata/1/thumb.png]

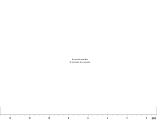

Supplement: Supplementary file 1 — ol4c00677_si_001.zip [file ol4c00677_si_001.zip › NHC SNAr OL/NMR/2c'/1H/pdata/1/thumb.png]

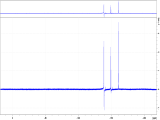

Supplement: Supplementary file 1 — ol4c00677_si_001.zip [file ol4c00677_si_001.zip › NHC SNAr OL/NMR/2j/19F/pdata/1/thumb.png]

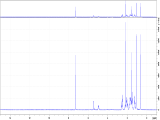

Supplement: Supplementary file 1 — ol4c00677_si_001.zip [file ol4c00677_si_001.zip › NHC SNAr OL/NMR/2j/1H/pdata/1/thumb.png]

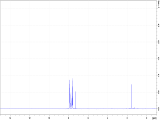

Supplement: Supplementary file 1 — ol4c00677_si_001.zip [file ol4c00677_si_001.zip › NHC SNAr OL/NMR/3a/1H/pdata/1/thumb.png]

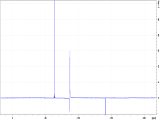

Supplement: Supplementary file 1 — ol4c00677_si_001.zip [file ol4c00677_si_001.zip › NHC SNAr OL/NMR/3b/19F/pdata/1/thumb.png]

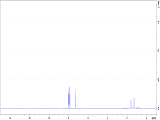

Supplement: Supplementary file 1 — ol4c00677_si_001.zip [file ol4c00677_si_001.zip › NHC SNAr OL/NMR/3b/1H/pdata/1/thumb.png]

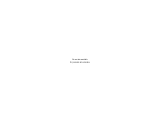

Supplement: Supplementary file 1 — ol4c00677_si_001.zip [file ol4c00677_si_001.zip › NHC SNAr OL/NMR/3c/19F/pdata/1/thumb.png]

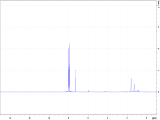

Supplement: Supplementary file 1 — ol4c00677_si_001.zip [file ol4c00677_si_001.zip › NHC SNAr OL/NMR/3c/1H/pdata/1/thumb.png]
