# Supplementary material for: FluoroFusion: NHC-Catalyzed Nucleophilic Aromatic Substitution Reaction Unveils Functional Perfluorinated Diarylmethanones
Source: Org Lett. 2024 Mar 8;26(11):2338–42. doi: 10.1021/acs.orglett.4c00677 (PMC10964231; doi:10.1021/acs.orglett.4c00677)

Supplementary Information

FluoroFusion: NHC-Catalyzed Nucleophilic Aromatic Substitution Reaction Unveils Functional Perfluorinated Diarylmethanones

Cheng-Lin Chan[1,2], Shao-Chi Lee[3,4], Pei-Shan Lin[1], Radyn Vanessa Phaz P. Tapales[1,2], Jia-Syuan Li[1], Chun-An Lai[1], Jyh-Tsung Lee[1], Chien-Hung Li[5] and Hsuan-Hung Liao*[,1,5]

[1]*Department of Chemistry, National Sun Yat-sen University, Kaohsiung, Taiwan (R.O.C.)*

[2]*Department of International PhD Program for Science, National Sun Yat-sen University, Kaohsiung, Taiwan (R.O.C.)*

[3]*Department of Chemistry, National Chung Hsing University, Taichung, Taiwan (R.O.C)*

[4]*KAUST Catalysis Center (KCC), King Abdullah University of Science and Technology (KAUST), Thuwal, Saudi Arabia*

[5]*Department of Applied and Medicinal Chemistry, Kaohsiung Medical University, Kaohsiung, Taiwan (R.O.C.)*

**Corresponding author. Email: hsuan-hung.liao@mail.nsysu.edu.tw*

Back to contents

Contents

1. General information 2
1.1 Pre-treatment of experiments, solvents, and reagents 2
1.2 Chromatography, data analysis, and collection 2
1.3 Instrument catalogue 2
1.4 Purchasing reagent 3
1.5 Synthesized compound 10
2. Experimental procedure 13
2.1. Preparation of substrates 13
2.2. Preparation of *N*-heterocyclic carbene (NHCs) 15
2.3. Standard condition of NHC-catalyzed $S_{N}Ar$ reaction 17
2.4 General procedures of NHC-Catalyzed $S_{N}Ar$ reaction 19
General procedure A 19
General procedure B 19
2.5 Substrate scopes overview of NHC-Catalyzed $S_{N}Ar$ reaction 20
2.6 Substrate scopes data of NHC-Catalyzed $S_{N}Ar$ reaction 23
2.7 Large-scale syntheses of NHC-Catalyzed $S_{N}Ar$ reaction 61
3. Synthesis of bioisostere 62
3.1 Procedure of synthesis 62
3.2 Procedure comparison 65
4. Experimental procedure of LIBs test 66
4.1 Preparation of LNiMnO cathode material pole piece and electrolyte 66
4.2 Cycle Life Test 67
5. Proposed mechanism 67
6. Reference 68
7. NMR spectra 69

1. General information

1.1 Pre-treatment of experiments, solvents, and reagents

Glassware All glassware has been oven-dried after cleaning with a cleaning machine, Steelco LAB 500CL.

Solvents All solvents were purchased from suppliers. Unless otherwise stated, the following ACS grade solvents (Acetonitrile, 1,4-Dioxane, Diethyl Ether, DCM, DMF, THF, Toluene) were stored over microwave-activated 3Å molecular sieves for at least one night and transferred into anhydrous engineering alumina column drying system (Vigor Gas Purification Technologies Co., Ltf, VSPS-7) before use.

Chemicals To maintain the activity, sensitive compounds such as NHC catalysts and aldehyde were stored in the glovebox. Other chemicals were directly used as received and were always filled with nitrogen before storage and twined with parafilm carefully. See section **S1.4** for the complete supplier list of each chemical.

1.2 Chromatography, data analysis, and collection

Thin-layer Chromatography (TLC) Merck aluminium-backed sheets coated with $60F_{254}$ silica gel. The silica plate was visualized using a UV lamp (λ_{max} = 254 nm).

Column chromatography Column chromatography was carried out using KM3 scientific silica gel (45 – 75 μm) purchased from KM3 scientific.

1.3 Instrument catalogue

Nuclear Magnetic Resonance (NMR) ^{1}H-, ^{13}C- and ^{19}F- Nuclear Magnetic Resonance (NMR) spectra were used to identify the structure of starting materials and products by using Bruker Avance 300 MHz, Jeol ECZS 400 MHz, Bruker Avance 500 MHz and Jeol ECZR 600 MHz. Coupling constants are abridged as follows: s = singlet, d = doublet, t = triplet, q = quartet, quin = quintet, sext = sextet, sept = septet, m = multiplet, dd = doublet of doublet.

Gas Chromatography–Mass Spectrometry (GC-MS) The operation method was set as follows: 1.0 mL sample was injected by auto-sampler in a split mode (100:1) with 0.5 mL air gap into the GC-MS system consisting of an Agilent 8860 gas chromatograph, an Agilent 5977B mass selective detector, and Agilent 7693A autoinjector. Gas chromatography was performed on a 30 m HP-5MS with 0.25 mm inner diameter (I.D.) and 0.25 mm film thickness with an initial injection temperature of 50 ℃ to 300 ℃, MSD transfer line of 280 ℃, and the ion source adjusted to 230 ℃. The helium carrier gas was set at a constant flow rate of 1.197 ml min-1. The mass spectrometer was operated in positive electron impact mode (EI), with ionization energy in the m/z 50 – 550 scan range. The spectra of all chromatogram peaks were evaluated using the MSD Chemstation.

High-Resolution Mass Spectra (HRMS) Jeol AccuTOF GCx-plus / Shimadzu QP2020

Table S1.1 Supplier of equipment and instruments.

Equipment	Supplier
Electronic balance	*Shimadzu* UW2200H/ ATX224
Hot plate stirrer	*Corning* PC-420D
Immersion cooler	*Panchum* IC-9090
Pump of rotary evaporator	*KNF Laboport* N820.3FT.18
Rotary evaporator	*Heidolph* Hei-Vap Core HL G3
Refrigerated circulator bath	*Panchum* CC-300
Ultra-low temp. reaction bath	*Panchum* UR-8500
Vacuum pump	*Edwards* RV5
Instruments	Supplier
Glovebox	*Vigor* SG1200/750TS-F
GC-MS (Gas Chromatograph Mass Spectrometer)	*Agilent* 5977B

1.4 Purchasing reagent

Table S1.2 Supplier of solvents.

Solvent	Supplier	Solvent	Supplier
ACS Acetone	Duksan	HPLC Acetonitrile	J.T. Baker
ACS Acetonitrile	J.T. Baker	HPLC DMF	Macron
ACS Benzene	Echo	HPLC Ethyl acetate	Merck
ACS Chloroform	Acros	HPLC Hexane	Echo
ACS Dimethyl sulfoxide	UR	HPLC Isopropanol	Echo
ACS Diethyl ether	Duksan	ACS Hexane	Duksan
ACS Dichloromethane	Macron /Duksan	ACS Methanol	Macron
ACS Ethanol	J.T. Baker	ACS THF	Macron
ACS Ethyl acetate	Macron	ACS Toluene	Echo

Back to contents

Table S.1.3 Supplier of solvents in solvent purification systems.

Solvent	Supplier	Solvent	Supplier
Acetonitrile	J.T. Baker	Dichloromethane	Macron
DMF	Macron	THF	Macron
1,4-Dioxane	J.T. Baker	Toluene	J.T. Baker
Diethyl ether	Echo		

Table S1.4 Supplier of deuterated solvents.

Solvent	Supplier	Solvent	Supplier
Acetonitrile-d_3	Sigma-Aldrich	DMSO-d_6	Sigma-Aldrich
Benzene-d_6	Sigma-Aldrich	Dichloromethane-d_2	Sigma-Aldrich
Chloroform-d_1	Merck	Methanol-d_4	Sigma-Aldrich

Table S1.5 Supplier of commercially available *N*-heterocyclic carbenes (NHCs).

Structure	Name	CAS#	Supplier
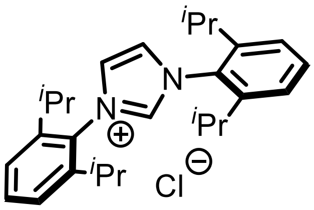	1,3-Bis(2,6-diisopropylphenyl)-1*H*-imidazol-3-ium chloride	250285-32-6	BLD
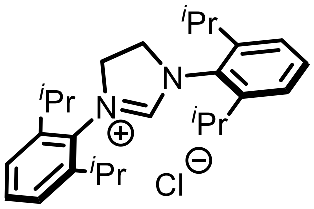	1,3-Bis(2,6-diisopropylphenyl)-4,5-dihydro-1*H*-imidazol-3-ium chloride	258278-25-0	BLD
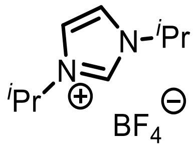	1,3-Diisopropyl-1*H*-imidazol-3-ium tetrafluoroborate	286014-34-4	BLD
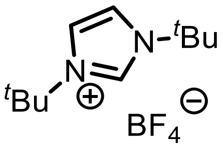	1,3-Di-tert-butyl-1*H*-imidazol-3-ium tetrafluoroborate	263163-17-3	BLD

Back to contents

Table S1.6 Supplier of commercially available aldehydes.

No.	Structure	Name	CAS Number	Supplier
**1a**	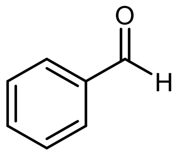	Benzaldehyde	100-52-7	Nova
**1b**	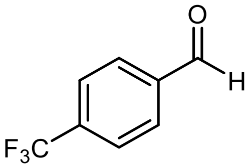	4-(Trifluoromethyl)benzaldehyde	455-19-6	Combi-Blocks
**1c**	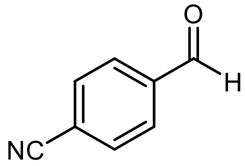	4-Formylbenzonitrile	105-07-7	Merck
**1e**	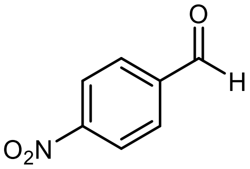	4-Nitrobenzaldehyde	555-16-8	BLD Pharm
**1f**	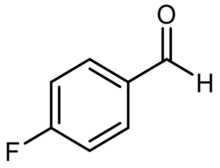	4-Fluorobenzaldehyde	459-57-4	Aldrich
**1g**	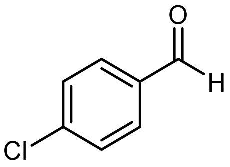	4-Chlorobenzaldehyde	104-88-1	Acros
**1h**	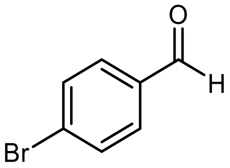	4-Bromobenzaldehyde	1122-91-4	vetec
**1i**	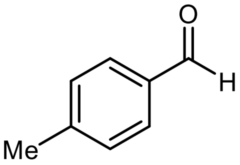	4-Methylbenzaldehyde	104-87-0	BLD Pharm
**1l**	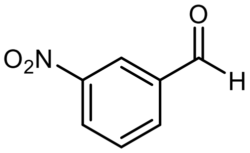	3-Nitrobenzaldehyde	99-61-6	Acros

Back to contents

**1m**	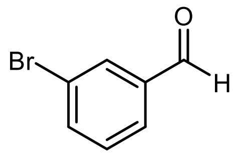	3-Bromobenzaldehyde	3132-99-8	Nova
**1o**	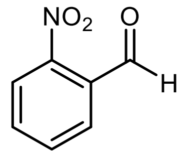	2-Nitrobenzaldehyde	552-89-6	Alfa Aesar
**1p**	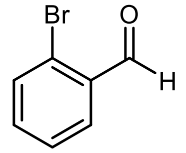	2-Bromobenzaldehyde	6630-33-7	Alfa Aesar
**1q**	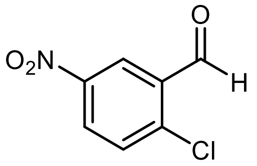	2-Chloro-5-nitrobenzaldehyde	6361-21-3	Alfa Aesar
**1r**	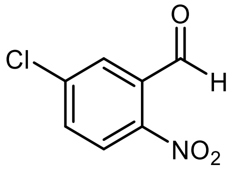	5-Chloro-2-nitrobenzaldehyde	6628-86-0	BLD Pharm
**1s**	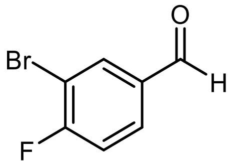	3-Bromo-4-fluorobenzaldehyde	77771-02-9	BLD Pharm
**1t**	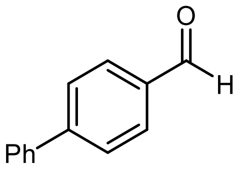	[1,1'-Biphenyl]-4-carbaldehyde	3218-36-8	BLD Pharm
**1v**	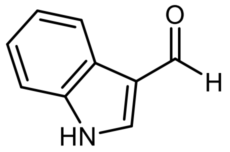	1*H*-Indole-3-carbaldehyde	487-89-8	Alfa Aesar
**1x**	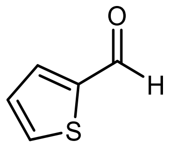	Thiophene-2-carbaldehyde	98-03-3	Alfa Aesar
**1y**	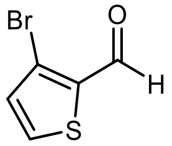	3-Bromothiophene-2-carbaldehyde	930-96-1	BLD Pharm
**1a’**	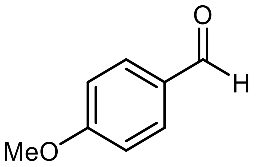	4-Methoxybenzaldehyde	123-11-5	Alfa Aesar

Back to contents

**1b’**	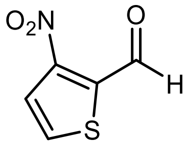	3-Nitrothiophene-2-carbaldehyde	58963-75-0	BLD Pharm
**1c’**	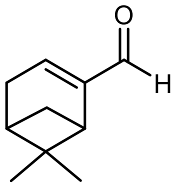	6,6-Dimethylbicyclo[3.1.1]hept-2-ene-2-carbaldehyde	564-94-3	BLD Pharm
**1d’**	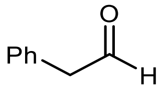	2-Phenylacetaldehyde	122-78-1	Alfa Aesar

Back to contents

Table S1.7 Supplier of commercially available polyfluoroarenes.

No.	Structure	Name	CAS Number	Supplier
**2a**	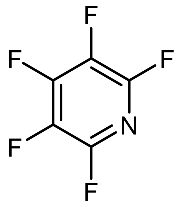	Pentafluoropyridine	700-16-3	BLD Pharm
**2b**	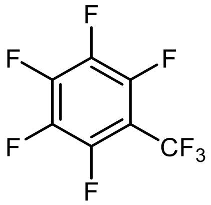	1,2,3,4,5-Pentafluoro-6-(trifluoromethyl)benzene	434-64-0	BLD Pharm
**2c**	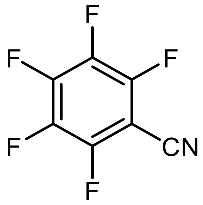	Pentafluorobenzonitrile	773-82-0	BLD Pharm
**2d**	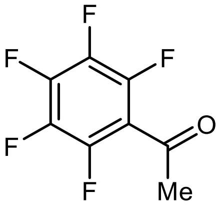	2,3,4,5,6-Pentafluoroacetophenone	652-29-9	BLD Pharm
**2e**	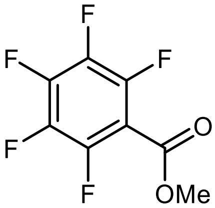	Methyl-pentafluorobenzoate	36629-42-2	TCI
**2f**	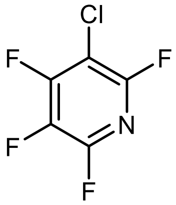	3-Chloro-2,4,5,6-tetrafluoropyridine	1735-84-8	BLD Pharm
**2a'**	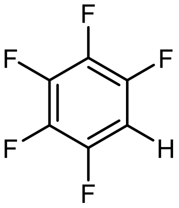	1,2,3,4,5-Pentafluorobenzene	363-72-4	BLD Pharm
**2b'**	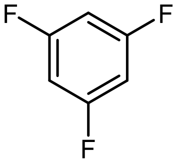	1,3,5-Trifluorobenzene	372-38-3	BLD Pharm

Back to contents

Table S1.8 Supplier of commercially available reagent.

Name	CAS Number	Supplier
Bis(triphenylphosphine)-palladium (II) dichloride	13965-03-2	Nova
Bis(pinacol)diborane	73183-34-3	Matrix Scientific
Caesium carbonate	534-17-8	Alfa Aesar
Caesium fluoride	13400-13-0	Nova
4-(4-Chloroquinazolin-7-yl) morpholine	1334602-74-2	BLD Pharm
4-Dimethylaminopyridine	1122-58-3	Merck
Methyl iodide	74-88-4	Jassen
1-Methyl-1*H*-imidazole	616-47-7	ACROS
N, *N*'-Dicyclohexylcarbodiimide	538-75-0	Merck
Palladium (II) chloride	7647-10-1	TCI
Potassium acetate	127-08-2	Sigma-Aldrich
Potassium carbonate	584-08-7	Duksan
Sodium borohydride	16940-66-2	TCI
Sodium carbonate	497-19-8	Shimakyus
Tricyclohexylphosphine	2622-14-2	Nova
Triethylamine	121-44-8	Alfa Aesar

Back to contents

1.5 Synthesized compound

Table S1.9 Synthesized aryl aldehydes and polyfluoroarenes.

No.	Structure	Name	Reference
**1d**	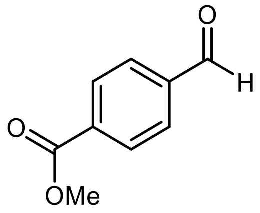	Methyl-4-formylbenzoate	Ref
**1j**	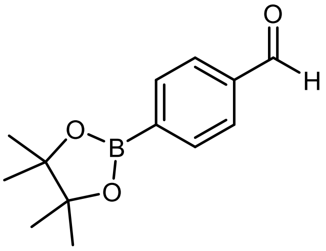	4-(4,4,5,5-Tetramethyl-1,3,2-dioxaborolan-2-yl)-benzaldehyde	Ref
**1k**	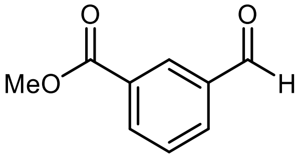	Methyl-3-formylbenzoate	Ref
**1n**	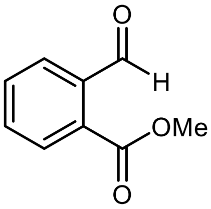	Methyl-2-formylbenzoate	Ref
**1u**	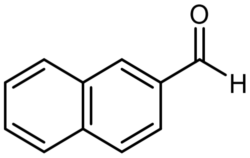	2-Naphthaldehyde	Ref
**1w**	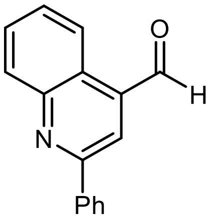	2-Phenylquinoline-4-carbaldehyde	Ref
**2g**	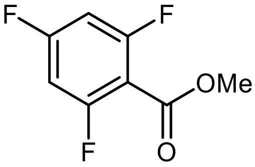	Methyl-2,4,6-trifluorobenzoate	Ref

**2c'**	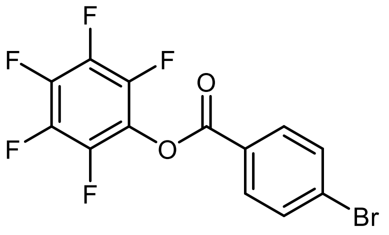	4-Bromophenyl 2,3,4,5,6-pentafluorobenzoate	link

Table S1.10 Synthesized bioactive analogues.

No.	Structure	Reference
**1z**	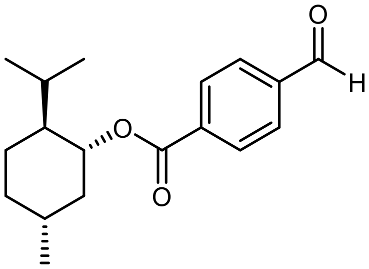 from *menthol*	Ref
**1aa**	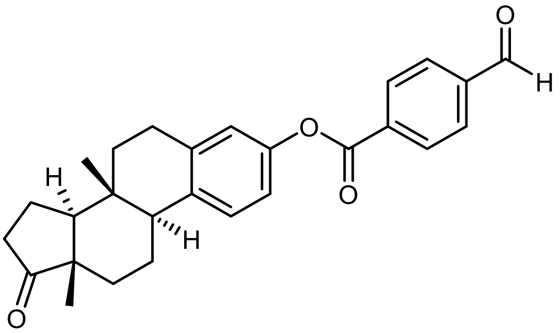 from *estrone*	Ref
**1ab**	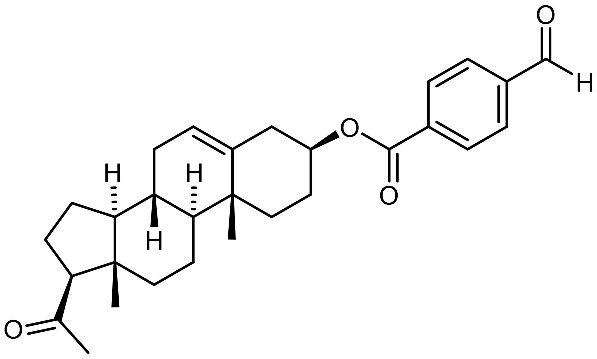 from *pregnenolone*	Ref

[Back to contents]

**1ac**	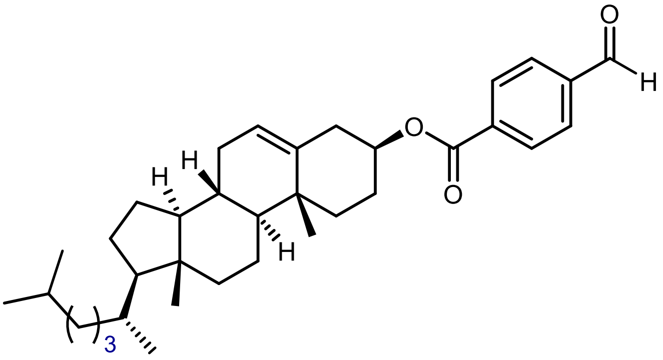 from *cholesterol*	Ref
**2h**	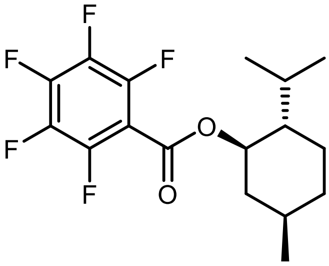 from *menthol*	Ref
**2i**	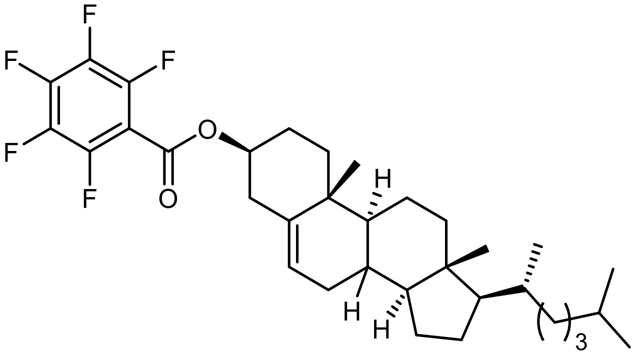 from *cholesterol*	Ref
**2j**	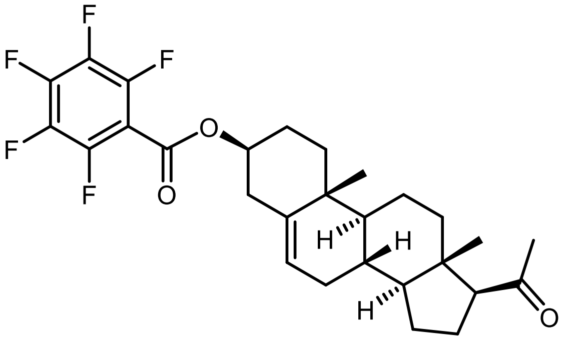 from *pregnenolone*	link

2. Experimental procedure

2.1. Preparation of substrates

Methylation

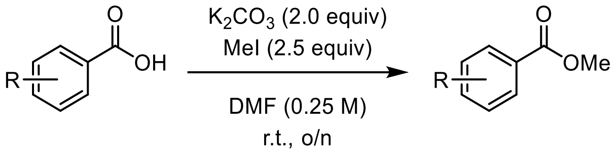

To a solution of benzoic acid (6.0 mmol, 1.0 equiv) in anhydrous DMF (24 mL, 0.25 M) was added finely ground potassium carbonate (1.7 g, 12 mmol, 2.0 equiv) and iodoethane (0.9 mL, 15 mmol, 2.5 equiv) under nitrogen atmosphere. The reaction stirred at rt until completion was indicated by TLC before being diluted with water and extracted with anhydrous ether. The combined organic layers were then washed with brine, dried over $MgSO_4$, filtered, and dried under high vacuum. The crude residue was purified by flash column chromatography (height of packing silica gel: 7.0 cm, Hexane/ethyl acetate: 6/1) to afford desire product.
All characterization data for each compound **1d**, **1k**, **1n** and **2g** are consistent with reported literatures.

Boronic esterification

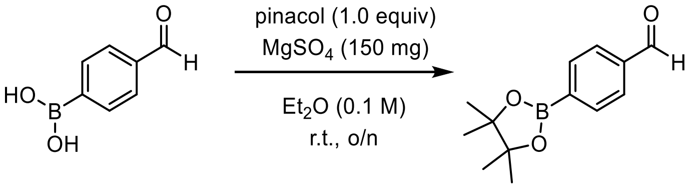

To the solution of 4-Formylbenzeneboronic acid (1.5 g, 10.0 mmol) in anhydrous ether (100 mL) was added pinacol (1.2 g, 10.0 mmol) and $MgSO_4$ (150 mg) under nitrogen atmosphere. The mixture was stirred for overnight at rt. After monitoring by TLC, the $MgSO_4$ was filtered. The solution was washed with water and dried over $MgSO_4$. The crude residue was purified by flash column chromatography (height of packing silica gel: 7.0 cm, Hexane/ethyl acetate: 6/1) to afford desire product.
The characterization data for compound **1j** is consistent with reported literature.

Reduction and oxidation

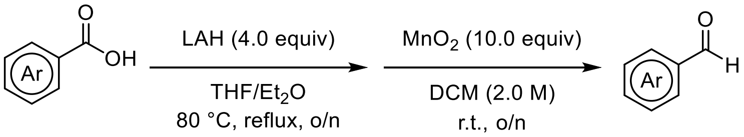

To the solution of lithium aluminium hydride (759.0 mg, 20.0 mmol, 4.0 equiv) in anhydrous ether (10.0 mL) was added aryl carboxylic acid solution (5.0 mmol in 10.0 mL of anhydrous THF) under nitrogen atmosphere. The reaction mixture was stirred at 80 ℃ for overnight. The reaction was cooled to 0 ℃ and quenched with water. The mixture was extracted

with 1.0 M $HCl_{(aq)}$. The organic layer was dried over $MgSO_4$ and evaporated to afford desire alcohol. The residue was further dissolved in anhydrous DCM. The solution was added to a flask which is equipped with MnO_2 (4.3 g, 50 mmol, 10.0 equiv). The reaction was stirred for overnight at room temperature. After monitoring by TLC, the reaction was filtered, extracted with DCM, and purified by column chromatography to afford desire product.
The characterization data for each compound **1u**, **1w** is consistent with reported literature.

Steglich esterification

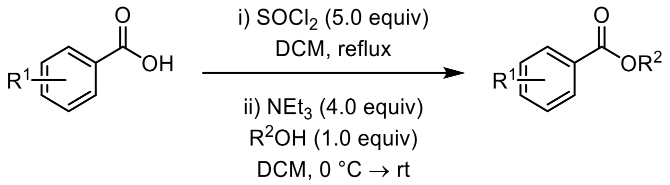

Thionyl chloride (1.8 mL, 25 mmol, 5.0 equiv) was added to the solution of benzoic acid (5.0 mmol, 1.0 equiv) in anhydrous DCM (20 mL) under nitrogen atmosphere. The reaction mixture was stirred under reflux for 5 h. After completion of the reaction, the excess of thionyl chloride and DCM were removed by high vacuum to give crude acyl chloride. Subsequently, the crude acyl chloride was resolved in 10 mL anhydrous DCM and was slowly added to the solution of triethylamine (2.0 mL, 15.0 mmol, 3.0 equiv) and R^2OH (5.0 mmol, 1.0 equiv) with 10 mL anhydrous DCM at 0 ℃ under nitrogen atmosphere. Then the reaction mixture was stirred for 2 - 5 h at room temperature. After monitoring by TLC, the reaction was quenched with water, extracted with DCM and water. The organic layer was dried over anhydrous $MgSO_4$ and evaporated in high vacuum. The residue was purified by column chromatography to provide the desired product.
The characterization data for each compound **1z**, **1aa**, **1ab**, **1ac**, **2h**, and **2i** is consistent with reported literature.

Perfluorophenyl 4-bromobenzoate, **2c'**

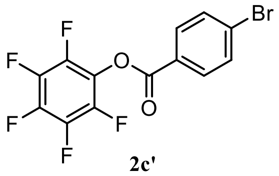

2c'

^{1}H NMR (300 MHz, $CDCl_3$) δ 8.06 (d, J = 8.7 Hz, 1H), 7.70 (d, J = 8.7 Hz, 1H) ppm.

^{13}C NMR (101 MHz, $CDCl_3$) δ 162.0, 142.6 – 140.0 (m), 141.0 – 139.1 (m), 138.6 – 136.7 (m), 132.4, 132.1, 130.3, 125.8. ppm.

^{19}F NMR (282 MHz, $CDCl_3$) δ -151.82 – -153.38 (m), -157.54 (t, J = 21.6 Hz), -162.09 (dd, J = 21.8, 17.2 Hz) ppm.

See NMR spectra

Back to contents

(8*S*,9*S*,10*R*,13*S*,14*S*,17*S*)-17-Acetyl-10,13-dimethyl-2,3,4,7,8,9,10,11,12,13,14,15,16,17-tetradecahydro-1H-cyclopenta[*a*]phenanthren-3-yl 2,3,4,5,6-pentafluorobenzoate, **2j**

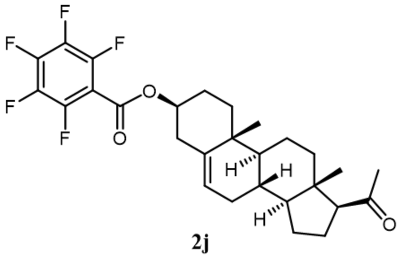

2j

^{1}H NMR (300 MHz, $CDCl_3$) δ 5.45 – 5.43 (m, 1H), 4.97 – 4.86 (m, 1H), 2.57 – 2.45 (m, 3H), 2.20 – 1.90 (m, 8H), 1.80 – 1.41(m, 8H), 1.25 – 1.17 (m, 3H), 1.05 (s, 4H), 0.64 (s, 3H) ppm.

^{13}C NMR (101 MHz, $CDCl_3$) δ 209.3, 158.2, 146.5 – 143.7 (m), 144.3 – 141.4 (m), 138.9, 138.8 – 136.1 (m), 122.9, 108.9 – 108.6 (m), 63.5, 56.7, 49.8, 43.8, 38.7, 37.7, 36.8, 36.5, 31.69, 31.67, 31.4, 27.5, 24.4, 22.7, 20.9, 19.1, 13.1. ppm.

^{19}F NMR (282 MHz, $CDCl_3$) δ -138.6 – -138.8 (m), -149.2 (tt), -160.4 – -160.6 (m) ppm.

See NMR spectra

2.2. Preparation of *N*-heterocyclic carbene (NHCs)

1,3-Dimethyl-1H-imidazol-3-ium iodide, NHC1

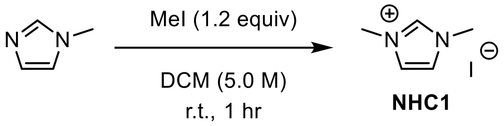

According to a literature procedure[1], an oven-dried flask containing a stirring bar was cooled to room temperature under high vacuum and refilled with nitrogen. Methyl iodide (851.6 mg, 6.0 mmol, 1.2 equiv) was added in portions to a DCM (1.0 mL) solution of the 1-methyl-1*H*-imidazole (410 mg, 5.0 mmol, 1.0 equiv) over the course of 30 min (**Caution**: exothermic reaction) at 0 °C. The reaction mixture was stirred for further 1 hr, after which the mixture was concentrated and dried under high vacuum to afford carbene **NHC1** (1030.4 mg, 4.6 mmol, 92%) as a white solid.

^{1}H NMR (300 MHz, MeOD) δ 9.24 (s, 1H), 7.87 (s, 2H), 4.20 (s, 6H) ppm.

^{13}C NMR (101 MHz, $CDCl_3$) δ 137.7, 123.4, 37.1 ppm.

See NMR spectra

Back to contents

3-(2,6-Diisopropylphenyl)-5,6,7,8-tetrahydro-4*H*-cyclohepta[*d*]thiazol-3-ium perchlorate, NHC4

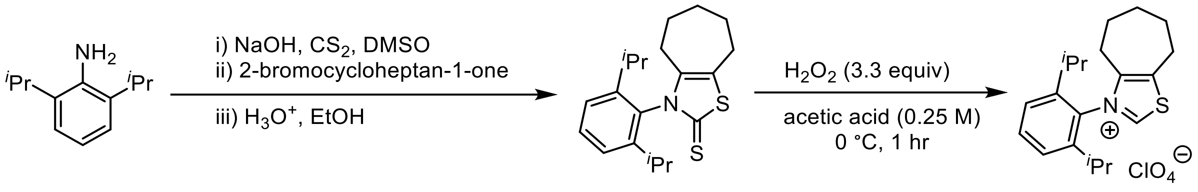

According to the literature[2], NaOH (0.4 g, 11 mmol) was dissolved to a solution of 2,6-diisopropylamine (1.5 mL, 11 mmol) in DMSO (5 mL). CS_2 was added dropwise at 0 ℃ and the media was allowed to stir at room temperature for 1 hr. 2-Bromocycloheptan-1-one was then added by portion, and the solution was stirred overnight at room temperature. The precipitate formed by the addition of water (10 mL) was filtrated, washed with water, and then suspended in absolute ethanol (13 mL). Concentrated HCl (0.5 mL) was then added, and the solution was heated to reflux for 1 hr. After cooling and the addition of water (10 mL), a brown solid was filtrated. The dithiocarbamate was obtained as a beige solid, which was then dissolved in acetic acid (25 mL) and treated with 30% H_2O_2 (1.6 mL, 19.1 mmol) for 1 hr at room temperature. Remove most of the solvents, and the residue was dissolved in methanol (5 mL). To this solution, a solution of $NaClO_4$ (3.0 g, MeOH 140 mL, and water 70 mL) was added at 0 ℃ and stirred for 1 hr at the same temperature. After evaporation of solvents and trituration with Et_2O, the product **SN_4** was obtained as a beige solid (1.7 g, 4.1 mmol, 40%).

An oven-dried flask containing a stirring bar was cooled to room temperature under high vacuum and refilled with nitrogen. A solution of the **SN_4** (2.2 g, 6.5 mmol, 1.0 equiv) in glacial acetic acid (27 mL, 0.25 M) was treated under a water bath cooling dropwise with H_2O_2 (30%, 2.0 mL, 2.1 mmol, 3.3 equiv) and was stirred for 1 hr at that temperature. After 1 hr, the volatiles were removed, the residue was dissolved in MeOH (5.0 mL), and a solution of sodium perchlorate monohydrate (3.7 g, 26.6 mmol, 4.1 eq) in MeOH/H2O = 2:1 (23.0 mL) was added at room temperature. After stirring for 10 min, the volume of the solvent was reduced to half. The solid was collected by suction filtration and was washed with H_2O (40.0 mL). The following recrystallization with MeOH afforded the product **NHC4** as a colorless solid.

^{1}H NMR (400 MHz, $CDCl_3$) δ 9.7 (s, 1H), 7.61 (t, J = 7.8 Hz, 1H), 7.37 (d, J = 7.8 Hz, 2H), 3.19 − 3.15 (m, 2H), 2.57 − 2.53 (m, 2H), 2.06 (sept, J = 6.9 Hz, 2H), 1.97 − 1.91 (m, 4H), 1.19 (d, J = 6.8 Hz, 6H), 1.17 (d, J = 6.8 Hz, 6H) ppm.

^{13}C NMR (400 MHz, $CDCl_3$) δ 155.7, 148.5, 144.9, 141.0, 141.0, 132.5, 132.1, 125.2, 30.8, 28.8, 28.2, 27.3, 26.6, 25.5, 24.8, 23.2 ppm.

See NMR spectra

2.3. Standard condition of NHC-catalyzed S_NAr reaction

Prepare two over-dried tubes (5.0 mL) with a screw cap containing a stirring bar that was charged into the nitrogen-filled glovebox. A tube was added **1a** (10.6 mg, 0.1 mmol, 1.0 equiv), **2a** (16.9 mg, 0.1 mmol, 1.0 equiv), and anhydrous solvent (1.0 mL, 0.1 M). The solution was added into the other tube, which contained an **NHC** catalyst (0.02 mmol, 20 mol%) and base (0.15 mmol, 1.5 equiv). The sealed tube was taken out of the glove box and stirred for 18 hr at room temperature. After the reaction was completed, the crude was filtered by flash column chromatography with ethyl acetate (the height of packing silica gel: 3.0 – 4.0 cm). After removing the solvent by suction filtration, 14.6 mg trifluoromethylbenzene was added to configure the NMR sample to calculate the spectrum yield.

Table S2.1 Screening of NHC-catalyst

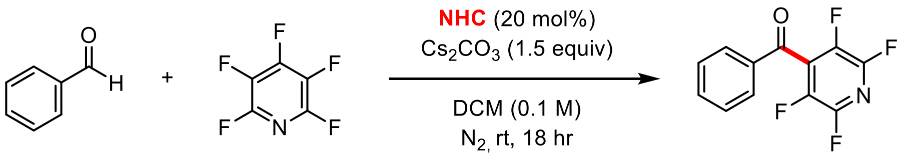

Entry	NHC	Yield of **4a**(%)[a]	Entry	NHC	Yield of **3a**(%)
1	**NHC1**	95(92)[b]	4	**NHC4**	-
2	**NHC2**	73	5	**NHC5**	-
3	**NHC3**	54	6	**NHC6**	trace

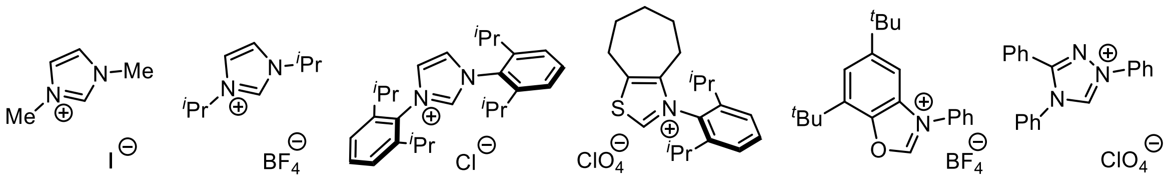

[a]The spectrum yield was determined by ^{19}F NMR using trifluoromethylbenzene as an internal standard. [b]Isolated yield.

Table S2.2 Screening of solvent

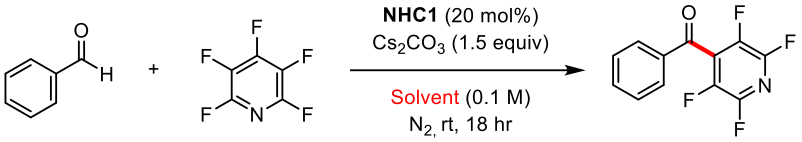

1a 2a 3a

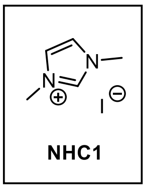

Entry	Solvent	Yield of **3a**(%)[a]
1	DCM	95(92)[b]
2	THF	65
3	ACN	32
4	DMSO	N.D.
5	Toluene	15

[a]The spectrum yield was determined by ^{19}F NMR using trifluoromethylbenzene as an internal standard. [b]Isolated yield.

Table S2.3 Screening of base

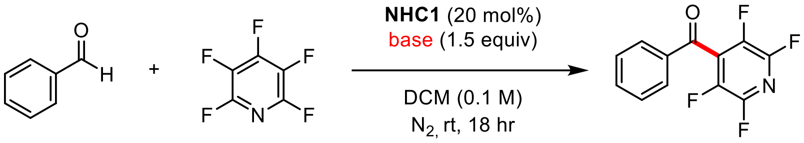

1a 2a 3a

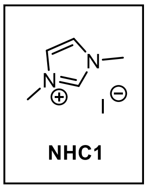

Entry	base	Yield of **3a**(%)[a]
1	Cs_2CO_3	95(92)[b]
2	K_2CO_3	N.D.
3	CsOAc	N.D.
4	Et_3N	N.D.

[a]The spectrum yield was determined by ^{19}F NMR using trifluoromethylbenzene as an internal standard. [b]Isolated yield.

Table S2.4 Background condition test

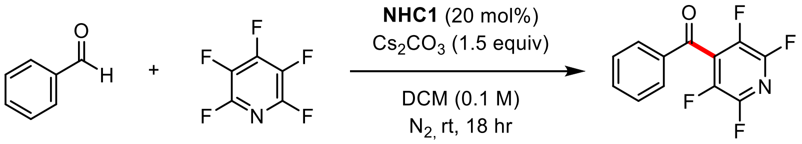

1a 2a 3a

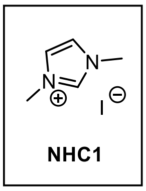

Entry	Variation from optimized condition	Yield of **3a**(%)[a]
1	None	95(92)[b]
2	Without NHC catalyst	-
3	Without base	-
4	Air condition	38

[a]The spectrum yield was determined by ^{19}F NMR using trifluoromethylbenzene as an internal standard. [b]Isolated yield.

Back to contents

2.4 General procedures of NHC-Catalyzed S_NAr reaction

General procedure A

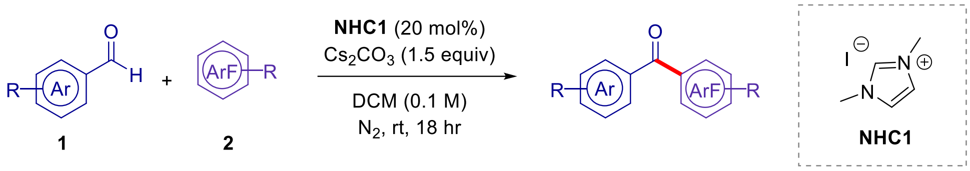

Prepare two over-dried tubes (5.0 mL) with a screw cap containing a stirring bar that was charged into the nitrogen-filled glovebox. A tube was added aromatic aldehyde (0.1 mmol, 1.0 equiv), perfluoroarene (0.1 mmol, 1.0 equiv), and anhydrous DCM (1.0 mL, 0.1 M). The DCM solution was added into the other tube, which contained **NHC1** precatalyst (4.5 mg, 0.02 mmol, 20 mol%) and Cs_2CO_3 (48.8 mg, 0.15 mmol, 1.5 equiv). The sealed tube was taken out of the glovebox and stirred for a further 18 hr. After the reaction was completed, the crude was purified by silica gel column chromatography (the height of packing silica gel: 6.0 – 8.0 cm, appropriate eluent) or preparative TLC on silica gel to afford the products.

General procedure B

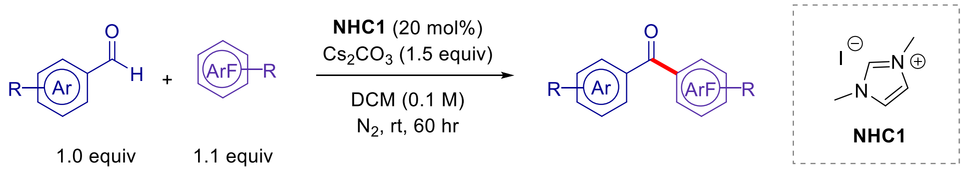

In the nitrogen-filled glovebox, an oven-dried pear-shaped flask (10.0 mL) with a septum and 250 ml reaction round bottom bottle equipped with a stir bar was prepared. Aryl aldehyde (7.0 mmol, 1.0 equiv) and perfluoroarene (7.7 mmol, 1.1 equiv) were added into the pear-shaped flask followed by septum covering. The round bottom bottle was sealed by septum after the addition of **NHC1** catalyst (313.6 mg, 1.4 mmol, 20 mol%) and Cs_2CO_3 (3423.0 mg, 10.5 mmol, 1.5 equiv). Both containers were taken out of the glovebox. Under nitrogen gas, 50 mL anhydrous DCM was added into the reaction round bottom bottle as well as 5 mL into the heart-shaped bottle. The mixture of **1** and **2** in DCM was injected into the reaction round bottom bottle. Wash the residue three times (5 mL DCM per time). The reaction was stirred for a further 60 hr. After the reaction is completed, wash the reaction with DCM and water, dry it with $MgSO_4$ then remove DCM. Purified by silica gel column chromatography (the height of packing silica gel: 8.0 – 10.0 cm) followed by removing the solvent in a vacuum to afford the products.

2.5 Substrate scopes overview of NHC-Catalyzed S_NAr reaction

Table S2.5 Substrate scopes of compound 3

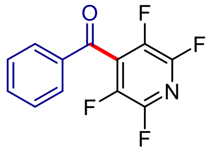 **3a**	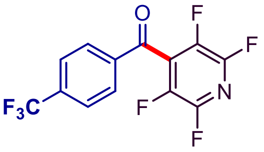 **3b**	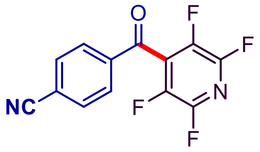 **3c**	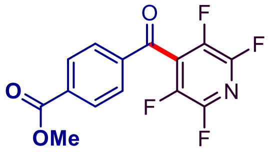 **3d**
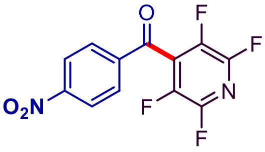 **3e**	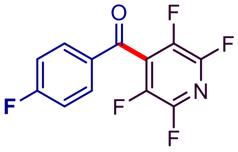 **3f**	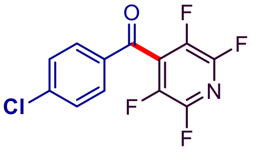 **3g**	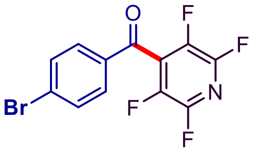 **3h**
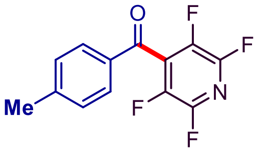 **3i**	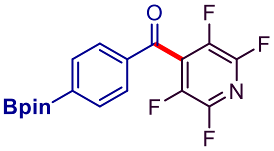 **3j**	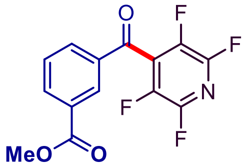 **3k**	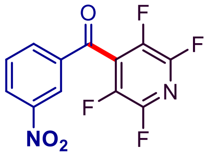 **3l**
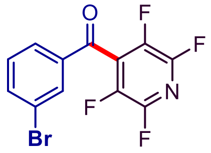 **3m**	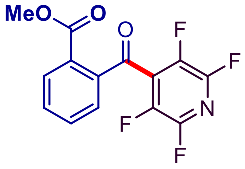 **3n**	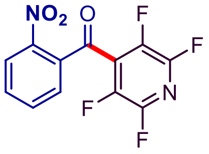 **3o**	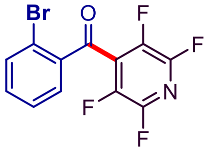 **3p**
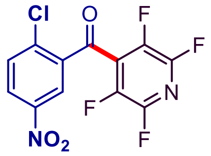 **3q**	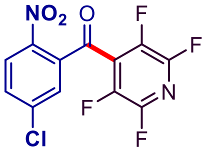 **3r**	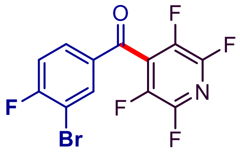 **3s**	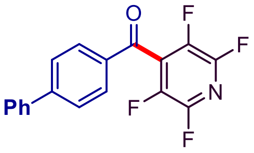 **3t**
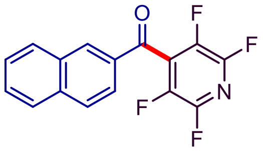 **3u**			
**Heteroaryl aldehyde**			
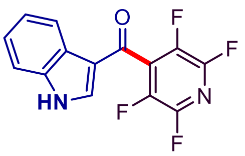 **3v**	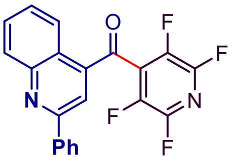 **3w**	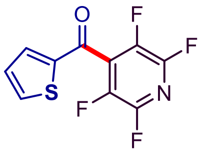 **3x**	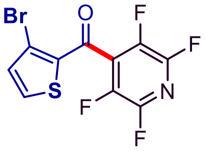 **3y**

Back to contents

Table S2.6 Substrate scopes of compound 4

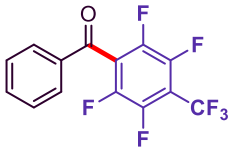 **4a**	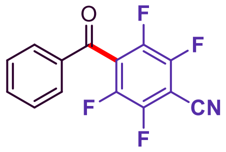 **4b**	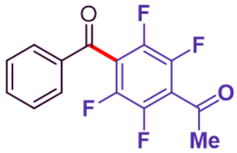 **4c**	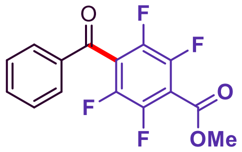 **4d**
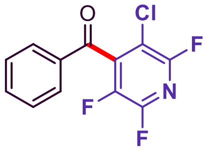 **4e**	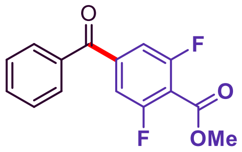 **4f**		

Table S2.7 Biorelevant molecules syntheses of compound 7

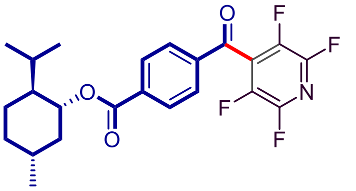 **5a**	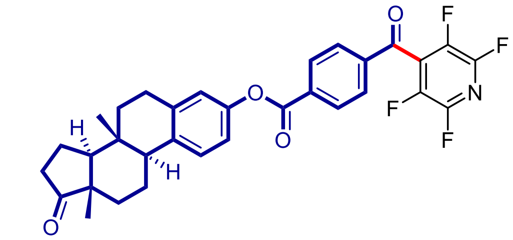 **5b**
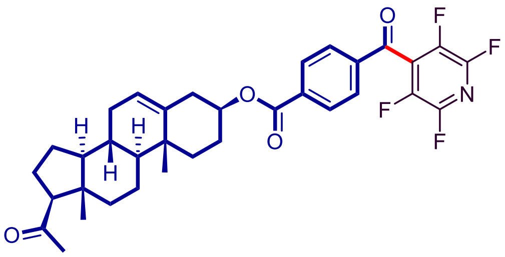 **5c**	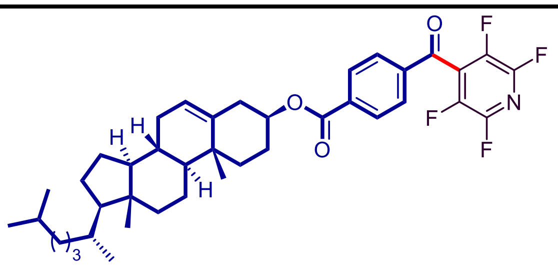 **5d**
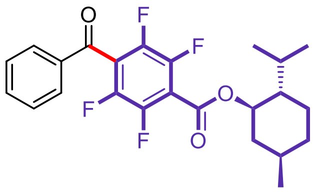 **5e**	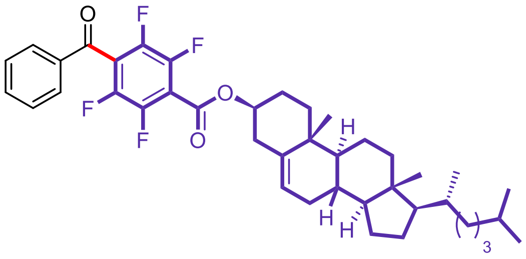 **5f**
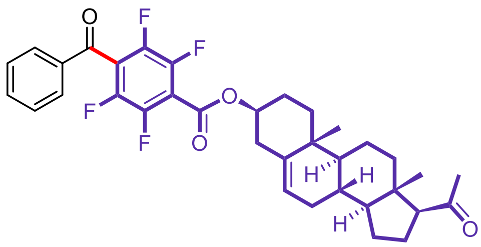 **5g**	

Back to contents

Table S2.8 Substrate scopes limitations

Started material	Result	Started material	Result
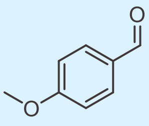 **1a'**	No Reaction (electron donating group)	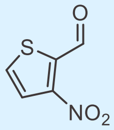 **1b'**	No reaction (low solubility)
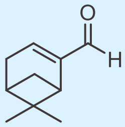 **1c'**	Trace (low conversion)	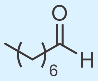 **1d'**	Messy reaction (aldol condensation side reaction)
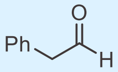 **1e'**	Messy reaction (aldol condensation side reaction)	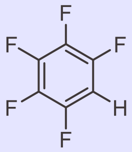 **2a'**	No reaction (without electron-withdrawing group)
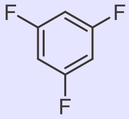 **2b'**	No reaction (without electron-withdrawing group)	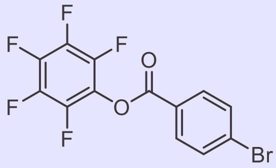 **2c'**	No desired product (carbonyl addition)

Back to the table

2.6 Substrate scopes data of NHC-Catalyzed S_NAr reaction

(Perfluoropyridin-4-yl)(phenyl)methanone, 3a

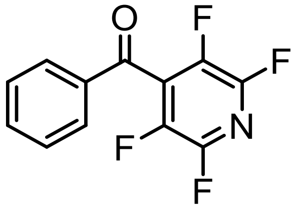

3a

According to **General Procedure A**, benzaldehyde **1a** (10.6 mg, 0.1 mmol, 1.0 equiv) and pentafluoropyridine **2a** (16.9 mg, 0.1 mmol, 1.0 equiv) were used to obtain crude residue, which was purified by silica gel column chromatography (Hexane) to afford the **3a** as pale-yellow liquid (23.5 mg, 0.092 mmol, 92%).

^{1}H NMR (300 MHz, $CDCl_3$) δ 7.87 (d, J = 7.7 Hz, 2H), 7.74 (t, J = 7.4 Hz, 1H), 7.57 (t, J = 7.7 Hz, 2H) ppm.

^{13}C{^{1}H} NMR (101 MHz, $CDCl_3$) δ 184.4, 144.9 – 142.1 (m), 140.0 – 137.0 (m), 135.7, 134.6, 131.5 (t, J = 18.7 Hz) 129.7, 129.4 ppm.

^{19}F NMR (376 MHz, $CDCl_3$) δ -87.9 – -88.0 (m), -141.4 – -141.5 (m) ppm.

See NMR spectra

HRMS (*m/z*): (EI) calc'd for $C_{12}H_5F_4NO$ $[M]^+$: 255.0299, found 255.0302

IR (neat) ν_{max}: 2920, 2850, 1460, 1287, 965, 832, 672 cm^{-1}

TLC: R_f = 0.46 (Hexane/ethyl acetate 9:1)

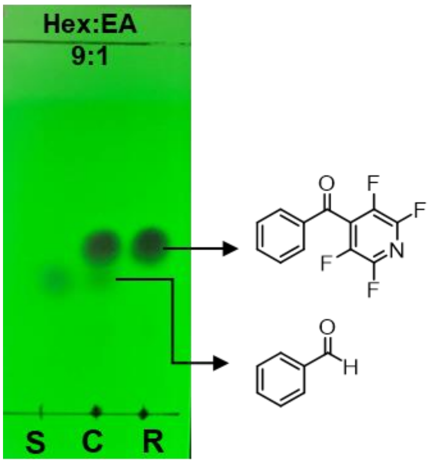

Back to the table

(Perfluoropyridin-4-yl)(4-(trifluoromethyl)phenyl)methanone, 3b

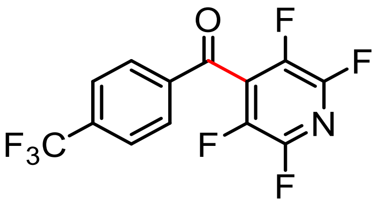

3b

According to **General Procedure A**, 4-(trifluoromethyl)benzaldehyde **1b** (17.4 mg, 0.1 mmol, 1.0 equiv) and pentafluoropyridine **2a** (16.9 mg, 0.1 mmol, 1.0 equiv) were used to obtain crude residue, which was purified by silica gel column chromatography (Hexane/ethyl acetate 10:1) to afford the **3b** as colorless oil (22.9 mg, 0.071mmol, 71%).

^{1}H NMR (300 MHz, $CDCl_3$) δ 7.99 (d, J = 8.1, 2H), 7.84 (d, J = 8.1, 2H) ppm.

^{13}C{^{1}H} NMR (101 MHz, $CDCl_3$) δ 183.6, 145.0 – 142.2 (m), 140.0 – 137.1 (m), 137.2, 136.9, 136.6, 130.7 – 130.3 (m), 130.02, 126.5 (q, J = 3.72 Hz), 123.1 (q, J = 273.04 Hz) ppm.

^{19}F NMR (282 MHz, $CDCl_3$) δ -63.5 (s, 1F), -87.3 (ddd, J = 26.7, 26.6, 12.1 Hz), -141.2 – -141.4 (m) ppm.

See NMR spectra

HRMS (*m/z*): (EI) calc’d for $C_{13}H_4NOF_7$ $[M]^+$: 323.0176, found 323.0174

IR (neat) ν_{max}: 2919, 2850, 1689, 1484, 1321, 1133, 992 cm^{-1}

TLC: R_f = 0.58 (Hexane/ethyl acetate 9:1)

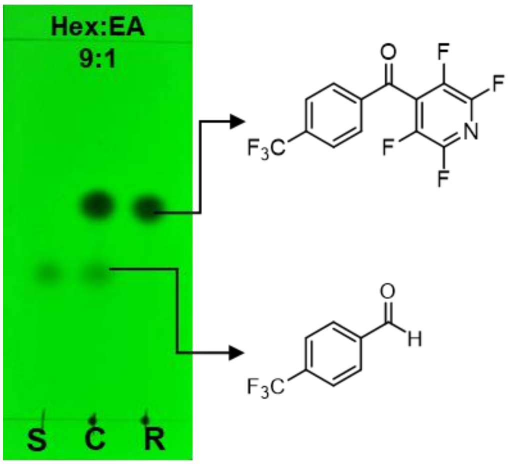

Back to the table

4-(Perfluoroisonicotinoyl)benzonitrile, 3c

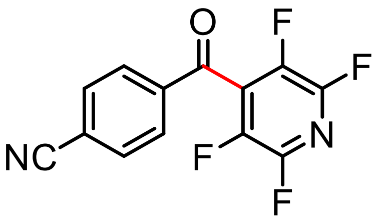

3c

According to **General Procedure A**, 4-formylbenzonitrile **1c** (13.1 mg, 0.1 mmol, 1.0 equiv) and pentafluoropyridine **2a** (16.9 mg, 0.1 mmol, 1.0 equiv) were used to obtain crude residue, which was purified by silica gel column chromatography (Hexane/ethyl acetate 10:1) to afford the **3c** as white solid (27.8 mg, 0.099 mmol, 99%).

^{1}H NMR (300 MHz, $CDCl_3$) δ 7.98 (d, J = 8.5 Hz, 2H), 7.87 (d, J = 8.7 Hz, 2H) ppm.

^{13}C{^{1}H} NMR (101 MHz, $CDCl_3$) δ 183.3, 145.0 – 142.2 (m), 140.0 – 137.0 (m), 137.3, 133.1, 129.9, 118.8, 117.2 ppm.

^{19}F NMR (282 MHz, $CDCl_3$) δ -86.9 (ddd, J = 27.0, 26.5, 9.4 Hz), -141.9 – -141.1 (m) ppm.

See NMR spectra

HRMS (*m/z*): (EI) calc'd for $C_{13}H_4N_2OF_4$ [M]$^+$: 280.0254, found 280.0257

IR (neat) ν_{max}: 2926, 2854, 2232, 1689, 1459, 1284 cm^{-1}

M.P.: 130 °C

TLC: R_f = 0.31 (Hexane/ethyl acetate 9:1)

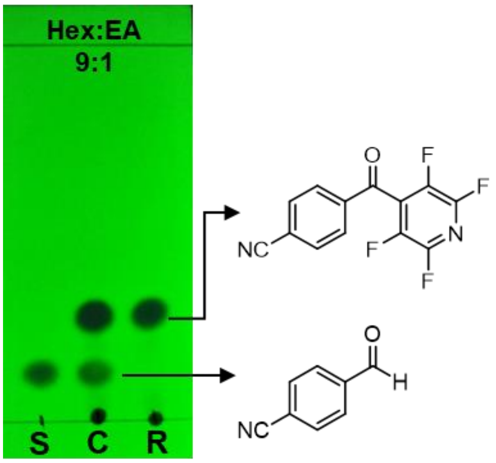

[Back to the table](#)

Methyl 4-(perfluoroisonicotinoyl)benzoate, 3d

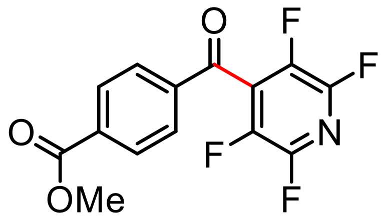

3d

According to **General Procedure A**, methyl 4-formylbenzoate **1d** (16.4 mg, 0.1 mmol, 1.0 equiv) and pentafluoropyridine **2a** (16.9 mg, 0.1 mmol, 1.0 equiv) were used to obtain crude residue, which was purified by silica gel column chromatography (Hexane/ethyl acetate 15:1) to afford the **3d** as white solid (20.9 mg, 0.067 mmol, 67%).

^{1}H NMR (300 MHz, $CDCl_3$) δ 8.20 (d, J = 8.1 Hz, 2H), 7.92 (d, J = 8.2 Hz, 2H), 3,97 (s, 3H) ppm.

^{13}C{^{1}H} NMR (101 MHz, $CDCl_3$) δ 184.0, 165.6, 144.9 – 142.2 (m), 140.0 – 137.0 (m), 137.5, 136.1, 130.8 (t, J = 19.0 Hz), 130.4, 129.6, 52.7 ppm.

^{19}F NMR (282 MHz, $CDCl_3$) δ -87.5 – -87.7 (m), -141.3 – -141.5 ppm.

See NMR spectra

HRMS (*m/z*): (EI) calc'd for $C_{14}H_7NO_3F_4$ $[M]^+$: 313.0357, found 313.0353

IR (neat) ν_{max}: 2961, 2923, 2851, 1723, 1683, 1462, 1281, 964 cm^{-1}

M.P.: 128 ℃

TLC: R_f = 0.45 (Hexane/ethyl acetate 9:1)

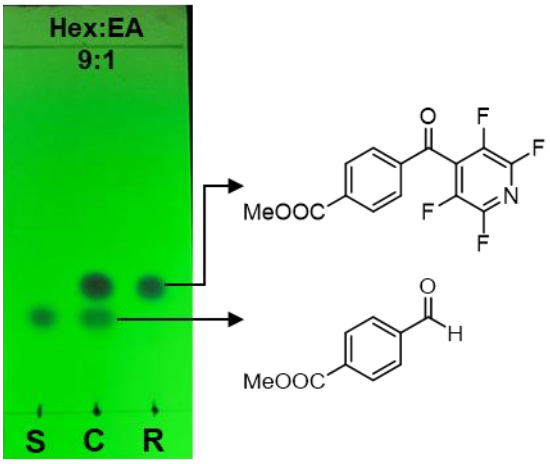

Back to the table

(4-Nitrophenyl)(perfluoropyridin-4-yl)methanone, 3e

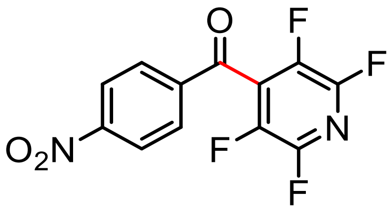

3e

According to **General Procedure A**, 4-nitrobenzaldehyde **1e** (15.1 mg, 0.1 mmol, 1.0 equiv) and pentafluoropyridine **2a** (16.9 mg, 0.1 mmol, 1.0 equiv) were used to obtain crude residue, which was purified by silica gel column chromatography (Hexane/ethyl acetate 10:1) to afford the **3e** as pale-yellow solid (26.0 mg, 0.087 mmol, 87%).

^{1}H NMR (300 MHz, $CDCl_3$) δ 8.42 – 8.39 (m, 2H), 8.08 – 8.05 (m, 2H) ppm.

^{13}C{^{1}H} NMR (101 MHz, $CDCl_3$) δ 183.1, 151.6, 145.1 – 142.3 (m), 140.0 – 137.1 (m), 138.7, 130.7, 129.9 (t, J = 18.7 Hz), 124.5 ppm.

^{19}F NMR (282 MHz, $CDCl_3$) δ -86.8 – -87.0 (m), -140.8 – -141.1 (m) ppm.

See NMR spectra

HRMS (*m/z*): (EI) calc'd for $C_{12}H_4F_4N_2O_3$ $[M]^+$: 300.0153, found 300.0151

IR (neat) ν_{max}: 1691, 1525, 1460, 1348, 1284, 962, 823 cm^{-1}

M.P.: 98 ℃

TLC: R_f = 0.26 (Hexane/ethyl acetate 9:1)

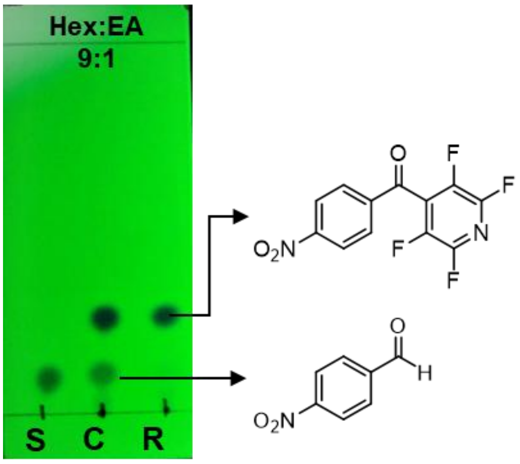

Back to the table

(4-Fluorophenyl)(perfluoropyridin-4-yl)methanone, 3f

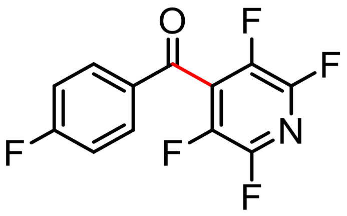

3f

According to **General Procedure A**, 4-fluorobenzaldehyde **1f** (12.4 mg, 0.1 mmol, 1.0 equiv) and pentafluoropyridine **2a** (16.9 mg, 0.1 mmol, 1.0 equiv) were used to obtain crude residue, which was purified by silica gel column chromatography (Hexane/ethyl acetate 9:1) to afford the **3f** as colorless oil (27.1 mg, 0.099 mmol, 99%).

^{1}H NMR (300 MHz, $CDCl_3$) δ 7.93 – 7.88 (m, 2H), 7.27 – 7.22 (m, 2H) ppm.

^{13}C{^{1}H} NMR (101 MHz, $CDCl_3$) δ 182.7, 167.3 (d, J = 260.1 Hz), 144.8 – 142.3 (m), 139.6 – 137.3 (m), 132.6 (d, J = 10.1 Hz), 131.3 (d, J = 3.0 Hz), 116.9 (d, J = 22.3 Hz) ppm.

^{19}F NMR (376 MHz, $CDCl_3$) δ -88.2 – -88.4 (m), -100.0, -142.0 – -142.2 (m) ppm.

See NMR spectra

HRMS (*m/z*): (EI) calc'd for $C_{12}H_4NOF_5$ $[M]^+$: 273.0208, found 273.0210

IR (neat) ν_{max}: 2922, 2851, 1680, 1595, 1455, 1287, 850 cm^{-1}

TLC: R_f = 0.50 (Hexane/ethyl acetate 9:1)

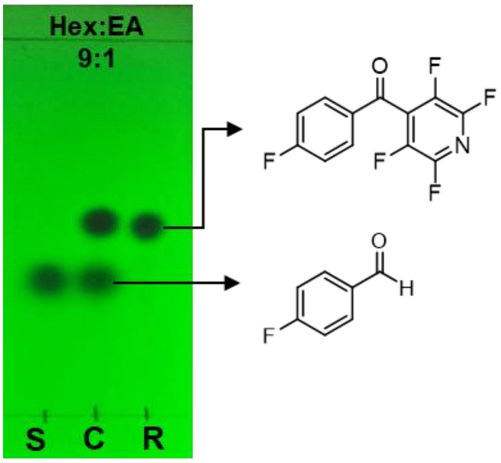

Back to the table

(4-Chlorophenyl)(perfluoropyridin-4-yl)methanone, 3g

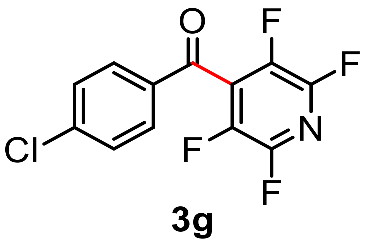

3g

According to **General Procedure A**, 4-chlorobenzaldehyde **1g** (14.0 mg, 0.1 mmol, 1.0 equiv) and pentafluoropyridine **2a** (16.9 mg, 0.1 mmol, 1.0 equiv) were used to obtain crude residue, which was purified by silica gel column chromatography (Hexane/ethyl acetate 10:1) to afford the **3g** as pale-yellow oil (26.1 mg, 0.090 mmol, 90%).

^{1}H NMR (300 MHz, $CDCl_3$) δ 7.80 (d, J = 8.5 Hz, 2H), 7.55 – 7.52 (d, J = 8.6 Hz, 2H) ppm.

^{13}C{^{1}H} NMR (101 MHz, $CDCl_3$) δ 183.2, 144.8 – 142.6 (m), 142.3 – 140.0 (m), 137.3 – 137.0 (m), 133.0 (m), 130.1, 129.8 ppm.

^{19}F NMR (282 MHz, $CDCl_3$) δ -87.7 (ddd, J = 26.7, 26.7, 9.7 Hz), -141.4 – -141.6 (m) ppm.

See NMR spectra

HRMS (*m/z*): (EI) calc'd for $C_{12}H_4ClF_4NO$ $[M]^+$: 288.9912, found 288.9910

IR (neat) ν_{max}: 2922, 2851, 1680, 1448, 1376, 1266, 831 cm^{-1}

TLC: R_f = 0.53 (Hexane/ethyl acetate 9:1)

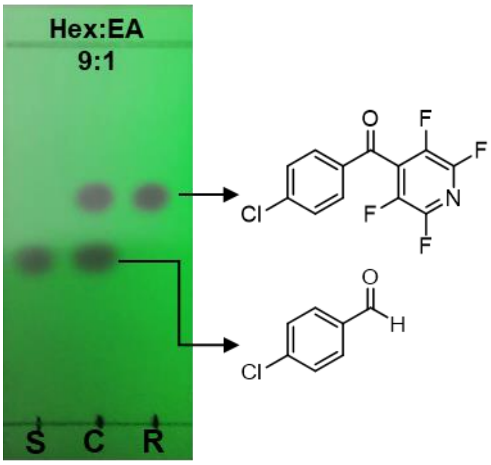

Back to the table

(4-Bromophenyl)(perfluoropyridin-4-yl)methanone, 3h

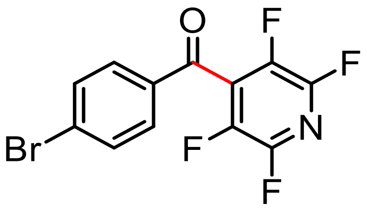

3h

According to **General Procedure A**, 4-bromobenzaldehyde **3h** (18.5 mg, 0.1 mmol, 1.0 equiv) and pentafluoropyridine **2a** (16.9 mg, 0.1 mmol, 1.0 equiv) were used to obtain crude residue, which was purified by silica gel column chromatography (Hexane/ethyl acetate 9:1) to afford the **3h** as white solid (33.1 mg, 0.099 mmol, 99%).

^{1}H NMR (300 MHz, $CDCl_3$) δ 7.71 (s, 4H) ppm.

^{13}C{^{1}H} NMR (101 MHz, $CDCl_3$) δ 183.4, 144.9 – 142.1 (m), 139.9 – 137.0 (m), 133.4, 132.8, 131.5, 130.9, 130.7 ppm.

^{19}F NMR (282 MHz, $CDCl_3$) δ -87.7 (ddd, J = 26.8, 26.6, 9.6 Hz), -141.4 – -141.6 (m) ppm.

See NMR spectra

HRMS (*m/z*): (EI) calc'd for $C_{12}H_4NOF_4Br$ $[M]^+$: 332.9407, found 332.9410

IR (neat) ν_{max}: 1682, 1587, 1458, 1410, 1284, 968 cm^{-1}

M.P.: 82 ℃

TLC: R_f = 0.62 (Hexane/ethyl acetate 9:1)

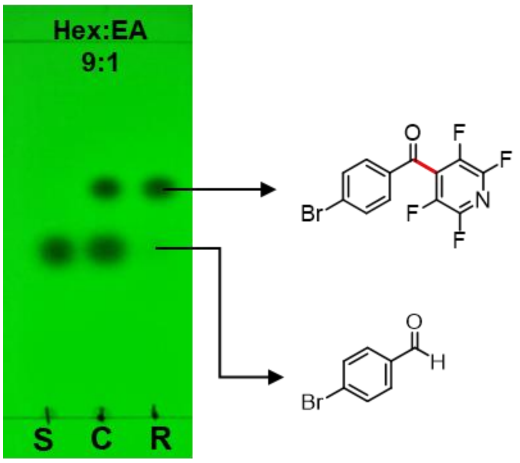

Back to the table

(Perfluoropyridin-4-yl)(p-tolyl)methanone, 3i

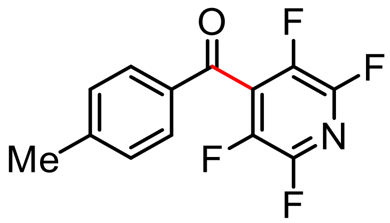

3i

According to **General Procedure A**, 4-methylbenzaldehyde **1i** (12.0 mg, 0.1 mmol, 1.0 equiv) and pentafluoropyridine **2a** (16.9 mg, 0.1 mmol, 1.0 equiv) were used to obtain crude residue, which was purified by preparative TLC plate (Hexane/ethyl acetate 15:1) to afford the **3i** as colorless oil (14.8 mg, 0.055 mmol, 55%).

^{1}H NMR (300 MHz, $CDCl_3$) δ 7.75 (d, 2H, J = 8.02 Hz), 7.36 (d, 2H, J = 7.97 Hz), 2.47 (s, 3H) ppm.

^{13}C NMR (400 MHz, $CDCl_3$) δ 183.8, 147.3, 145.3 – 141.9 (m), 139.6 – 137.4 (m), 132.3, 130.1, 129.9, 22.0 ppm.

^{19}F NMR (282 MHz, $CDCl_3$) δ -88.3 (ddd, J = 26.9, 26.8, 9.4 Hz), -141.7 – -141.9 (m) ppm.

See NMR spectra

HRMS (*m/z*): (EI) calc'd for $C_{13}H_7NOF_4$ $[M]^+$: 269.0458, found 269.0456

IR (neat) ν_{max}: 2923, 2851, 1676, 1604, 1458, 1287, 969, 842

TLC: R_f = 0.55 (Hexane/ethyl acetate 9:1)

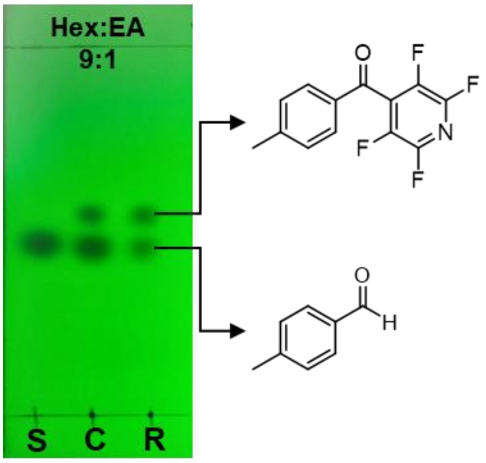

Back to the table

(Perfluoropyridin-4-yl)(4-(4,4,5,5-tetramethyl-1,3,2-dioxaborolan-2-yl)phenyl)methanone, 3j

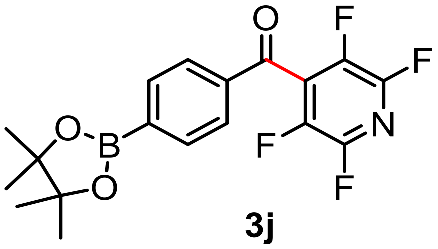

3j

According to **General Procedure A**, 4-(4,4,5,5-tetramethyl-1,3,2-dioxaborolan-2-yl)benzaldehyde **1j** (23.2 mg, 0.1 mmol, 1.0 equiv) and pentafluoropyridine **2a** (16.9 mg, 0.1 mmol, 1.0 equiv) were used to obtain a crude residue, which was purified by silica gel column chromatography (Hexane/ethyl acetate 15:1) to afford the **3j** as white solid (24.1 mg, 0.064 mmol, 64%).

^{1}H NMR (300 MHz, $CDCl_3$) δ 7.98 (d, J = 8.4 Hz, 2H), 7.82 (d, J = 7.9 Hz, 2H), 1.36 (s, 12H) ppm.

^{13}C{^{1}H} NMR (101 MHz, $CDCl_3$) δ 184.7, 144.8 – 142.3 (m), 140.0 – 137.4 (m), 136.3, 135.5, 128.6, 84.5, 24.8 ppm.

^{19}F NMR (376 MHz, $CDCl_3$) δ -88.0 (ddd, J = 26.6, 26.5, 9.8 Hz), -141.4 – -141.9 (m) ppm.

See NMR spectra

HRMS (*m/z*): (EI) calc'd for $C_{18}H_{16}BNO_3F_4$ $[M]^+$: 381.1154, found 381.1157

IR (neat) ν_{max}: 2981, 2930, 1683, 1459, 1359, 1289, 1142, 1090, 961, 809 cm^{-1}

M.P.: 84 °C

TLC: R_f = 0.73 (Hexane/ethyl acetate 7:3)

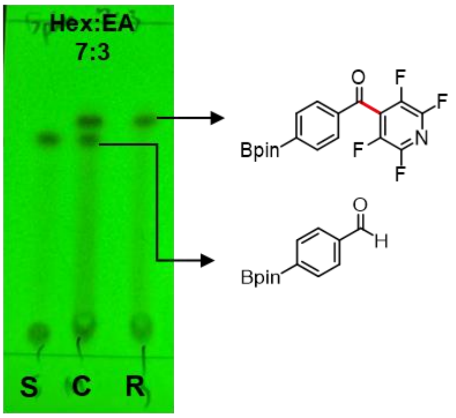

Back to the table

Methyl 3-(perfluoroisonicotinoyl)benzoate, 3k

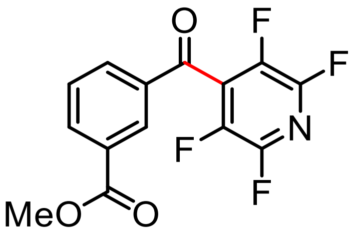

3k

According to **General Procedure A**, methyl 3-formylbenzoate **1k** (16.4 mg, 0.1 mmol, 1.0 equiv) and pentafluoropyridine **2a** (16.9 mg, 0.1 mmol, 1.0 equiv) were used to obtain crude residue, which was purified by silica gel column chromatography (Hexane/ethyl acetate 15:1) to afford the **3k** as colorless oil (25.1 mg, 0.080 mmol, 80%).

^{1}H NMR (300 MHz, $CDCl_3$) δ 8.42 (s, 1H), 8.40 – 8.37 (m, 1H), 8.11 – 8.08 (m, 1H), 7.68 (t, J = 7.8 Hz, 1H), 3.96 (s, 3H) ppm.

^{13}C{^{1}H} NMR (101 MHz, $CDCl_3$) δ 183.8, 165.5, 145.0 – 142.2 (m), 140.0 – 137.4 (m), 137.1, 136.3, 135.0, 133.3, 131.6, 130.7, 129.7, 52.7 ppm.

^{19}F NMR (282 MHz, CDCl3) δ -87.4 – -87.7 (m), -141.3 – -141.5 (m) ppm.

See NMR spectra

HRMS (*m/z*): (ESI) calc'd for $C_{14}H_8O_3NF_4$ $[M+H]^+$: 314.0435, found: 314.0434

IR (neat) ν_{max}: 2957, 1726, 1461, 1268, 1209, 969, 744 cm^{-1}

TLC: R_f = 0.35 (Hexane/ethyl acetate 9:1)

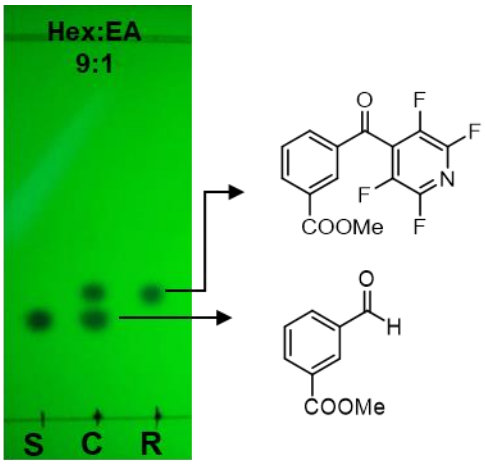

Back to the table

(3-Nitrophenyl)(perfluoropyridin-4-yl)methanone, 3l

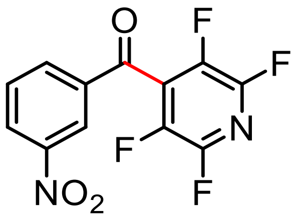

3l

According to **General Procedure A**, 3-nitrobenzaldehyde **1l** (15.1 mg, 0.1 mmol, 1.0 equiv) and pentafluoropyridine **2a** (16.9 mg, 0.1 mmol, 1.0 equiv) were used to obtain crude residue, which was purified by silica gel column chromatography (Hexane/ethyl acetate 25:1→15:1) to afford the **3l** as pale-yellow oil (14.9 mg, 0.050 mmol, 50%).

^{1}H NMR (300 MHz, $CDCl_3$) δ 8.68 – 8.66 (m, 1H), 8.61 – 8.57 (m, 1H), 8.22 – 8.19 (m, 1H), 7.82 (t, J = 8.0 Hz, 1H) ppm.

^{13}C{^{1}H} NMR (101 MHz, $CDCl_3$) δ 182.6, 148.8, 145.0 – 142.3 (m), 140.1 – 137.2 (m), 135.9, 134.8, 130.8, 130.0 – 129.8 (m), 129.7, 124.3 ppm.

^{19}F NMR (282 MHz, $CDCl_3$) δ -86.6 – -86.8 (m), -140.8 – -141.1 (m) ppm.

See NMR spectra

HRMS (*m/z*): (EI) calc'd for $C_{12}H_4N_2O_3F_4$ [M]$^+$: 300.0153, found 300.0149

IR (neat) ν_{max}: 3088, 2923, 2851, 1533, 1461, 1351, 969 cm^{-1}

TLC: R_f = 0.20 (Hexane/ethyl acetate 9:1)

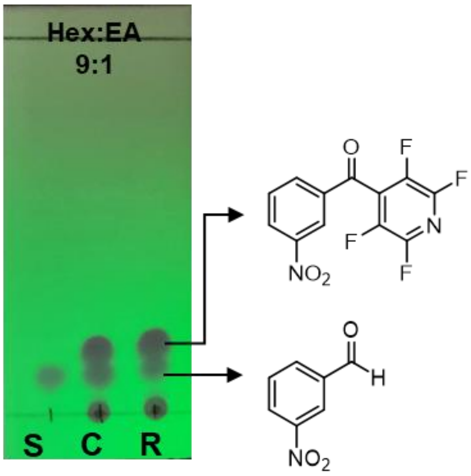

Back to the table

(3-Bromophenyl)(perfluoropyridin-4-yl)methanone, 3m

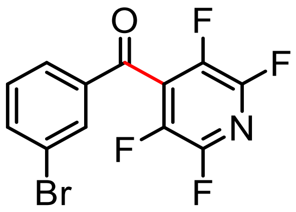

3m

According to **General Procedure A**, 3-bromobenzaldehyde **1m** (18.5 mg, 0.1 mmol, 1.0 equiv) and pentafluoropyridine **2a** (16.9 mg, 0.1 mmol, 1.0 equiv) were used to obtain crude residue, which was purified by silica gel column chromatography (Hexane/ethyl acetate 20:1) to afford the **3m** as colorless oil (19.1 mg, 0.057 mmol, 57%).

^{1}H NMR (400 MHz, $CDCl_3$) δ 8.010 – 8.006 (m, 1H), 7.86 – 7.74 (m, 2H), 7.47 – 7.43 (m, 1H) ppm.

^{13}C{^{1}H} NMR (101 MHz, $CDCl_3$) δ 183.2, 144.9 – 142.1 (m), 140.0 – 137.0 (m), 138.6, 136.3, 132.3, 130.9, 130.7 – 130.3 (m), 128.3, 123.7 ppm.

^{19}F NMR (282 MHz, $CDCl_3$) δ -87.5 (ddd, 28.59, 28.39, 12.55 Hz), -141.2 – -142.4 (m) ppm.

See NMR spectra

HRMS (*m/z*): (EI) calc'd for $C_{12}H_4NOF_4Br$ [M^+]: 332.9407, found 332.9404

IR (neat) ν_{max}: 2917, 2851, 1683, 1462, 1288, 968, 679 cm^{-1}

TLC: R_f = 0.42 (Hexane/ethyl acetate 9:1)

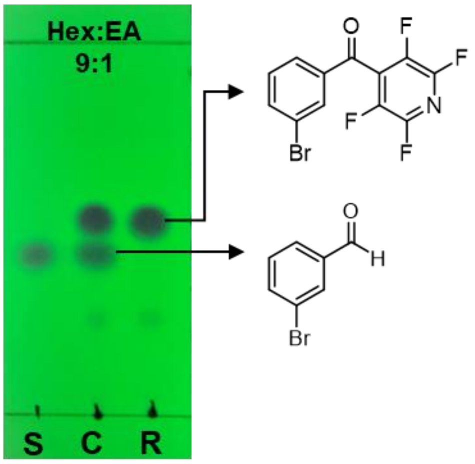

Back to the table

Methyl 2-(perfluoroisonicotinoyl)benzoate, 3n

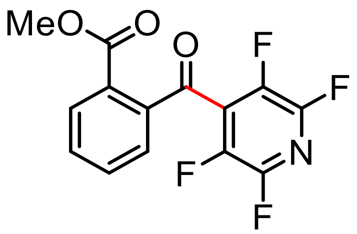

3n

According to **General Procedure A**, methyl 2-formylbenzoate **1n** (16.4 mg, 0.1 mmol, 1.0 equiv) and pentafluoropyridine **2a** (16.9 mg, 0.1 mmol, 1.0 equiv) were used to obtain crude residue, which was purified by silica gel column chromatography (Hexane/ethyl acetate 30:1 →20:1) to afford the **3n** as a colorless oil (22.2 mg, 0.071 mmol, 71%).

^{1}H NMR (300 MHz, $CDCl_3$) δ 7.99 – 7.96 (m, 1H), 7.74 – 7.65 (m, 2H), 7.58 – 7.55 (m, 1H), 3.81 (s, 3H) ppm.

^{13}C{^{1}H} NMR (101 MHz, $CDCl_3$) δ 186.5, 166.5, 145.1 – 142.3 (m), 140.8 – 137.8 (m), 140.1, 132.8, 132.2, 130.7 – 130.4 (m), 130.2, 129.3, 128.8, 52.9 ppm.

^{19}F NMR (282 MHz, CDCl3) δ -88.8 – -89.0 (m), -141.0 – -141.3 (m) ppm.

See NMR spectra

HRMS (*m/z*): (ESI) calc'd for $C_{14}H_7O_3NF_4{}^{23}Na$ $[M+Na]^+$: 336.0254 , found 336.0254

IR (neat) ν_{max}: 2919, 2852, 1696, 1463, 1284, 961, 708 cm^{-1}

TLC: R_f = 0.22 (Hexane/ethyl acetate 9:1)

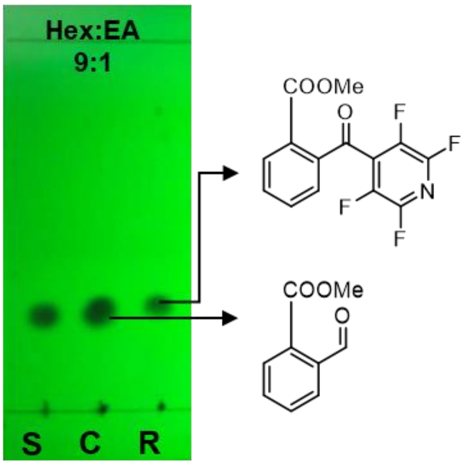

Back to the table

(2-Nitrophenyl)(perfluoropyridin-4-yl)methanone, 3o

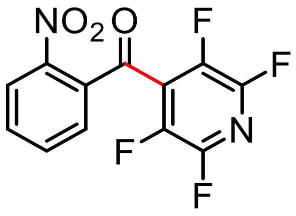

3o

According to **General Procedure A**, 2-nitrobenzaldehyde **1o** (15.1 mg, 0.1 mmol, 1.0 equiv) and pentafluoropyridine **2a** (16.9 mg, 0.1 mmol, 1.0 equiv) were used to obtain crude residue, which was purified by preparative TLC plate (Hexane/ethyl acetate 25:1) to afford the **3o** as pale-yellow solid (9.1 mg, 0.030 mmol, 30%).

^{1}H NMR (300 MHz, $CDCl_3$) δ 8.23 (d, J = 8.1 Hz, 1H), 7.93 – 7.76 (m, 2H), 7.67 (d, J = 7.4, 1.6 Hz, 1H) ppm.

^{13}C{^{1}H} NMR (101 MHz, $CDCl_3$) δ 183.3, 146.1, 145.4 – 142.4 (m), 140.9 – 137.9 (m), 135.0, 132.8, 129.6, 124.7 ppm.

^{19}F NMR (282 MHz, $CDCl_3$) δ -87.7 – -88.0 (m), -141.1 – -141.3 (m) ppm.

See NMR spectra

HRMS (*m/z*): (EI) calc'd for $C_{12}H_4F_4N_2O_3$ [M^+]: 300.0153, found 300.0150

IR (neat) ν_{max}: 2922, 2854, 1699, 1530, 1461, 1345, 1294, 969, 828 cm^{-1}

M.P.: 99 °C

TLC: R_f = 0.20 (Hexane/ethyl acetate 9:1)

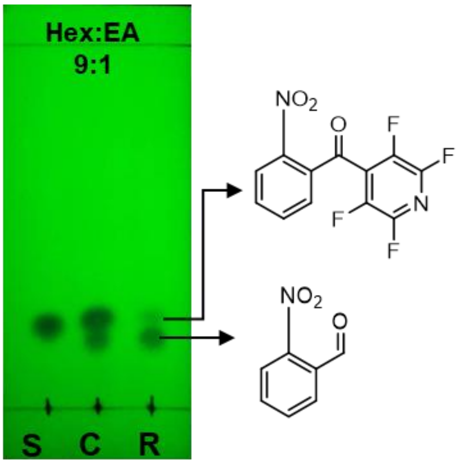

Back to the table

(2-Bromophenyl)(perfluoropyridin-4-yl)methanone, 3p

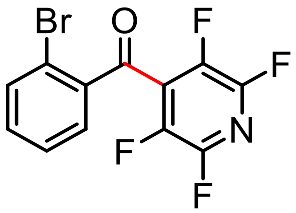

3p

According to **General Procedure A**, 2-bromobenzaldehyde **1p** (18.5 mg, 0.1 mmol, 1.0 equiv) and pentafluoropyridine **2a** (16.9 mg, 0.1 mmol, 1.0 equiv) were used to obtain crude residue, which was purified by silica gel column chromatography (Hexane/ethyl acetate 20:1) to afford the **3p** as colorless oil (24.7 mg, 0.074 mmol, 74%).

^{1}H NMR (400 MHz, $CDCl_3$) δ 7.72 – 7.64 (m, 2H), 7.52 – 7.46 (m, 2H) ppm.

^{13}C{^{1}H} NMR (101 MHz, $CDCl_3$) δ 184.8, 144.9 – 142.1 (m), 140.4 – 137.4 (m), 137.1, 134.7, 131.8, 131.7 – 131.3 (m), 128.1, 121.1 ppm.

^{19}F NMR (282 MHz, $CDCl_3$) δ -88.1 – -88.4 (m), -141.6 – -141.8 (m) ppm.

See NMR spectra

HRMS (*m/z*): (EI) calc'd for $C_{12}H_4NOF_4Br$ [M^+]: 332.9407, found 332.9405

IR (neat) ν_{max}: 1684, 1586, 1459, 1406, 1294, 968, 832, 744 cm^{-1}

TLC: R_f = 0.65 (Hexane/ethyl acetate 9:1)

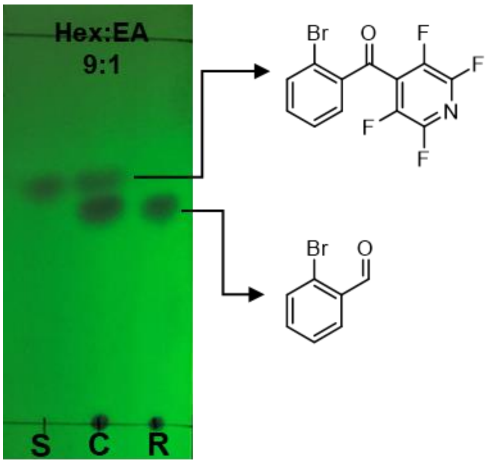

Back to the table

(2-Chloro-5-nitrophenyl)(perfluoropyridin-4-yl)methanone, 3q

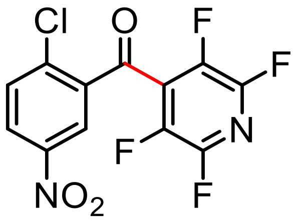

3q

According to **General Procedure A**, 2-chloro-5-nitrobenzaldehyde **1q** (18.5 mg, 0.1 mmol, 1.0 equiv) and pentafluoropyridine **2a** (16.9 mg, 0.1 mmol, 1.0 equiv) were used to obtain crude residue, which was purified by preparative TLC plate (Hexane/ethyl acetate 25:1→15:1) to afford the **3q** as pale-yellow solid (27.1 mg, 0.081 mmol, 81%).

^{1}H NMR (300 MHz, $CDCl_3$) δ 8.57 (d, J = 2.7 Hz, 1H), 8.42 (dd, J = 8.8, 2.7 Hz, 1H), 7.72 (d, J = 8.8 Hz, 1H) ppm.

^{13}C{^{1}H} NMR (101 MHz, $CDCl_3$) δ 182.3, 146.9, 145.1 – 142.3 (m), 140.4 – 137.4 (m), 139.2, 136.3, 132.5, 130.5 – 130.1 (m), 128.6, 126.2 ppm.

^{19}F NMR (282 MHz, $CDCl_3$) δ -86.9 – -87.1 (m), -141.5 – -141.7 (m) ppm.

See NMR spectra

HRMS (*m/z*): (EI) calc'd for $C_{12}H_3N_2O_3F_4Cl$ [M^+]: 333.9763, found 333.9760

IR (neat) ν_{max}: 2923, 2854, 1608, 1530, 1461, 1348, 739 cm^{-1}

M.P.: 102 °C

TLC: R_f = 0.36 (Hexane/ethyl acetate 9:1)

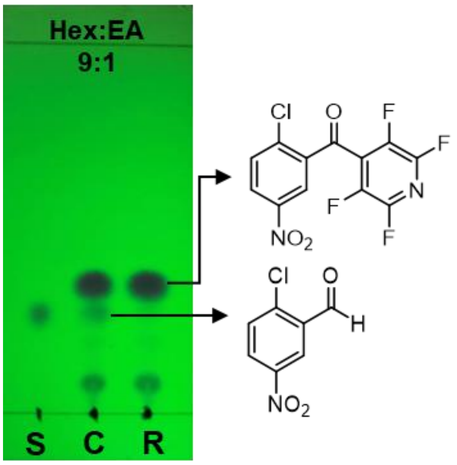

Back to the table

(5-Chloro-2-nitrophenyl)(perfluoropyridin-4-yl)methanone, 3r

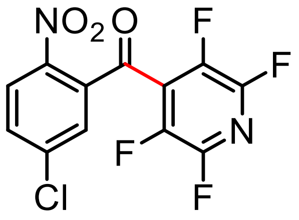

3r

According to **General Procedure A**, 5-chloro-2-nitrobenzaldehyde **1r** (18.5 mg, 0.1 mmol, 1.0 equiv) and pentafluoropyridine **2a** (16.9 mg, 0.1 mmol, 1.0 equiv) were used to obtain crude residue, which was purified by silica gel column chromatography (Hexane/ethyl acetate 25:1 →15:1) to afford the **3r** as pale-yellow solid (24.0 mg, 0.072 mmol, 72%).

^{1}H NMR (300 MHz, $CDCl_3$) δ 8.20 (d, J = 8.8 Hz, 1H), 7.74 (dd, J = 8.8, 2.2 Hz, 1H), 7.62 (d, J = 2.2 Hz, 1H) ppm.

^{13}C{^{1}H} NMR (101 MHz, $CDCl_3$) δ 181.8, 145.2 – 142.5 (m), 144.1, 142.2, 140.7 – 138.0 (m), 136.5, 132.5, 129.5, 126.1 ppm.

^{19}F NMR (282 MHz, $CDCl_3$) δ -87.3 – -87.5 (m), -140.8 – -141.1 (m) ppm.

See NMR spectra

HRMS (*m/z*): (EI) calc'd for $C_{12}H_3N_2O_3F_4Cl$ [M^+]: 333.9763, found 333.9766

IR (neat) ν_{max}: 2919, 2852, 1696, 1463, 1284, 961, 708 cm^{-1}

TLC: R_f = 0.38 (Hexane/ethyl acetate 9:1)

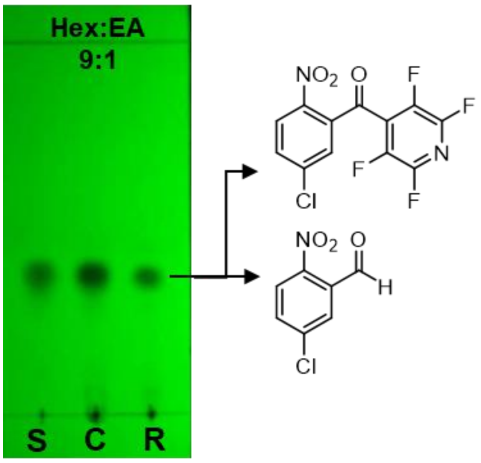

Back to the table

(3-Bromo-4-fluorophenyl)(perfluoropyridin-4-yl)methanone, 3s

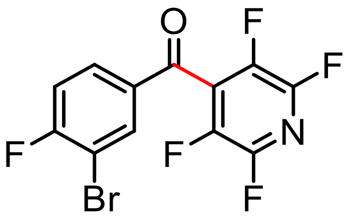

3s

According to **General Procedure A**, 3-bromo-4-fluorobenzaldehyde **1s** (20.3 mg, 0.1 mmol, 1.0 equiv) and pentafluoropyridine **2a** (16.9 mg, 0.1 mmol, 1.0 equiv) were used to obtain crude residue, which was purified by silica gel column chromatography (Hexane/ethyl acetate 20:1) to afford the **3s** as pale-yellow liquid (23.2 mg, 0.066 mmol, 66%).

^{1}H NMR (400 MHz, $CDCl_3$) δ 8.12 (dd, J = 6.4, 2.2 Hz, 1H), 7.82 – 7.77 (m, 1H), 7.30 (dd, J = 8.6, 7.8 Hz, 1H) ppm.

^{13}C{^{1}H} NMR (101 MHz, $CDCl_3$) δ 181.8, 163.6 (d, J = 260.0 Hz), 143.5 (dt, J = 248.9, 14.0 Hz), 139.9 – 137.0 (m), 135.4, 132.2 (d, J = 3.5 Hz), 131.1 (d, J = 9.3 Hz), 130.4 (t, J = 19.3 Hz), 117.6 (d, J = 23.4 Hz), 111.0 (d, J = 22.2 Hz) ppm.

^{19}F NMR (282 MHz, $CDCl_3$) δ -87.1 – -87.3 (m), -93.8, -141.2 – -141.4 (m) ppm.

See NMR spectra

HRMS (*m/z*): (EI) calc'd for $C_{12}H_3NOF_5Br$ $[M]^+$: 350.9313, found 350.9313

IR (neat) ν_{max}: 1686, 1459, 1289, 1264, 1048, 969, 777 cm^{-1}

TLC: R_f = 0.62 (Hexane/ethyl acetate 9:1)

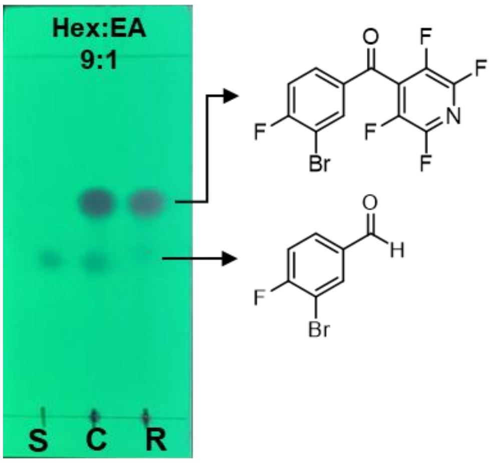

Back to the table

[1,1'-Biphenyl]-4-yl(perfluoropyridin-4-yl)methanone, 3t

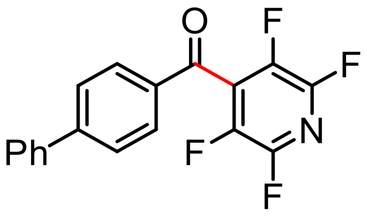

3t

According to **General Procedure A**, [1,1'-biphenyl]-4-carbaldehyde **1t** (18.2 mg, 0.1 mmol, 1.0 equiv) and pentafluoropyridine **2a** (16.9 mg, 0.1 mmol, 1.0 equiv) were used to obtain crude residue, which was purified by silica gel column chromatography (Hexane/ethyl acetate 9:1) to afford the **3t** as pale-yellow solid (25.8 mg, 0.078 mmol, 78%).

^{1}H NMR (300 MHz, $CDCl_3$) δ 7.94 (d, J = 8.5 Hz, 2H), 7.78 (d, J = 8.6 Hz, 2H), 7.66 – 7.63 (m, 2H), 7.53 – 7.44 (m, 3H) ppm.

^{13}C{^{1}H} NMR (101 MHz, $CDCl_3$) δ 183.8, 148.5, 144.9 – 142.1 (m), 140.0 – 137.1 (m), 139.1, 133.3, 131.8 – 131.4 (m), 130.4, 129.1, 128.9, 127.9, 127.4 ppm.

^{19}F NMR (282 MHz, $CDCl_3$) δ -88.0 (ddd, J = 28.8, 28.5, 12.6 Hz), -141.4 – -141.7 (m) ppm.

See NMR spectra

HRMS (*m/z*): (EI) calc'd for $C_{18}H_9NOF_4$ $[M]^+$: 331.0615, found 331.0617

IR (neat) ν_{max}: 2923, 2851, 1683, 1459, 1273, 967, 695 cm^{-1}

M.P.: 105 °C

TLC: R_f = 0.51(Hexane/ethyl acetate 9:1)

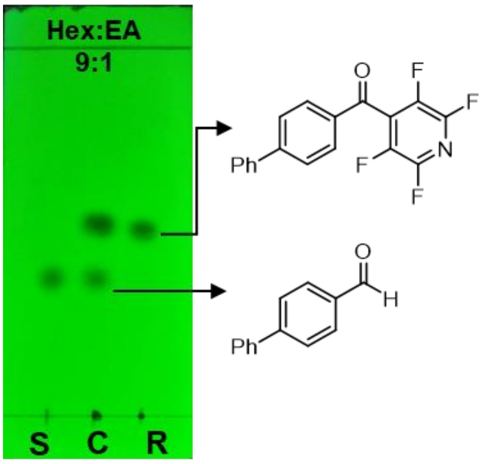

Back to the table

Naphthalen-2-yl(perfluoropyridin-4-yl)methanone, 3u

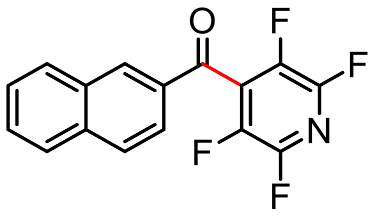

3u

According to **General Procedure A**, 2-naphthaldehyde **1u** (15.6 mg, 0.1 mmol, 1.0 equiv) and pentafluoropyridine **2a** (16.9 mg, 0.1 mmol, 1.0 equiv) were used to obtain crude residue, which was purified by silica gel column chromatography (Hexane/ethyl acetate 25:1) to afford the **3u** as white solid (20.4 mg, 0.067 mmol, 67%).

^{1}H NMR (300 MHz, $CDCl_3$) δ 8.23 (s, 1H), 8.02 – 7.92 (m, 4H), 7.73 – 7.58 (m, 2H) ppm.

^{13}C{^{1}H} NMR (101 MHz, $CDCl_3$) δ 184.2, 144.9 – 142.2 (m), 140.1 – 137.2 (m), 136.7, 133.4, 132.3, 132.2, 131.8 – 131.6 (m), 130.1, 130.0, 129.6, 128.1, 127.6, 123.4 ppm.

^{19}F NMR (282 MHz, $CDCl_3$) δ -87.9 – -88.1 (m), -141.4 – -141.6 (m) ppm.

See NMR spectra

HRMS (*m/z*): (EI) calc'd for $C_{16}H_7NOF_4$ $[M]^+$: 305.0458, found 305.0460

IR (neat) ν_{max}: 2925, 2856, 1676, 1451, 1294, 962, 740 cm^{-1}

M.P.: 92 °C

TLC: R_f = 0.54 (Hexane/ethyl acetate 9:1)

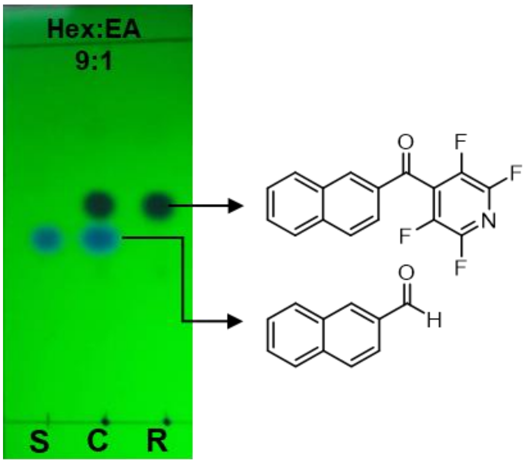

Back to the table

(1*H*-Indol-3-yl)(perfluoropyridin-4-yl)methanone, 3v

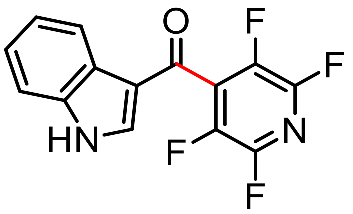

3v

According to **General Procedure A**, 1*H*-indole-3-carbaldehyde **1v** (14.5 mg, 0.1 mmol, 1.0 equiv) and pentafluoropyridine **2a** (16.9 mg, 0.1 mmol, 1.0 equiv) were used to obtain crude residue, which was purified by silica gel column chromatography (Hexane/ethyl acetate 9:1) to afford the **3v** as pale brown solid (15.0 mg, 0.051 mmol, 51%).

^{1}H NMR (300 MHz, $CDCl_3$) δ 10.18 (s, 1H), 8.42 – 8.39 (m, 1H), 7.89 – 7.87 (m, 1H), 7.47 – 7.44 (m, 2H), 7.24 – 7.21 (m, 1H) ppm.

^{13}C{^{1}H} NMR (101 MHz, $CDCl_3$) δ 184.9, 145.5 – 143.1 (m), 138.9 – 135.8 (m), 137.0, 136.5, 128.7 – 128.5 (m), 125.9, 125.0, 124.6, 122.7, 122.5, 111.02 – 110.97 (m) ppm.

^{19}F NMR (282 MHz, $CDCl_3$) δ -86.0 – -86.2 (m), -145.2 – -145.5 (m) ppm.

See NMR spectra

HRMS (*m/z*): (EI) calc'd for $C_{14}H_6N_2OF_4$ $[M]^+$: 294.0411, found 294.0412

IR (neat) ν_{max}: 3118, 3069, 1672, 1550, 1472, 1181, 1043 cm^{-1}

M.P.: 169 °C

TLC: R_f = 0.51(Hexane/ethyl acetate 9:1)

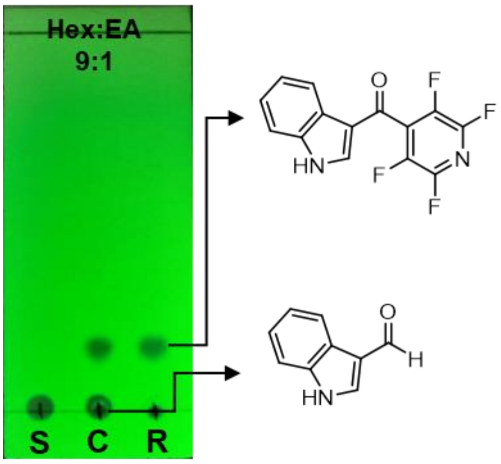

[Back to the table](#)

(Perfluoropyridin-4-yl)(2-phenylquinolin-4-yl)methanone, 3w

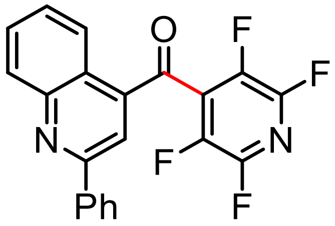

3w

According to **General Procedure A**, 2-phenylquinoline-4-carbaldehyde **1v** (23.3 mg, 0.1 mmol, 1.0 equiv) and pentafluoropyridine **2a** (16.9 mg, 0.1 mmol, 1.0 equiv) were used to obtain crude residue, which was purified by silica gel column chromatography (Hexane/ethyl acetate 15:1) to afford the **3w** yellow solid (19.9 mg, 0.052 mmol, 52%).

^{1}H NMR (300 MHz, $CDCl_3$) δ 8.76 (d, J = 8.5 Hz, 1H), 8.32 (d, J = 8.4 Hz, 1H), 8.07 – 8.05 (m, 2H), 7.91 – 7.85 (m, 2H), 7.75 (t, J = 7.8 Hz, 1H), 7.55 – 7.53 (m, 3H) ppm.

^{13}C{^{1}H} NMR (101 MHz, $CDCl_3$) δ 186.0, 157.1, 149.6, 145.3 – 142.3 (m), 140.0 – 137.3 (m), 138.9, 138.2, 131.6 – 131.3 (m), 130.9, 130.7, 130.2, 129.5, 129.1, 127.5, 124.7, 122.1, 121.2 ppm.

^{19}F NMR (282 MHz, $CDCl_3$) δ -87.0 (ddd, J = 27.0, 26.9, 9.9 Hz), -141.2 (ddd, J = 26.9, 26.7, 10.0 Hz) ppm.

See NMR spectra

HRMS (*m/z*): (ESI) calc'd for $C_{21}H_{11}ON_2F_4$ $[M+H]^+$: 383.0802, found: 383.0801

IR (neat) ν_{max}: 2925, 2854, 1689, 1460, 1264, 966, 770 cm^{-1}

M.P.: 132 ℃

TLC: R_f = 0.51 (Hexane/ethyl acetate 9:1)

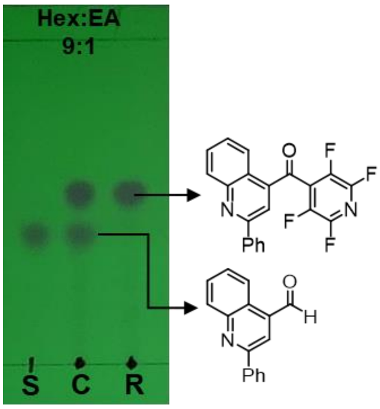

Back to the table

(Perfluoropyridin-4-yl)(thiophen-2-yl)methanone, 3x

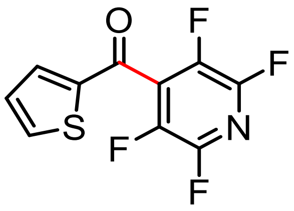

3x

According to **General Procedure A**, thiophene-2-carbaldehyde **1x** (11.2 mg, 0.1 mmol, 1.0 equiv) and pentafluoropyridine **2a** (16.9 mg, 0.1 mmol, 1.0 equiv) were used to obtain crude residue, which was purified by preparative TLC plate (Hexane/ethyl acetate 25:1) to afford the **3x** (11.8 mg, 0.045 mmol, 45%).

^{1}H NMR (300 MHz, $CDCl_3$) δ 7.94 (dd, J = 4.9, 1.0 Hz, 1H), 7.56 (d, J = 3.9 Hz, 1H), 7.24 – 7.21 (m, 1H) ppm.

^{13}C{^{1}H} NMR (101 MHz, $CDCl_3$) δ 175.5, 138.3, 136.6, 129.0 ppm.

^{19}F NMR (282 MHz, $CDCl_3$) δ -87.6 – -87.9 (m), -141.3 – -141.5 (m) ppm.

See NMR spectra

HRMS (*m/z*): (EI) calc'd for $C_{10}H_3F_4NOS$ $[M]^+$: 260.9868, found 260.9866

IR (neat) ν_{max}: 2956, 2917, 2849, 1662, 1463, 1410, 1300, 797 cm^{-1}

M.P.: 54 °C

TLC: R_f = 0.35 (Hexane/ethyl acetate 9:1)

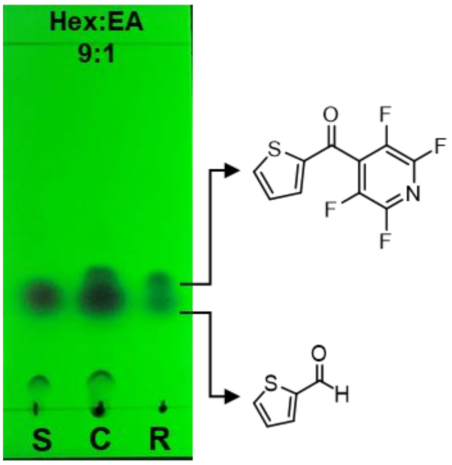

Back to the table

(3-Bromothiophen-2-yl)(perfluoropyridin-4-yl)methanone, 3y

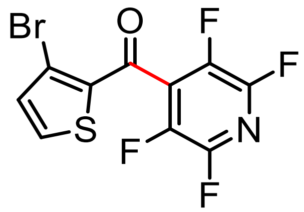

3y

According to **General Procedure A**, 3-bromothiophene-2-carbaldehyde **1y** (19.1 mg, 0.1 mmol, 1.0 equiv) and pentafluoropyridine **2a** (16.9 mg, 0.1 mmol, 1.0 equiv) were used to obtain crude residue, which was purified by silica gel column chromatography (Hexane/ethyl acetate 9:1) to afford the **3y** white solid (25.8 mg, 0.076 mmol, 76%).

^{1}H NMR (300 MHz, $CDCl_3$) δ 7.81 (d, J = 1.4 Hz, 1H), 7.45 (d, J = 1.2 Hz, 1H) ppm.

^{13}C{^{1}H} NMR (101 MHz, $CDCl_3$) δ 174.8, 145.0 – 142.2 (m), 141.8, 139.9 – 136.9 (m), 138.1, 135.2, 130.2 (t, J = 18.8 Hz), 112.2 ppm.

^{19}F NMR (282 MHz, $CDCl_3$) δ -87.1 (ddd, J = 27.7, 27.6, 12.0 Hz), -141.0 – -141.2 (m) ppm.

See NMR spectra

HRMS (*m/z*): (EI) calc'd for $C_{10}H_2NOF_4SBr$ $[M]^+$: 338.8971, found 338.8971

IR (neat) ν_{max}: 3106, 1660, 1458, 1388, 1296, 1188, 966, 781, 694 cm^{-1}

M.P.: 84 °C

TLC: R_f = 0.61 (Hexane/ethyl acetate 9:1)

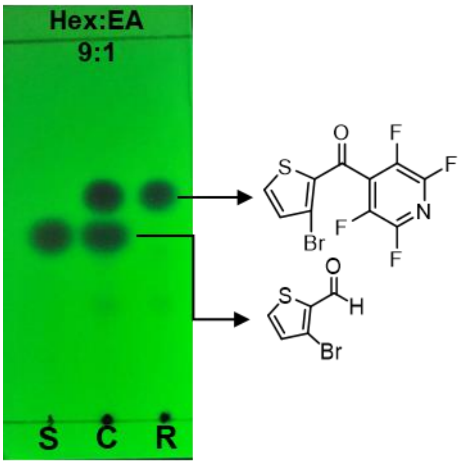

Back to the table

Phenyl(2,3,5,6-tetrafluoro-4-(trifluoromethyl)phenyl)methanone, 4a

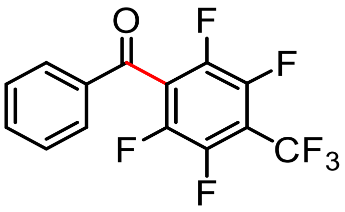

4a

According to **General Procedure A**, benzaldehyde **1a** (10.6 mg, 0.1 mmol, 1.0 equiv) and 1,2,3,4,5-pentafluoro-6-(trifluoromethyl)benzene **2b** (23.6 mg, 0.1 mmol, 1.0 equiv) were used to obtain crude residue, which was purified by silica gel column chromatography (Hexane) to afford the **4a** as colorless liquid (27.6 mg, 0.086 mmol, 86%).

^{1}H NMR (400 MHz, $CDCl_3$) δ 7.86 (d, J = 7.8 Hz, 2H), 7.72 (t, J = 7.3 Hz, 1H), 7.55 (t, J = 7.4 Hz, 2H) ppm.

^{13}C{^{1}H} NMR (101 MHz, $CDCl_3$) δ 184.7, 145.6 – 142.2 (m), 135.5, 135.2, 129.7, 129.3, 122.7 – 121.9 (m) ppm.

^{19}F NMR (376 MHz, $CDCl_3$) δ -56.3 – -56.4 (m), -138.0 – -138.1 (m), -138.27 – -138.30 (m) ppm.

See NMR spectra

HRMS (*m/z*): (EI) calc'd for $C_{14}H_5F_7O$ $[M]^+$: 322.0220, found 322.0223

IR (neat) ν_{max}: 2925, 2853, 1996, 1680, 1482, 1333, 1150, 990, 715 cm^{-1}

TLC: R_f = 0.56 (Hexane/ethyl acetate 9:1)

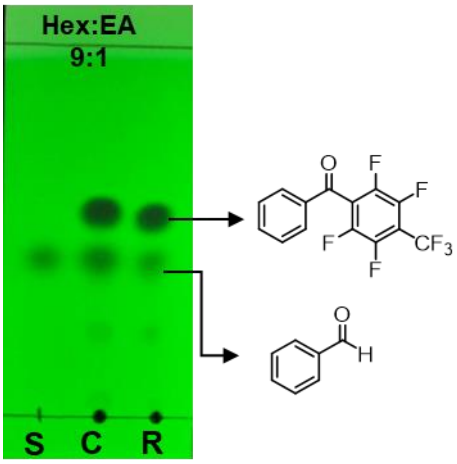

[Back to the table]

4-Benzoyl-2,3,5,6-tetrafluorobenzonitrile, 4b

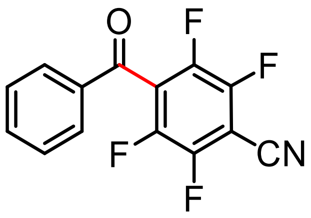

4b

According to **General Procedure A**, benzaldehyde **1a** (10.6 mg, 0.1 mmol, 1.0 equiv) and 2,3,4,5,6-pentafluorobenzonitrile **2c** (19.3 mg, 0.1 mmol, 1.0 equiv) were used to obtain crude residue, which was purified by silica gel column chromatography (Hexane) to afford the **4b** as colorless oil (15.1 mg, 0.054 mmol, 54%).

^{1}H NMR (300 MHz, $CDCl_3$) δ 7.85 – 7.82 (m, 2H), 7.75 – 7.70 (m, 1H), 7.56 (t, J = 7.8 Hz, 2H) ppm.

^{13}C{^{1}H} NMR (101 MHz, $CDCl_3$) δ 184.1, 148.4 – 145.8 (m), 144.5 – 141.9 (m), 135.7, 135.0, 129.7, 129.3, 124.9 – 124.5 (m), 106.9 ppm.

^{19}F NMR (282 MHz, $CDCl_3$) δ -129.9 – -130.1 (m), -137.2 – -137.3 (m) ppm.

See NMR spectra

HRMS (*m/z*): (EI) calc'd for $C_{14}H_5NOF_4$ $[M]^+$: 279.0302, found 279.0299

IR (neat) ν_{max}**:** 3055, 1684, 1490, 1323, 1265, 992, 737 cm^{-1}

TLC: R_f = 0.36 (Hexane/ethyl acetate 9:1)

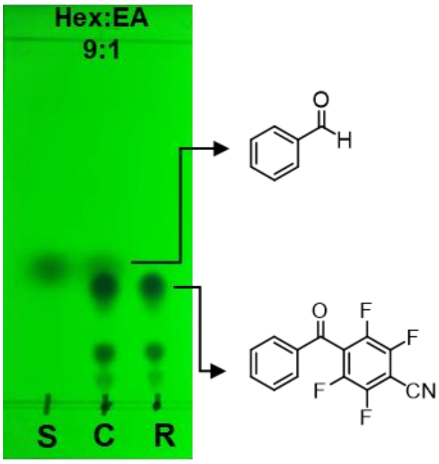

[Back to the table](#)

1-(4-Benzoyl-2,3,5,6-tetrafluorophenyl)ethan-1-one, 4c

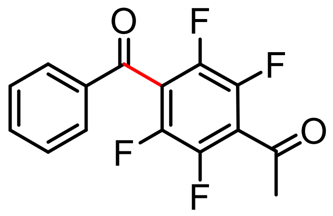

4c

According to **General Procedure A**, benzaldehyde **1a** (10.6 mg, 0.1 mmol, 1.0 equiv) and 1-(perfluorophenyl)ethan-1-one **2d** (21.0 mg, 0.1 mmol, 1.0 equiv) were used to obtain crude residue, which was purified by silica gel column chromatography (Hexane) to afford the **4c** as colorless oil (26.0 mg, 0.088 mmol, 88%).

^{1}H NMR (400 MHz, TMS) δ 7.86 (d, J = 7.2 Hz, 2H), 7.72 – 7.68 (m, 1H), 7.54 (t, J = 7.8 Hz, 2H), 2.68 (s, 3H).

^{13}C{^{1}H} NMR (101 MHz, $CDCl_3$) δ 191.6, 185.4, 144.7 (ddt, J = 37.5, 15.0, 5.0), 142.2 (ddt, J = 34.7, 15.2, 5.1), 135.5, 135.2, 129.7, 129.1, 121.3 – 120.8 (m), 32.4 ppm.

^{19}F NMR (376 MHz, $CDCl_3$) δ -139.0 – -139.1 (m), -140.0 – -140.1 (m) ppm.

See NMR spectra

HRMS (*m/z*): (EI) calc'd for $C_{15}H_8F_4O_2$ $[M]^+$: 296.0455, found 296.0455

IR (neat) ν_{max}**:** 2925, 1715, 1678, 1471, 1303, 1172, 962, 667 cm^{-1}

TLC: R_f = 0.32 (Hexane/ethyl acetate 9:1)

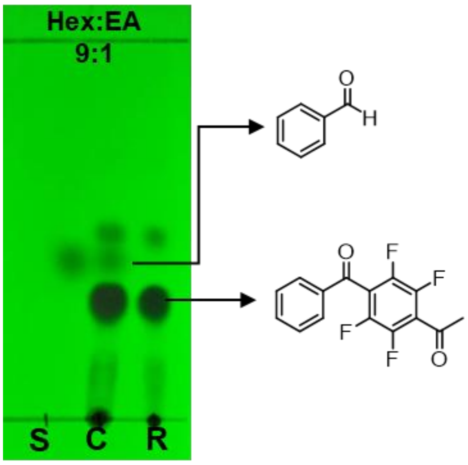

Back to the table

Methyl 4-benzoyl-2,3,5,6-tetrafluorobenzoate, 4d

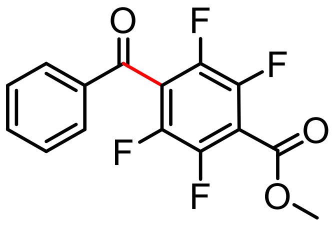

4d

According to **General Procedure A**, benzaldehyde **1a** (10.6 mg, 0.1 mmol, 1.0 equiv) and methyl 2,3,4,5,6-pentafluorobenzoate **2e** (22.6 mg, 0.1 mmol, 1.0 equiv) were used to obtain crude residue, which was purified by silica gel column chromatography (Hexane/ethyl acetate 9:1) to afford the **4d** as yellow oil (24.9 mg, 0.080 mmol, 80%).

^{1}H NMR (300 MHz, $CDCl_3$) δ 7.87 – 7.84 (m, 2H), 7.72 – 7.66 (m, 1H), 7.56 – 7.51 (m, 2H), 4.02 (s, 3H) ppm.

^{13}C{^{1}H} NMR (101 MHz, $CDCl_3$) δ 185.3, 159.6, 145.8 – 143.2 (m), 144.5 – 141.9 (m), 135.5, 135.2, 129.7, 129.1, 121.6 – 121.2 (m), 114.4 – 114.0 (m), 53.5 ppm.

^{19}F NMR (282 MHz, $CDCl_3$) δ -137.5 – -137.7 (m), -139.4 – -139.6 (m) ppm.

See NMR spectra

HRMS (*m/z*): (EI) calc'd for $C_{15}H_8O_3F_4$ $[M]^+$: 312.0404, found 312.0404

IR (neat) ν_{max}: 2956, 1741, 1683, 1474, 1321, 1213, 993, 668 cm^{-1}

TLC: R_f = 0.32 (Hexane/ethyl acetate 9:1)

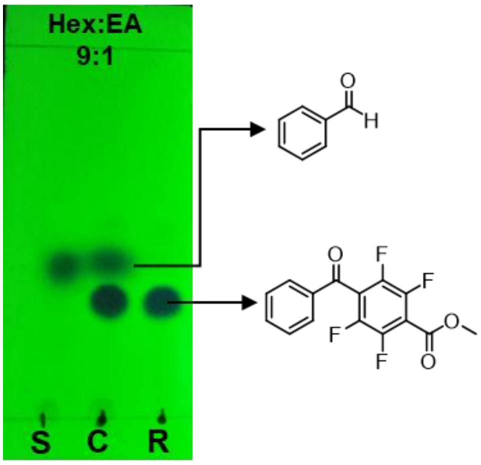

[Back to the table]

(3-Chloro-2,5,6-trifluoropyridin-4-yl)(phenyl)methanone, 4e

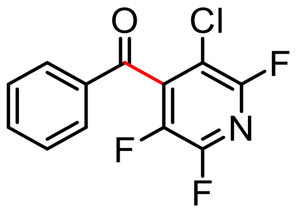

4e

According to **General Procedure A**, benzaldehyde **1a** (10.6 mg, 0.1 mmol, 1.0 equiv) and 3-chloro-2,4,5,6-tetrafluoropyridine **2f** (18.6 mg, 0.1 mmol, 1.0 equiv) were used to obtain crude residue, which was purified by silica gel column chromatography (Hexane) to afford the **4e** as colorless oil (12.8 mg, 0.047 mmol, 47%).

^{1}H NMR (300 MHz, $CDCl_3$) δ 7.84 (d, J = 7.0 Hz, 2H), 7.73 (t, J = 7.5 Hz, 1H,), 7.56 (t, J = 7.9 Hz, 2H) ppm.

^{13}C{^{1}H} NMR (101 MHz, $CDCl_3$) δ 186.4, 152.4 – 150.0 (m), 148.4 – 146.1 (m), 142.2, 142.0, 140.7 – 137.8 (m), 135.7, 134.1, 129.7, 129.4, 110.8 – 109.9 (m) ppm.

^{19}F NMR (282 MHz, $CDCl_3$) δ -72.8 (dd, J = 29.2, 11.7 Hz), -86.0 (dd, J = 22.0, 11.7 Hz), -143.5 (dd, J = 29.1, 22.1 Hz) ppm.

[See NMR spectra]

HRMS (*m/z*): (EI) calc'd for $C_{12}H_5NOF_3Cl$ $[M]^+$: 271.0006, found 271.0008

IR (neat) ν_{max}: 1451, 1378, 1262, 890, 830, 737 cm^{-1}

TLC: R_f = 0.49 (Hexane/ethyl acetate 9:1)

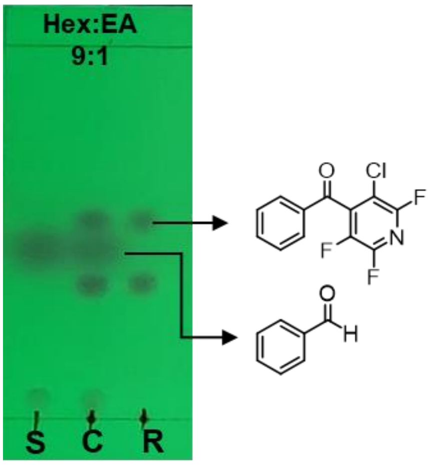

Back to the table

Methyl 4-benzoyl-2,6-difluorobenzoate, 4f

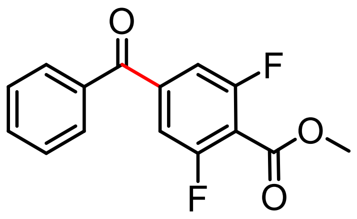

4f

According to **General Procedure A**, benzaldehyde **1a** (10.6 mg, 0.1 mmol, 1.0 equiv) and methyl-2,4,6-trifluorobenzoate **2g** (19.0 mg, 0.1 mmol, 1.0 equiv) were used to obtain crude residue, which was purified by silica TLC plate (Hexane) to afford the **4f** as white solid (7.2 mg, 0.026 mmol, 26%).

^{1}H NMR (400 MHz, $CDCl_3$) δ 7.78 (dt, J = 7.0, 1.4 Hz, 2H), 7.67 – 7.63 (m, 1H), 7.54 – 7.50 (m, 2H), 7.39 – 7.35 (m, 2H), 3.99 (s, 3H) ppm.

^{13}C{^{1}H} NMR (101 MHz, $CDCl_3$) δ 193.1, 161.5 (d, J = 6.1 Hz), 160.1 (d, J = 242.9 Hz), 141.8 – 141.7 (m), 135.8, 133.5, 130.0, 128.7, 114.1, 113.3 (d, J = 24.2 Hz), 53.1 ppm.

^{19}F NMR (282 MHz, $CDCl_3$) δ -108.5 ppm.

See NMR spectra

HRMS (*m/z*): (EI) calc'd for $C_{15}H_{10}F_2O_3$ $[M]^+$: 276.0593, found 276.0596

IR (neat) ν_{max}: 2955, 2924, 2851, 1739, 1421, 1334, 1240, 1045, 725 cm^{-1}

TLC: R_f = 0.18 (Hexane/ethyl acetate 9:1)

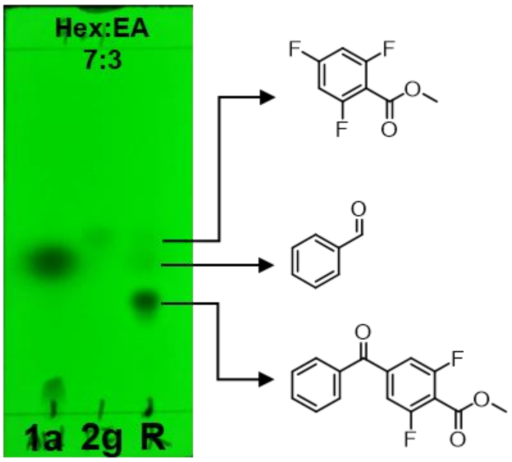

Back to the table

(1*R*,2*S*,5*R*)-2-Isopropyl-5-methylcyclohexyl 4-(perfluoroisonicotinoyl)benzoate, 5a

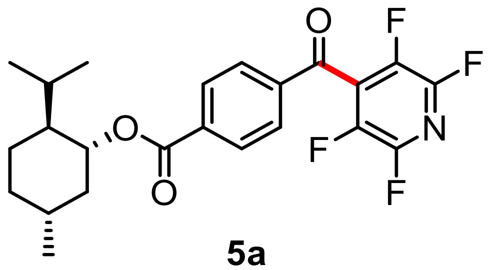

5a

According to **General Procedure A**, (1*R*,2*S*,5*R*)-2-isopropyl-5-methylcyclohexyl 4-formylbenzoate **1z** (28.8 mg, 0.1 mmol, 1.0 equiv) and pentafluoropyridine **2a** (16.9 mg, 0.1 mmol, 1.0 equiv) were used to obtain crude residue, which was purified by silica gel column chromatography (Hexane) to afford the **5a** as colorless liquid (31.0 mg, 0.071 mmol, 71%).

^{1}H NMR (300 MHz, $CDCl_3$) δ 8.20 (d, J = 8.4 Hz, 2H), 7.92 (d, J = 8.2 Hz, 2H), 4.96 (td, J = 10.9, 4.4 Hz, 1H), 2.12 (d, J = 11.8 Hz, 1H), 1.92 (pd, J = 9.1, 2.7 Hz, 1H), 1.77 – 1.70 (m, 2H), 1.63 – 1.53 (m, 2H), 1.25 – 1.06 (m, 2H), 0.99 – 0.90 (m, 7H), 0.79 (d, J = 7.0 Hz, 3H). ppm.

^{13}C{^{1}H} NMR (101 MHz, $CDCl_3$) δ 184.0, 164.5, 144.9 – 142.2 (m), 140.0 – 137.1 (m), 137.4, 136.9, 131.1 – 130.7 (m), 130.3, 129.6, 76.0, 47.2, 40.8, 34.2, 31.4, 26.5, 23.6, 21.9, 20.7, 16.4 ppm.

^{19}F NMR (282 MHz, $CDCl_3$) δ -87.6 (ddd, J = 26.5, 26.4, 9.8 Hz), -141.3 – -141.5 (m) ppm.

See NMR spectra

HRMS (*m/z*): (ESI) calc'd for $C_{23}H_{23}O_3NF_4{}^{23}Na$ $[M+Na]^+$: 460.1506, found: 460.1503

IR (neat) ν_{max}: 2959, 2928, 1714, 1688, 1461, 1270, 1109, 961, 826 cm^{-1}

TLC: R_f = 0.58 (Hexane/ethyl acetate 9:1)

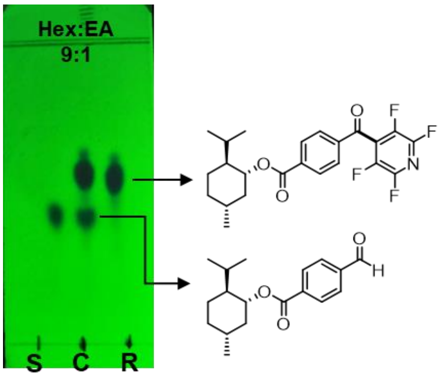

Back to the table

(8*R*,9*S*,13*S*,14*S*)-8,13-Dimethyl-17-oxo-7,8,9,11,12,13,14,15,16,17-decahydro-6*H*-cyclopenta[*a*]phenanthren-3-yl 4-(perfluoroisonicotinoyl)benzoate, 5b

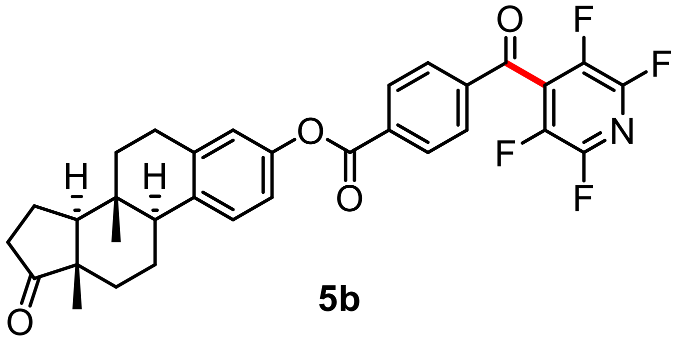

5b

According to **General Procedure A**, (8*R*,9*S*,13*S*,14*S*)-8,13-Dimethyl-17-oxo-7,8,9,11,12,13,14,15,16,17-decahydro-6*H*-cyclopenta[*a*]phenanthren-3-yl 4-formylbenzoate **1aa** (41.7 mg, 0.1 mmol, 1.0 equiv) and pentafluoropyridine **2a** (16.9 mg, 0.1 mmol, 1.0 equiv) were used to obtain crude residue, which was purified by silica gel column chromatography (Hexane) to afford the **5b** as white solid (28.3 mg, 0.050 mmol, 50%).

^{1}H NMR (300 MHz, $CDCl_3$) δ 8.36 (d, J = 8.5 Hz, 2H), 7.99 (d, J = 8.3 Hz, 2H), 7.36 (d, J = 8.5 Hz, 1H), 7.01 – 6.95 (m, 2H), 2.95 (dd, J = 9.0, 4.2 Hz, 2H), 2.56 – 2.41 (m, 3H), 2.21 – 1.96 (m, 5H), 1.66 – 1.45 (m, 7H), 0.93 (s, 3H). ppm.

^{13}C{^{1}H} NMR (101 MHz, $CDCl_3$) δ 220.7, 183.9, 163.9, 148.4, 144.9 – 142.3 (m), 140.0 – 137.0 (m), 138.3, 137.9, 137.9, 135.6, 130.9, 130.7 – 130.5 (m), 129.7, 126.6, 121.4, 118.5, 50.4, 47.9, 44.1, 37.9, 35.8, 34.1, 31.5, 29.4, 26.3, 25.7, 21.5, 13.8 ppm.

^{19}F NMR (282 MHz, $CDCl_3$) δ -87.3 – -87.5 (m), -141.1 – -141.3 (m) ppm.

See NMR spectra

HRMS (*m/z*): (EI) calc'd for: $C_{32}H_{27}NO_4F_4$ $[M]^+$: 565.1871, found 565.1869

IR (neat) ν_{max}: 2932, 2863, 1735, 1687, 1462, 1260, 1075, 962, 908, 731 cm^{-1}

M.P.: 183 °C

TLC: R_f = 0.58 (Hexane/ethyl acetate 9:1)

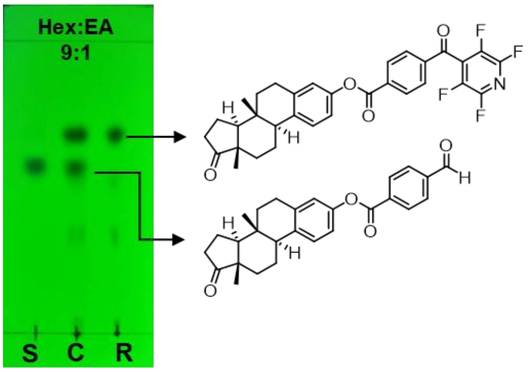

[Back to the table]

(3*S*,8*S*,9*S*,10*R*,13*S*,14*S*,17*S*)-17-Acetyl-8,10,13-trimethyl-2,3,4,7,8,9,10,11,12,13,14,15,16,17-tetradecahydro-1*H*-cyclopenta[*a*]phenanthren-3-yl 4-(perfluoroisonicotinoyl)benzoate, 5c

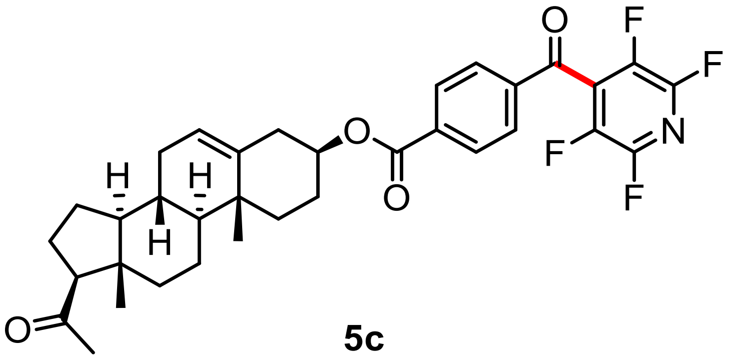

5c

According to **General Procedure A**, (3*S*,8*S*,9*S*,10*R*,13*S*,14*S*,17*S*)-17-acetyl-8,10,13-trimethyl-2,3,4,7,8,9,10,11,12,13,14,15,16,17-tetradecahydro-1*H*-cyclopenta[*a*]phenanthren-3-yl 4-formylbenzoate **1ab** (46.2 mg, 0.1 mmol, 1.0 equiv) and pentafluoropyridine **2a** (16.9 mg, 0.1 mmol, 1.0 equiv) were used to obtain crude residue, which was purified by silica gel column chromatography (Hexane/ethyl acetate 15:1) to afford the **5c** as white solid (30.6 mg, 0.050 mmol, 50%).

^{1}H NMR (400 MHz, $CDCl_3$) δ 8.20 (d, J = 8.6 Hz, 2H), 7.91 (d, J = 8.4 Hz, 2H), 5.43 (dd, J = 5.2, 1.9 Hz, 1H), 4.93 – 4.85 (m, 1H), 2.54 (t, J = 8.9 Hz, 1H), 2.48 (d, J = 7.4 Hz, 2H), 2.22 – 2.16 (m, 1H), 2.12 (s, 3H), 2.05 – 2.00 (m, 2H), 1.94 (dt, J = 13.5, 3.4 Hz, 1H), 1.78 – 1.60 (m, 5H), 1.57 – 1.46 (m, 4H), 1.27 – 1.19 (m, 3H), 1.07 (s, 3H), 1.05 – 1.02 (m, 1H), 0.64 (s, 3H) ppm.

^{13}C{^{1}H} NMR (101 MHz, $CDCl_3$) δ 209.5, 184.0, 164.4, 144.8 – 142.3 (m), 140.0 – 137.0 (m), 139.3, 137.4, 136.8, 130.8 (t, J = 53.2 Hz), 130.4, 129.5, 122.8, 122.8, 75.5, 63.6, 56.8, 49.8, 43.9, 38.7, 38.0, 36.9, 36.6, 31.8, 31.8, 31.5, 27.7, 24.5, 22.8, 21.0, 19.3, 13.2 ppm.

^{19}F NMR (282 MHz, $CDCl_3$) δ -87.3 – -87.5 (m), -141.1 – -141.3 (m) ppm.

[See NMR spectra]

HRMS (*m/z*): (EI) calc'd for $C_{34}H_{35}NO_4F_4$ $[M]^+$: 597.2497, found: 597.2494

IR (neat) ν_{max}: 2939, 2889, 1721, 1690, 1458, 1280, 1116, 964, 828, 728 cm^{-1}

M.P.: 193 °C

TLC: R_f = 0.24 (Hexane/ethyl acetate 9:1)

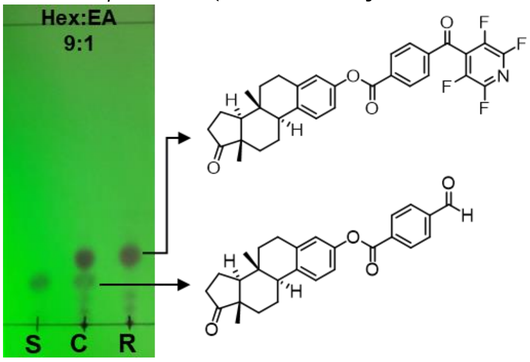

Back to the table

(3*S*,8*S*,9*S*,10*R*,13*R*,14*S*,17*R*)-10,13-Dimethyl-17-((*R*)-6-methylheptan-2-yl) 2,3,4,7,8,9,10,11,12,13,14,15,16,17-tetradecahydro-1*H*-cyclopenta[*a*]phenanthren-3-yl 4-(perfluoroisonicotinoyl)benzoate, 5d

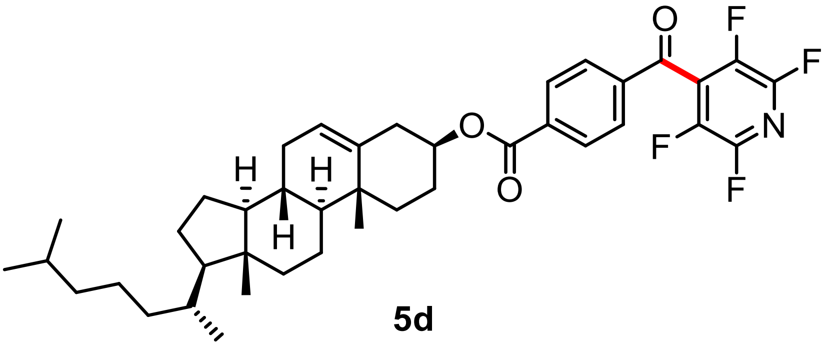

5d

According to **General Procedure A**, (3*S*,8*S*,9*S*,10*R*,13*R*,14*S*,17*R*)-10,13-dimethyl-17-((*R*)-6-methylheptan-2-yl)-2,3,4,7,8,9,10,11,12,13,14,15,16,17-tetradecahydro-1*H*-cyclopenta[*a*]phenanthren-3-yl 4-formylbenzoate **1ac** (51.8 mg, 0.1 mmol, 1.0 equiv) and pentafluoropyridine **2a** (16.9 mg, 0.1 mmol, 1.0 equiv) were used to obtain crude residue, which was purified by silica gel column chromatography (Hexane) to afford the **5d** as white solid (60.7 mg, 0.091 mmol, 91%).

^{1}H NMR (300 MHz, $CDCl_3$) δ 8.20 (d, J = 8.5 Hz, 2H), 7.91 (d, J = 8.2 Hz, 2H), 5.43 (d, J = 4.3 Hz, 1H), 4.94 – 4.84 (m, 1H), 2.47 (d, J = 7.5 Hz, 2H), 2.05 – 1.73 (m, 6H), 1.70 – 1.52 (m, 5H), 1.45 – 1.07 (m, 13H), 1.07 (s, 3H), 1.04 – 0.96 (m, 3H), 0.92 (d, J = 6.4 Hz, 2H), 0.86 (dd, J = 6.6, 1.3 Hz, 6H), 0.69 (s, 3H) ppm.

^{13}C{^{1}H} NMR (101 MHz, $CDCl_3$) δ 184.0, 164.5, 144.9 – 142.2 (m), 139.7 – 137.0 (m), 139.3, 137.4, 136.9, 131.1 – 130.7 (m), 130.4, 129.5, 123.2, 75.7, 56.7, 56.1, 50.0, 42.3, 39.7, 39.5, 38.1, 37.0, 36.6, 36.2, 35.8, 31.9, 31.9, 28.2, 28.0, 27.8, 24.3, 23.8, 22.8, 22.5, 21.0, 19.3, 18.7, 11.9 ppm.

^{19}F NMR (282 MHz, $CDCl_3$) δ -87.4 – -87.7 (m), -141.2 – -141.4 (m) ppm.

See NMR spectra

HRMS (*m/z*): (EI) calc'd for $C_{40}H_{49}NO_3F_4$ $[M]^+$: 667.3643, found: 667.3642

IR (neat) ν_{max}: 2935, 2867, 1721, 1458, 1279, 1108, 964, 829, 750 cm^{-1}

M.P.: 182 °C

TLC: R_f = 0.63 (Hexane/ethyl acetate 9:1)

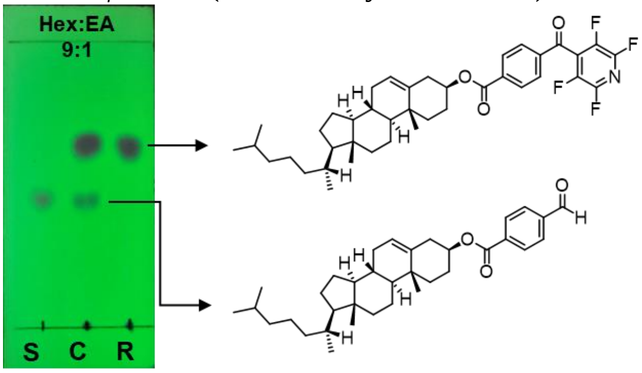

Back to the table

(1*R*,2*S*,5*R*)-2-Isopropyl-5-methylcyclohexyl-4-benzoyl-2,3,5,6-tetrafluorobenzoate, 5e

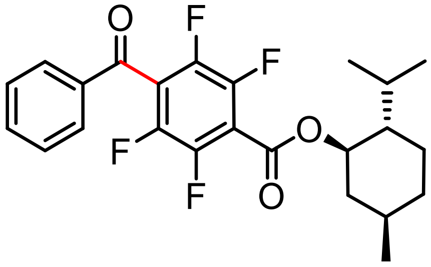

5e

According to **General Procedure A**, benzaldehyde **1a** (10.6 mg, 0.1 mmol, 1.0 equiv) and (1*R*,2*S*,5*R*)-2-isopropyl-5-methylcyclohexyl-2,3,4,5,6-pentafluorobenzoate **2h** (35.0 mg, 0.1 mmol, 1.0 equiv) were used to obtain crude residue, which was purified by silica gel column chromatography (Hexane/ethyl acetate 9:1) to afford the **5e** as pale-yellow oil (34.9 mg, 0.080 mmol, 80 %).

^{1}H NMR (300 MHz, $CDCl_3$) δ 7.90 – 7.82 (m, 2H), 7.72 – 7.65 (m, 1H), 7.57 – 7.48 (m, 2H), 5.03 (td, *J* = 10.9, 4.4 Hz, 1H), 2.19 (dd, *J* = 11.8, 3.0 Hz, 1H), 2.08 – 1.95 (m, 1H), 1.80 – 1.68 (m, 2H), 1.59 – 1.45 (m, 1H), 1.30 – 1.03 (m, 3H), 0.99 – 0.89 (m, 6H), 0.87 – 0.79 (m, 3H) ppm.

^{13}C{^{1}H} NMR (101 MHz, $CDCl_3$) δ 185.4, 158.8, 145.4 – 144.3 (m), 142.9 – 141.8 (m), 135.6, 135.1, 129.7, 129.1, 120.8 (t, *J* = 57.5), 115.3 (t, *J* = 49.2), 78.0, 46.9, 40.6, 34.0, 31.5, 25.9, 23.1, 21.9, 20.8, 15.9 ppm.

^{19}F NMR (282 MHz, $CDCl_3$) δ -138.3 – -138.5 (m), -138.5 – -139.7 (m) ppm.

See NMR spectra

HRMS (*m/z*): (EI) calc'd for $C_{24}H_{24}F_4O_3$: 436.1656, found 436.1653

IR (neat) ν_{max}: 2957, 2927, 2871, 1731, 1475, 1309, 1208, 990 cm^{-1}

TLC: R_f = 0.56 (Hexane/ethyl acetate 9:1)

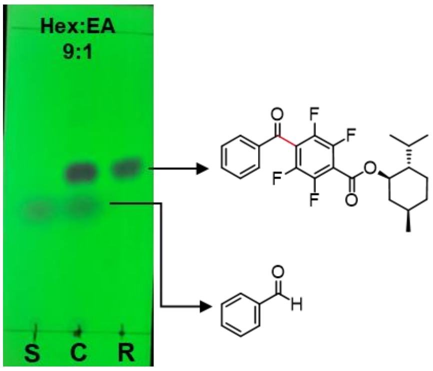

Back to the table

(3*S*,8*S*,9*S*,10*R*,13*R*,14*S*)-10,13-Dimethyl-17-((*R*)-6-methylheptan-2-yl)-2,3,4,7,8,9,10,11,12,13,14,15,16,17-tetradecahydro-1*H*-cyclopenta[*a*]phenanthren-3-yl 4-benzoyl-2,3,5,6-tetrafluorobenzoate, 5f

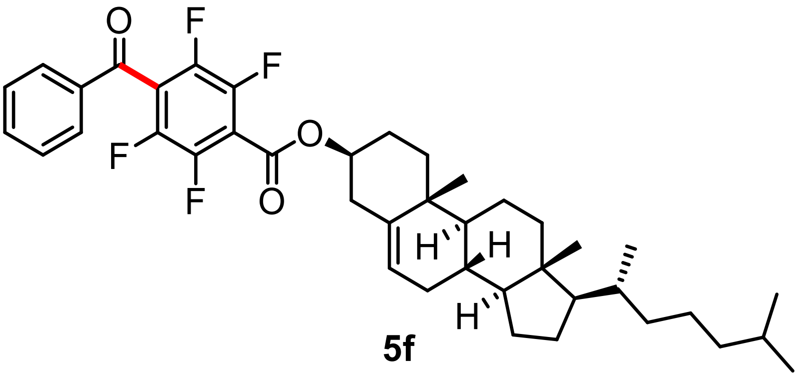

5f

According to **General Procedure A**, benzaldehyde **1a** (10.6 mg, 0.1 mmol, 1.0 equiv) and (3*S*,8*S*,9*S*,10*R*,13*R*,14*S*)-10,13-Dimethyl-17-((*R*)-6-methylheptan-2-yl)-2,3,4,7,8,9,10,11,12,13,14,15,16,17-tetradecahydro-1*H*-cyclopenta[*a*]phenanthren-3-yl 2,3,4,5,6-pentafluorobenzoate **2i** compound with methane (1:1) (59.7 mg, 0.1 mmol, 1.0 equiv) were used to obtain crude residue, which was purified by silica gel column chromatography (Hexane/ethyl acetate 9:1) to afford the **5f** as white solid (61.9 mg, 0.093 mmol, 93%).

^{1}H NMR (300 MHz, $CDCl_3$) δ 7.86 (d, J = 7.7 Hz, 2H), 7.69 (t, J = 7.4 Hz, 1H), 7.53 (t, J = 7.6 Hz, 2H), 5.46 (d, J = 5.0 Hz, 1H), 5.02 – 4.91 (m, 1H), 2.50 (d, J = 8.0 Hz, 2H), 2.06 – 1.69 (m, 7H), 1.61 – 1.45 (m, 12H), 1.36 – 1.09 (m, 5H), 1.06 (s, 3H), 1.02 – 0.97 (m, 3H), 0.92 (d, J = 6.5 Hz, 2H), 0.87 (dd, J = 6.6, 1.4 Hz, 6H), 0.69 (s, 3H) ppm

^{13}C{^{1}H} NMR (101 MHz, $CDCl_3$) δ 185.4, 158.5, 158.4, 145.4 – 142.9 (m), 144.5 – 141.6 (m), 144.2, 139.0, 135.6, 135.2, 129.7, 129.1, 123.4, 123.4, 121.2 – 121.0 (m), 115.1 – 115.0 (m), 56.7, 56.1, 50.0, 42.3, 39.7, 39.5, 37.9, 36.9, 36.6, 36.2, 35.8, 31.9, 31.8, 28.2, 28.0, 27.6, 24.3, 23.8, 22.8, 22.5, 21.0, 19.3, 18.7, 11.8 ppm.

^{19}F NMR (282 MHz, $CDCl_3$) δ -138.2 – -138.30, -139.6 – -139.7 ppm.

See NMR spectra

HRMS (*m/z*): (EI) calc'd for $C_{41}H_{50}F_4O_3$ $[M]^+$: 666.3691, found 666.3691

IR (neat) ν_{max}: 2935, 2869, 1733, 1475, 1323, 1232, 1208, 992, 733 cm^{-1}

M.P.: 116 °C

TLC: R_f = 0.61 (Hexane/ethyl acetate 9:1)

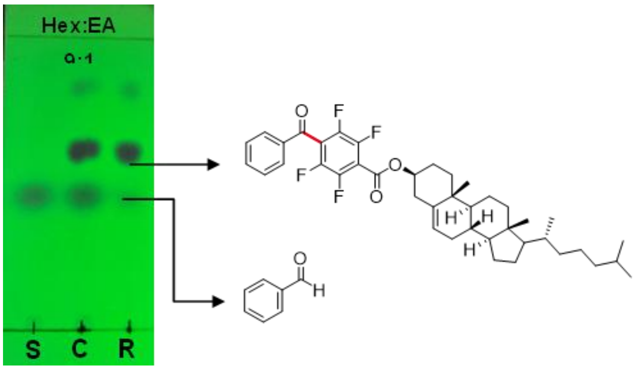

Back to the table

(3*S*,8*S*,9*S*,10*R*,13*S*,14*S*,17*S*)-17-Acetyl-8,10,13-trimethyl-2,3,4,7,8,9,10,11,12,13,14,15,16,17-tetradecahydro-1*H*-cyclopenta[*a*]phenanthren-3-yl 4-benzoyl-2,3,5,6-tetrafluorobenzoate, 5g

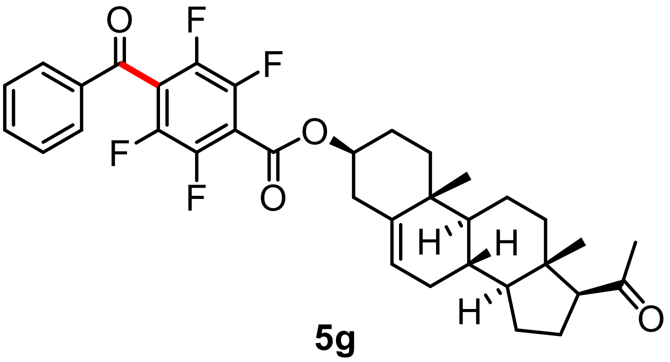

5g

According to **General Procedure A**, benzaldehyde **1a** (10.6 mg, 0.1 mmol, 1.0 equiv) and (3*S*,8*S*,9*S*,10*R*,13*S*,14*S*,17*S*)-17-Acetyl-8,10,13-trimethyl-2,3,4,7,8,9,10,11,12,13,14,15,16,17-tetradecahydro-1*H*-cyclopenta[*a*]phenanthren-3-yl 2,3,4,5,6-pentafluorobenzoate **2j** (52.4 mg, 0.1 mmol, 1.0 equiv) were used to obtain crude residue, which was purified by silica gel column chromatography (Hexane/ethyl acetate 9:1) to afford the **5g** as pale-yellow oil (32.9 mg, 0.055 mmol, 55 %).

^{1}H NMR (300 MHz, $CDCl_3$) δ 7.86 – 7.83 (m, 2H), 7.71 – 7.65 (m, 1H), 7.55 – 7.50 (m, 2H), 5.46 – 5.44 (m, 1H), 5.01 – 4.90 (m, 1H), 2.57 – 2.47, (m, 3H), 2.20 – 1.91 (m, 8H), 1.83 – 1.41 (m, 8H), 1.26 – 1.16 (m, 3H), 1.12 – 1.05 (m, 4H), 0.64 (s, 3H) ppm.

^{13}C{^{1}H} NMR (101 MHz, $CDCl_3$) δ 209.5, 185.4, 158.5, 145.6 – 142.9 (m), 141.8 – 138.0 (m), 139.0, 135.6, 135.2, 129.7, 129.1, 123.1, 121.2 – 120.8 (m), 115.4 – 114.9 (m), 63.6, 56.8, 49.8, 43.9, 38.7, 37.8, 36.9, 36.6, 31.8, 31.5, 27.6, 24.4, 22.8, 21.0, 19.3, 13.2 ppm.

^{19}F NMR (282 MHz, $CDCl_3$) δ -138.1 – -138.3 (m), -139.5 – -139.7 (m) ppm.

See NMR spectra

HRMS (*m/z*): (EI) calc'd for $C_{35}H_{36}O_4F_4$ $[M]^+$: 596.2544, found 596.2546

IR (neat) ν_{max}: 2939, 2853, 2252, 1597, 1475, 1322, 1208, 1051, 993 cm^{-1}

M.P.: 138 °C

TLC: R_f = 0.20 (Hexane/ethyl acetate 9:1)

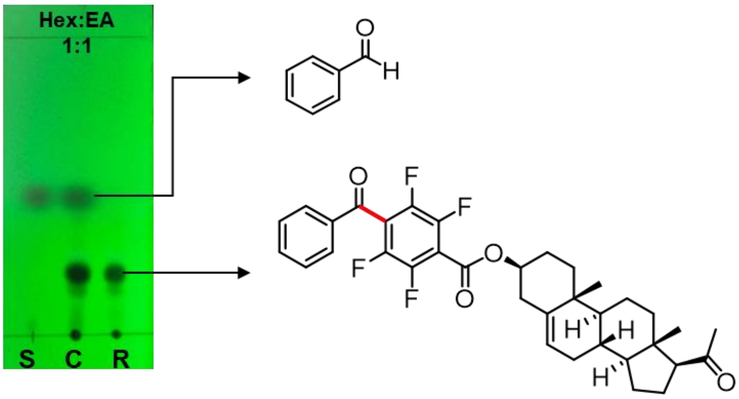

Back to the contents

2.7 Large-scale syntheses of NHC-Catalyzed S_NAr reaction

Gram-scale synthesis of 3a

According to **General Procedure B**, benzaldehyde **1a** (0.74 g, 7 mmol, 1.0 equiv) and pentafluoropyridine **2a** (1.30 g, 7.7 mmol, 1.1 equiv) were used to obtain crude residue, which was purified by silica gel column chromatography (Hexane) to afford the **3a** as pale-yellow liquid (1.1810 g, 4.63 mmol, 66%).

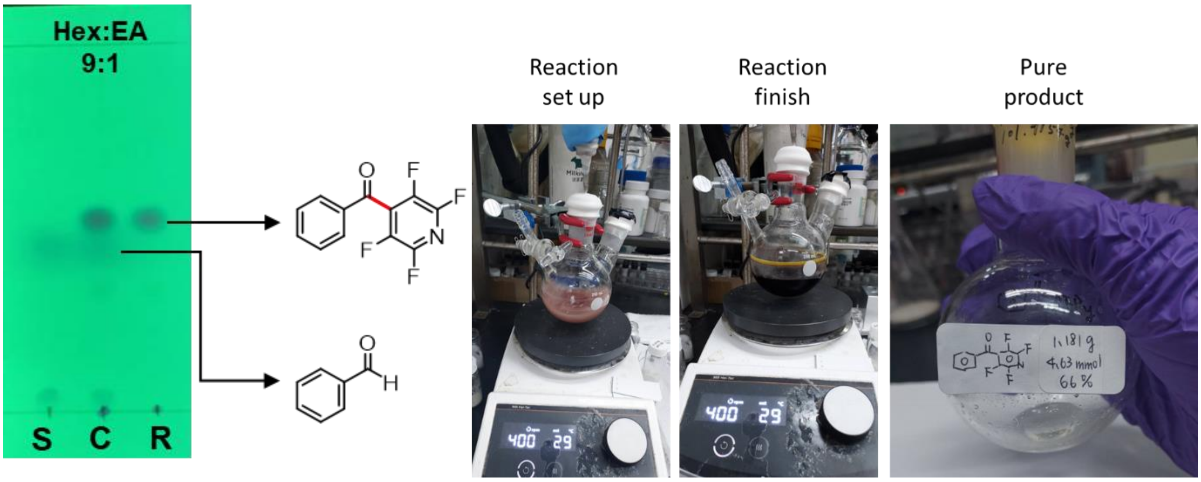

Gram-scale synthesis of 3s

According to **General Procedure B**, 3-bromo-4-fluorobenzaldehyde **1s** (1.41 g, 7 mmol, 1.0 equiv) and pentafluoropyridine **2a** (1.3 g, 7.7 mmol, 1.1 equiv) were used to obtain crude residue, which was purified by silica gel column chromatography (Hexane) to afford the **3s** as pale-yellow liquid (1.7438 g, 4.95 mmol, 71%).

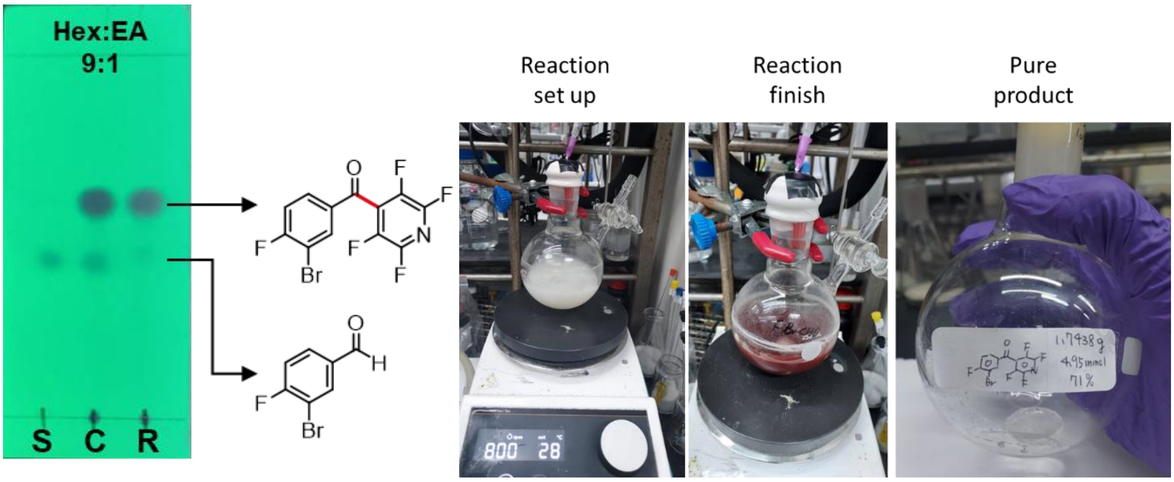

Back to the contents

3. Synthesis of bioisostere

3.1 Procedure of synthesis

Synthesis of compound 6

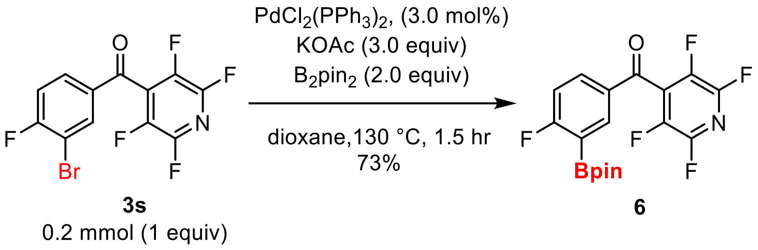

In the nitrogen-filled glovebox, an oven-dried tube equipped with a stirring bar was added bis(triphenylphosphine)palladium chloride (4.2 mg, 0.006 mmol, 3.0 mol%), potassium acetate (58.9 mg, 0.6 mmol, 3.0 equiv) and bis(pinacolato)diboron (101.6 mg, 0.4 mmol, 2.0 equiv). The tube was added 0.5 mL preparative 0.2 M solution **3s** (70.4 mg, 0.2 mmol, 1.0 equiv) in dioxane followed by additional 0.5 mL dioxane. Then the tube was sealed and moved out, stirred at 130 ℃ for further 1.5 hr. After the reaction completed, the crude was purified by silica gel column chromatography (the height of packing silica gel: 8.0 cm, Hexane/ethyl acetate 25:1) afford **6** (58.3 mg, 0.146 mmol, 73%).

^{1}H NMR (300 MHz, $CDCl_3$) δ 8.24 (dd, J = 5.4, 2.5 Hz, 1H), 7.95 (ddd, J = 8.1, 5.0, 2.5 Hz, 1H), 7.20 (t, J = 8.6 Hz, 1H), 1.37 (s, 12H) ppm.

^{13}C{^{1}H} NMR (101 MHz, $CDCl_3$) δ 182.9, 171.4 (d, J = 264.8 Hz), 144.9 – 142.1 (m), 140.0 – 137.0 (m), 139.5 (d, J = 10.5 Hz), 135.1 (d, J = 11.1 Hz), 131.2 (t, J = 18.1 Hz), 130.8 (d, J = 2.6 Hz), 116.9 (d, J = 25.3 Hz), 84.6, 24.8 ppm.

^{19}F NMR (282 MHz, $CDCl_3$) δ -87.8 – -88.0 (m), -89.1, -141.4 – -141.6 (m) ppm.

See NMR spectra

HRMS (*m/z*): (EI) calc'd for $C_{18}H_{15}BNO_3F_5$ $[M]^+$: 399.1060, found: 399.1062

IR (neat) ν_{max}: 2980, 2957, 1607, 1464, 1352, 1286, 1143, 853 cm^{-1}

TLC: R_f = 0.25 (Hexane/ethyl acetate 9:1)

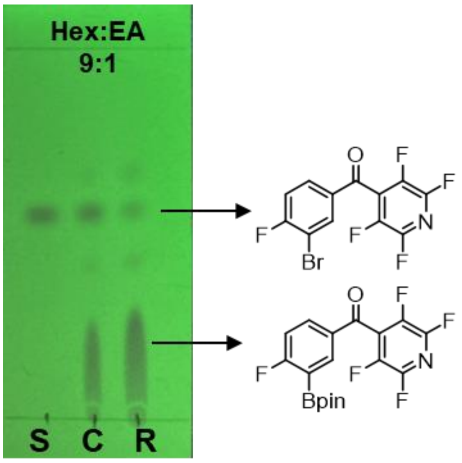

Back to the contents

Synthesis of compound 7

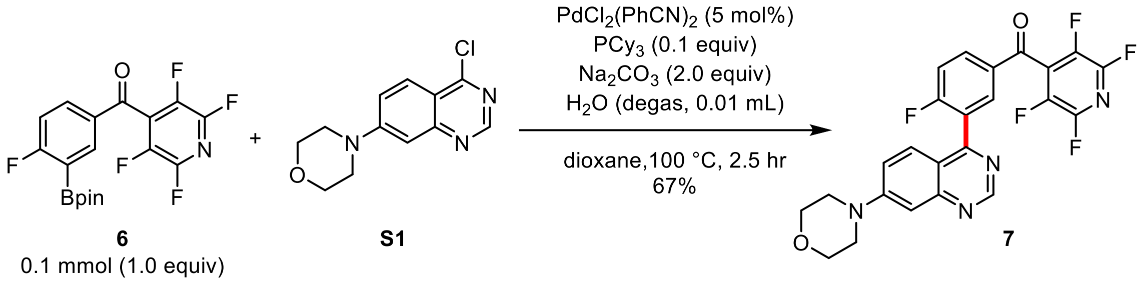

In the nitrogen-filled glovebox, an oven-dried tube (5.0 mL) equipped with a stirring bar was added bis(benzonitrile)palladium dichloride (1.92 mg, 0.005 mmol, 5.0 mol%), tricyclohexylphosphine (2.8 mg, 0.01 mmol, 10.0 mol%), sodium carbonate (21.2 mg, 0.2 mmol, 2.0 equiv) and **S1** (25.0 mg, 0.1 mmol, 1.0 equiv). The tube was added 0.5 mL preparative 0.2 M solution **6** (0.1 mmol, 1.0 equiv) in dioxane followed by additional 0.5 mL dioxane. Then the tube was sealed, moved out and added 0.01 mL degassed water then stirred at 100 ℃ for further 2.5 hr. After the reaction completed, the crude was washed by ethyl acetate and water. Organic mixture was purified by preparative TLC plate (Hexane/ethyl acetate 7:3) to afford **7** (32.6 mg, 0.067 mmol, 67%).

^{1}H NMR (300 MHz, DMSO-d_6) δ 9.11 (s, 1H), 8.37 – 8.32 (m, 2H), 7.74 (t, J = 9.0 Hz, 1H), 7.53 (s, 2H), 7.21 (s, 1H), 3.77 (t, J = 4.7 Hz, 4H), 3.44 (t, J = 4.9 Hz, 4H) ppm.

^{13}C{^{1}H} NMR (101 MHz, DMSO-d_6) δ 183.3, 163.6 (d, J = 259.6 Hz), 159.7, 154.8, 154.7, 152.1, 144.4 – 141.7 (m), 140.1 – 137.2 (m), 134.2 (d, J = 5.2 Hz), 133.9 (d, J = 10.2 Hz), 131.4, 129.9 (t, J = 18.8 Hz), 127.1, 126.3 (d, J = 16.9 Hz), 119.3, 118.0 (d, J = 23.0 Hz), 116.8, 106.1, 65.8, 46.9 ppm.

^{19}F NMR (282 MHz, DMSO-d_6) δ -90.0 – -90.3 (m), -102.8, -142.7 – -143.0 (m) ppm.

See NMR spectra

HRMS (*m/z*): (ESI) calc'd for $C_{24}H_{16}O_2N_4F_5$ $[M+H]^+$: 487.1188, found 487.1186

IR (neat) ν_{max}: 2969, 2955, 1613, 1464, 1269, 968 cm^{-1}

TLC: R_f = 0.50 (Hexane/ethyl acetate 1:1)

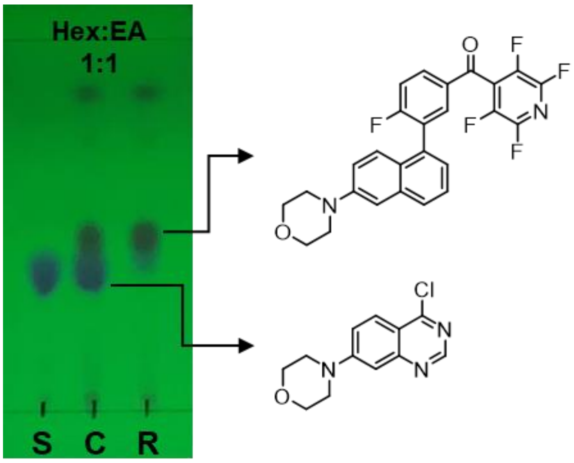

[Back to the contents]

Synthesis of PF-D1

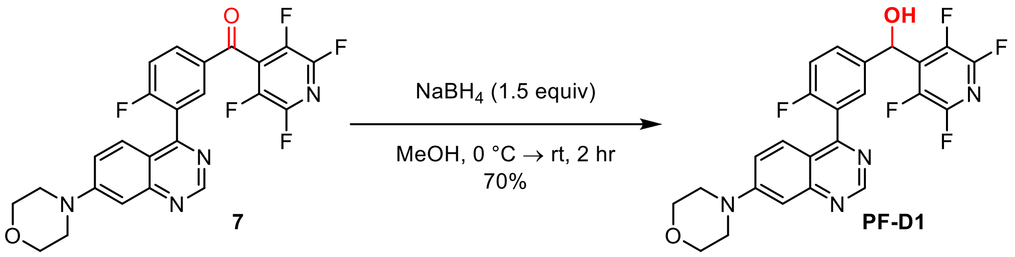

Under the nitrogen gas, an oven-dried tube equipped with a stirring bar was added sodium borohydride (5.7 mg, 0.15 mmol, 1.5 equiv). At 0 ℃, the tube was added 0.5 mL preparative 0.2 M solution **7** (48.6 mg, 0.1 mmol, 0.1 equiv) in methanol followed by additional 0.5 mL methanol. The mixture was stirred at room temperature for 2 hr. After the reaction completed, the crude was washed by ethyl acetate and water. Organic mixture was purified by preparative TLC plate (Hexane/ethyl acetate 7:3) to afford **PF-D1** as a yellow solid (34.0 mg, 0.070 mmol, 70%).

^{1}H NMR (300 MHz, DMSO-d_6) δ 9.10 (s, 1H), 7.77 – 7.67 (m, 2H), 7.52 – 7.42 (m, 4H), 7.20 (s, 1H), 6.92 (s, 1H), 6.28 (s, 1H), 3.77 (t, J = 4.7 Hz, 4H), 3.44 (t, J = 4.8 Hz, 4H) ppm.

^{13}C{^{1}H} NMR (101 MHz, DMSO-d_6) δ 161.1, 158.5 (d, J = 247.5 Hz), 155.0, 154.5, 152.1, 144.3 – 141.6 (m), 141.0 – 138.1 (m), 137.1 (d, J = 2.1 Hz), 136.4 (t, J = 13.6 Hz), 129.2 (d, J = 8.7 Hz), 128.8, 127.2 (d, J = 1.6 Hz), 124.6 (d, J = 15.9 Hz), 119.0, 116.7, 116.1 (d, J = 22.3 Hz), 106.3, 65.8, 64.9, 46.9 ppm.

^{19}F NMR (282 MHz, $CDCl_3$) δ -92.2 – -92.5 (m), -116.2, -143.7, – -143.9 (m) ppm.

[See NMR spectra]

HRMS (*m/z*): (ESI) calc'd for $C_{24}H_{18}O_2N_4F_5$ $[M+H]^+$: 489.1344, found 489.1341

IR (neat) ν_{max}: 3222, 2956, 2922, 2852, 1612, 1468, 1235, 1124, 948, 824 cm^{-1}

M.P.: 128 °C

TLC: R_f = 0.31 (Hexane/ethyl acetate 9:1)

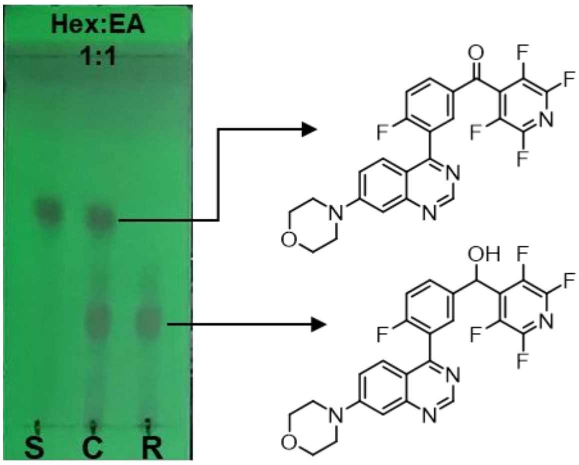

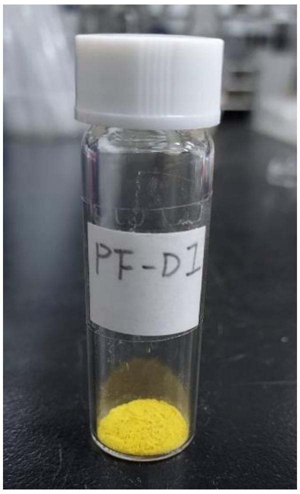

3.2 Procedure comparison

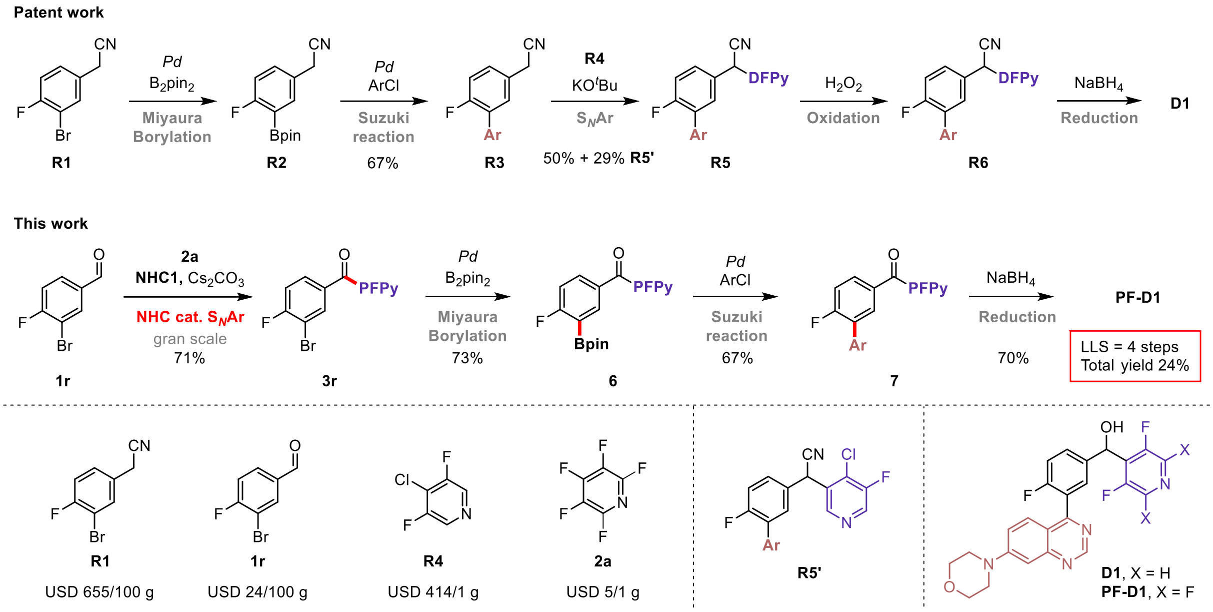

Compared to the existing literature methods[3], our protocol offers several advantages, such as lower cost (Price information comes from the official selling price of BLD pharm on September 27, 2023), shorter reaction time, higher yield (Only some steps have documented yields in the literature, and 29% of by-product **R5'** will be obtained in the harsh S$_N$Ar reaction.), and broader substrate scope. Although the pharmacological properties of compound **PF-D1** remain to be elucidated, we have demonstrated the utility and versatility of our methodology for constructing perfluorinated aryl ketones in drug design, as well as the modifiability of the ketone group for further functionalization. Our synthesis method opens up new possibilities for the discovery and optimization of novel drug candidates based on perfluorinated aryl ketones.

4. Experimental procedure of LIBs test

4.1 Preparation of LNiMnO cathode material pole piece and electrolyte

Preparation of pole pieces

1. First, mix $LiNi_{0.5}Mn_{1.5}O_4$, PVDF, and Super P in a weight ratio of 70:10:20. Then, add a specified amount of *N*-methyl-2-pyrrolidone (NMP) as a solvent and stir the mixture for approximately 20 hr to create a thick slurry. Prior to using a coating machine, ensure the slurry is homogenized using a homogenizer for even dispersion.

2. Utilize a coating machine to apply the slurry evenly onto battery-grade aluminum foil while maintaining a thickness of 200 μm. Solidify the electrode surface at 110 °C using a heater, and then subject it to a 12-hour heat treatment at 80 °C in a vacuum oven to remove the solvent.

3. Using an automatic rolling machine, roll the dried electrode sheet with a 59% rolling rate.

4. Cut the electrode sheets into circular pieces with a 1.3 cm diameter by an electrode sheet cutter, and then transfer them into an argon-filled glovebox to assemble coin cell batteries.

Preparation of electrolyte

Inside the glovebox, thoroughly mix ethylene carbonate (EC) and diethyl carbonate (DEC) in a 1:1 (v/v) ratio. Then, add 1 M lithium hexafluorophosphate ($LiPF_6$) until it completely dissolves, creating the standard electrolyte. The additives are dissolved in the standard electrolyte at a weight concentration of 0.05 wt%.

Preparation of half-Cell

The processed $LiNi_{0.5}Mn_{1.5}O_4$ cathode material electrode is placed into an argon gas-filled glovebox. The battery being prepared in this experiment is a CR2032 half-cell with the following assembly steps:

1. Weigh the electrode, position it centrally in the lower cover, ensuring that the coated side faces upward.
2. Submerge the PP separator in the electrolyte to ensure full saturation and place it on top of the electrode. This ensures effective isolation of lithium metal from direct contact with the cathode material to prevent short circuits.
3. Attach lithium metal, facing downward, to the spacer on the spring tab, directly onto the separator.
4. Seal the cell by affixing the upper cover and using a battery sealing machine to ensure a secure closure.

4.2 Cycle Life Test

In this study, constant current charge-discharge tests are performed. The voltage is charged from the initial voltage to the termination voltage over time and then discharged from the termination voltage back to the initial voltage, completing one cycle. The purpose is to observe voltage variations during this cycle and obtain electrochemical data such as charge-discharge capacity for each cycle and efficiency per cycle. This data is crucial for assessing capacitance retention and the stability of battery lifespan.

5. Proposed mechanism

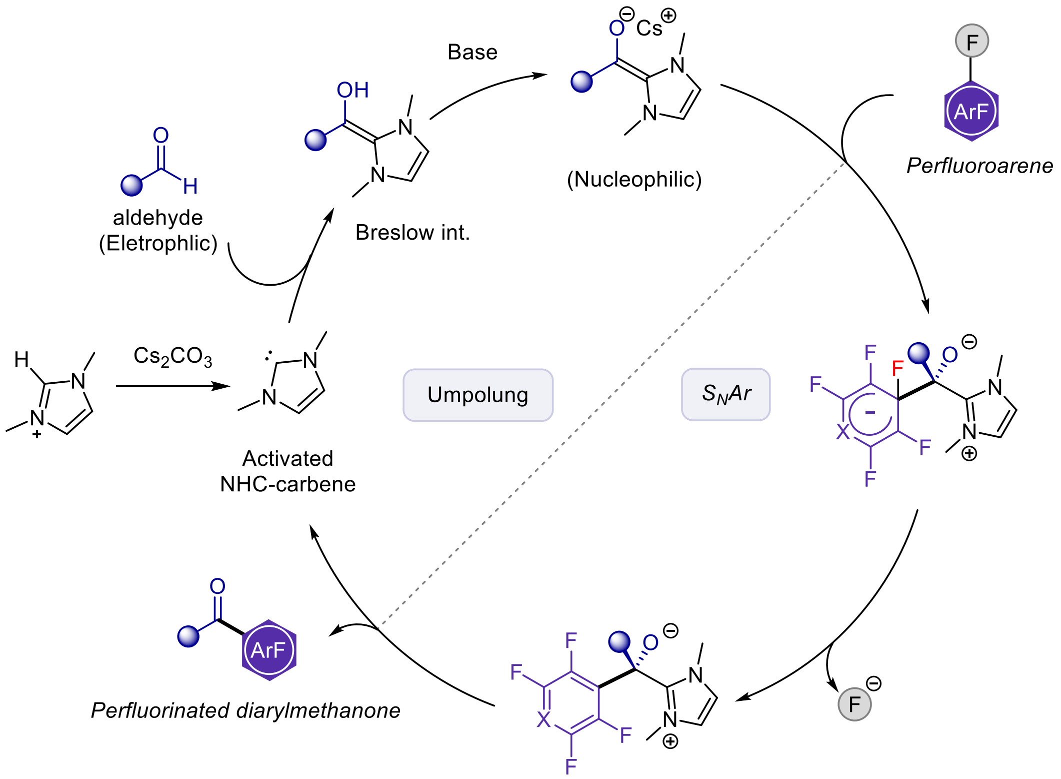

The potential mechanism for the NHC-catalyzed S_NAr reaction may commence with the deprotonation of **NHC1** salt, activating the NHC carbene. After activation, the Breslow intermediate form through nucleophilic addition to the aldehyde. Under basic conditions, the Breslow intermediate attacks the 4-position of the perfluoroarene, leading to defluorination and yielding the desired perfluorinated diarylmethanones.

6. Reference

1. Gardner, S.; Kawamoto, T.; Curran, D. P. Synthesis of 1, 3-dialkylimidazol-2-ylidene boranes from 1, 3-dialkylimidazolium iodides and sodium borohydride. *J. Org. Chem.* **2015**, *80*, 19, 9794–9797

2. Lebeuf, R.; Hirano, K.; Glorius, F. Palladium-catalyzed C-allylation of benzoins and an NHC-catalyzed three component coupling derived thereof: compatibility of NHC-and Pd-catalysts. *Organic Letters* **2008**, *10*, 19, 4243–4246.

3. Fuchss, T.; Emde, U., Buchstaller, H-P.; Mederski, W. Arylquinazolines. U.S. Patent US 2022/0117970 A1, April 21, 2022

[Back to the contents](#)

7. NMR spectra

^{1}H NMR (300 MHz, $CDCl_3$) of **2c'**, See procedure

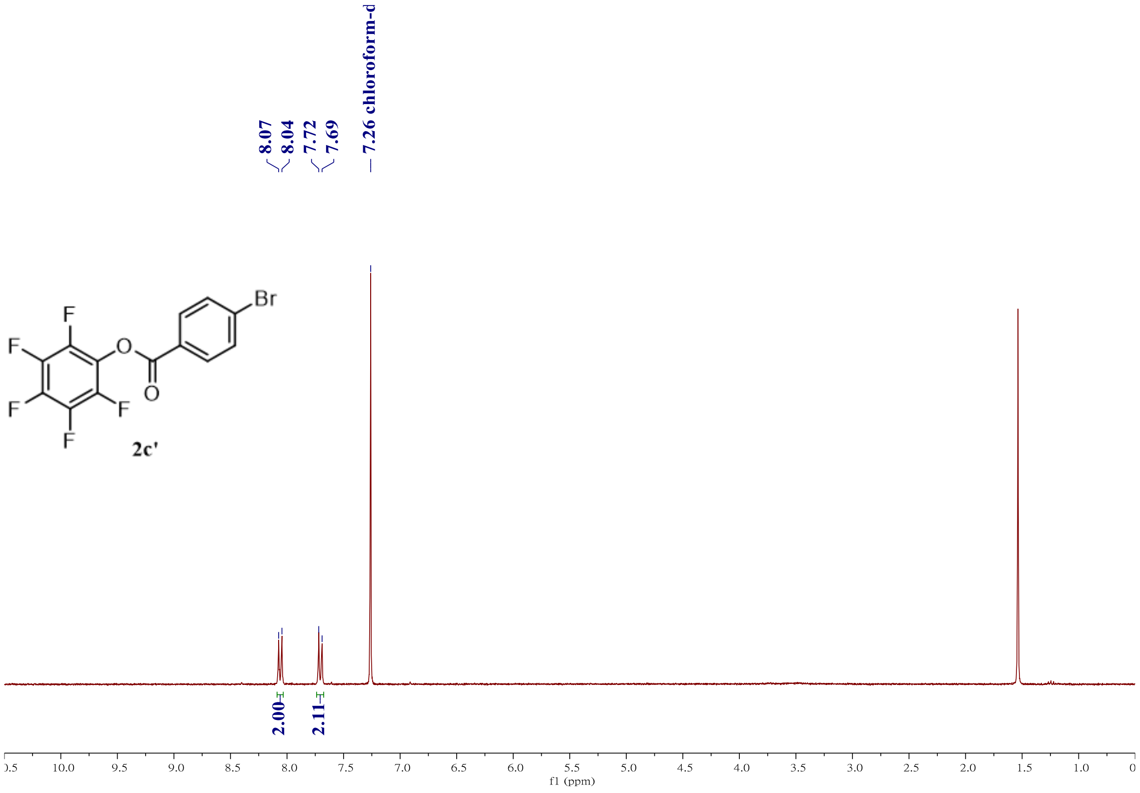

^{13}C NMR (101 MHz, $CDCl_3$) of **2c'**

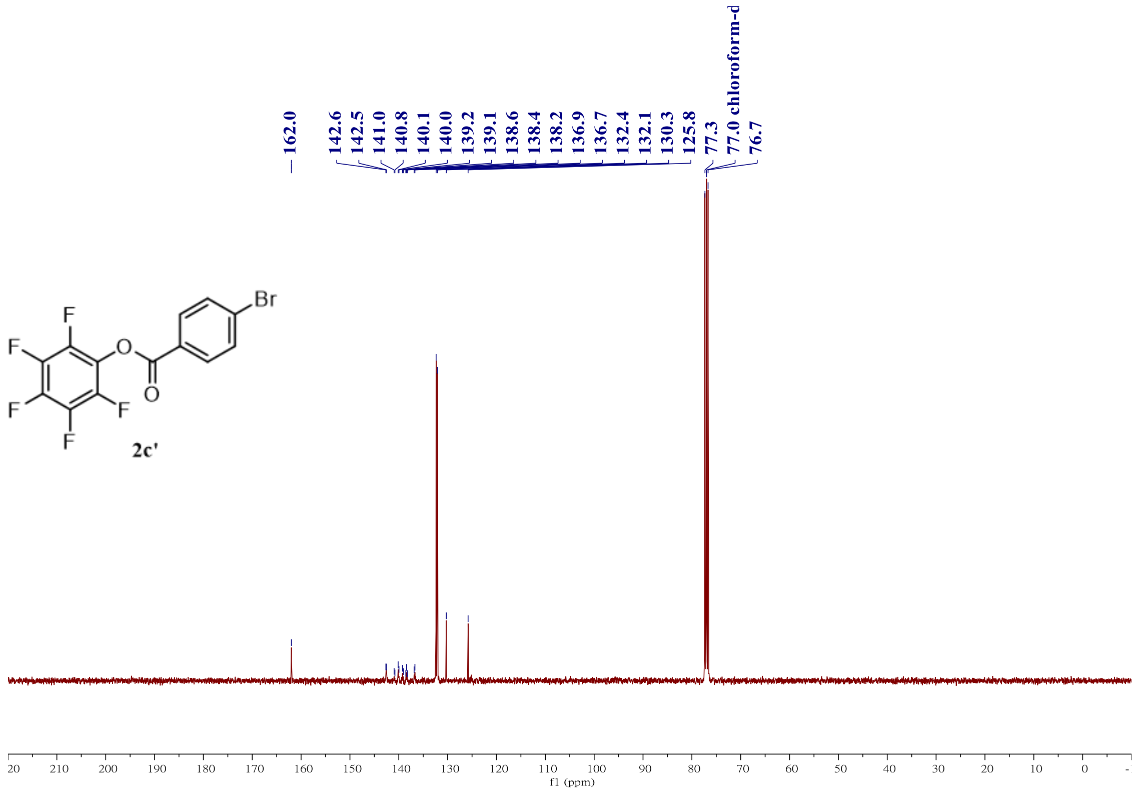

Back to the contents

^{19}F NMR (282 MHz, $CDCl_3$) of **2c'**

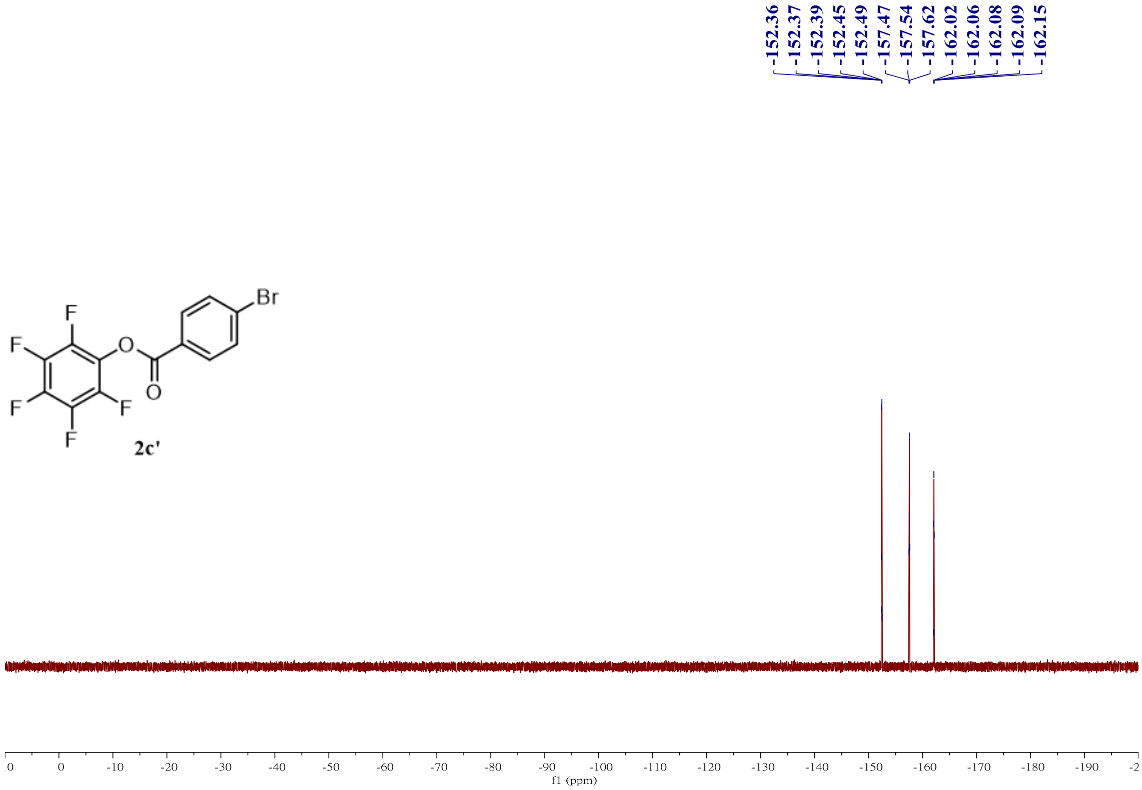

^{1}H NMR (300 MHz, $CDCl_3$) of **2j**, *See procedure*

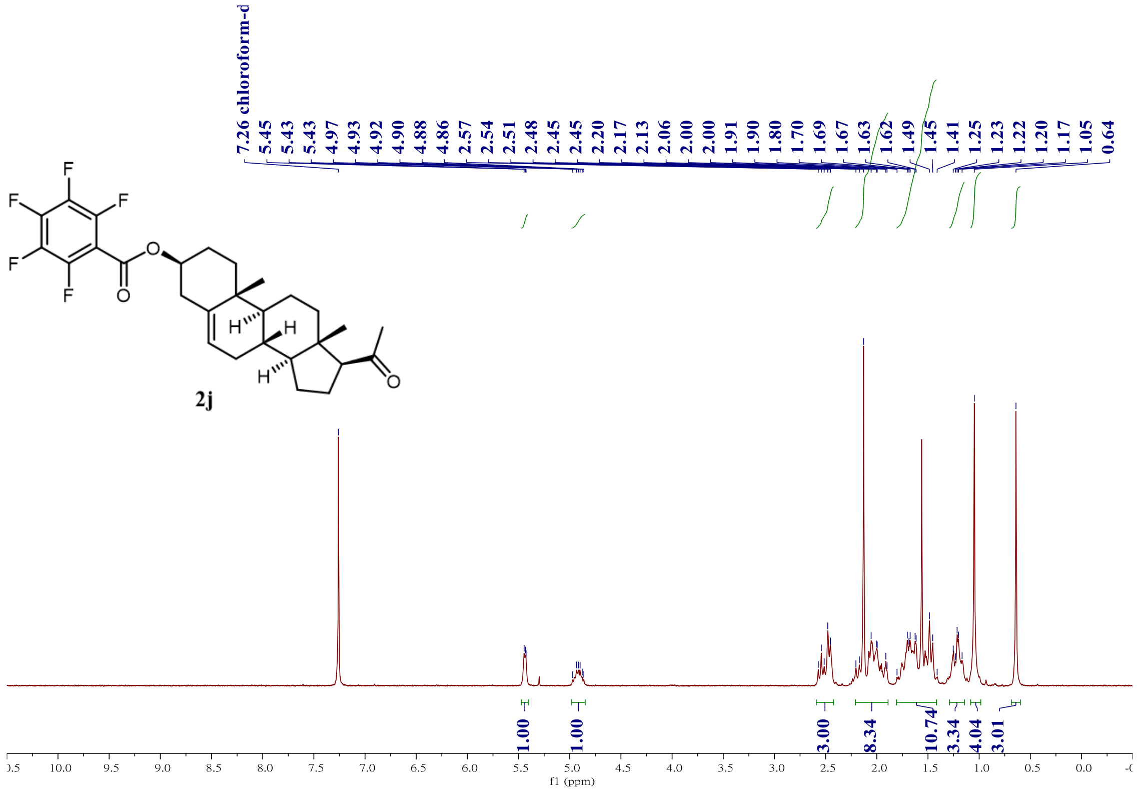

Back to the contents

^{13}C NMR (101 MHz, $CDCl_3$) of **2j**

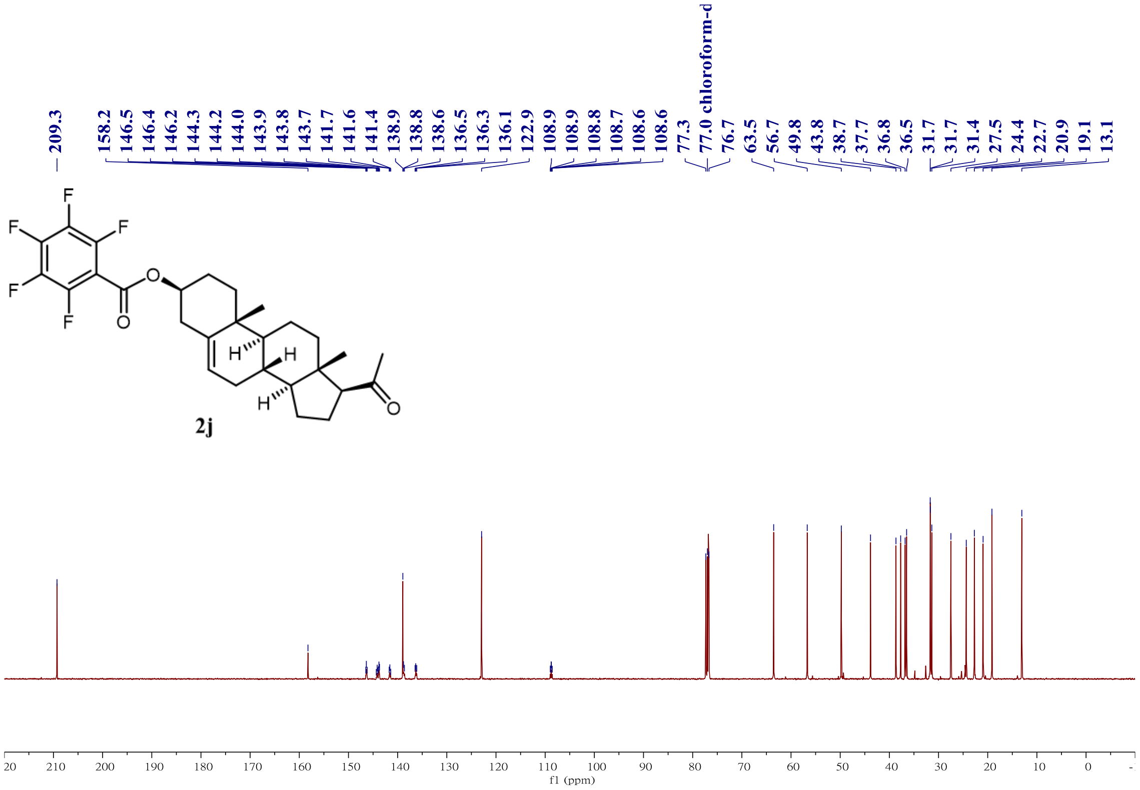

^{19}F NMR (282 MHz, $CDCl_3$) of **2j**

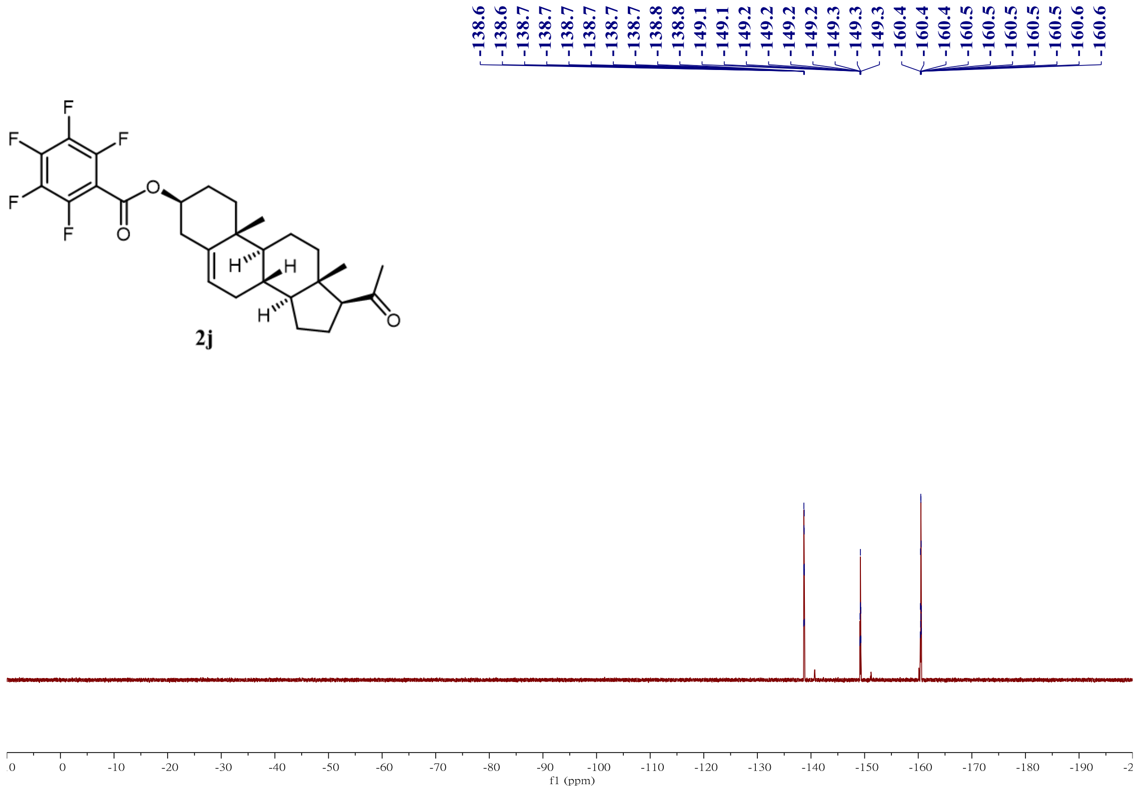

Back to the contents

^{1}H NMR (300 MHz, *d*-methanol) of **NHC1**, See procedure

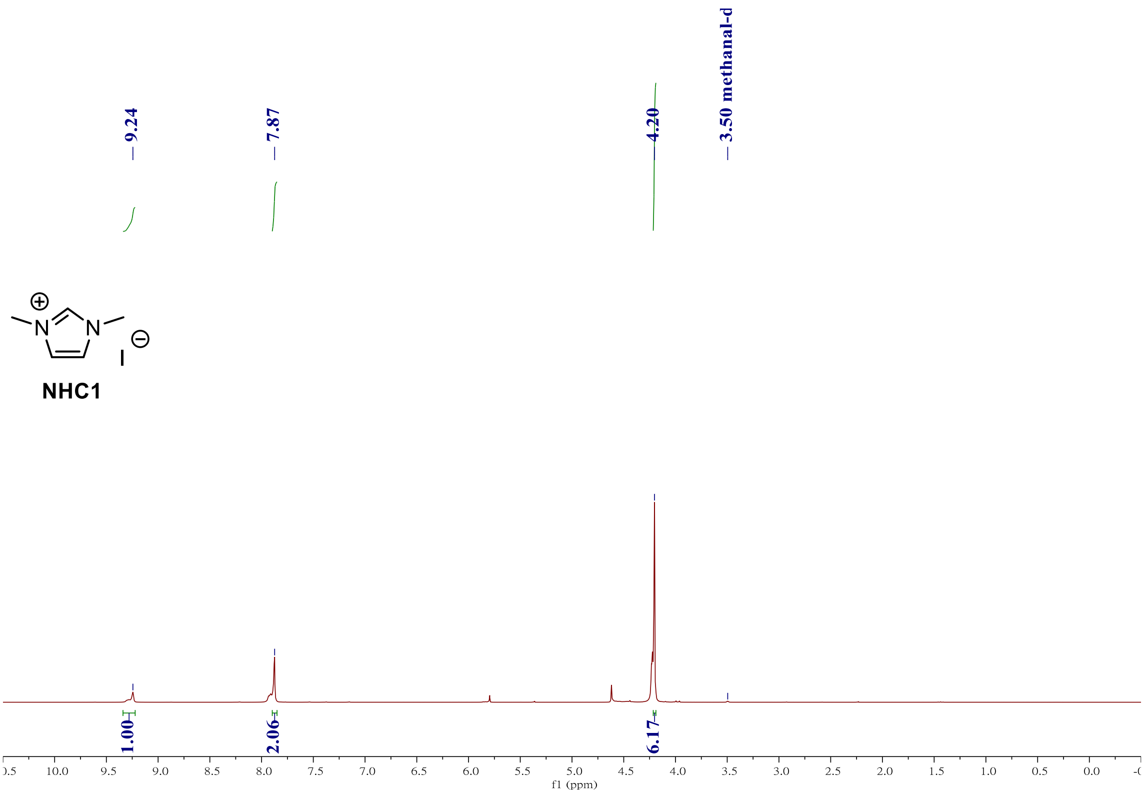

^{13}C NMR (101 MHz, $CDCl_3$) of **NHC1**

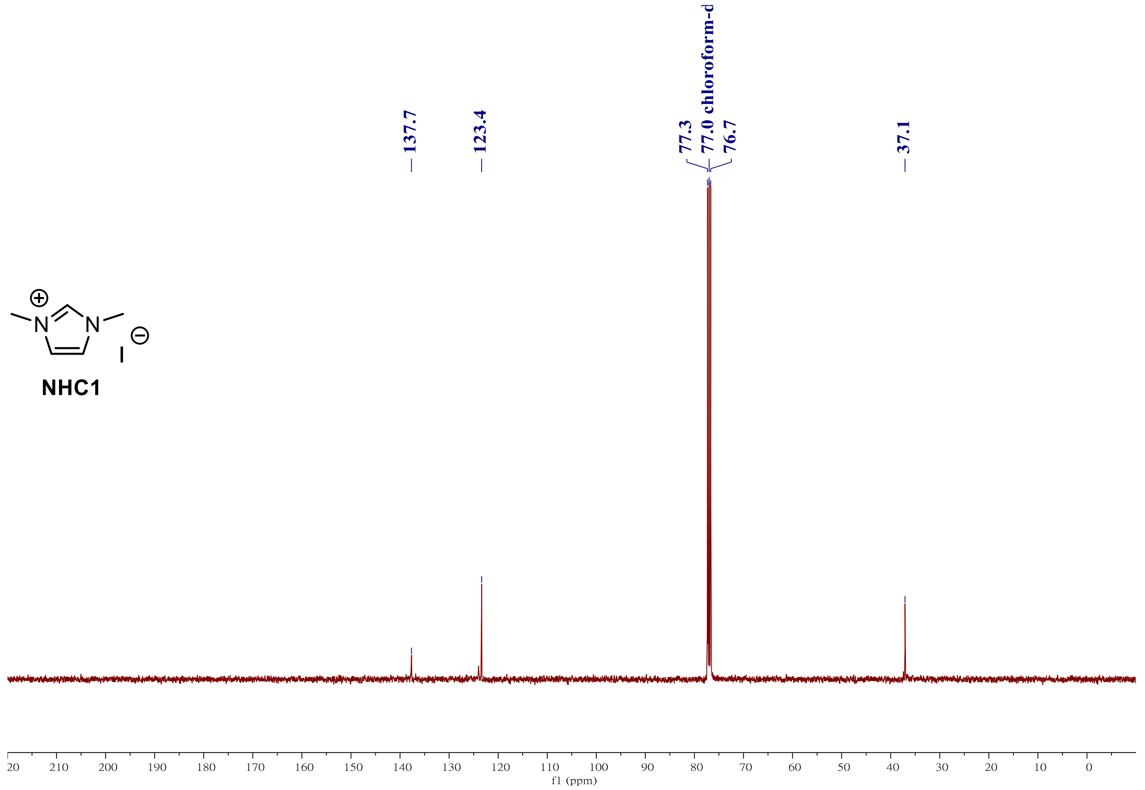

Back to the contents

^{1}H NMR (300 MHz, $CDCl_3$) of **NHC4**, See procedure

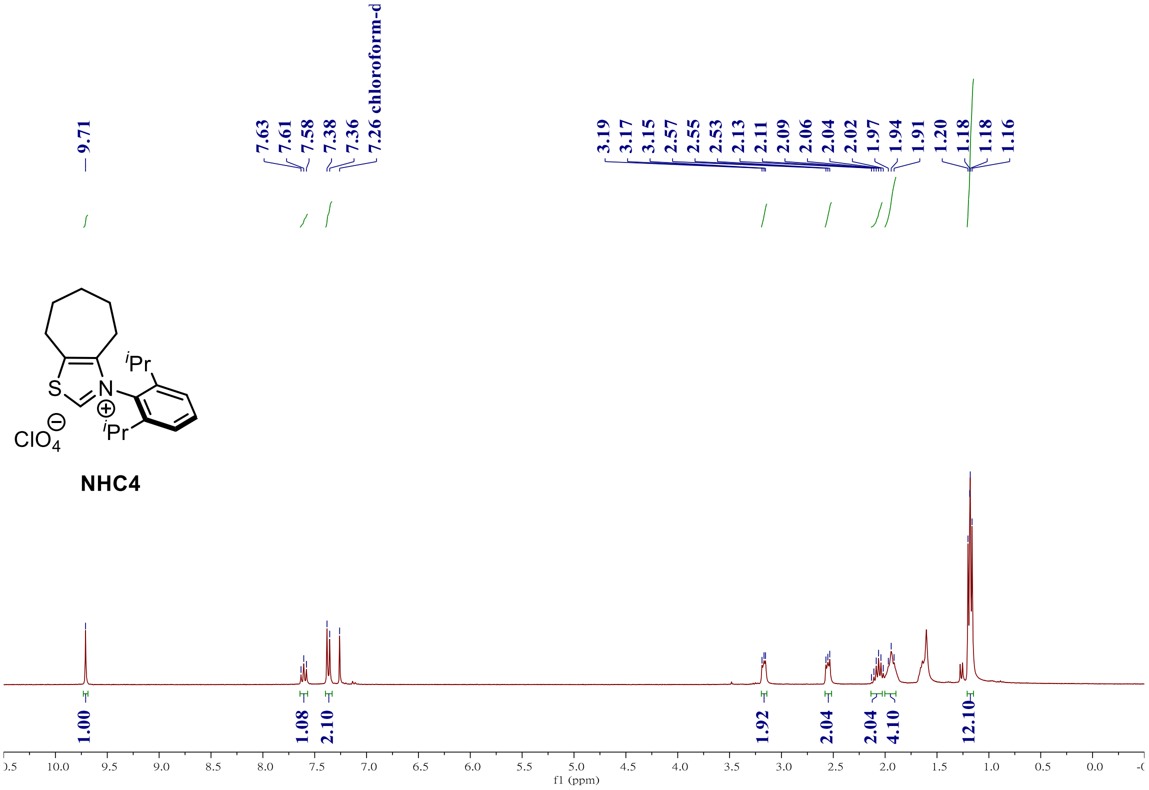

^{13}C NMR (101 MHz, $CDCl_3$) of **NHC4**

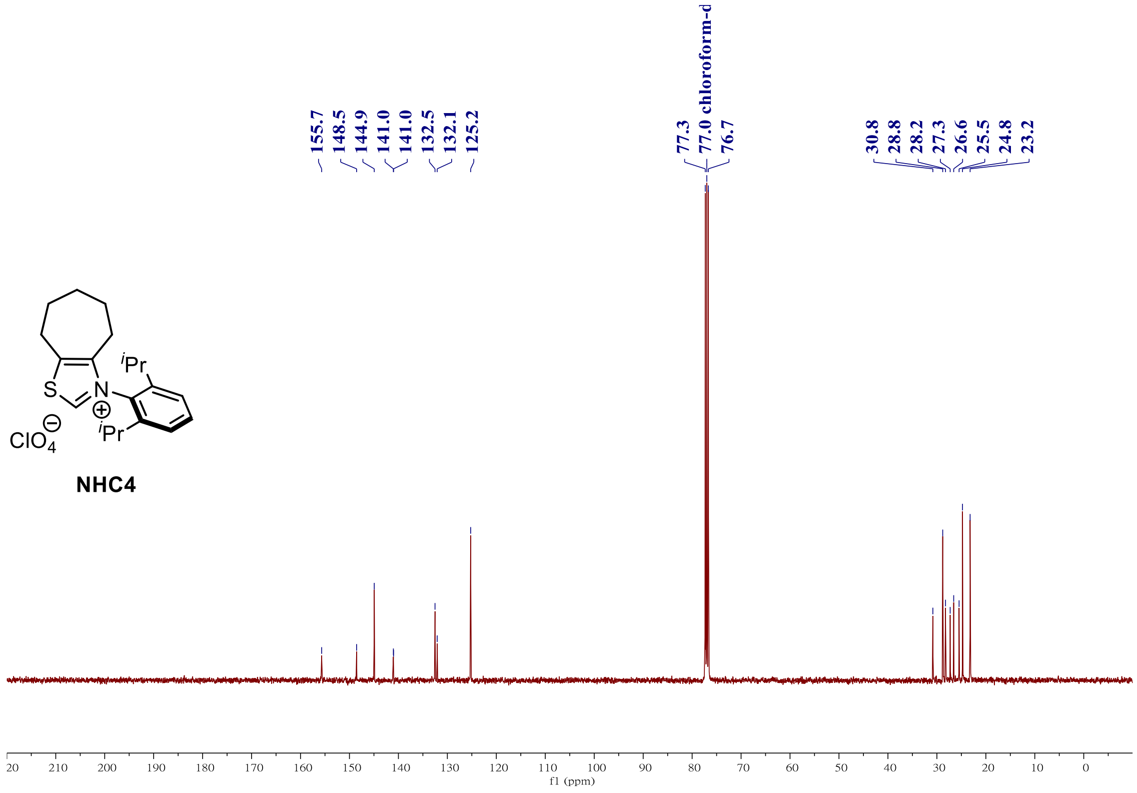

[Back to the contents](#)

Spectrum of substrate scope

^{1}H NMR (400 MHz, $CDCl_3$) of **3a**, See procedure

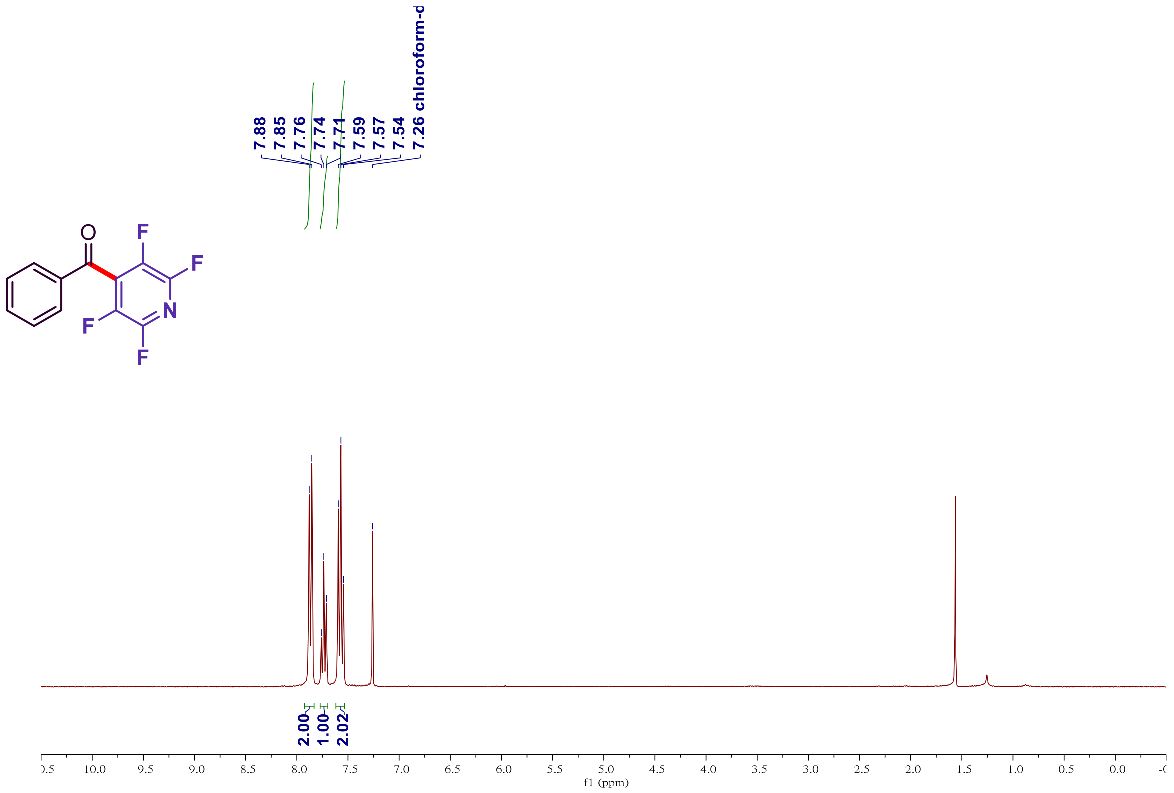

^{13}C NMR (101 MHz, $CDCl_3$) of **3a**

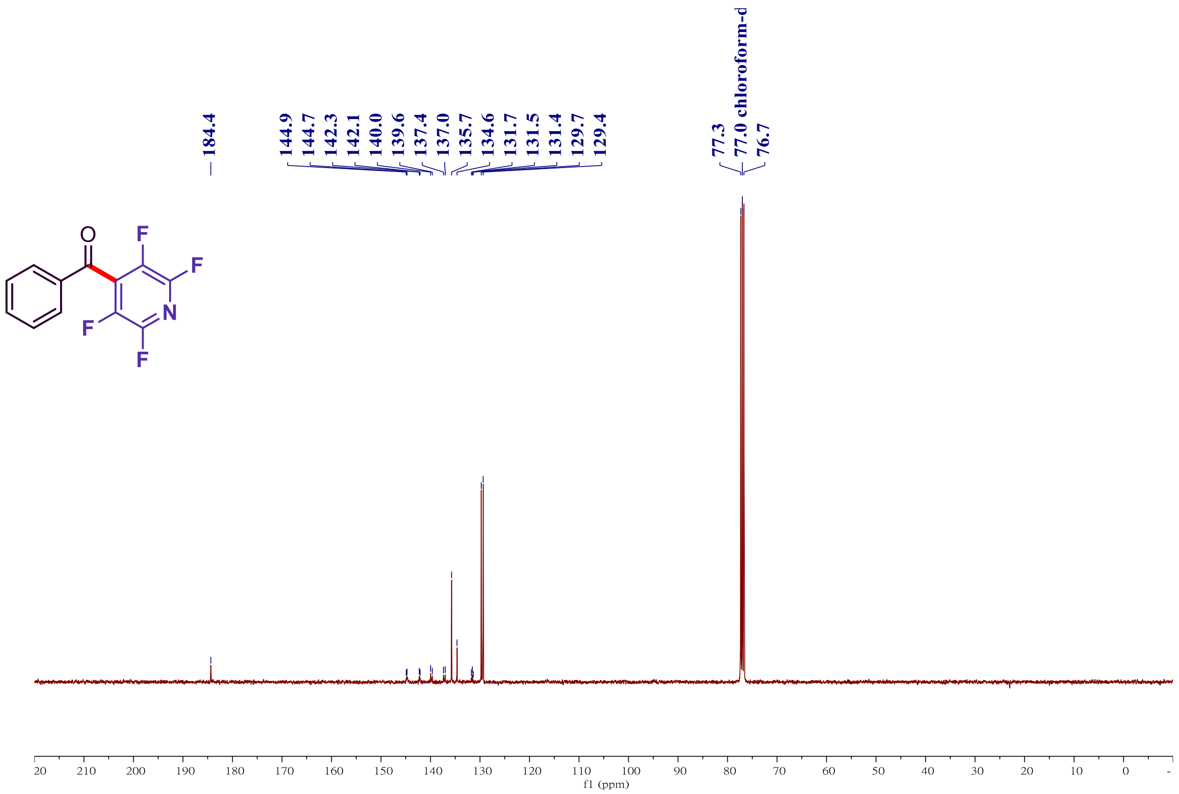

Back to the contents

19F NMR (282 MHz, $CDCl_3$) of **3a**

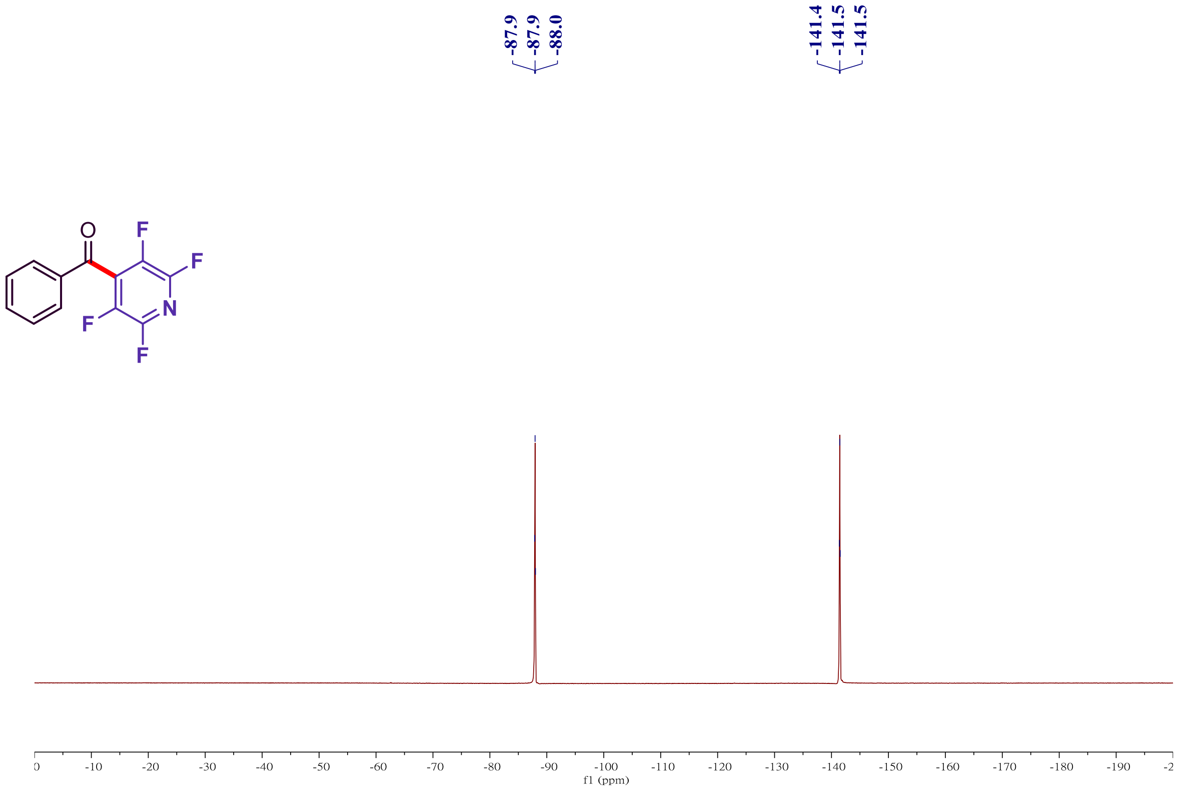

1H NMR (300 MHz, $CDCl_3$) of **3b**, See procedure

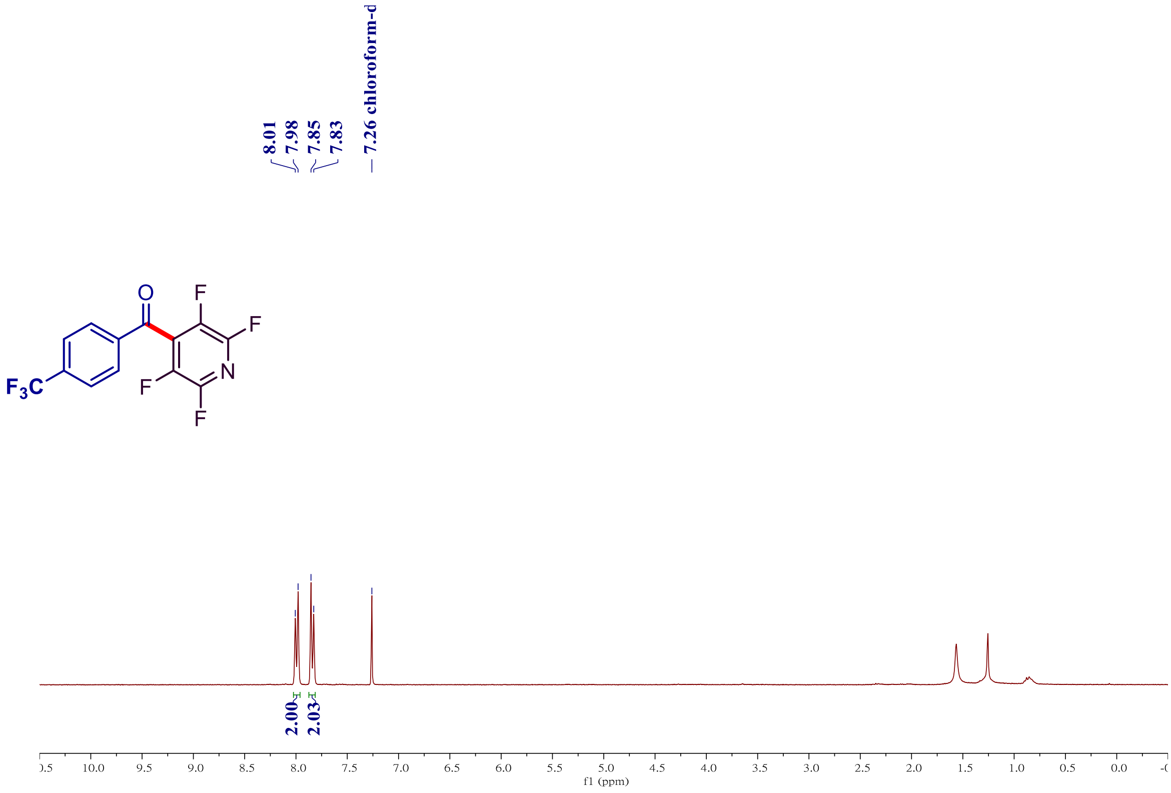

Back to the contents

^{13}C NMR (101 MHz, $CDCl_3$) of **3b**

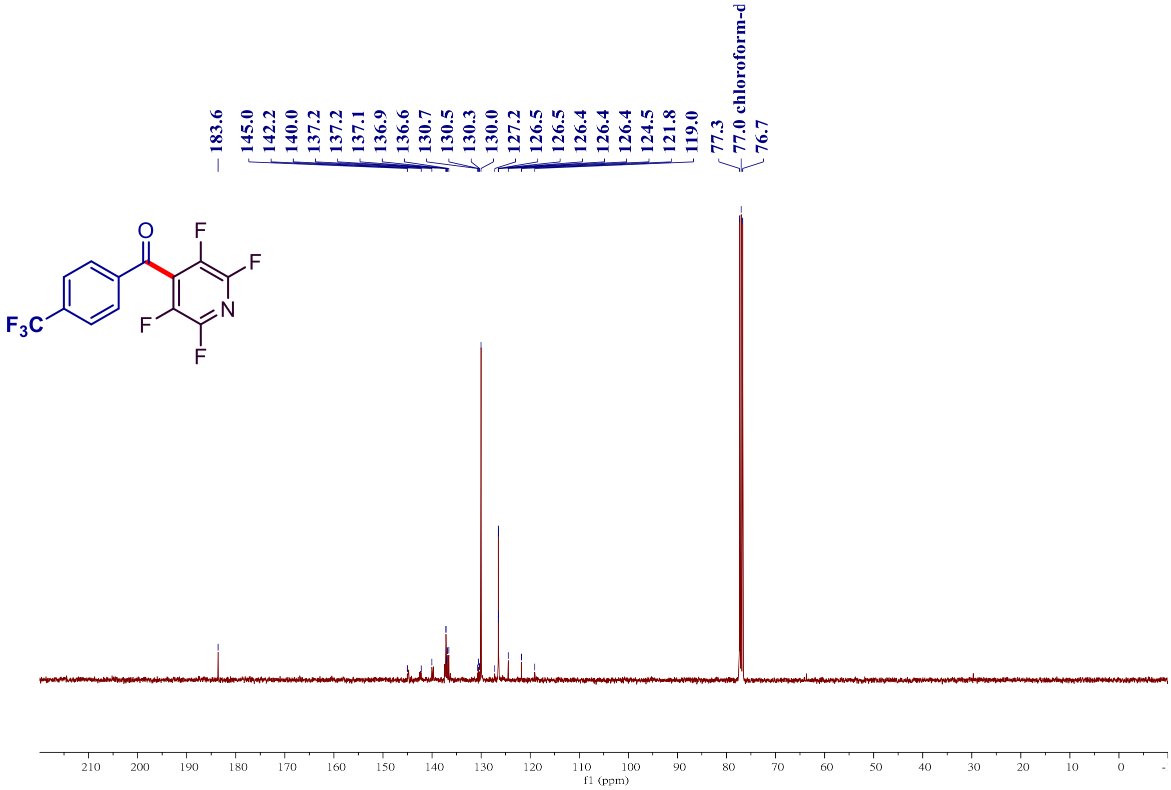

^{19}F NMR (282 MHz, $CDCl_3$) of **3b**

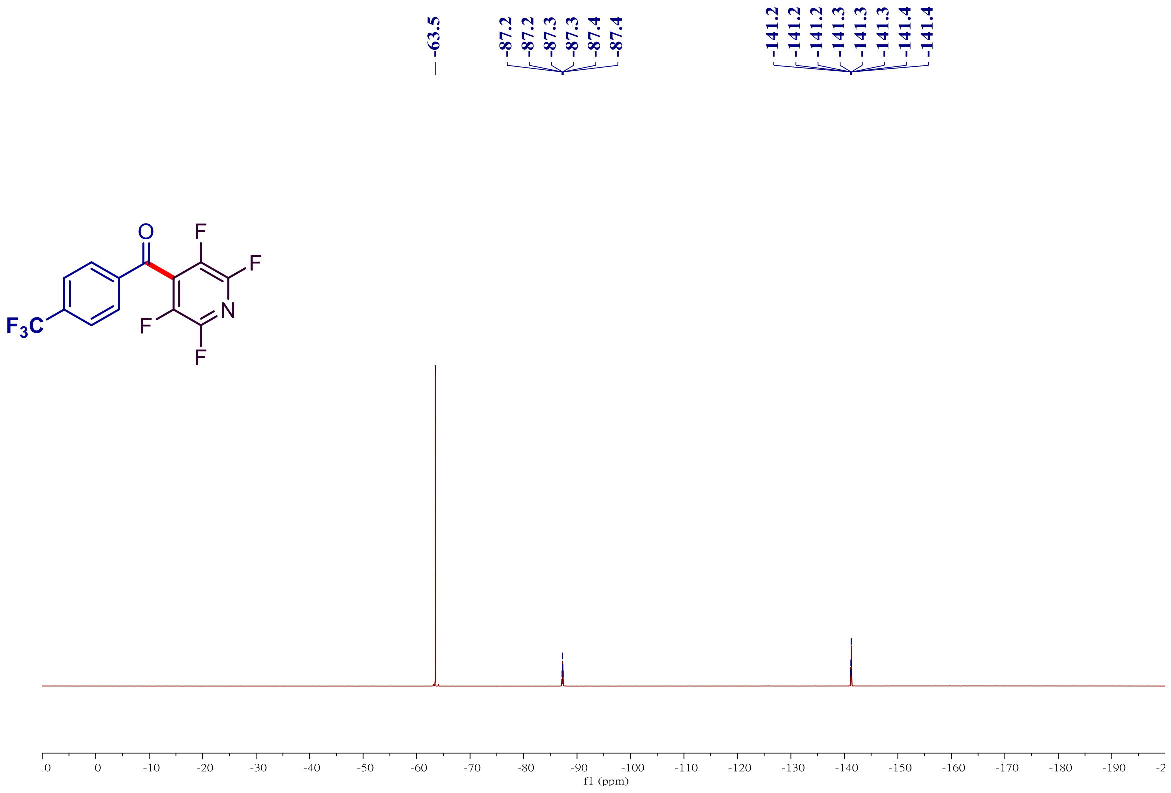

Back to the contents

^{1}H NMR (300 MHz, $CDCl_3$) of **3c**, See procedure

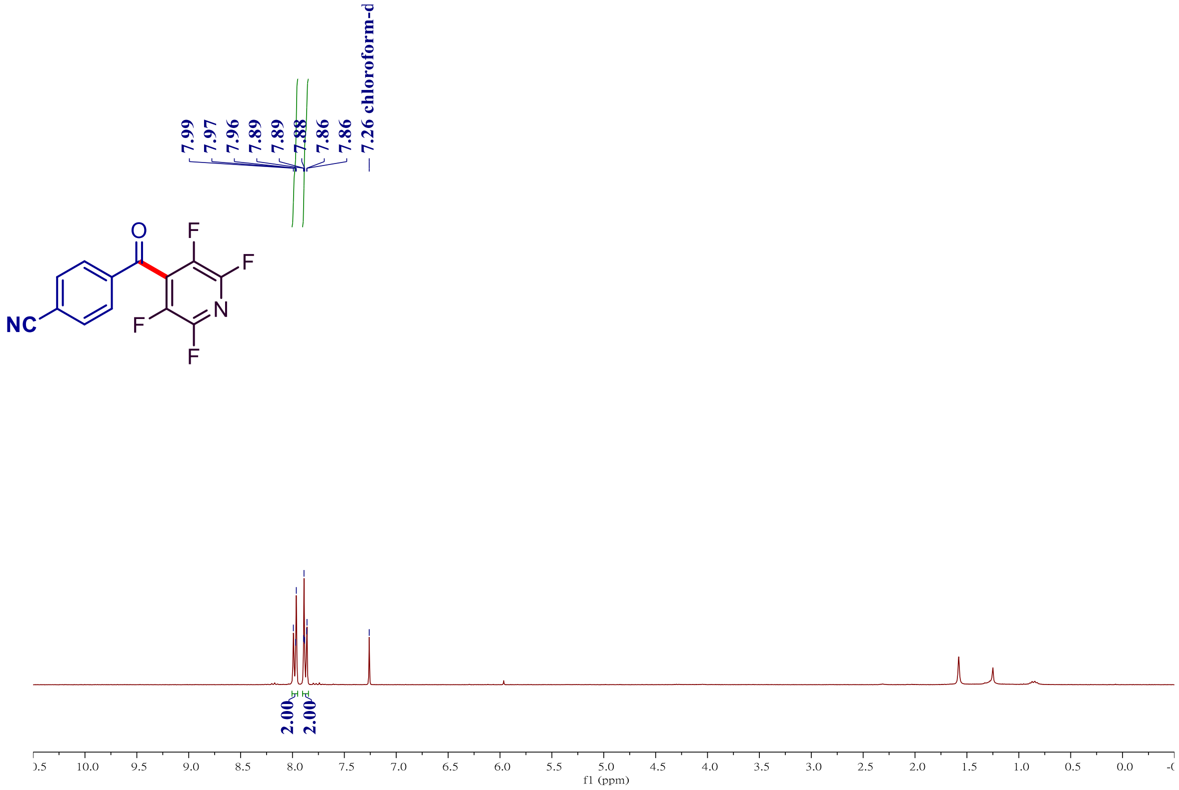

^{13}C NMR (101 MHz, $CDCl_3$) of **3c**

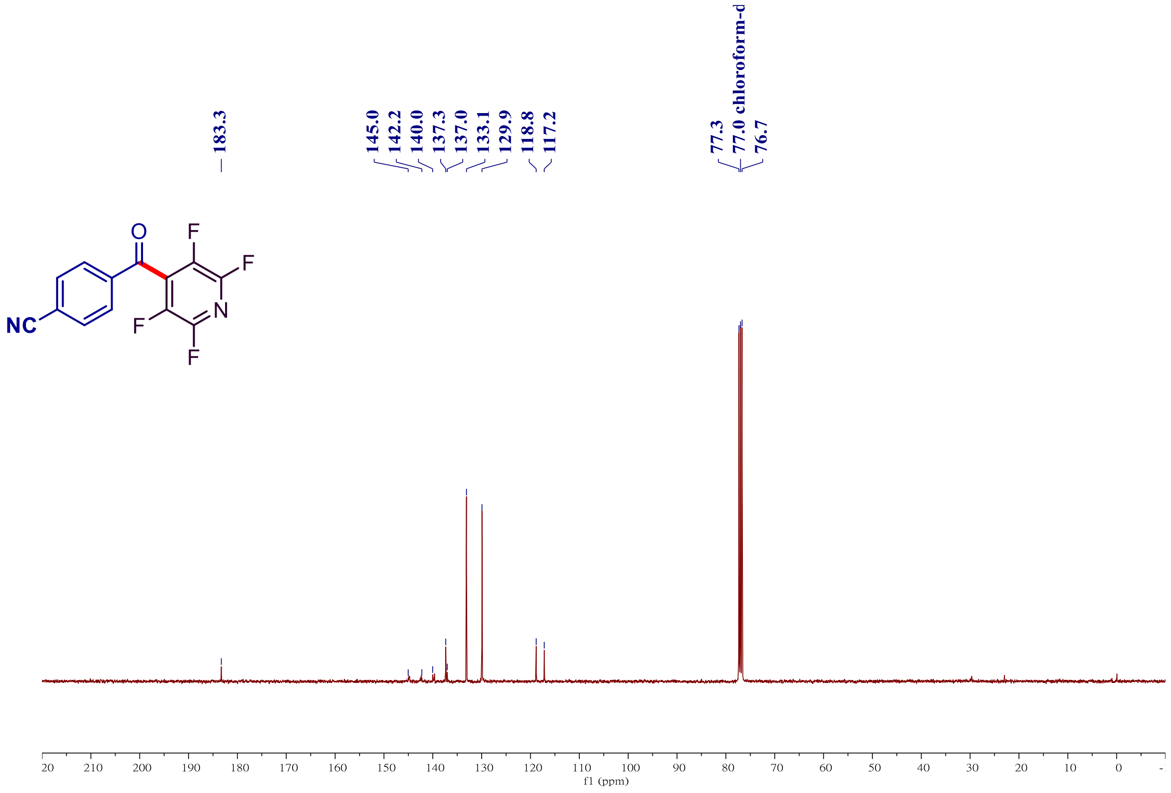

Back to the contents

^{19}F NMR (282 MHz, $CDCl_3$) of **3c**

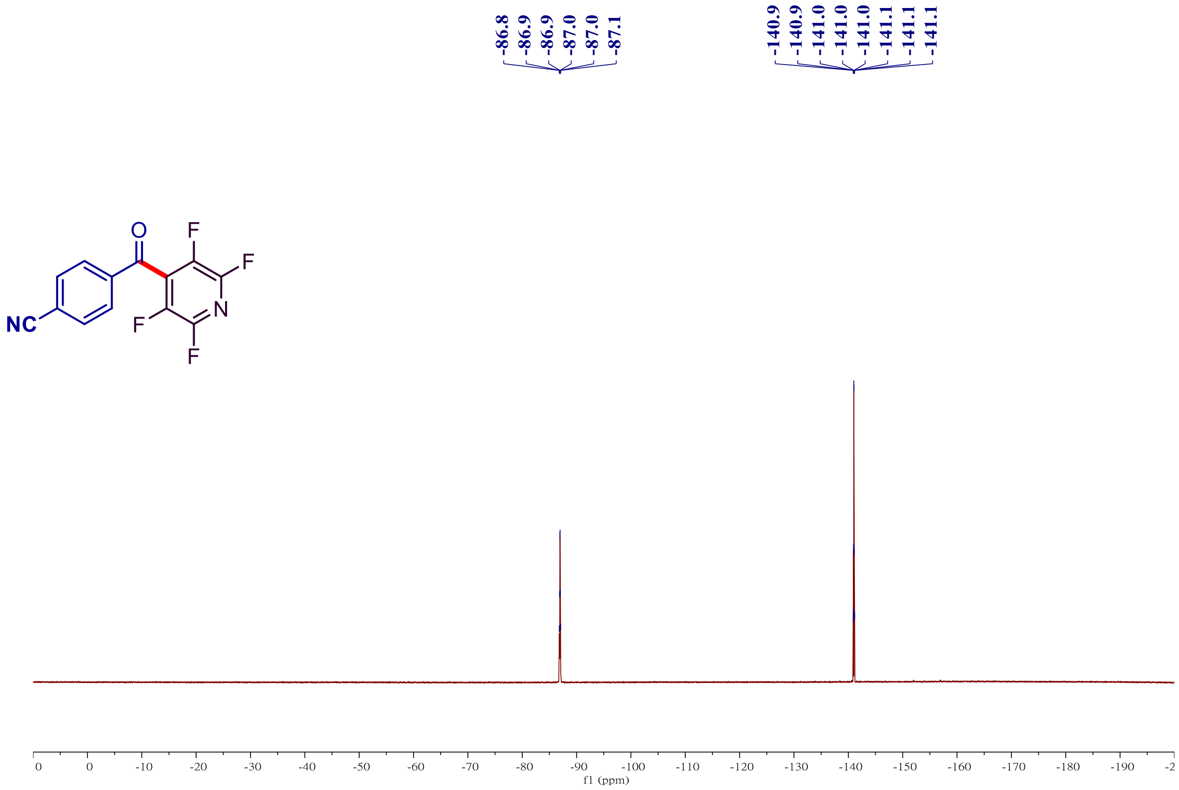

^{1}H NMR (300 MHz, $CDCl_3$) of **3d**, See procedure

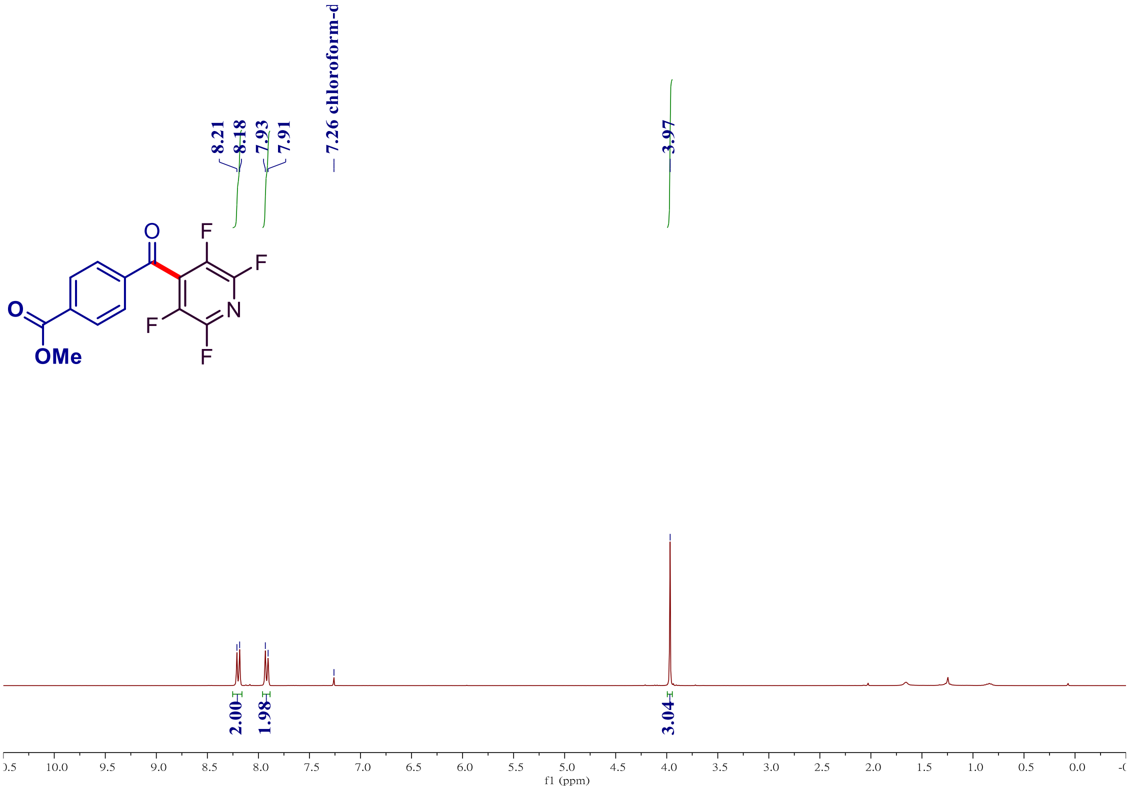

[Back to the contents](#)

^{13}C NMR (101 MHz, $CDCl_3$) of **3d**

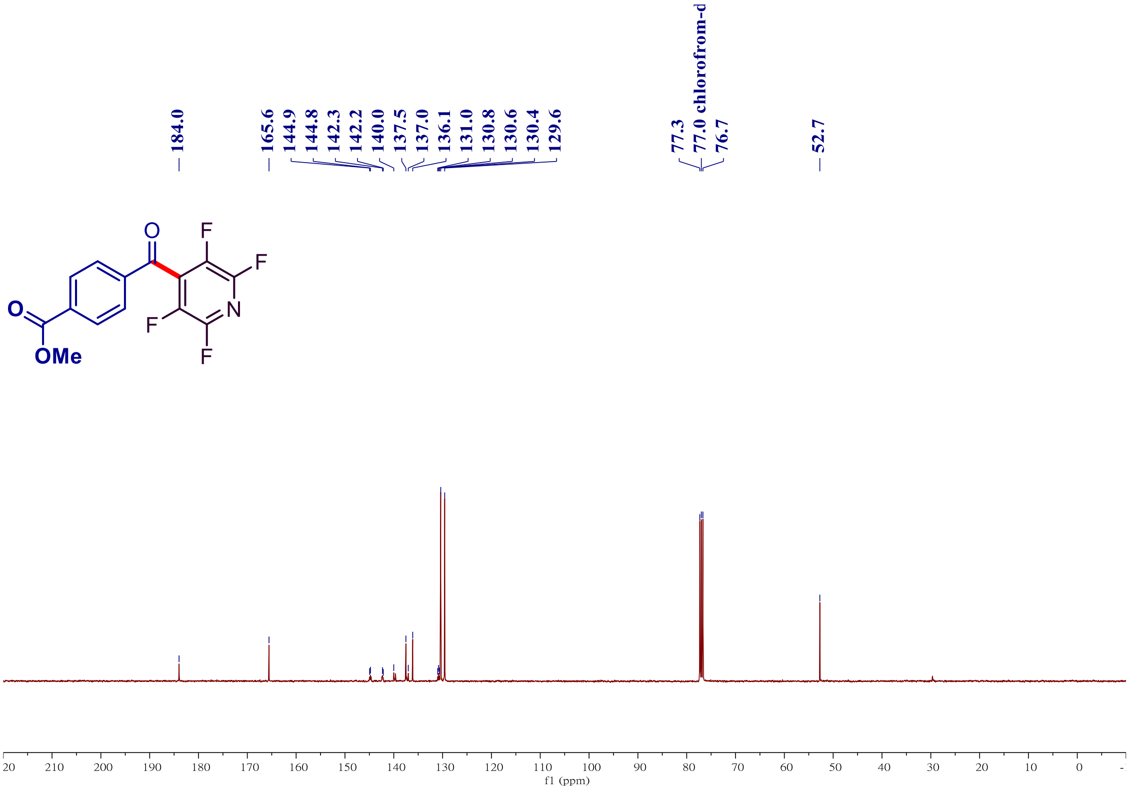

^{19}F NMR (282 MHz, $CDCl_3$) of **3d**

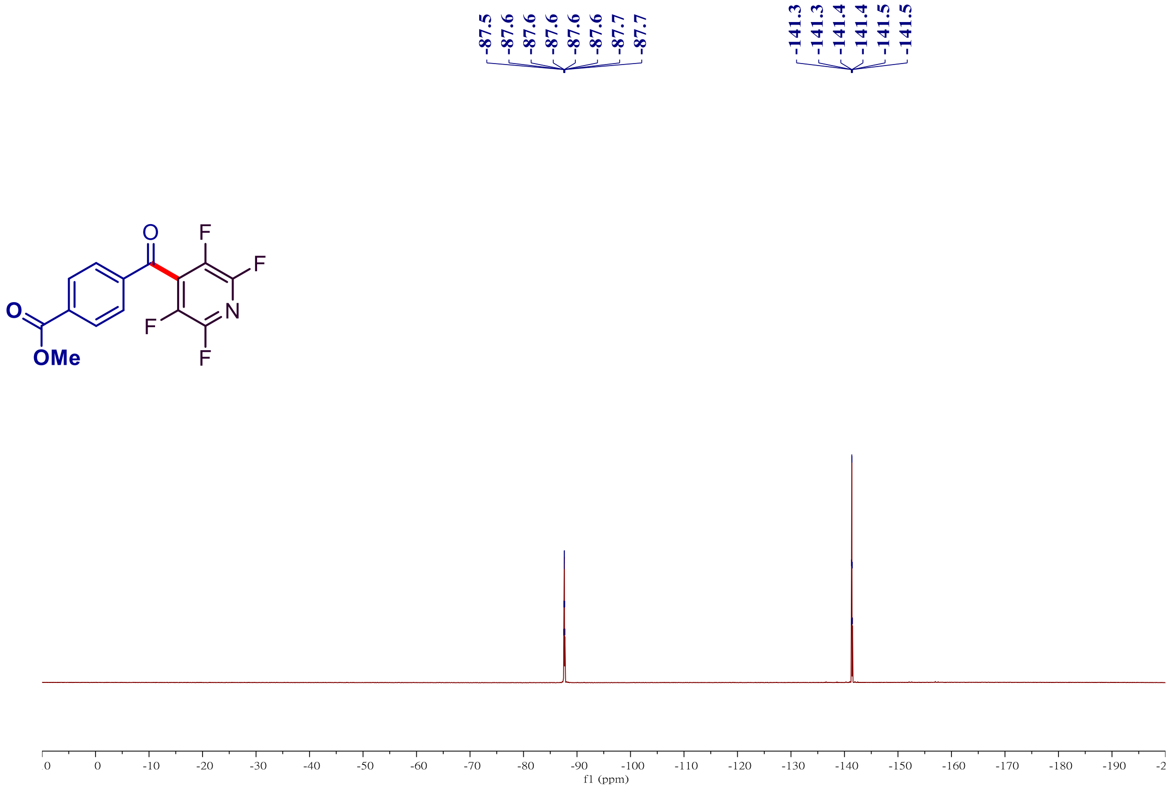

Back to the contents

^{1}H NMR (300 MHz, $CDCl_3$) of **3e**, See procedure

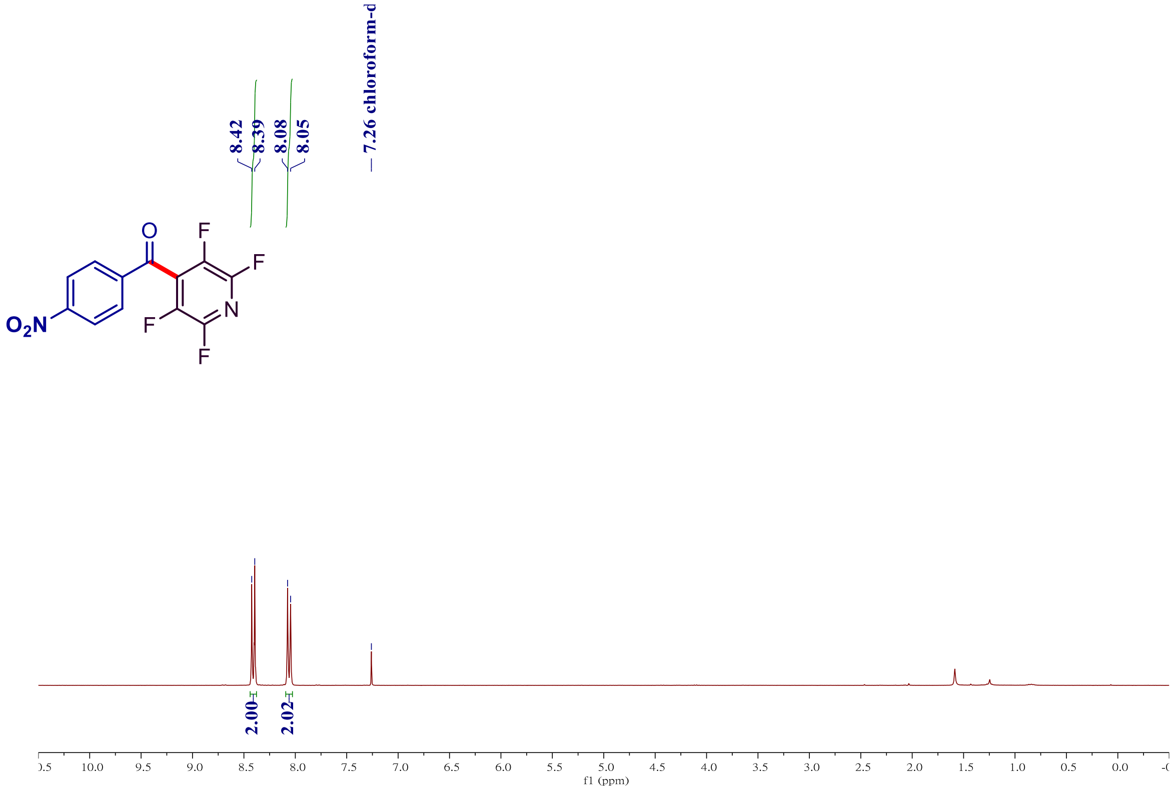

^{13}C NMR (101 MHz, $CDCl_3$) of **3e**

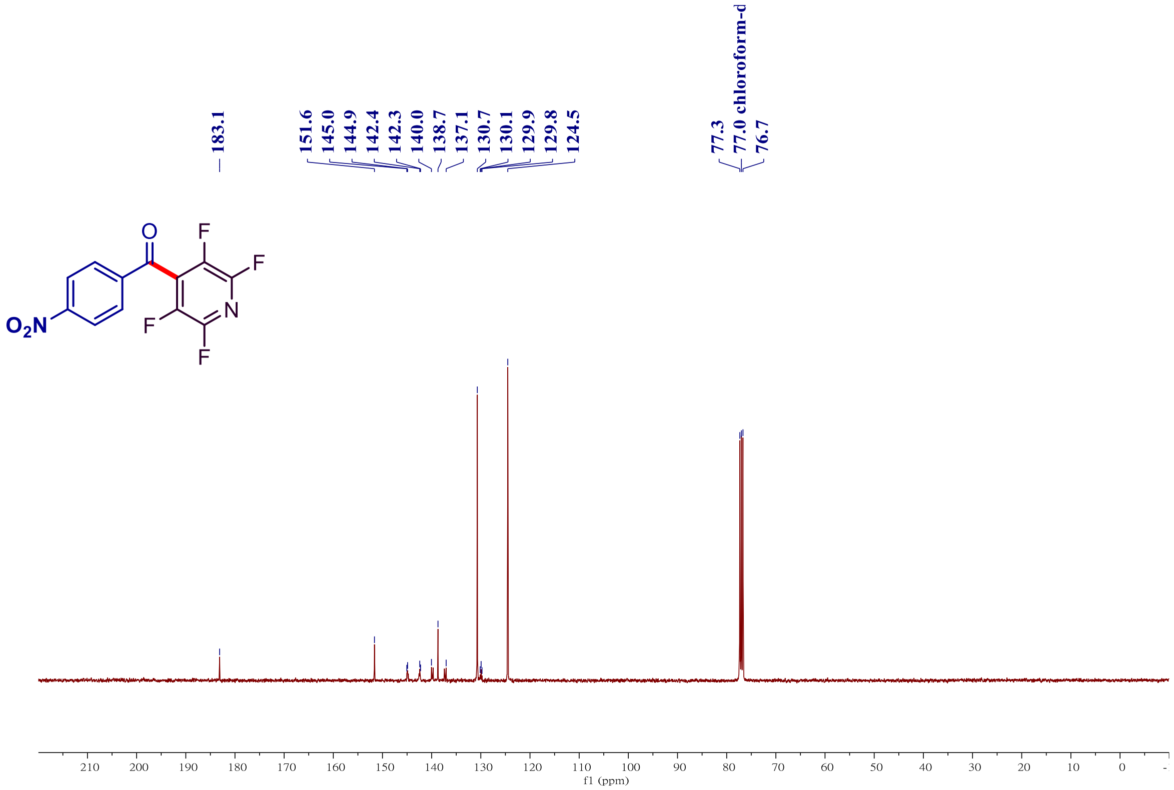

Back to the contents

^{19}F NMR (282 MHz, $CDCl_3$) of **3e**

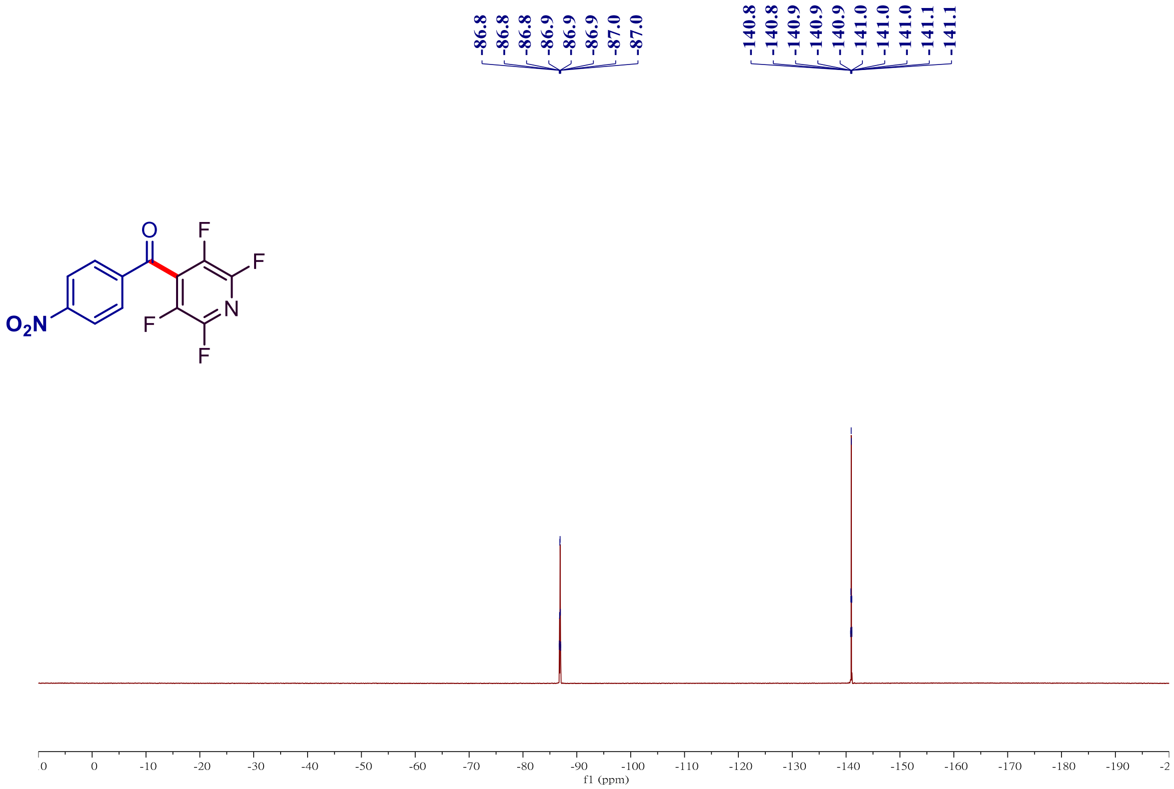

^{1}H NMR (300 MHz, $CDCl_3$) of **3f**, See procedure

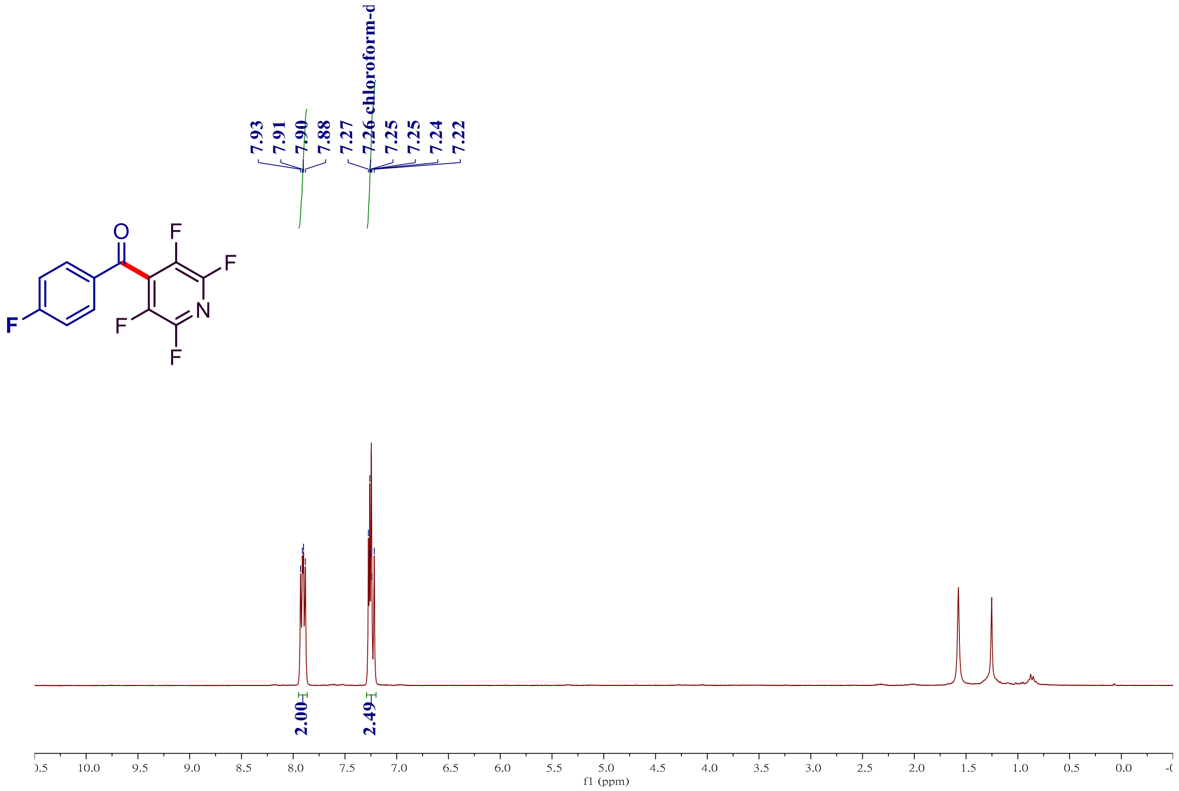

Back to the contents

^{13}C NMR (101 MHz, $CDCl_3$) of **3f**

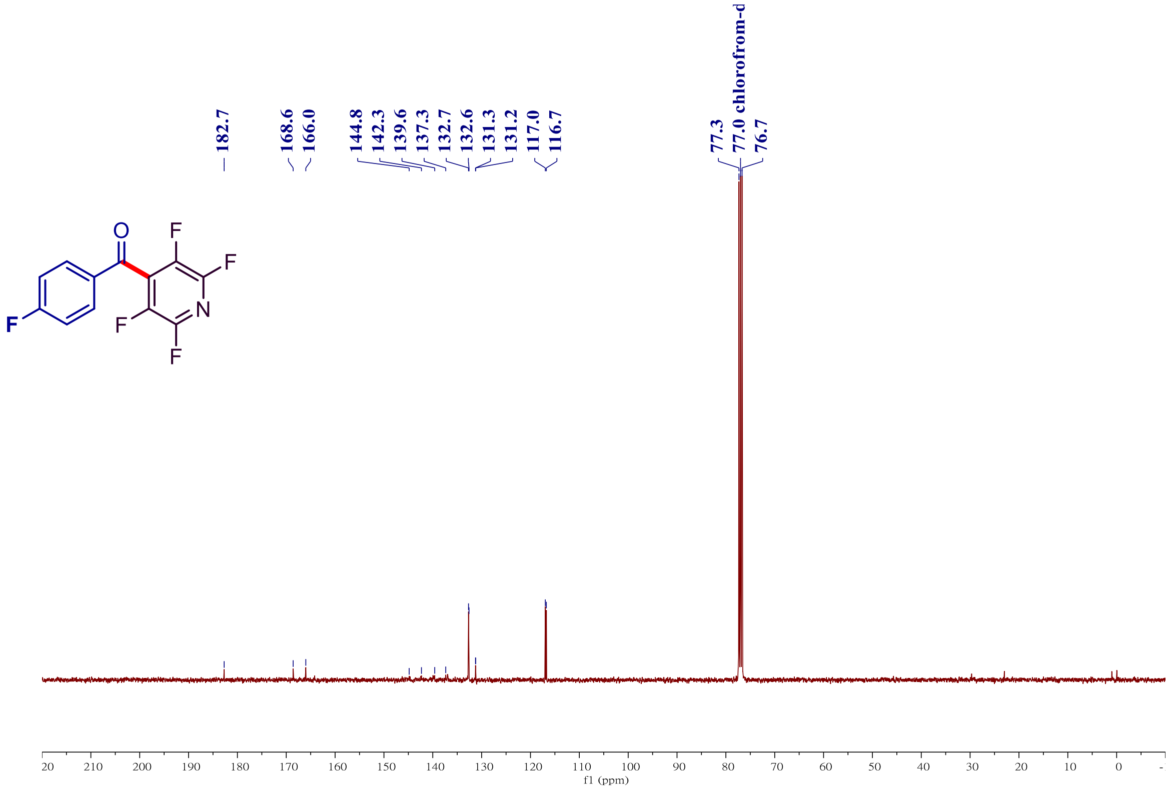

^{19}F NMR (376 MHz, $CDCl_3$) of **3f**

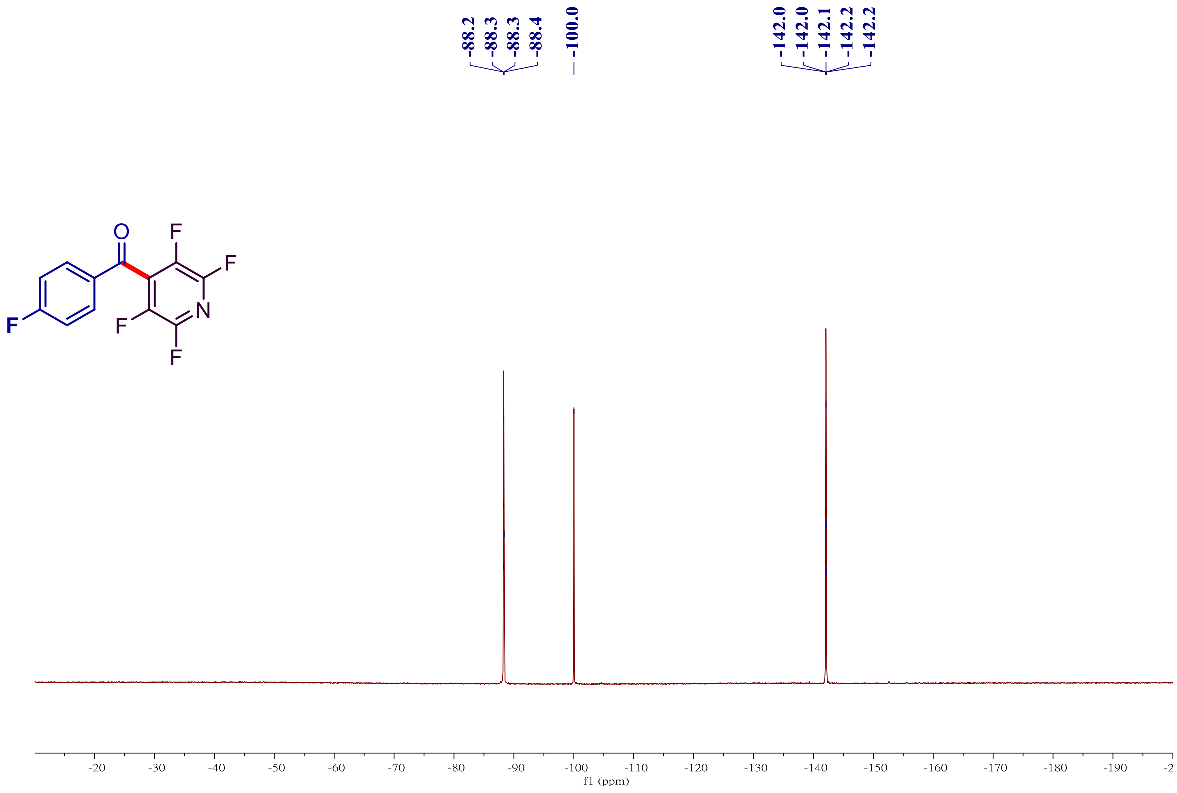

Back to the contents

¹H NMR (300 MHz, CDCl₃) of **3g**, See procedure

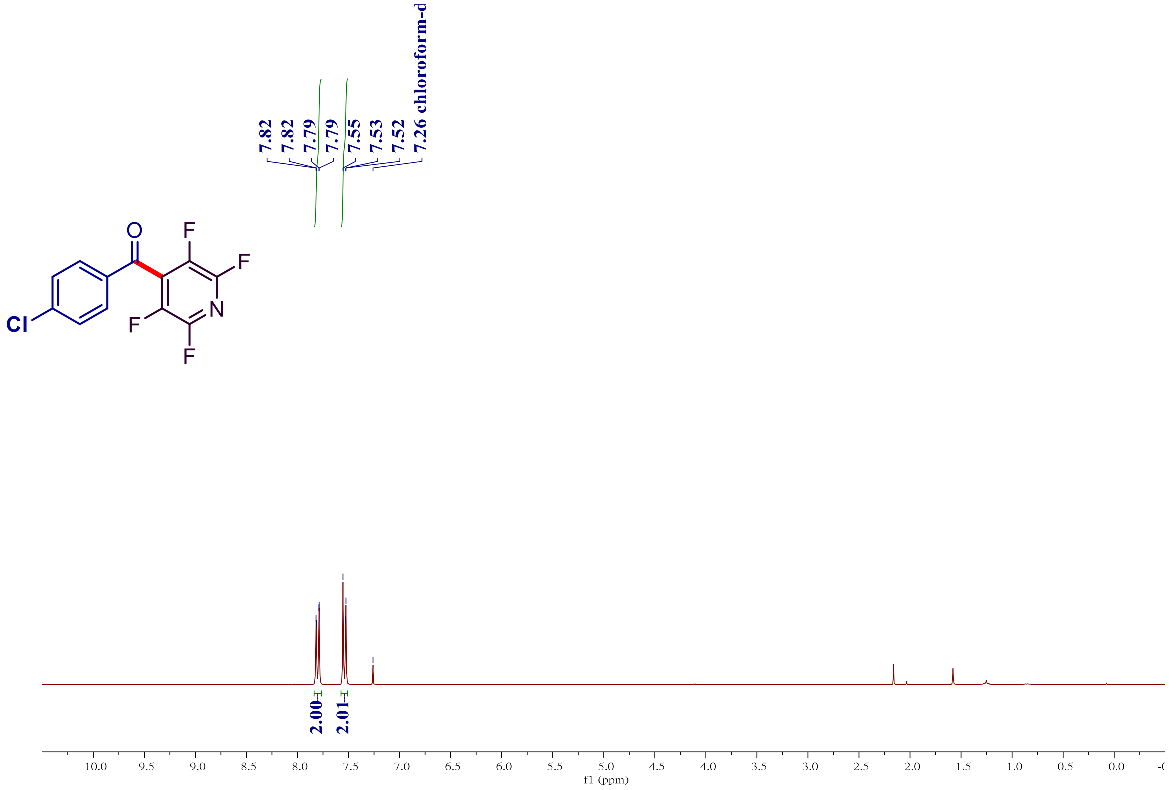

¹³C NMR (101 MHz, CDCl₃) of **3g**

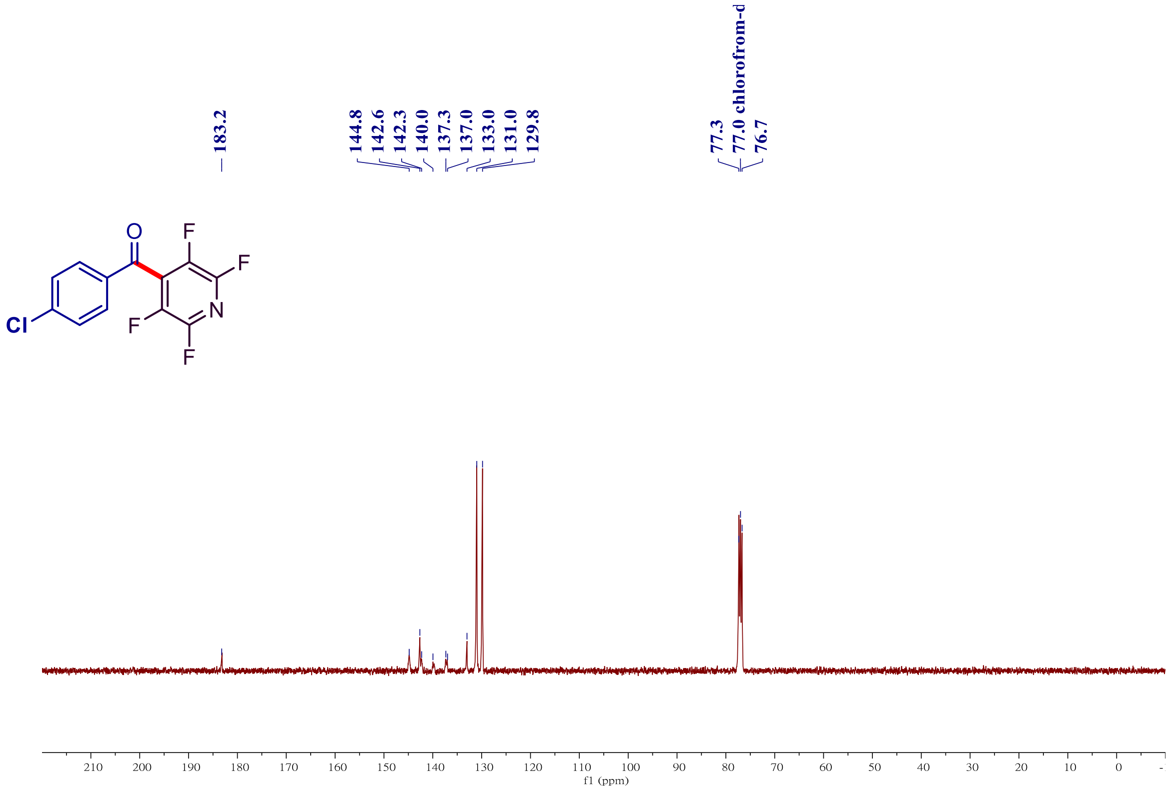

[Back to the contents](#)

^{19}F NMR (282 MHz, $CDCl_3$) of **3g**

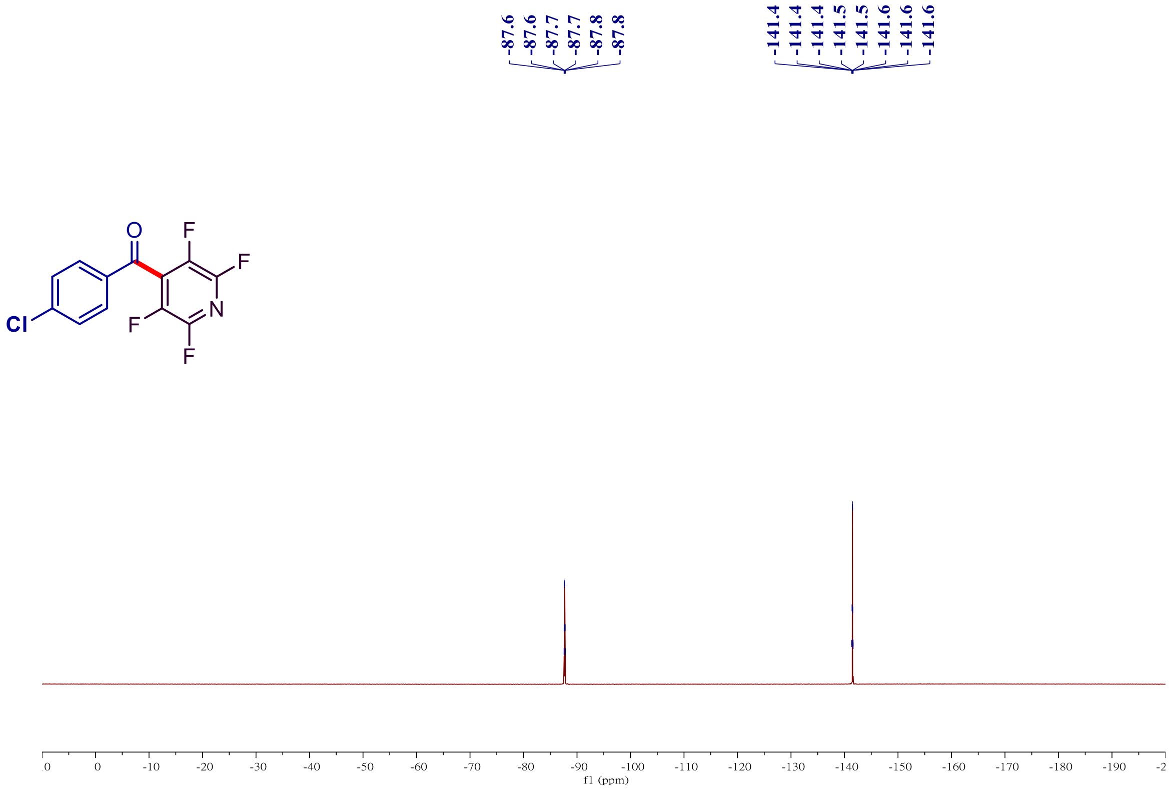

^{1}H NMR (300 MHz, $CDCl_3$) of **3h**, [See procedure](#)

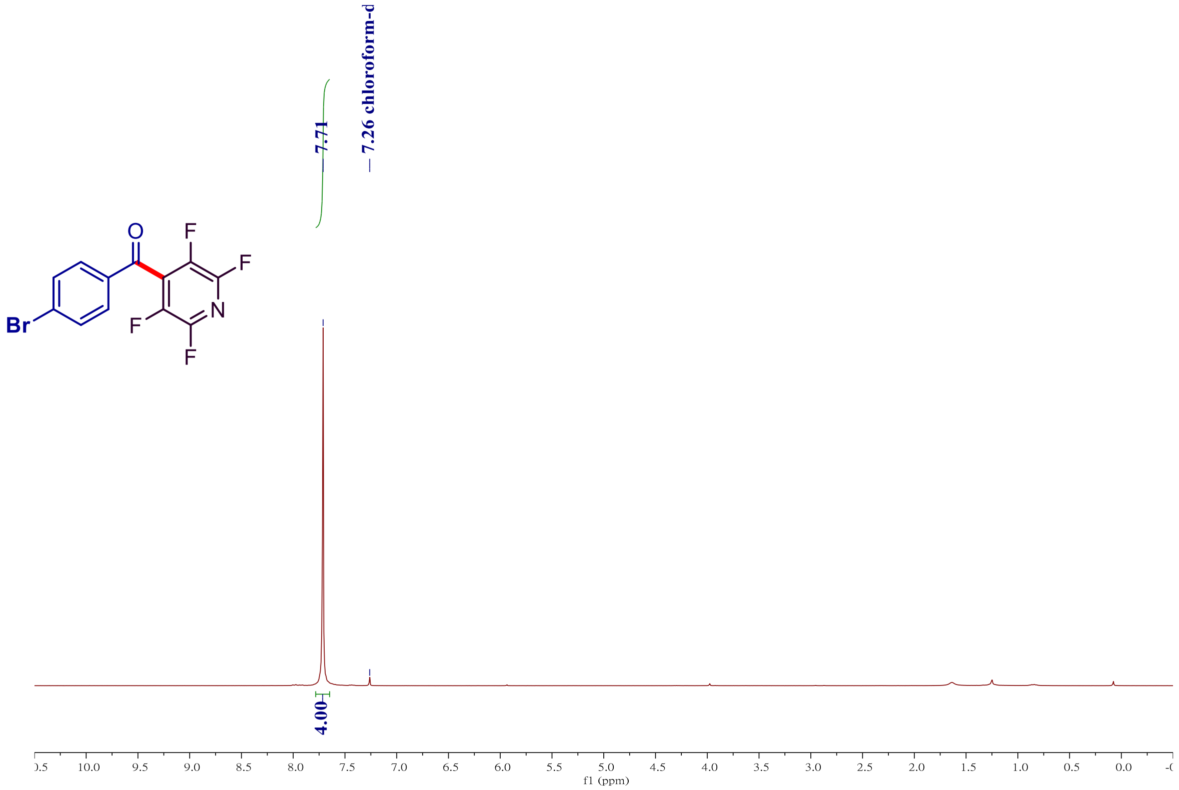

[Back to the contents](#)

^{13}C NMR (101 MHz, $CDCl_3$) of **3h**

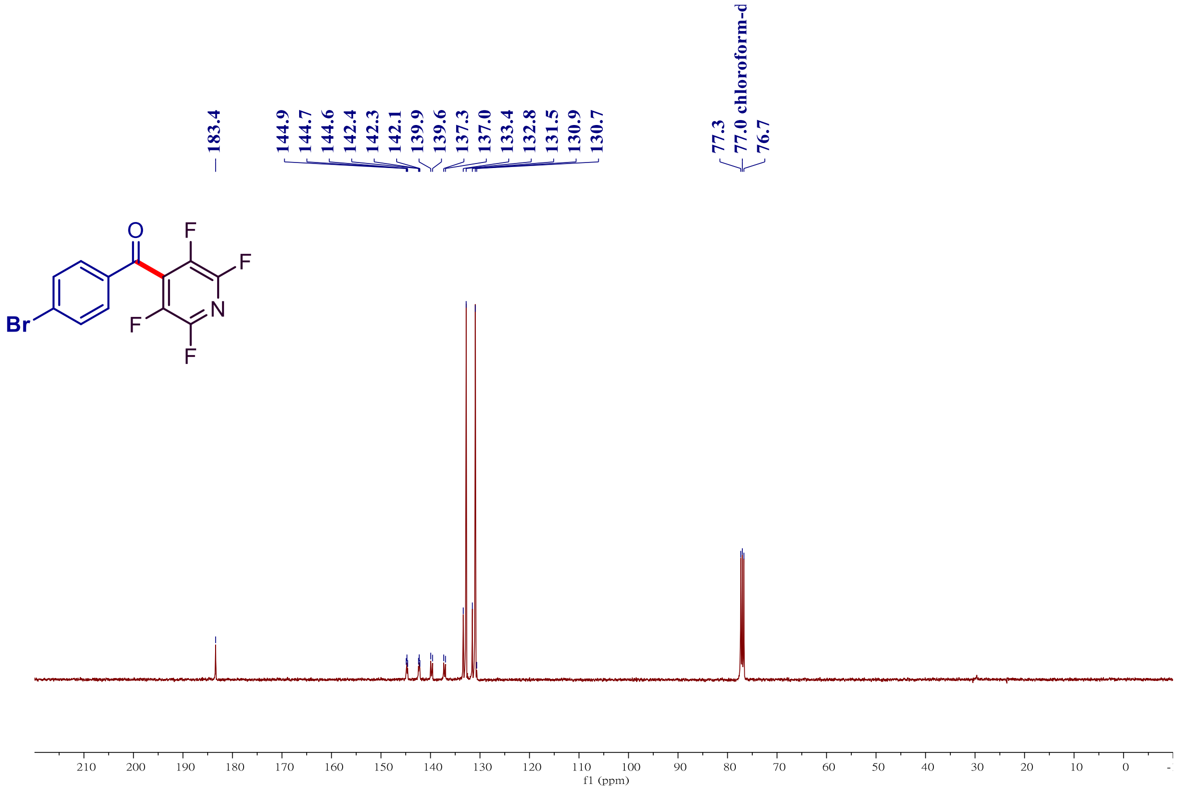

^{19}F NMR (282 MHz, $CDCl_3$) of **3h**

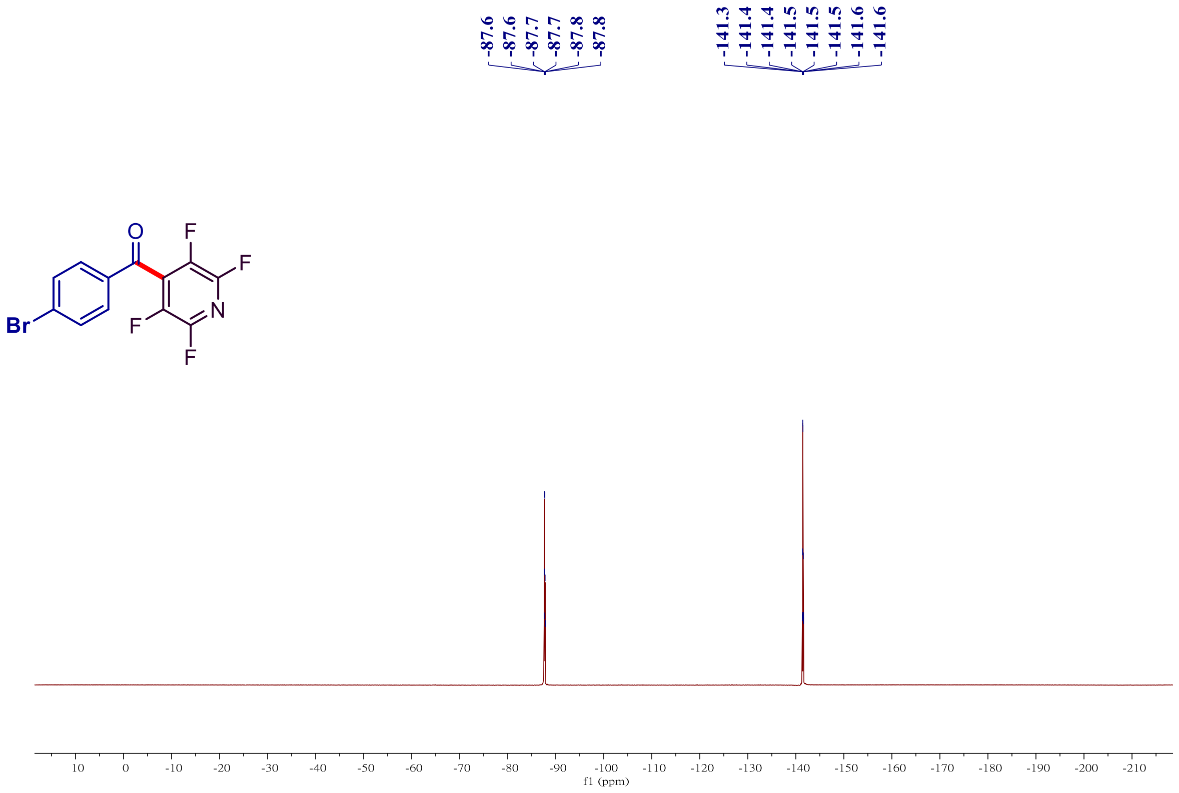

Back to the contents

¹H NMR (300 MHz, $CDCl_3$) of **3i**, See procedure

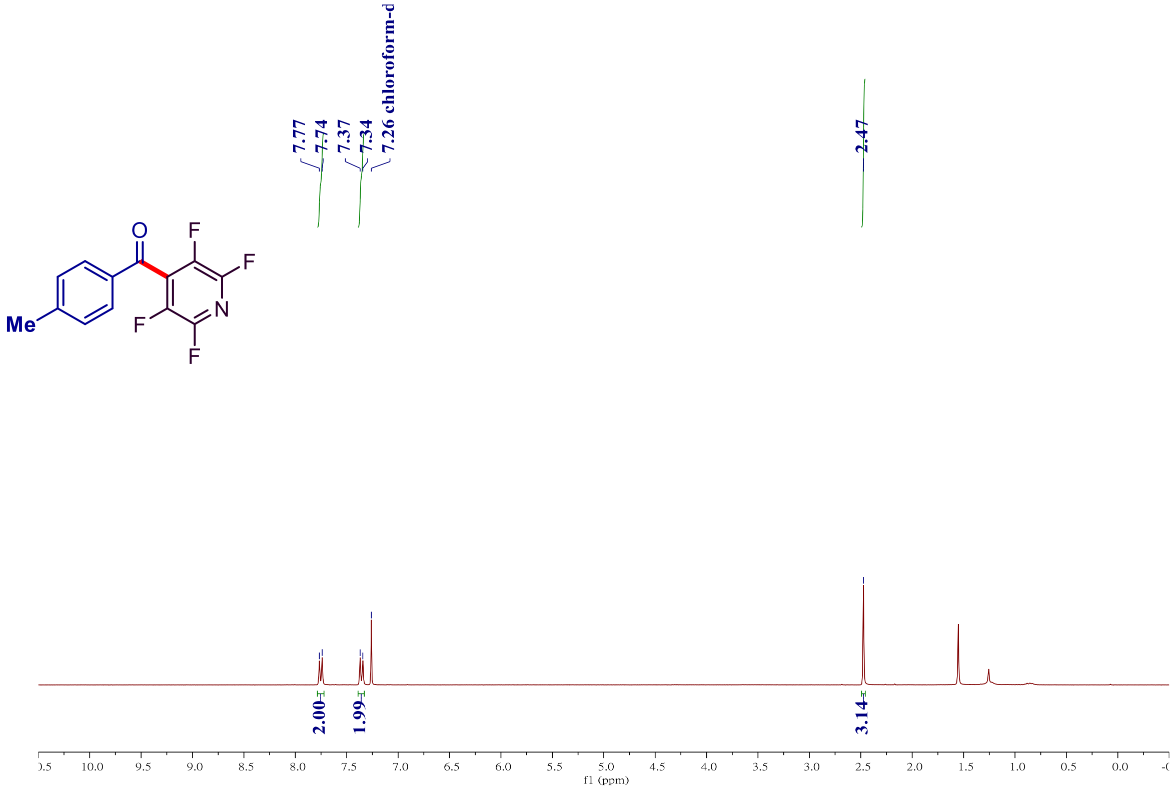

¹³C NMR (101 MHz, $CDCl_3$) of **3i**

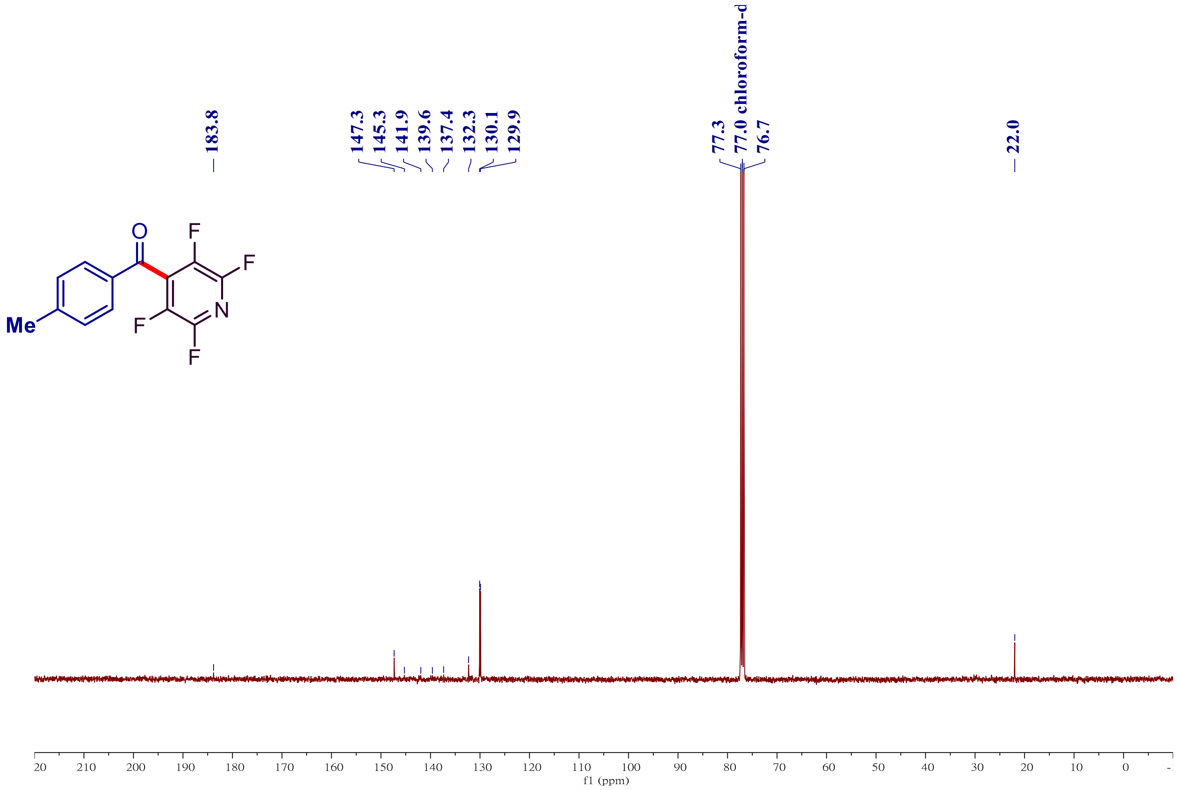

[Back to the contents]

^{19}F NMR (282 MHz, $CDCl_3$) of **3i**

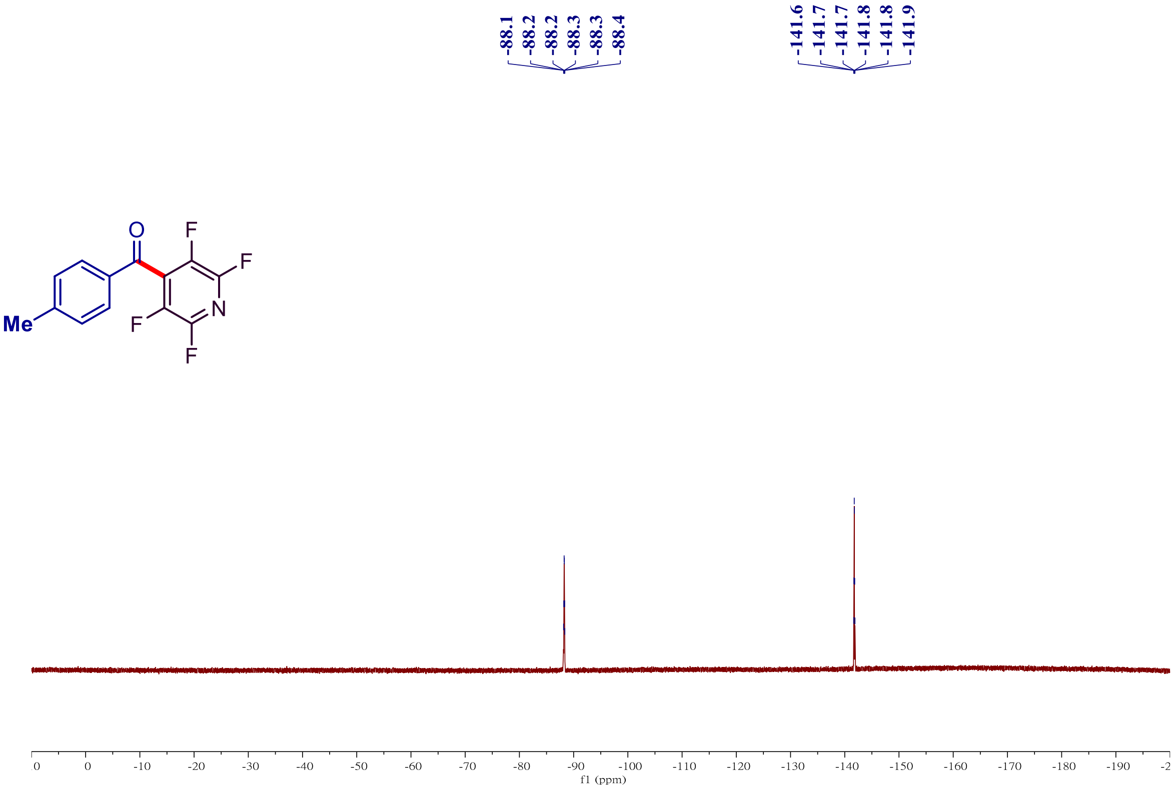

^{1}H NMR (400 MHz, $CDCl_3$) of **3j**, See procedure

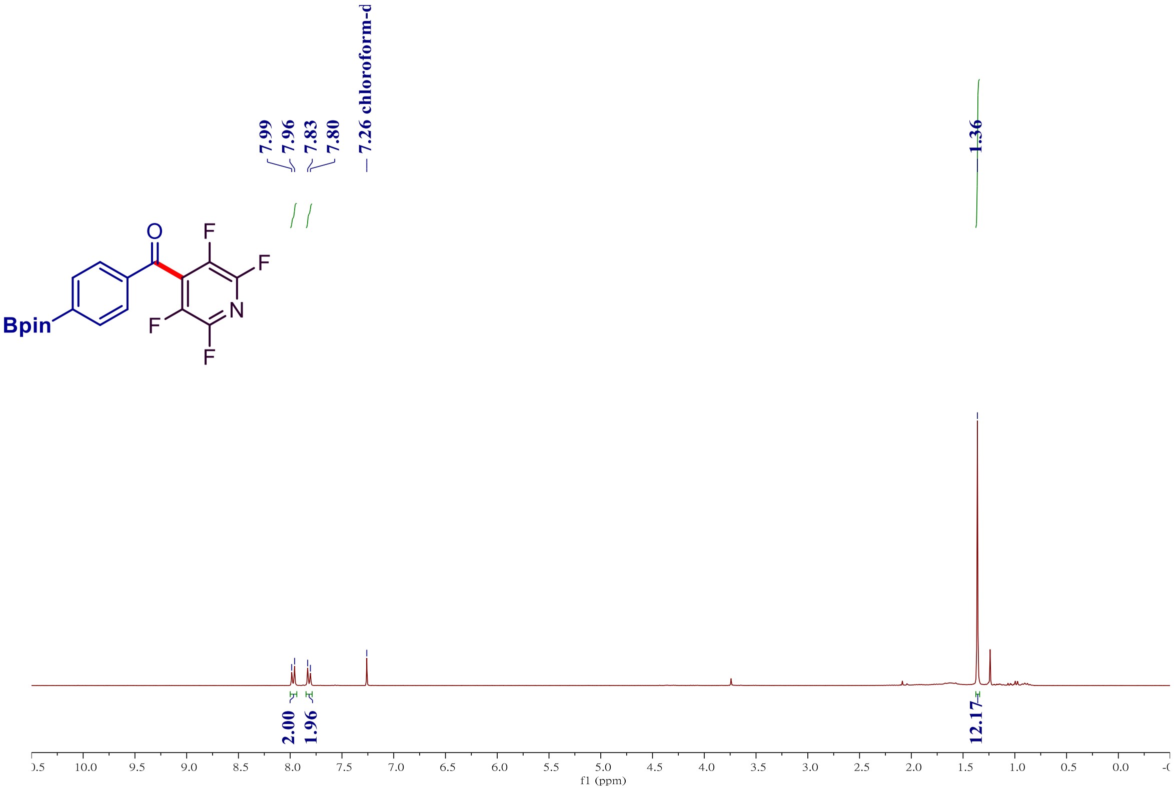

Back to the contents

^{13}C NMR (101 MHz, $CDCl_3$) of **3j**

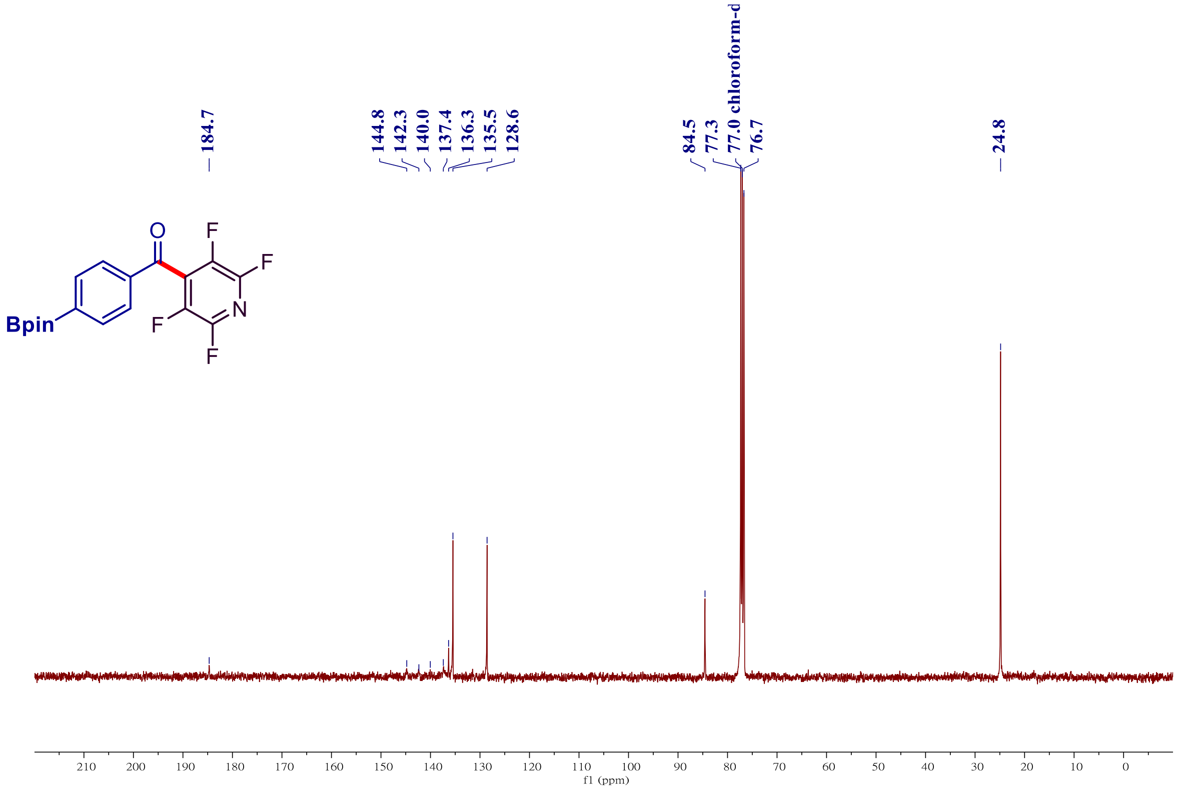

^{19}F NMR (282 MHz, $CDCl_3$) of **3j**

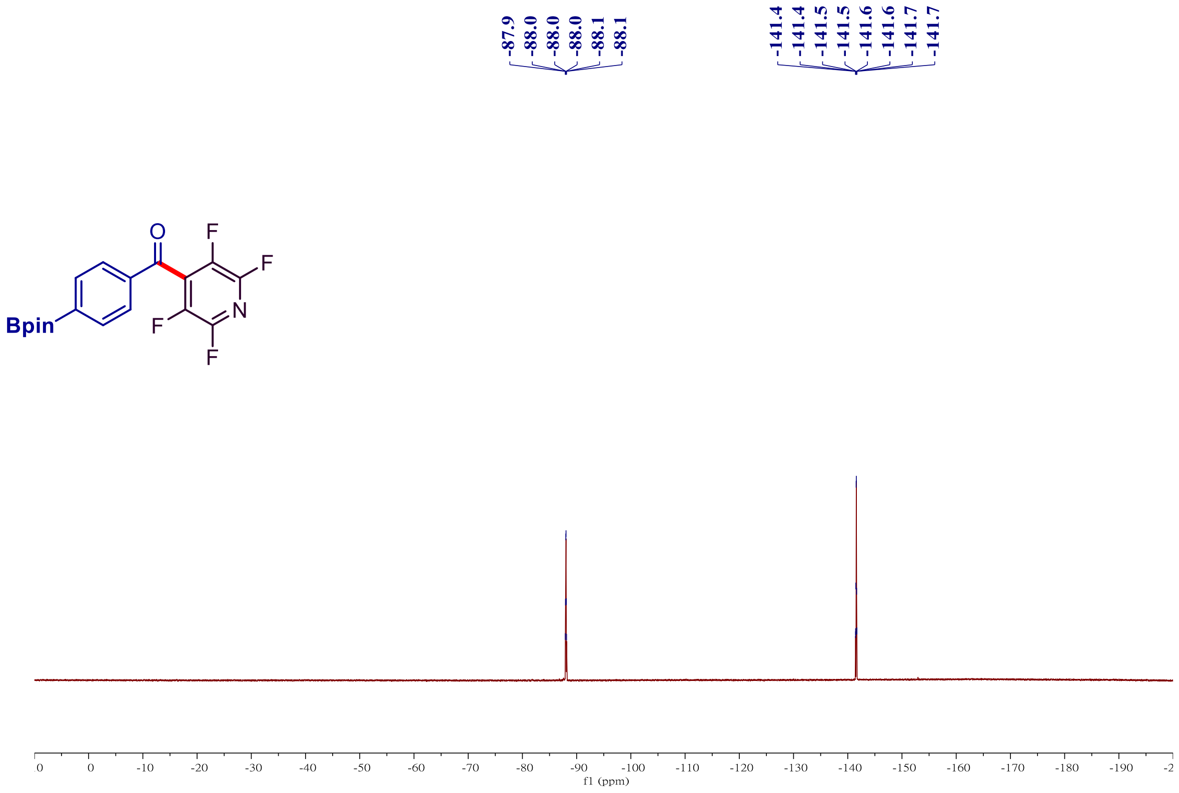

Back to the contents

^{1}H NMR (300 MHz, $CDCl_3$) of **3k**, *See procedure*

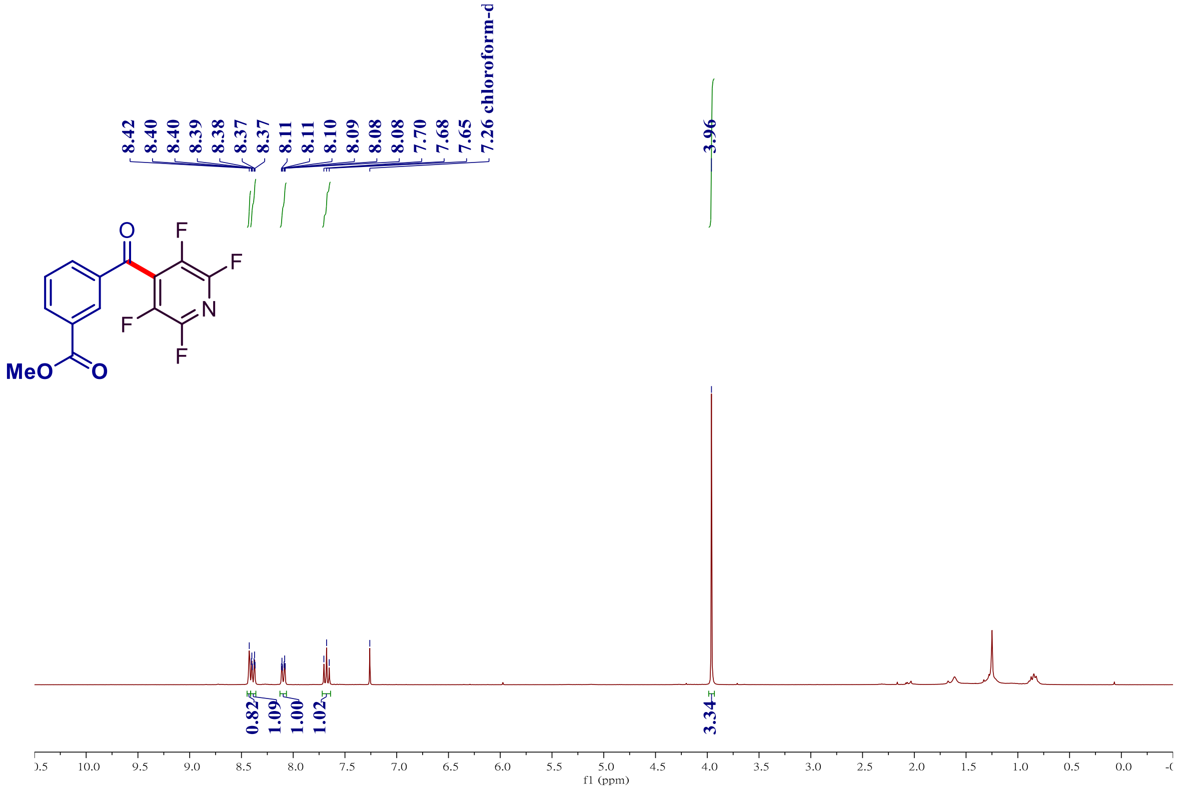

^{13}C NMR (101 MHz, $CDCl_3$) of **3k**

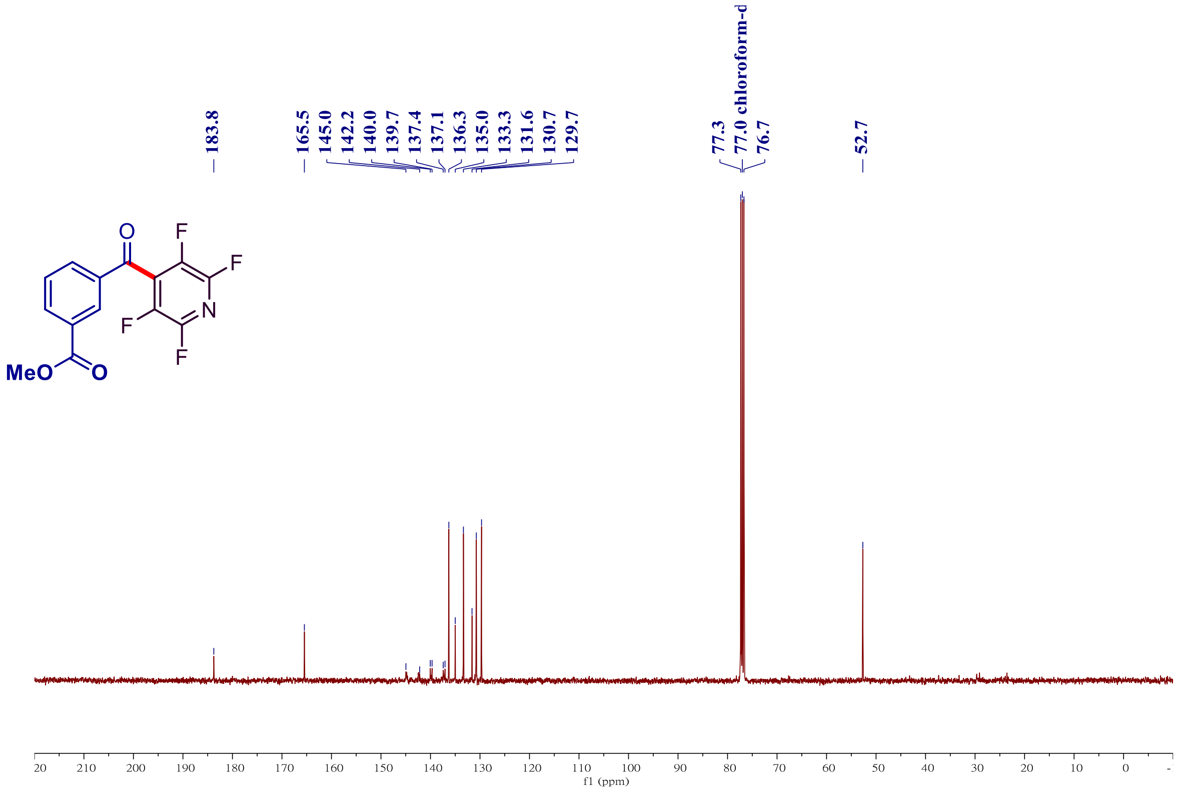

Back to the contents

^{19}F NMR (282 MHz, $CDCl_3$) of **3k**

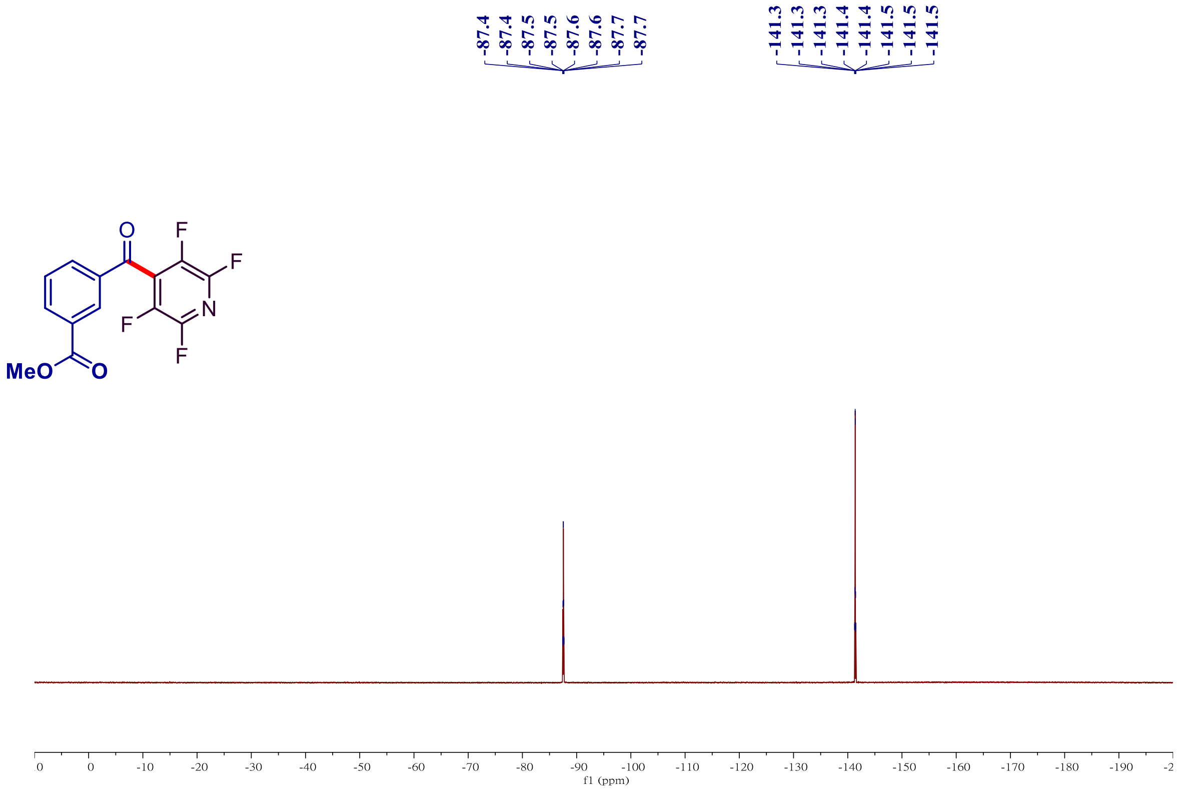

^{1}H NMR (300 MHz, $CDCl_3$) of **3l**, See procedure

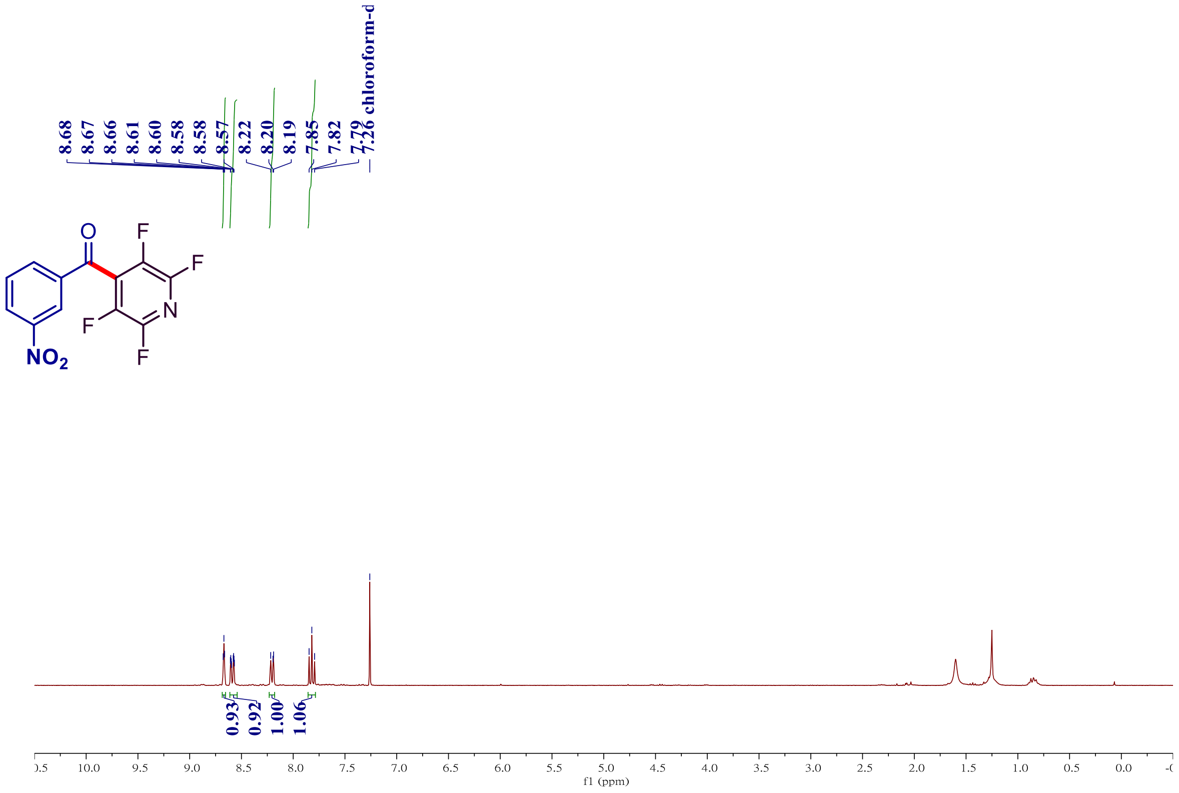

Back to the contents

^{13}C NMR (101 MHz, $CDCl_3$) of **3l**

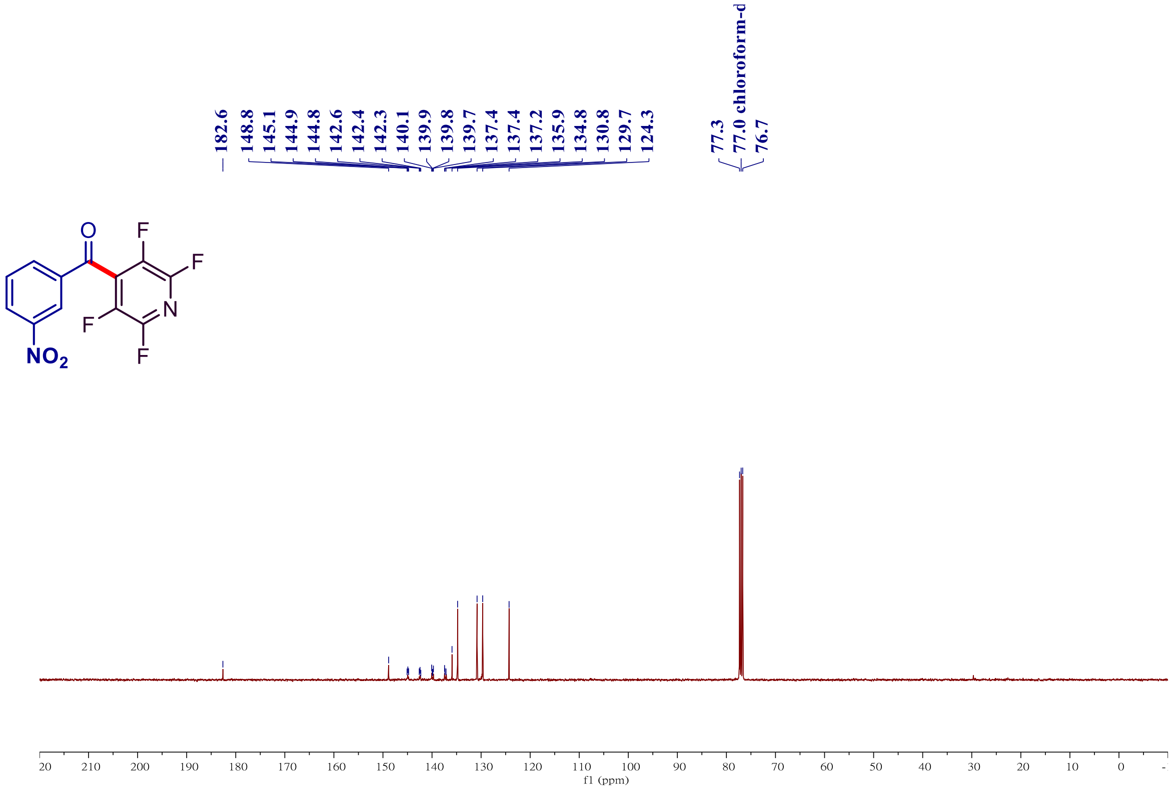

^{19}F NMR (282 MHz, $CDCl_3$) of **3l**

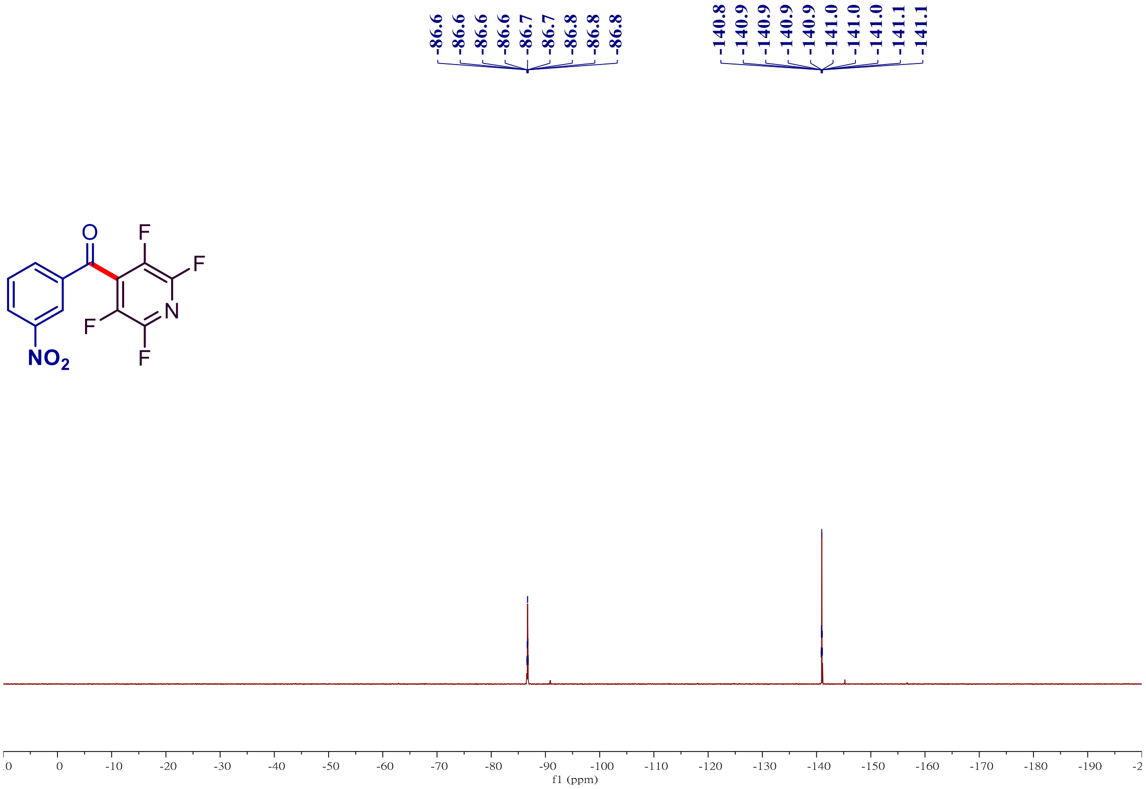

[Back to the contents]

^{1}H NMR (300 MHz, $CDCl_3$) of **3m**, See procedure

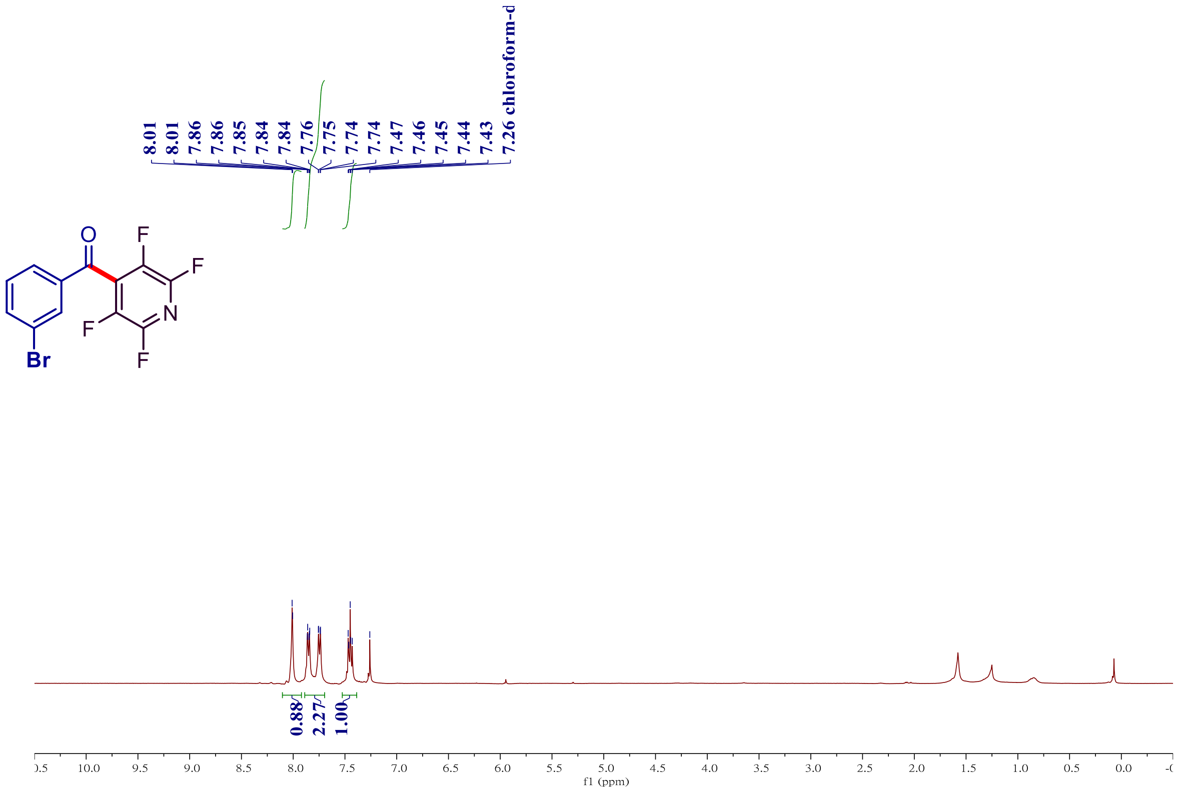

^{13}C NMR (101 MHz, $CDCl_3$) of **3m**

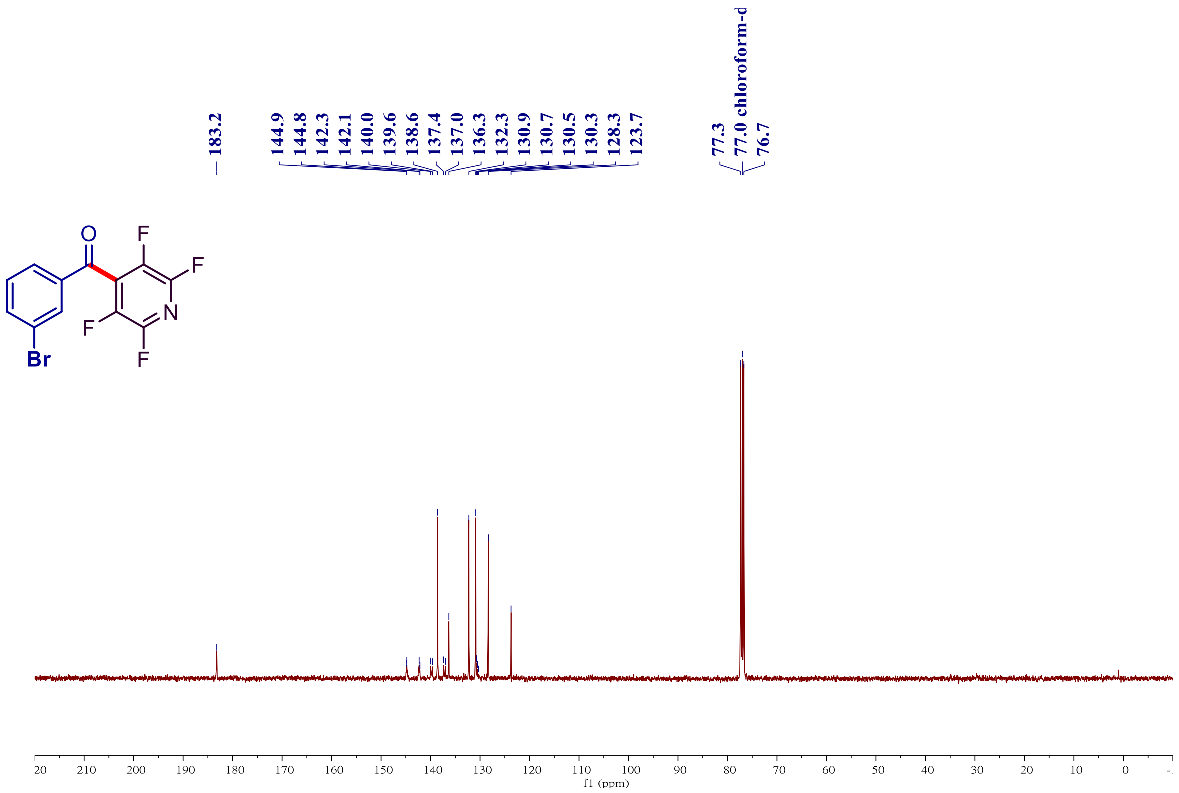

Back to the contents

^{19}F NMR (282 MHz, $CDCl_3$) of **3m**

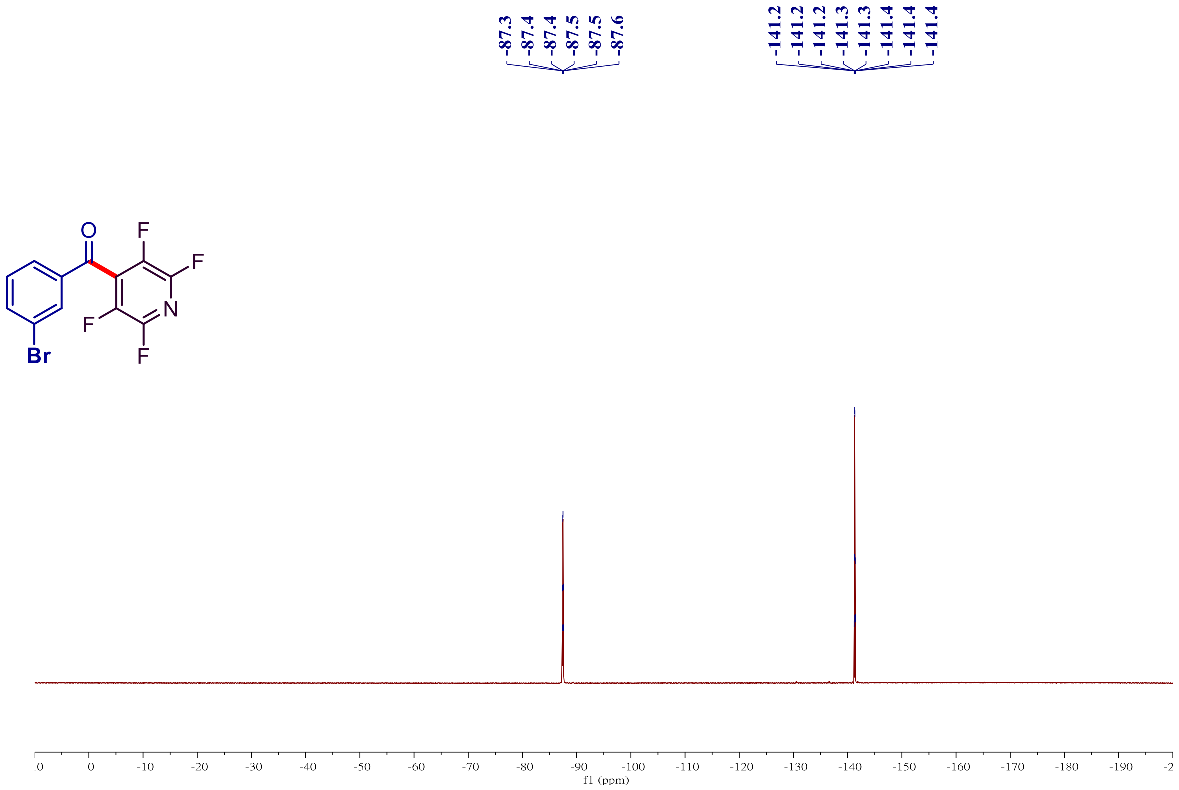

^{1}H NMR (300 MHz, $CDCl_3$) of **3n**, See procedure

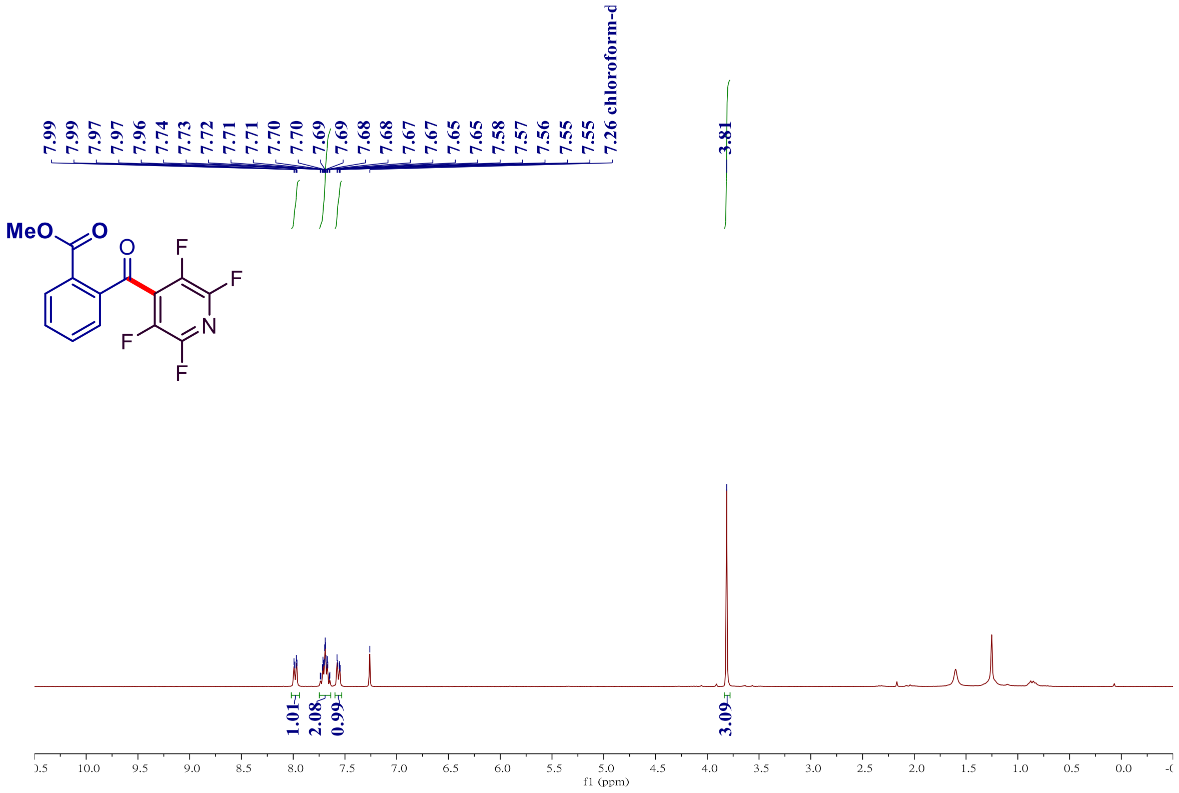

Back to the contents

^{13}C NMR (101 MHz, $CDCl_3$) of **3n**

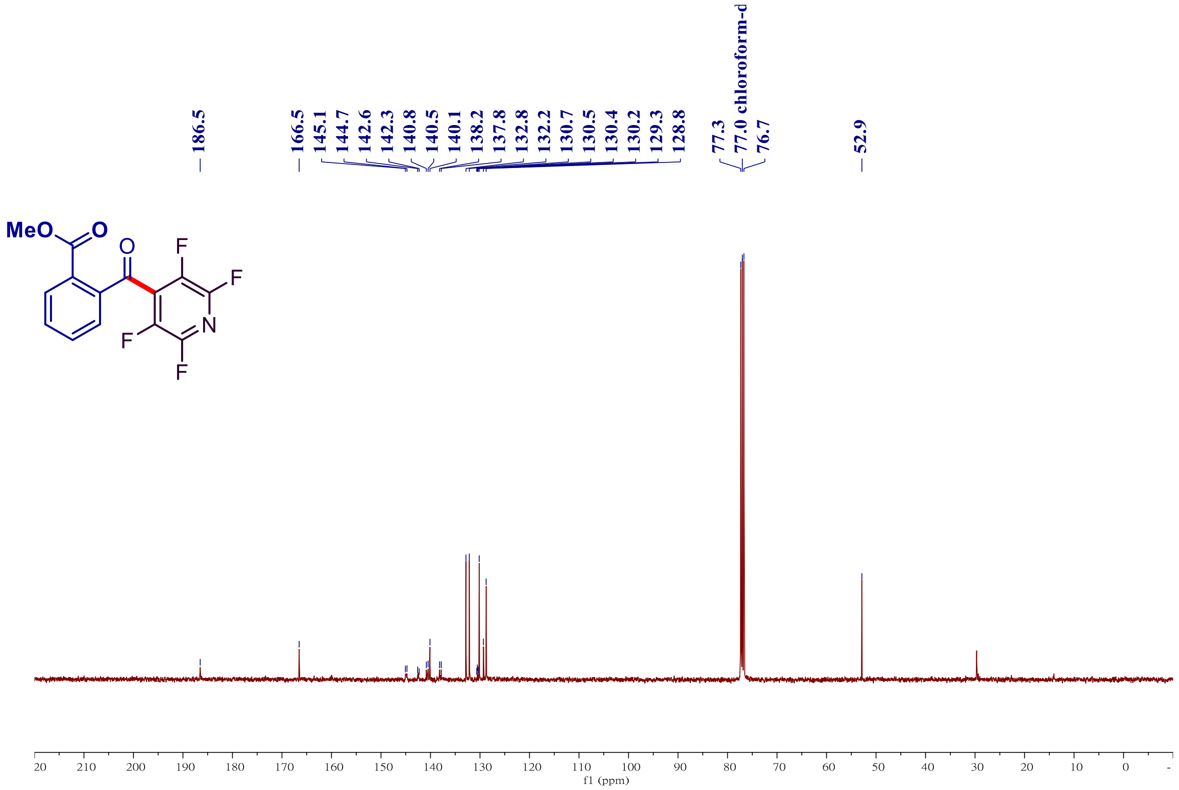

^{19}F NMR (282 MHz, $CDCl_3$) of **3n**

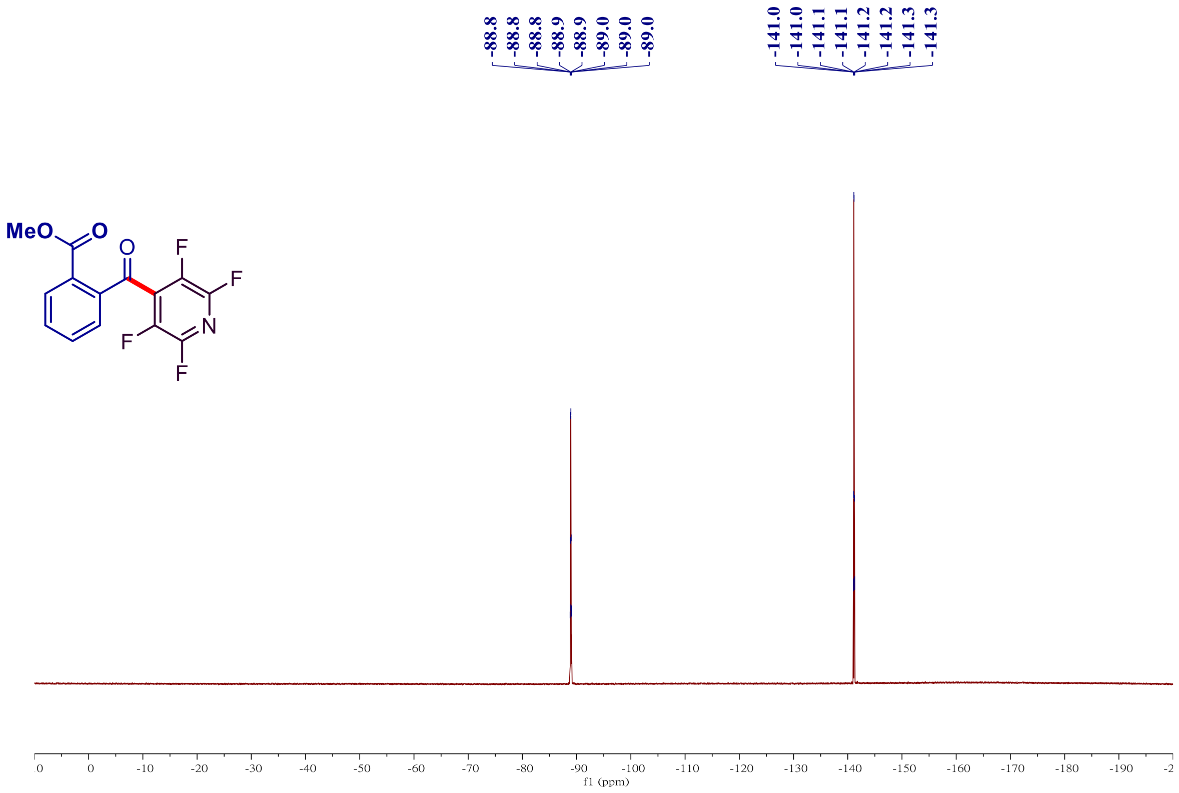

Back to the contents

^{1}H NMR (300 MHz, $CDCl_3$) of **3o**, See procedure

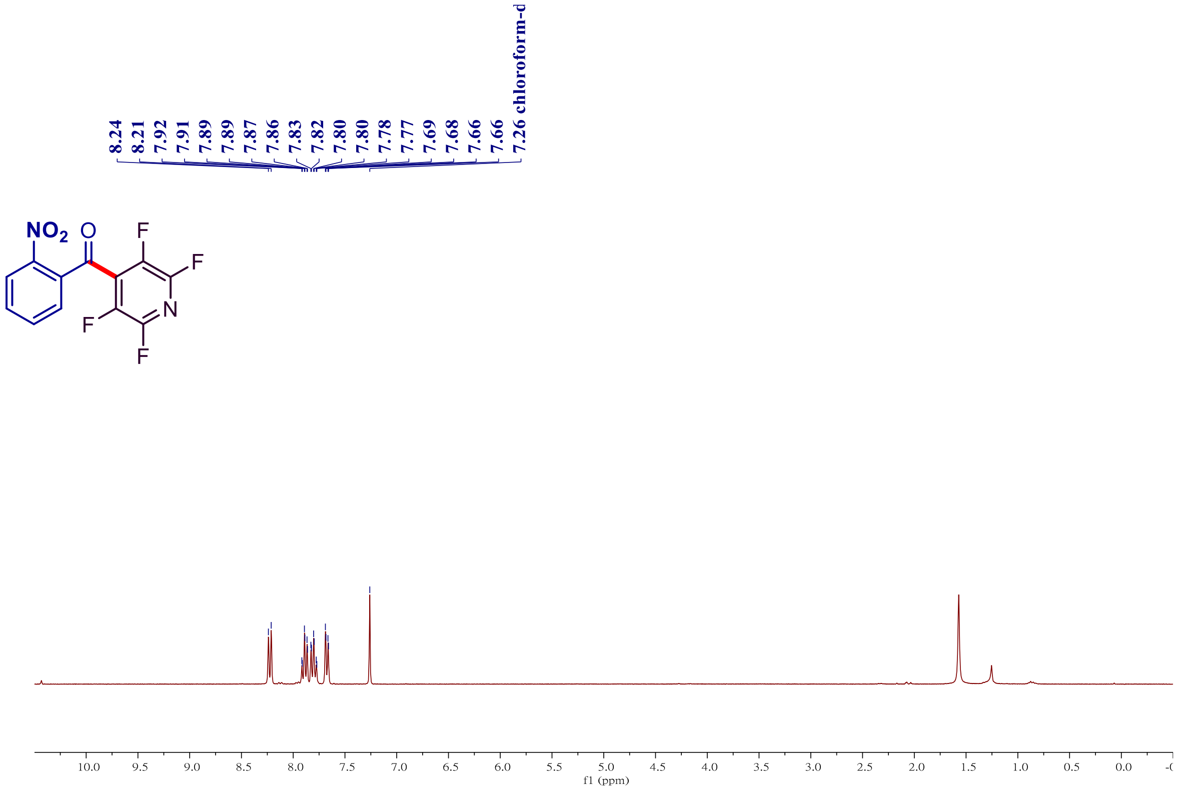

^{13}C NMR (101 MHz, $CDCl_3$) of **3o**

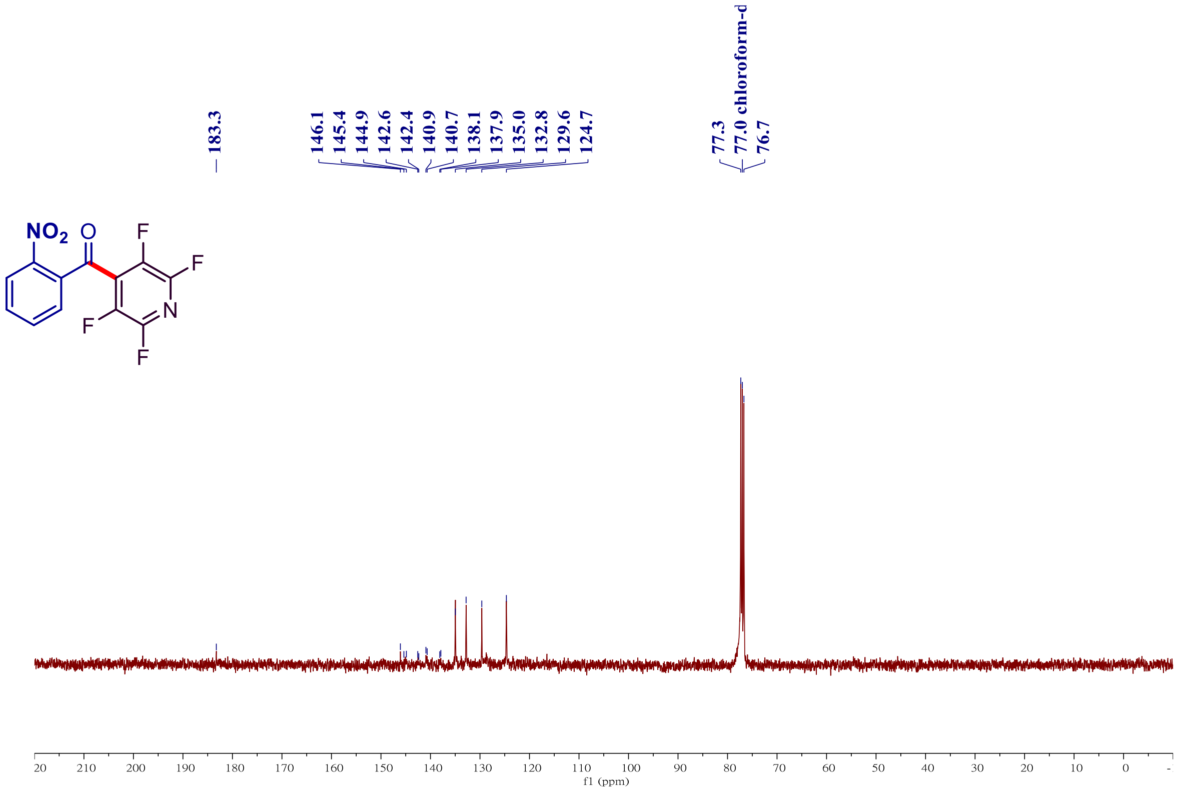

[Back to the contents]

^{19}F NMR (282 MHz, $CDCl_3$) of **3o**

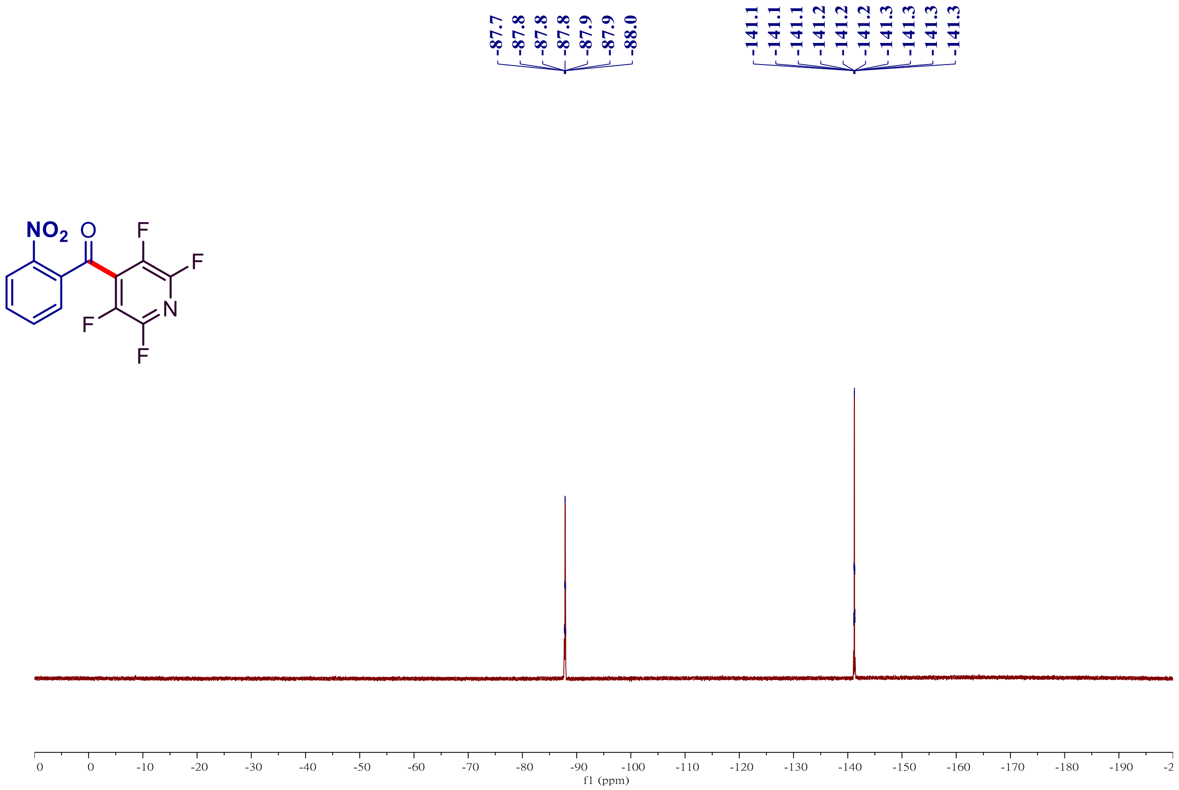

^{1}H NMR (300 MHz, $CDCl_3$) of **3p**, See procedure

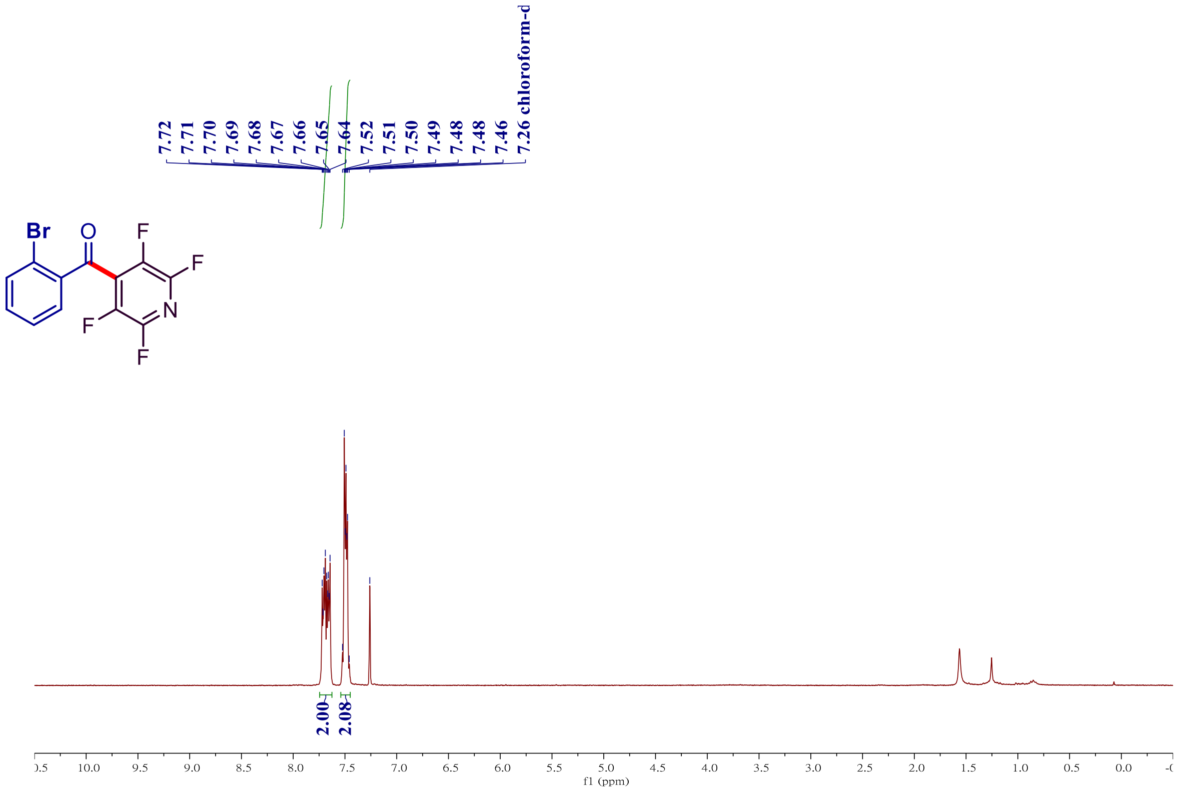

Back to the contents

^{13}C NMR (101 MHz, $CDCl_3$) of **3p**

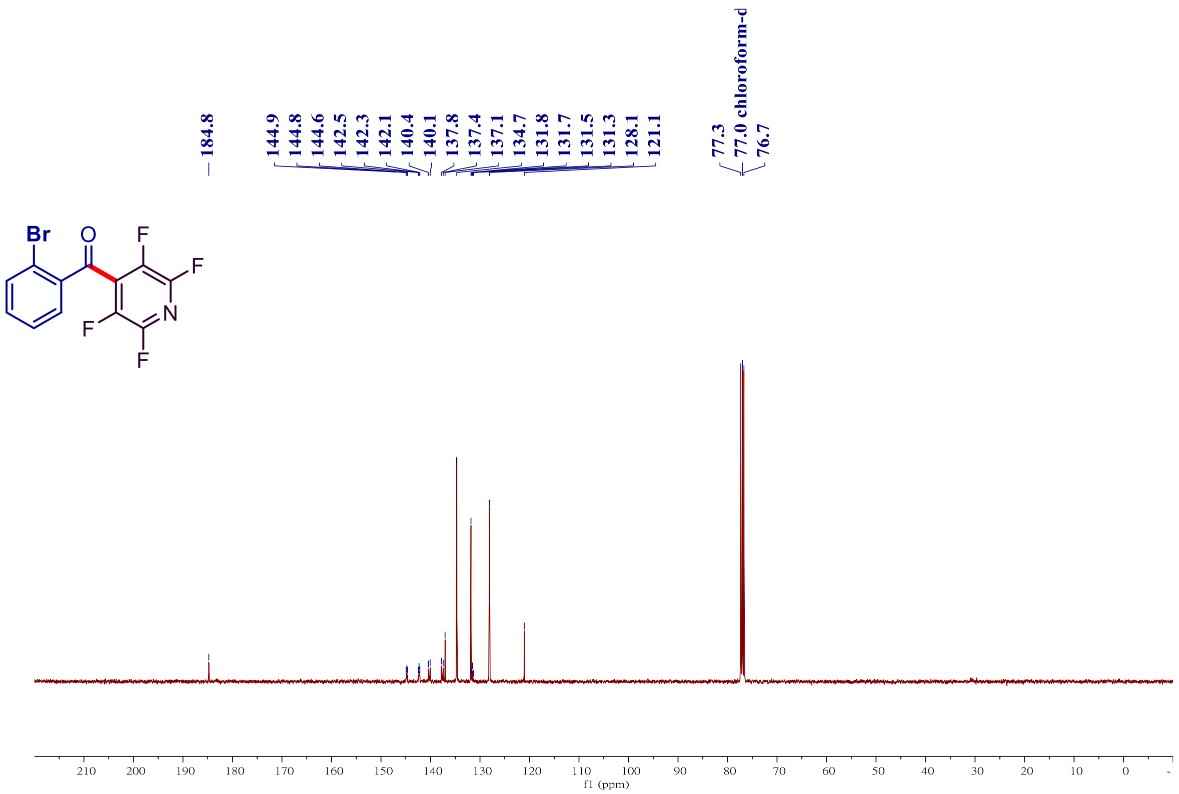

^{19}F NMR (282 MHz, $CDCl_3$) of **3p**

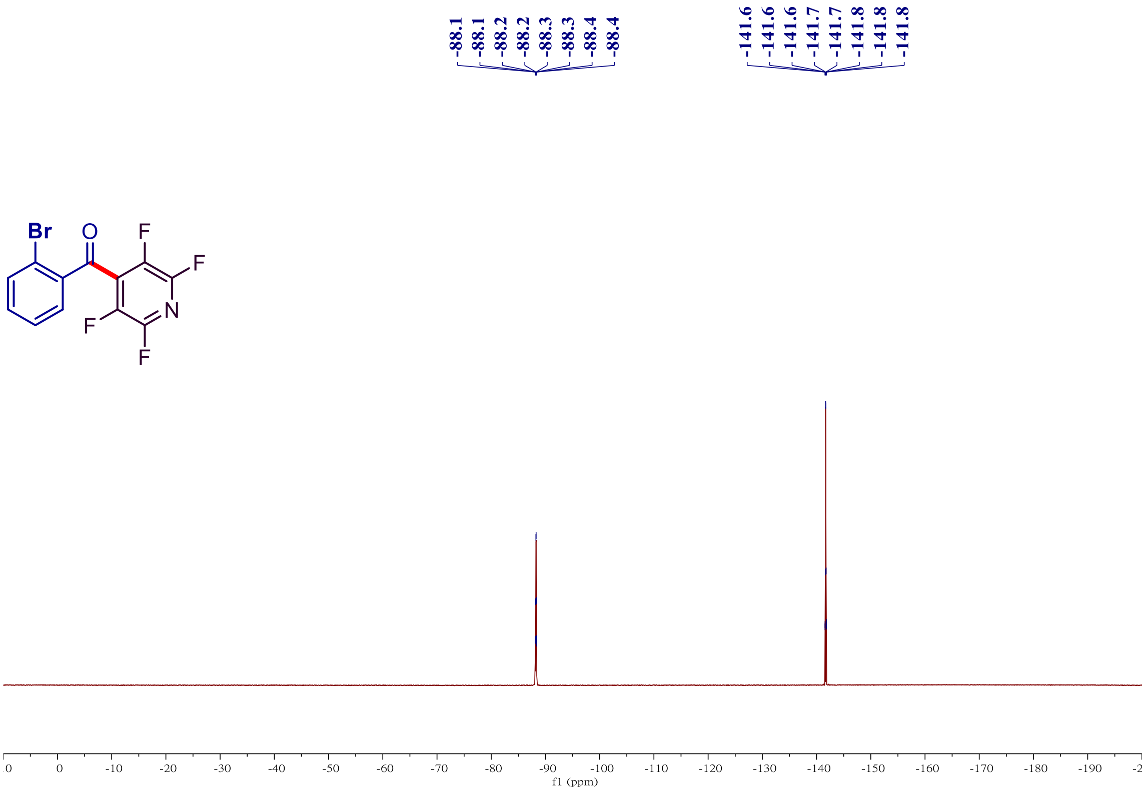

[Back to the contents](#)

^{1}H NMR (300 MHz, $CDCl_3$) of **3q**, See procedure

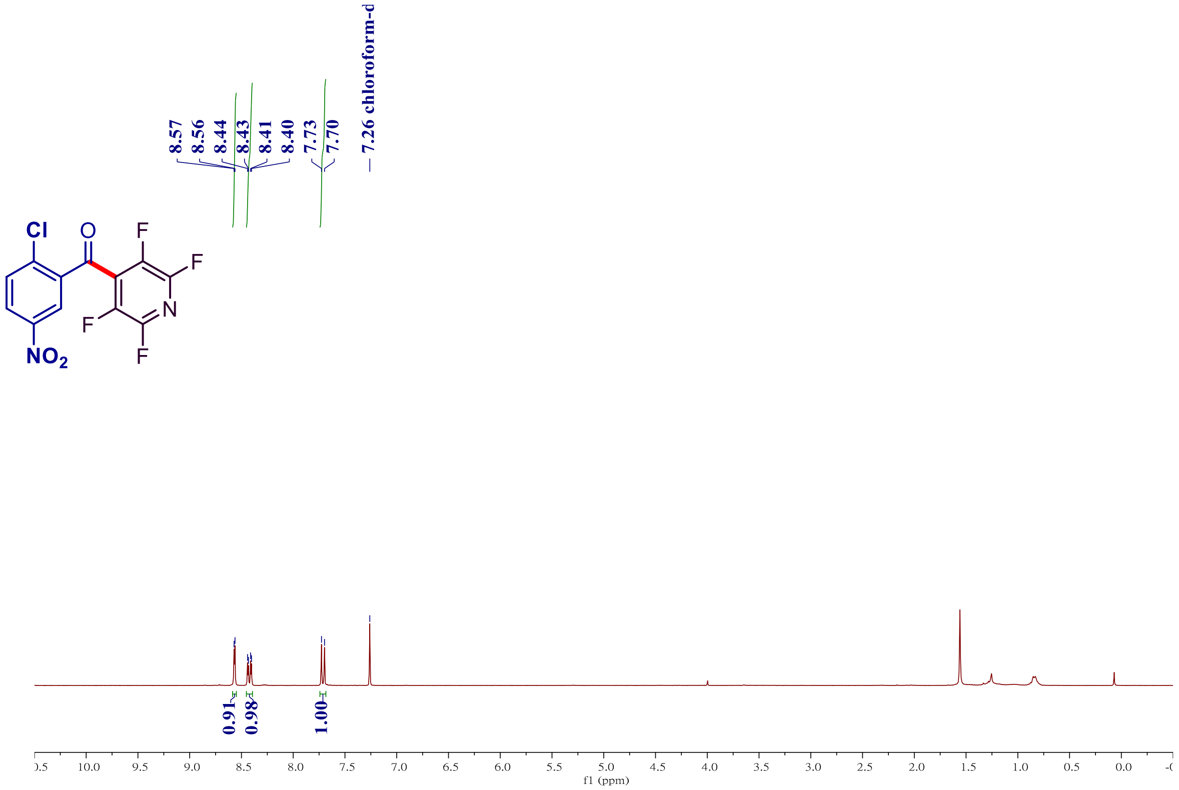

^{13}C NMR (101 MHz, $CDCl_3$) of **3q**

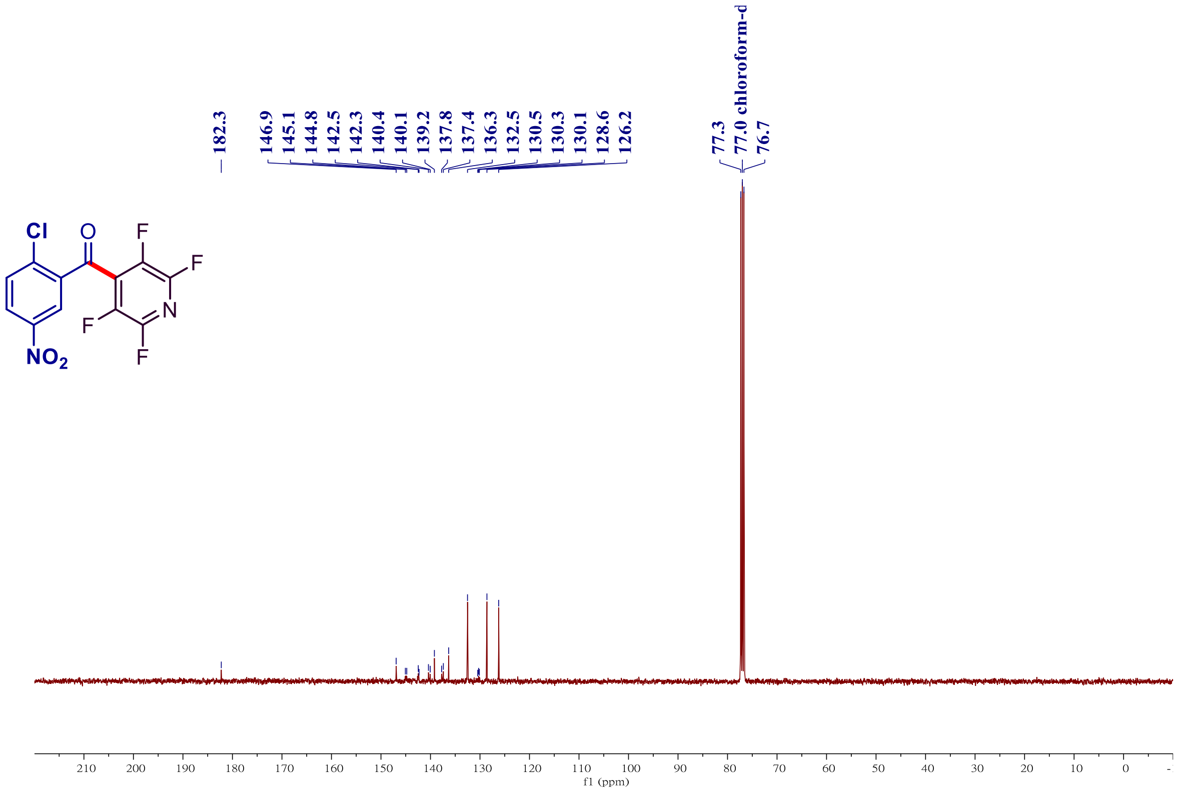

^{19}F NMR (282 MHz, $CDCl_3$) of **3q**

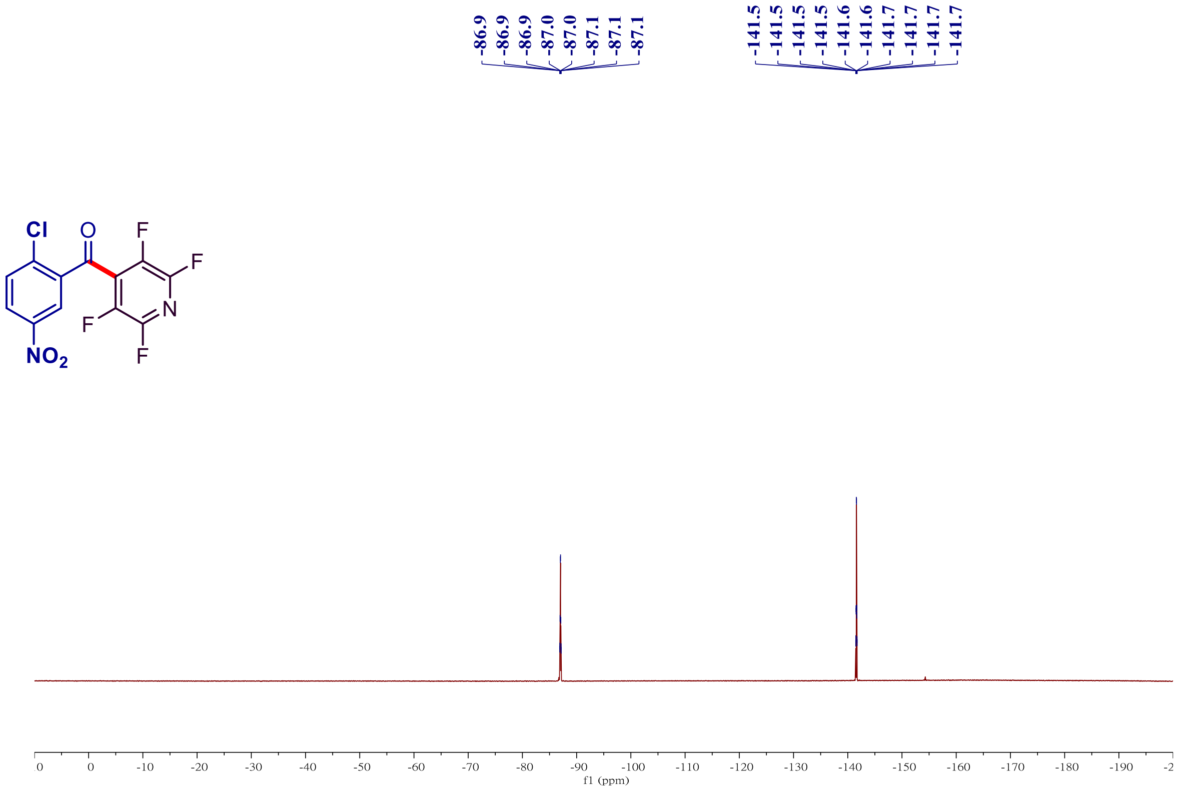

^{1}H NMR (300 MHz, $CDCl_3$) of **3r**, [See procedure](#)

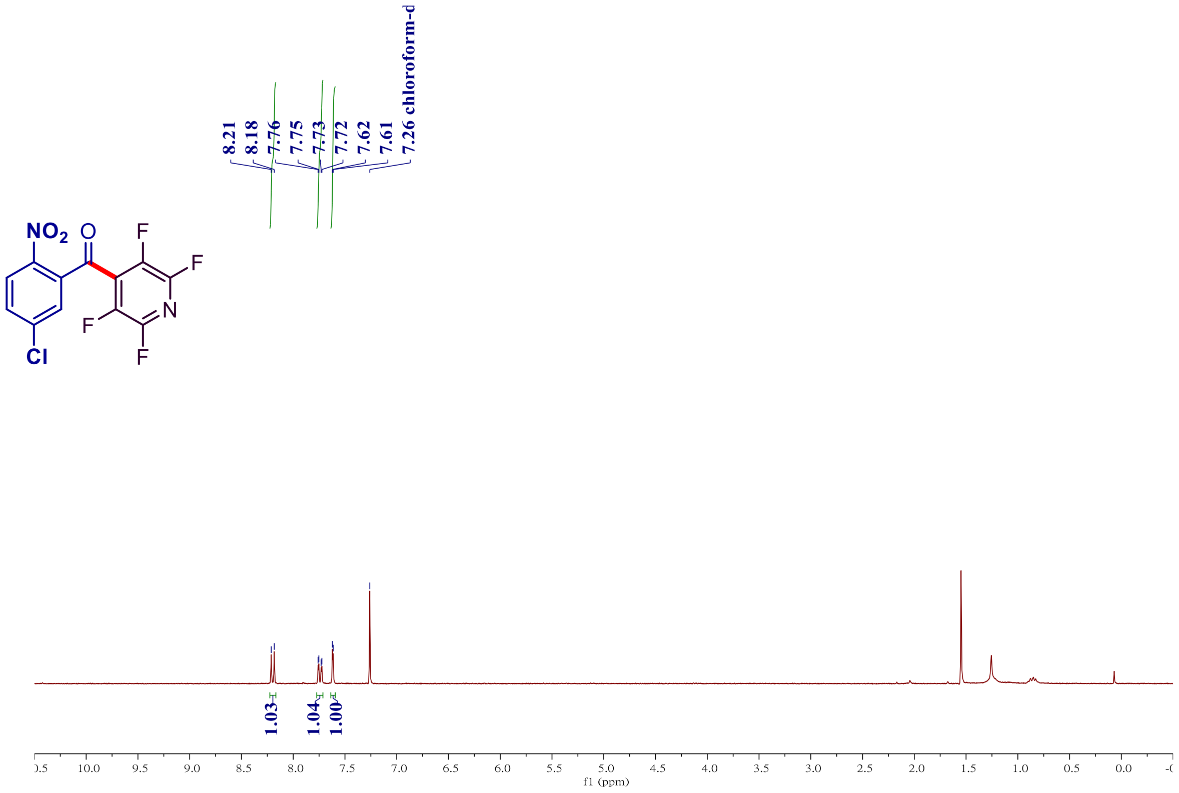

Back to the contents

^{13}C NMR (101 MHz, $CDCl_3$) of **3r**

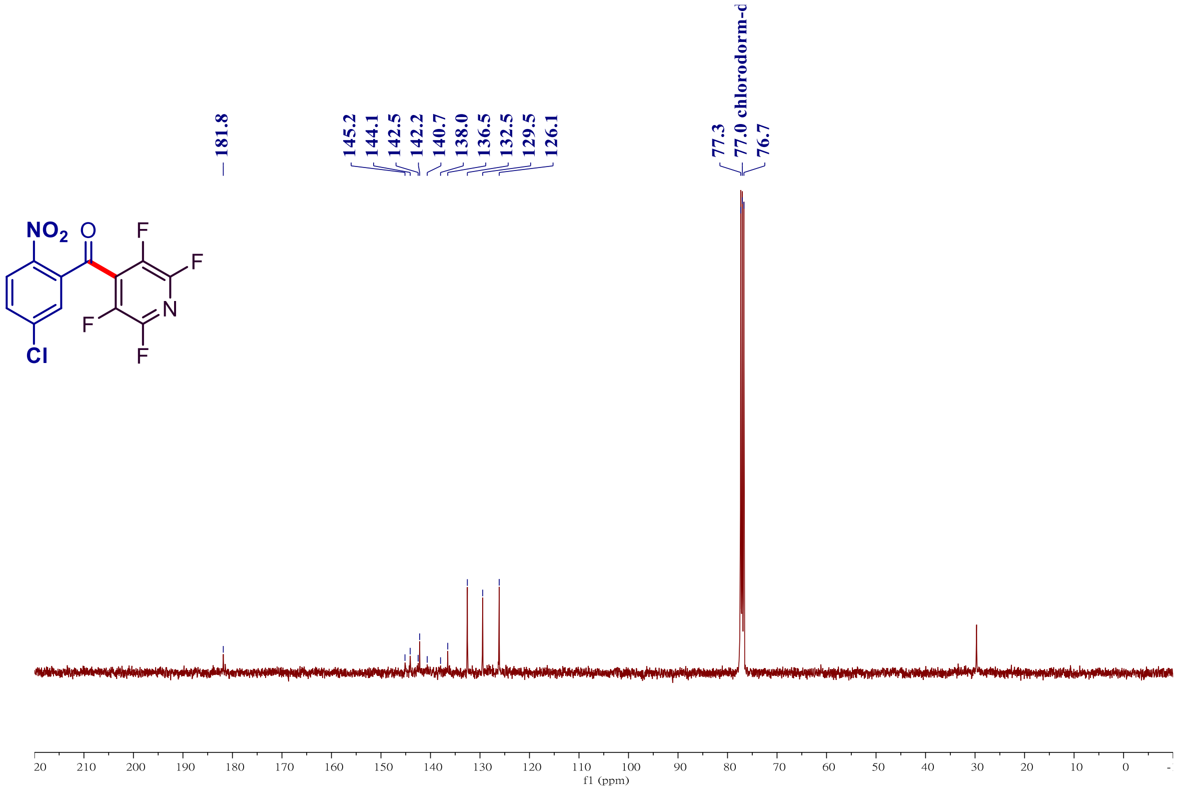

^{19}F NMR (282 MHz, $CDCl_3$) of **3r**

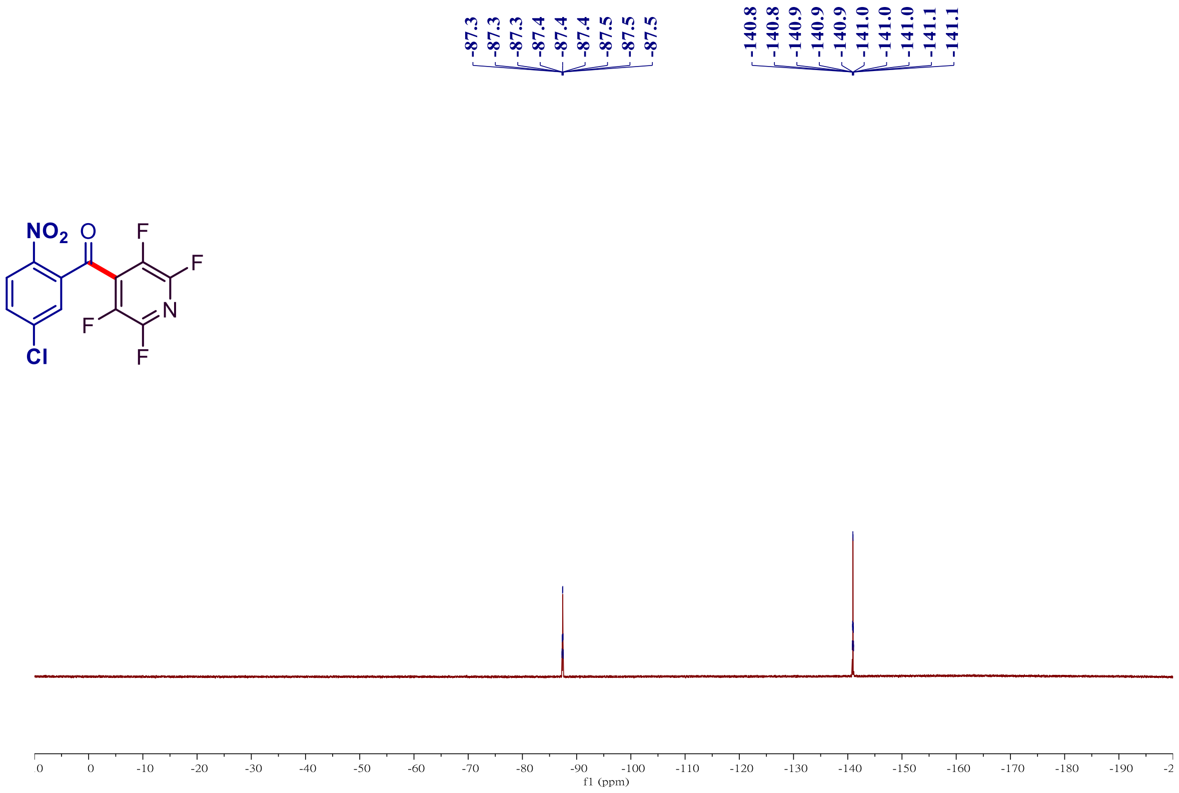

Back to the contents

^{1}H NMR (300 MHz, $CDCl_3$) of **3s**, See procedure

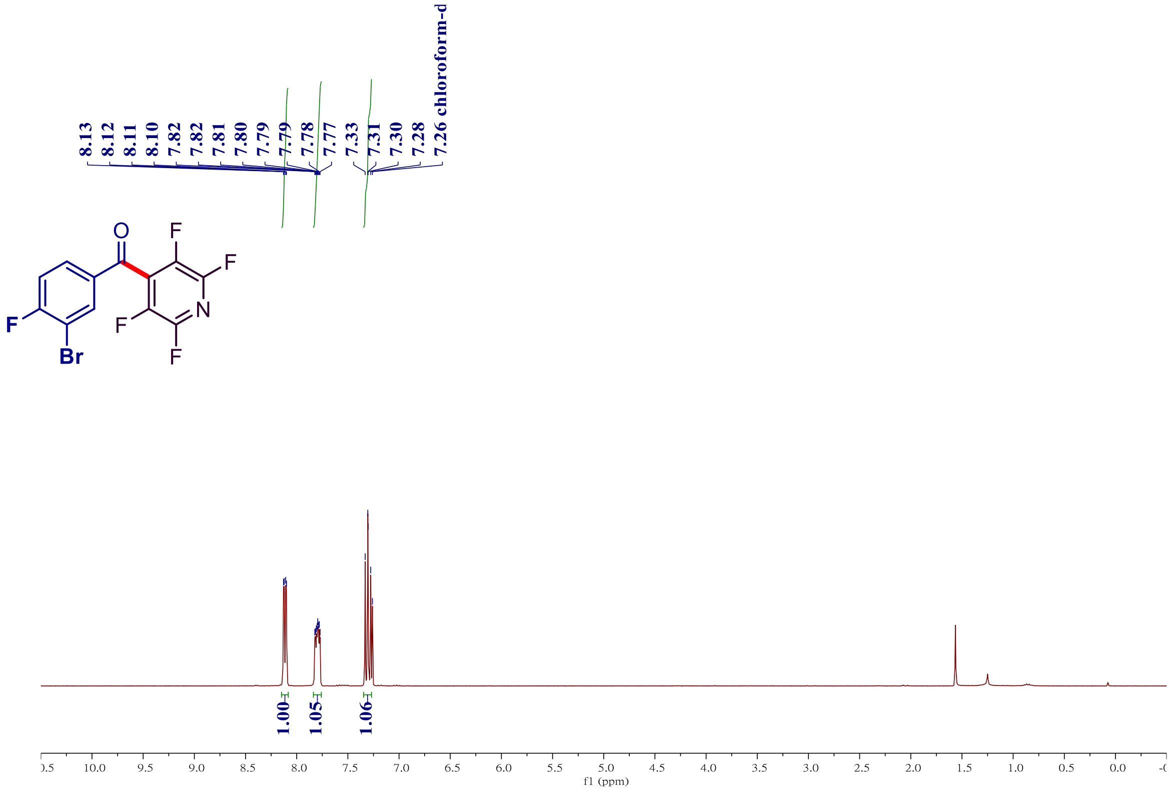

^{13}C NMR (101 MHz, $CDCl_3$) of **3s**

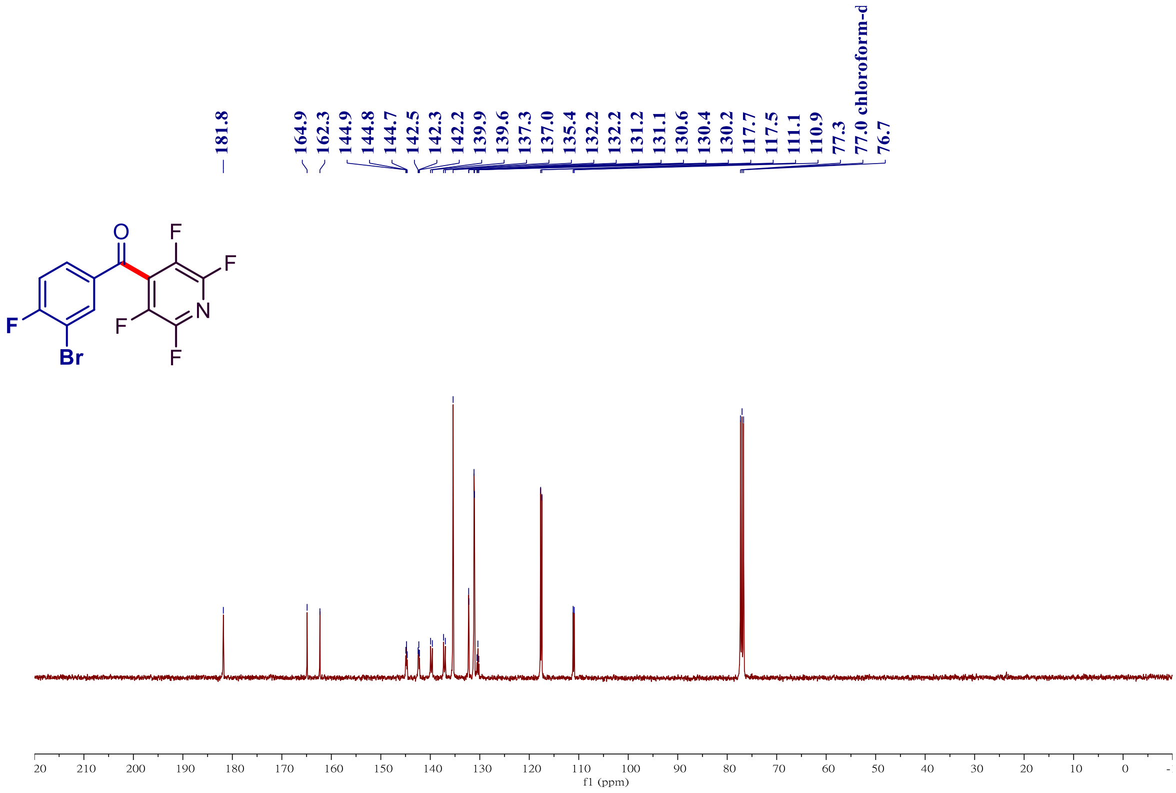

[Back to the contents](#)

19F NMR (282 MHz, $CDCl_3$) of **3s**

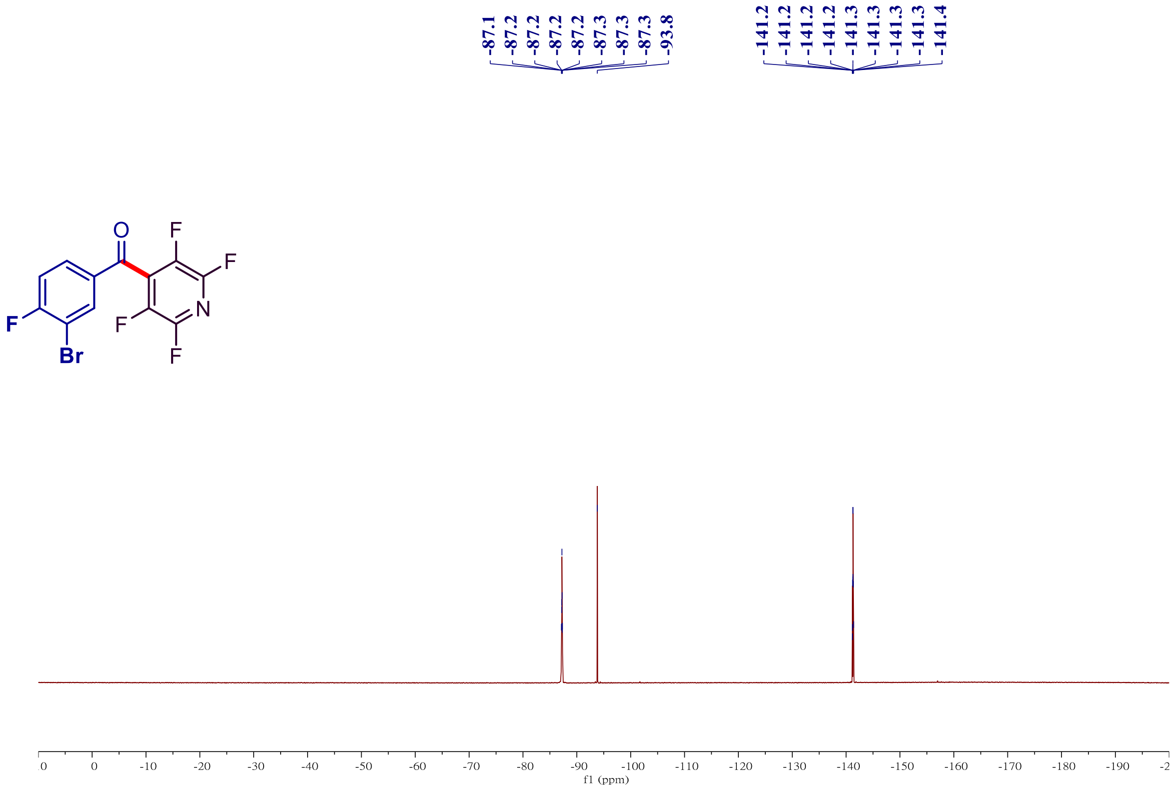

1H NMR (300 MHz, $CDCl_3$) of **3t**, [See procedure](#)

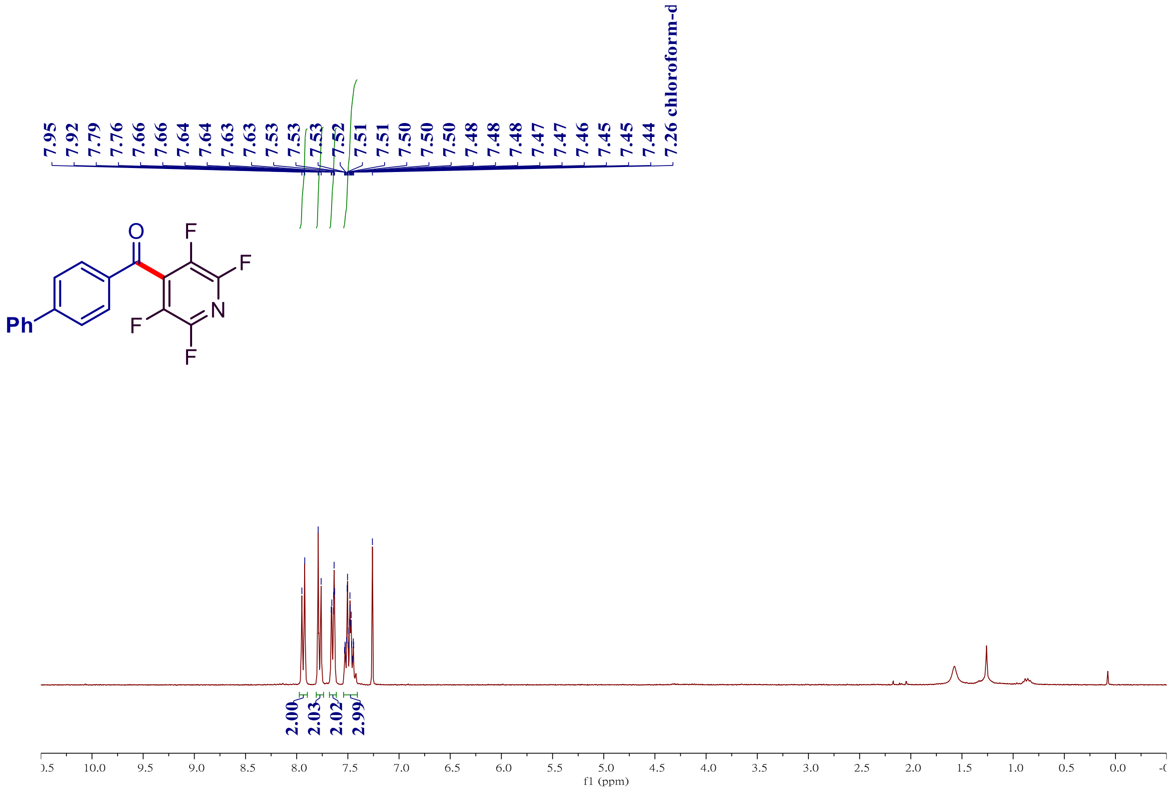

Back to the contents

^{13}**C NMR** (101 MHz, $CDCl_3$) of **3t**

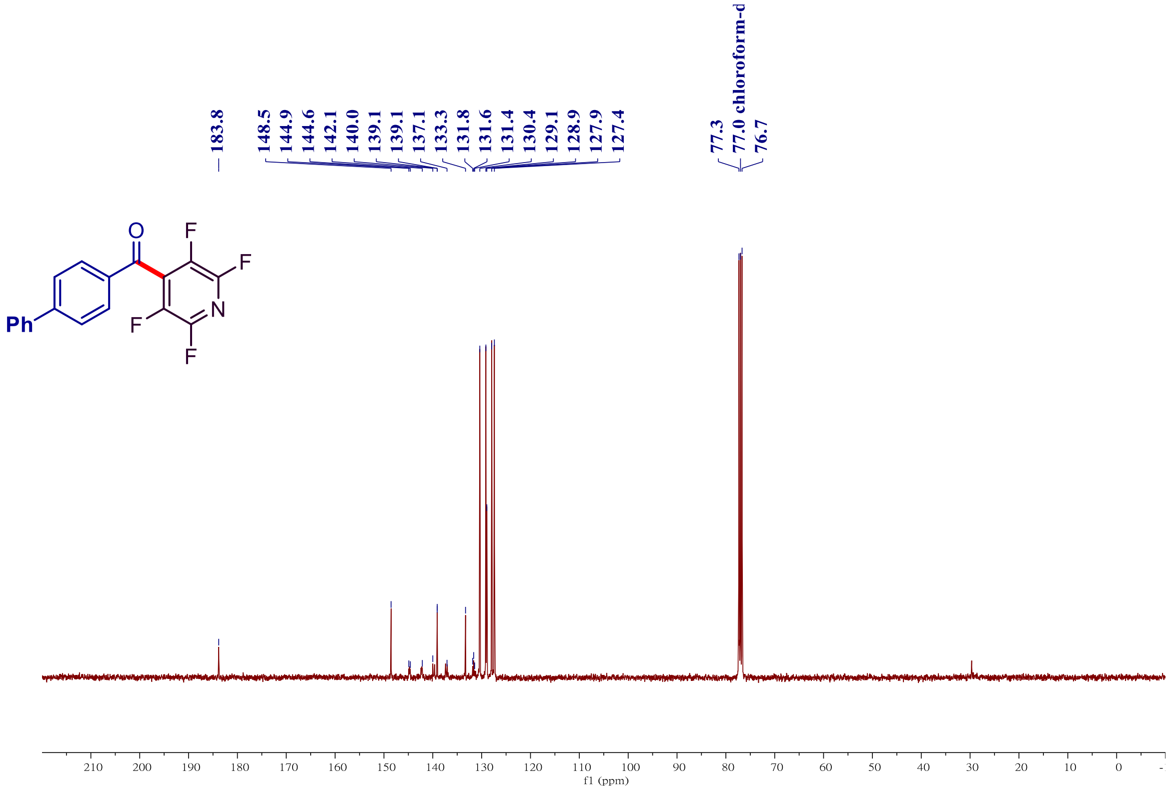

^{19}**F NMR** (282 MHz, $CDCl_3$) of **3s**

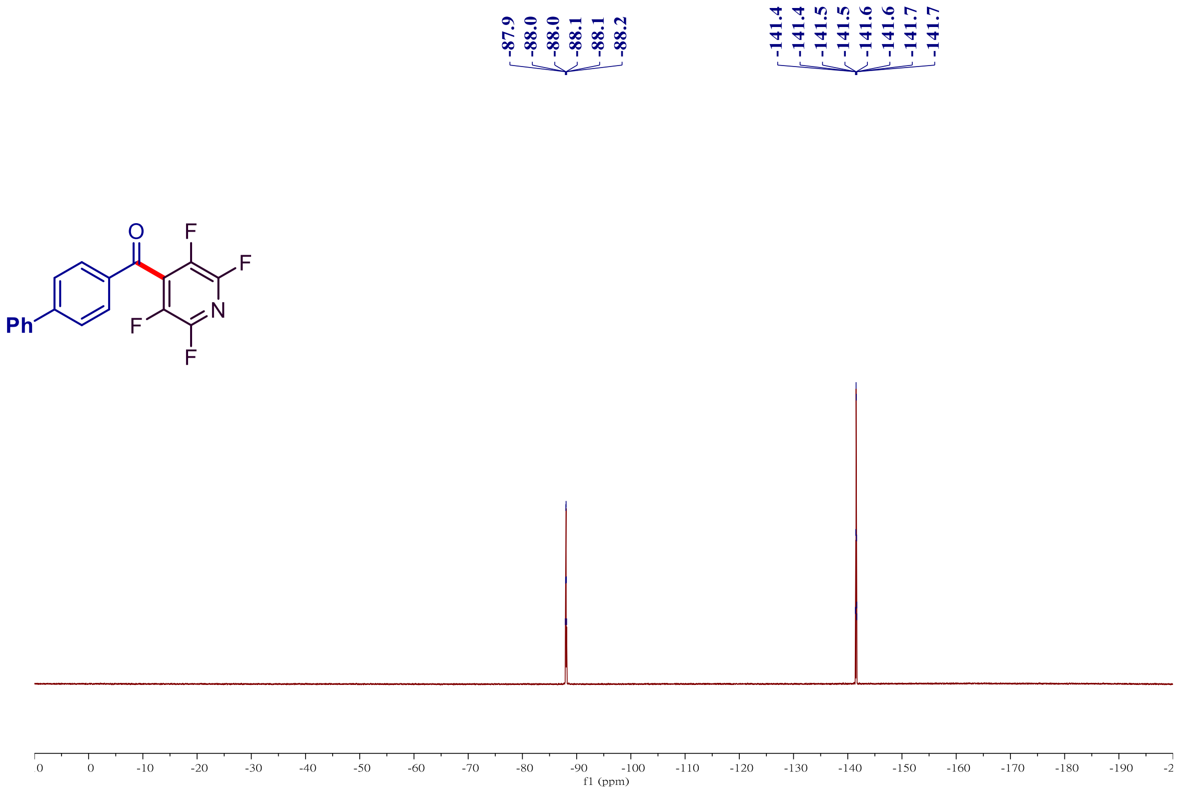

Back to the contents

¹H NMR (300 MHz, $CDCl_3$) of **3u**, See procedure

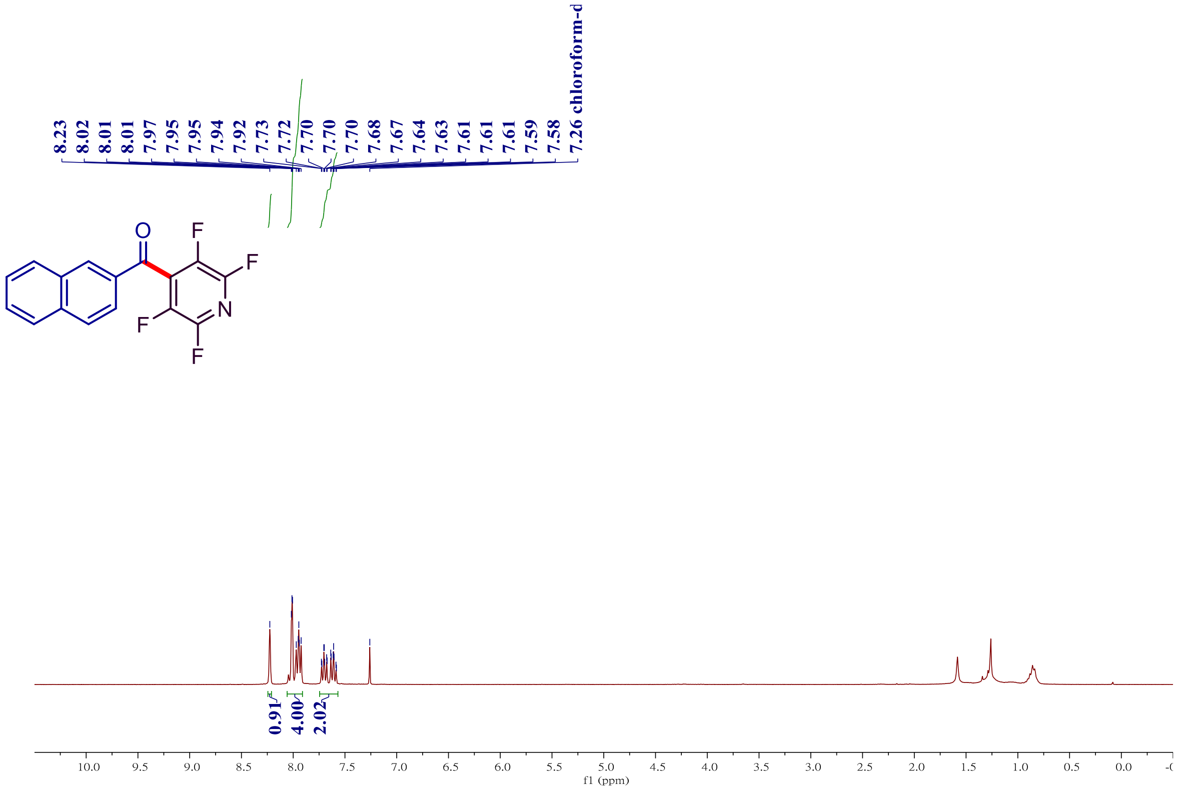

¹³C NMR (101 MHz, $CDCl_3$) of **3u**

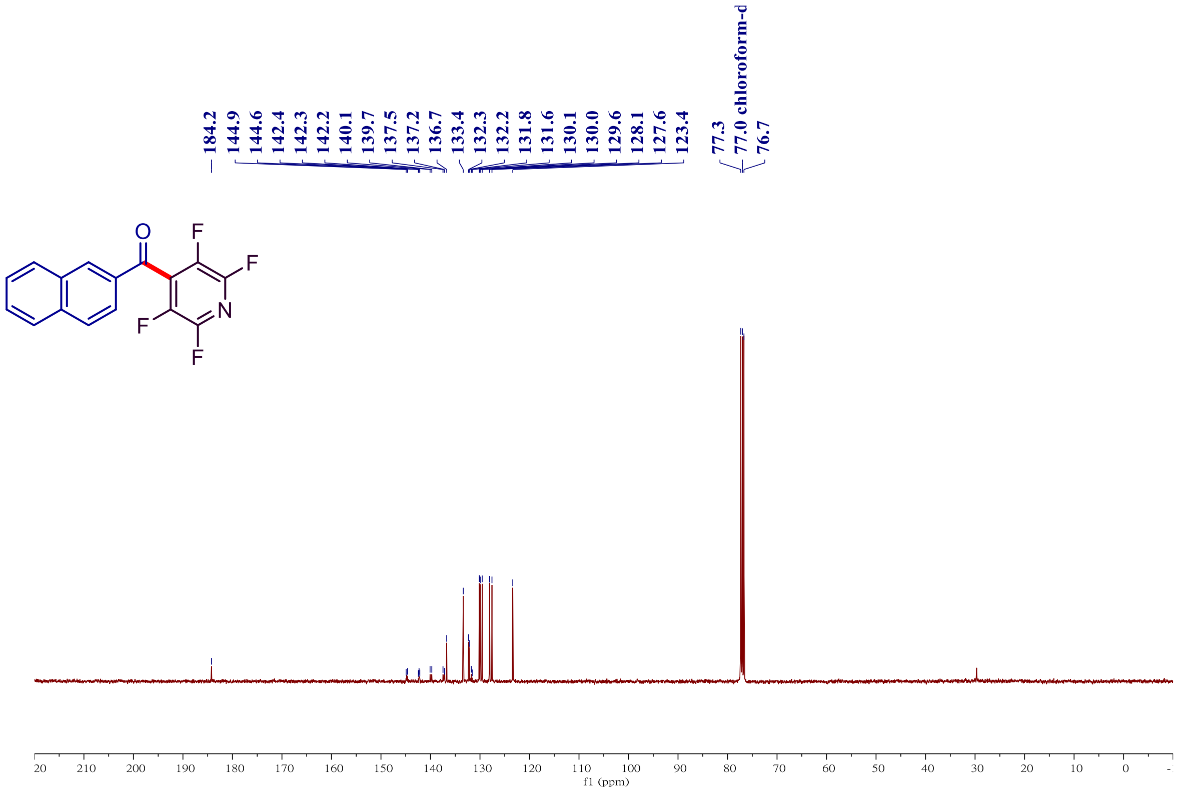

Back to the contents

^{19}F NMR (282 MHz, $CDCl_3$) of **3u**

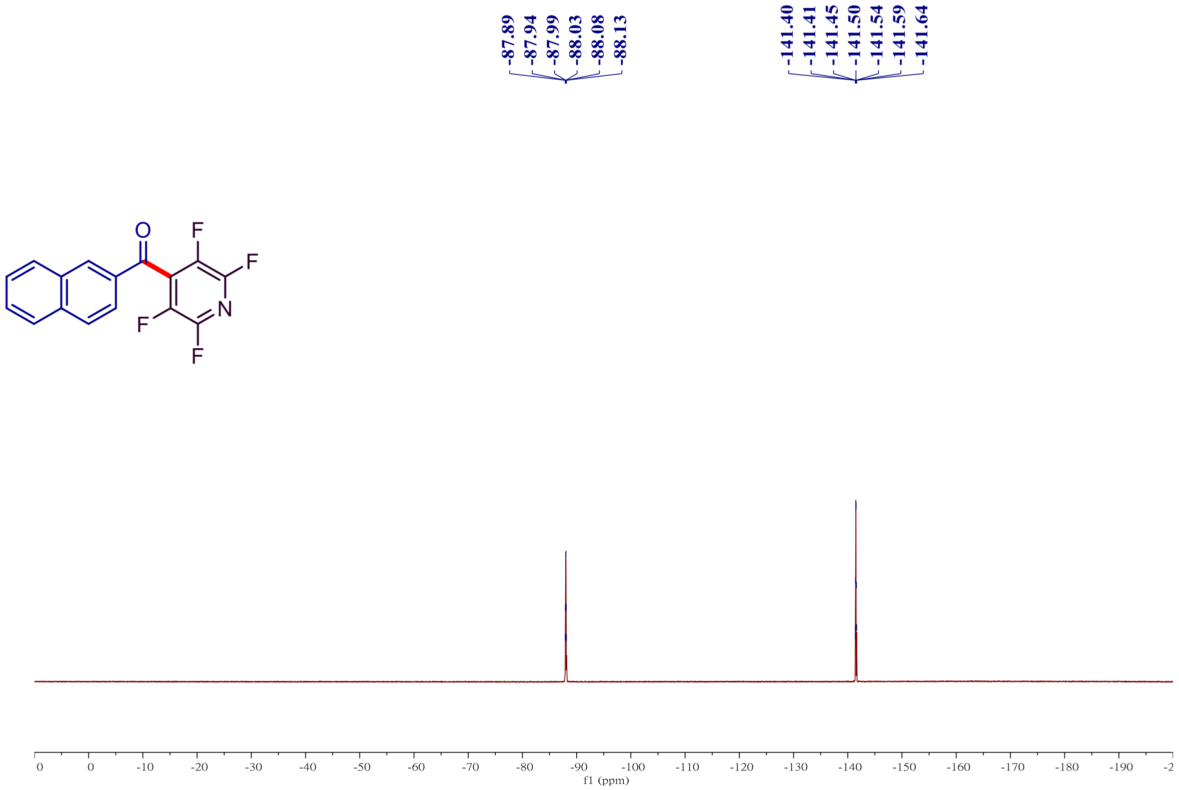

^{1}H NMR (300 MHz, $CDCl_3$) of **3v**, See procedure

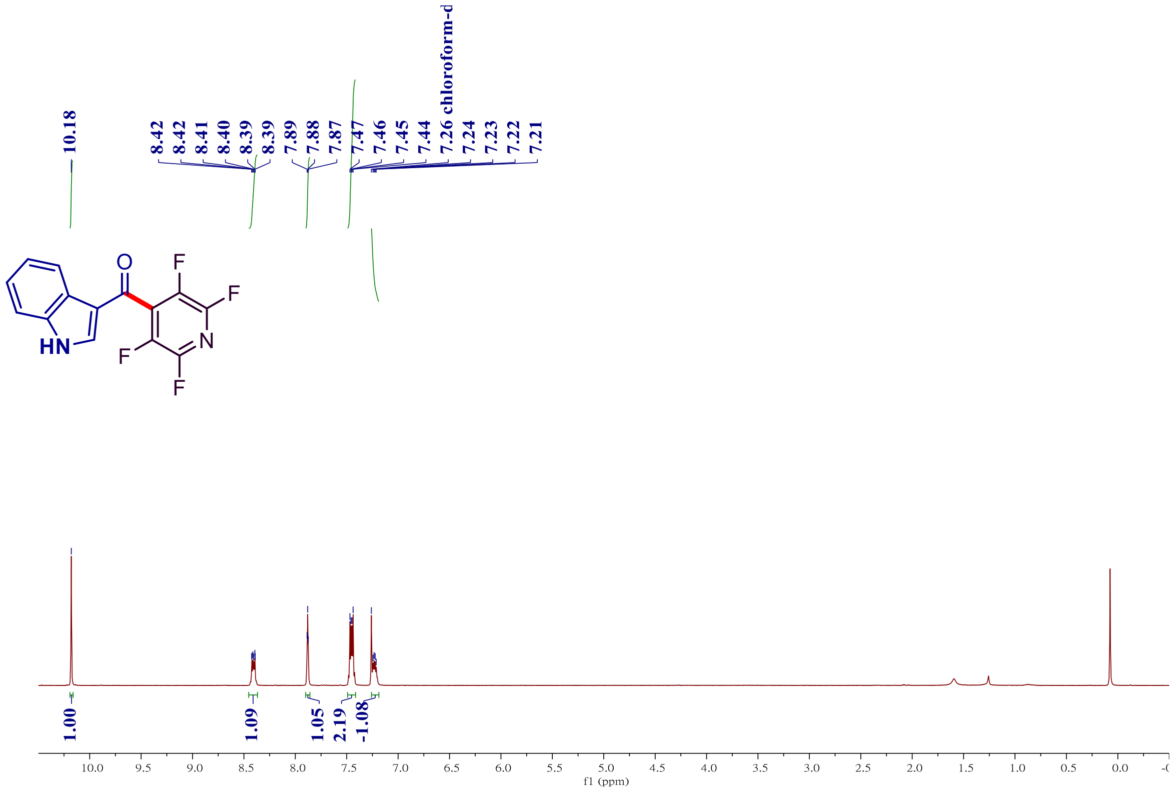

Back to the contents

^{13}C NMR (101 MHz, $CDCl_3$) of **3v**

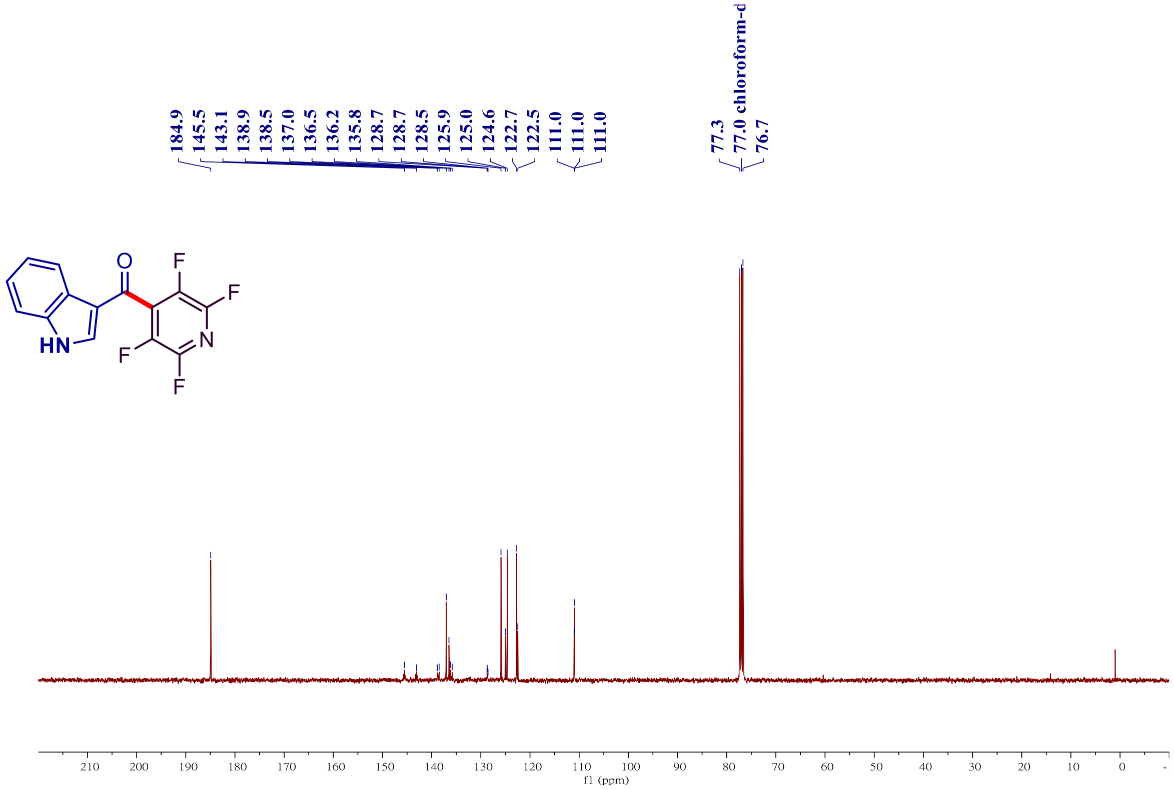

^{19}F NMR (282 MHz, $CDCl_3$) of **3v**

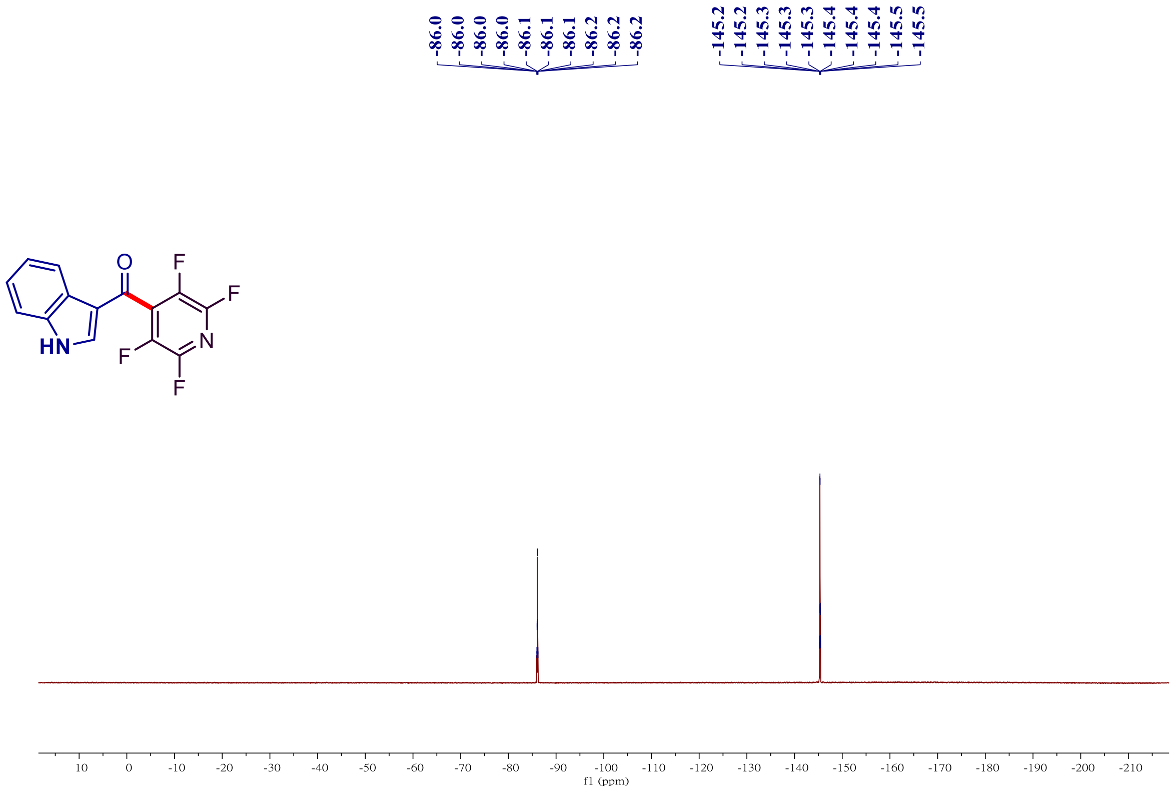

Back to the contents

¹H NMR (300 MHz, $CDCl_3$) of **3w**, See procedure

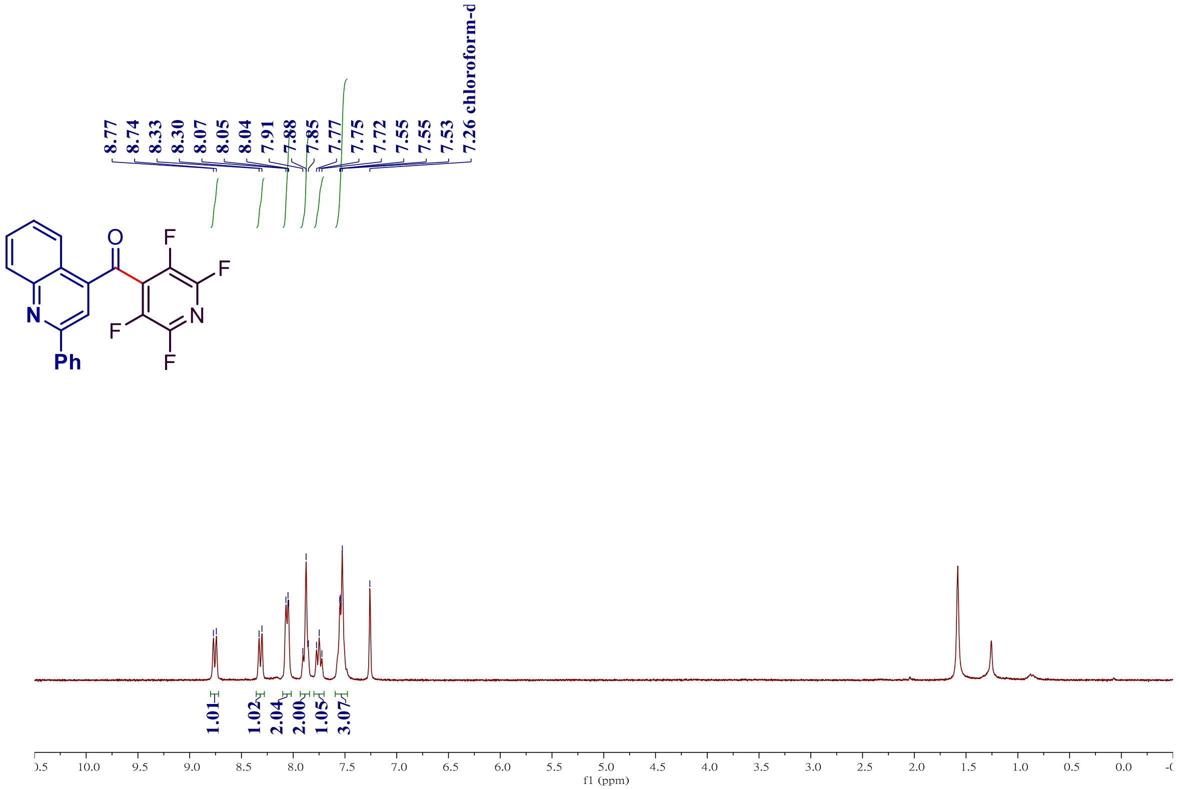

¹³C NMR (101 MHz, $CDCl_3$) of **3w**

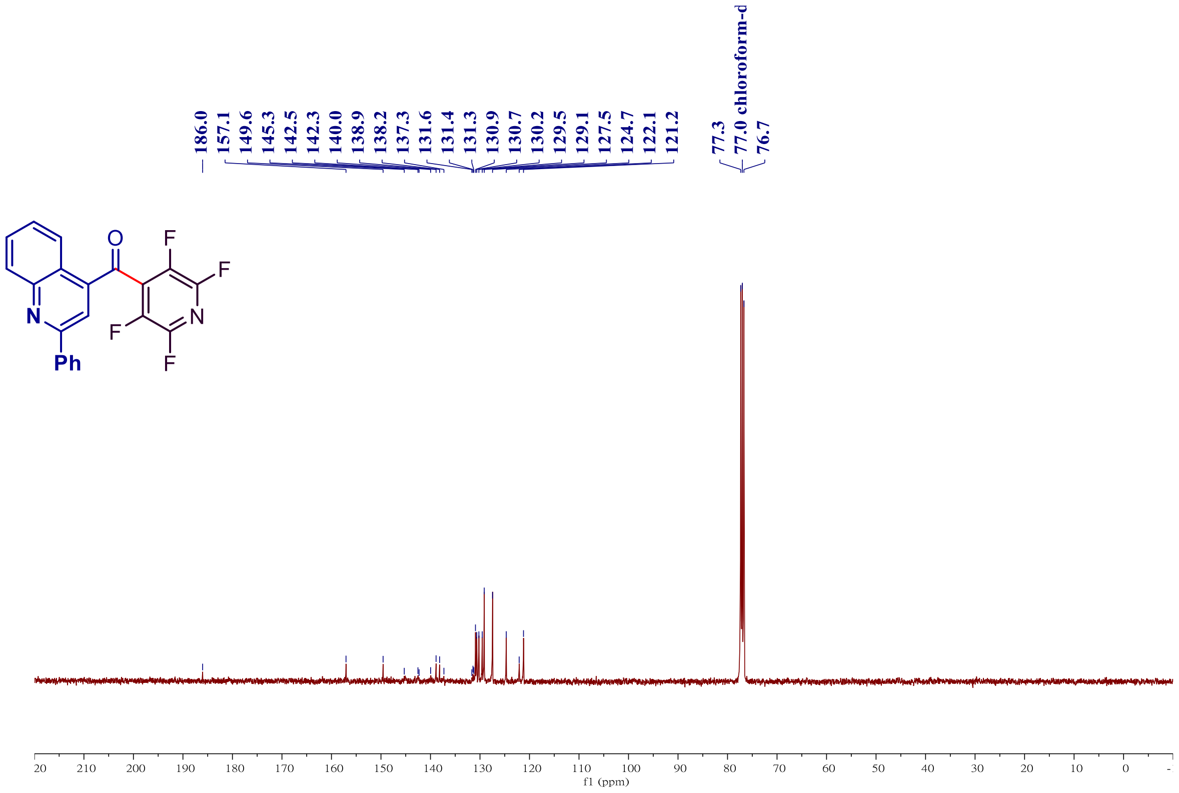

Back to the contents

^{19}F NMR (282 MHz, $CDCl_3$) of **3w**

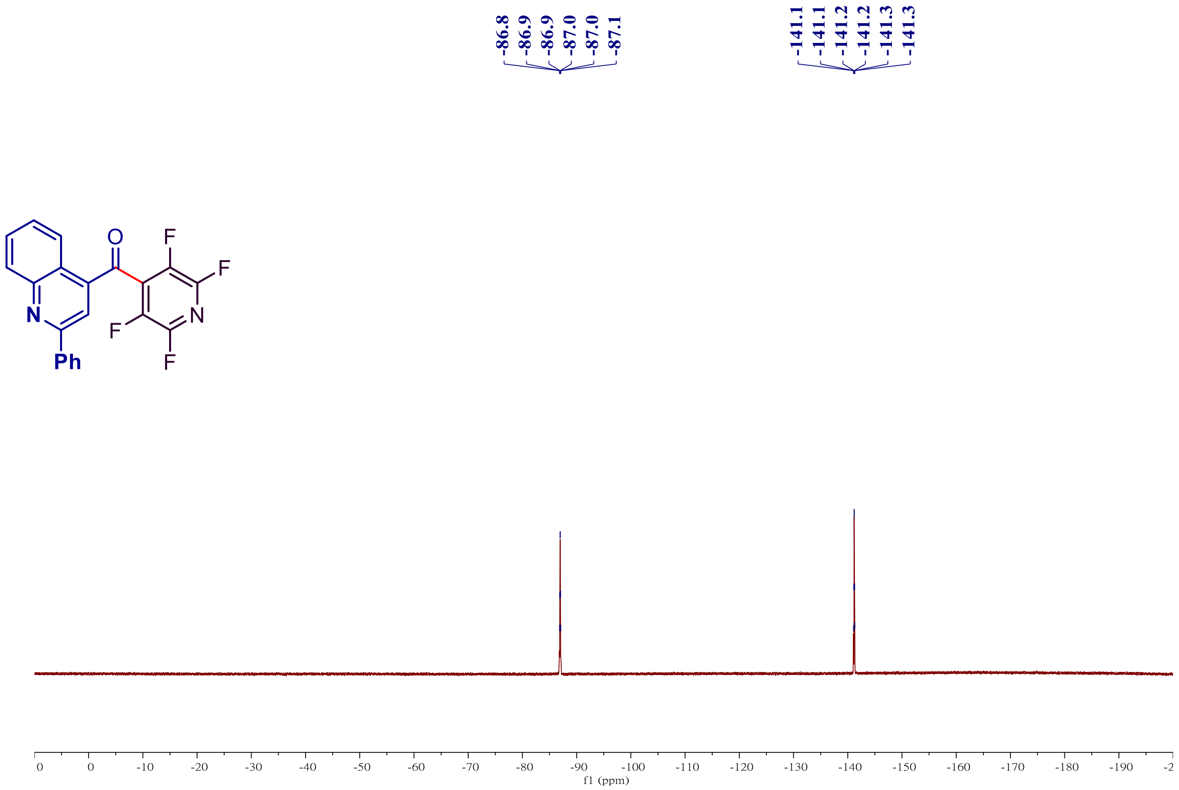

^{1}H NMR (300 MHz, $CDCl_3$) of **3x**, See procedure

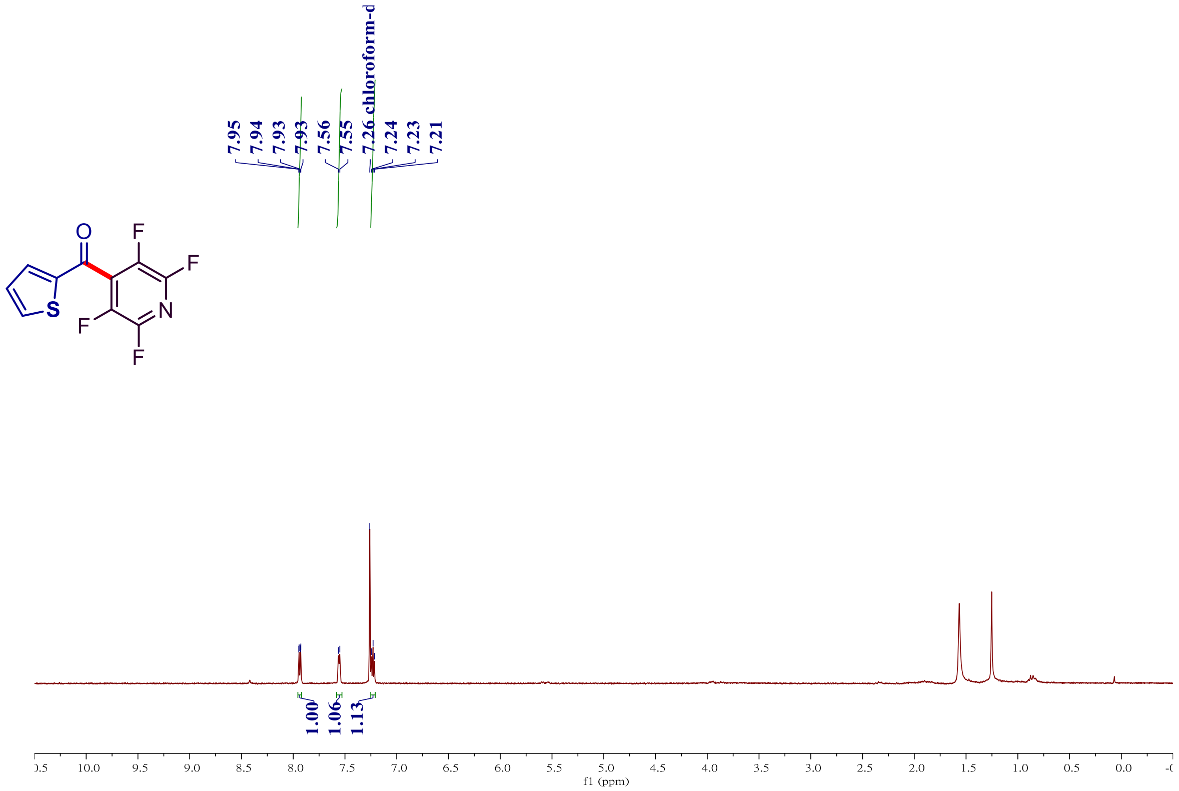

Back to the contents

^{13}C NMR (101 MHz, $CDCl_3$) of **3x**

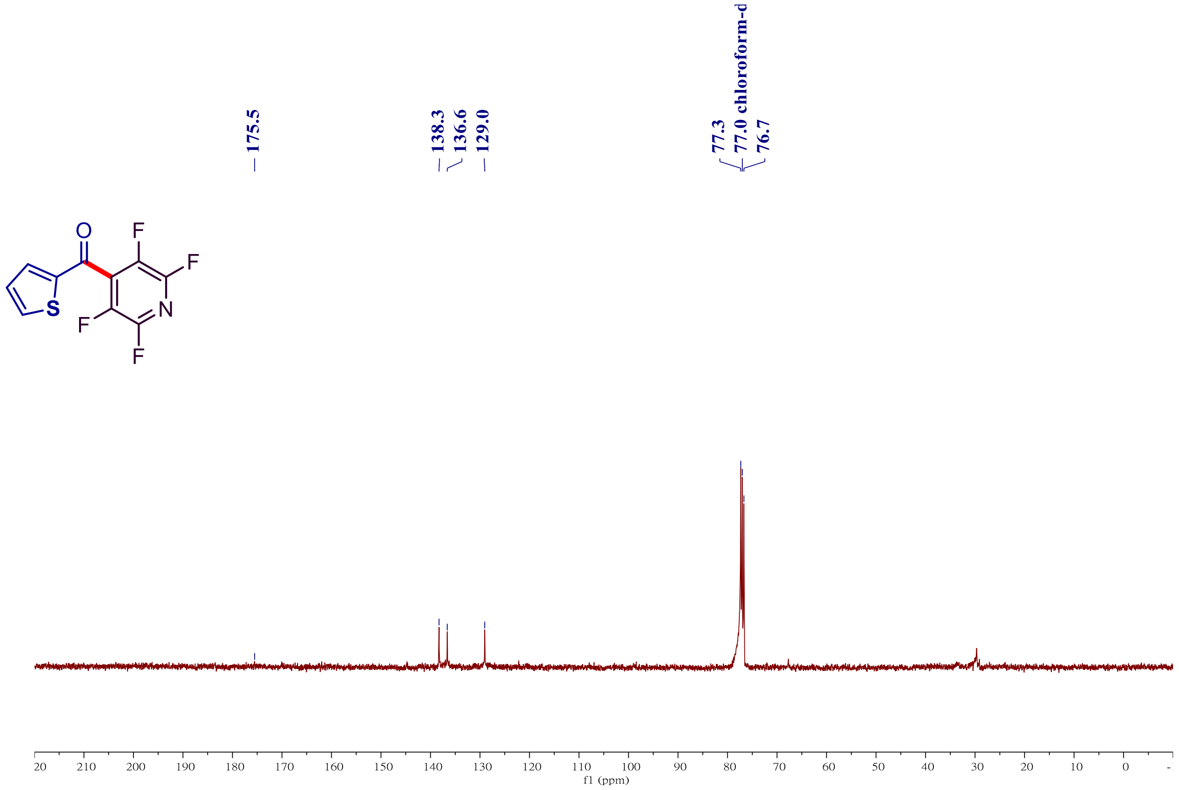

^{19}F NMR (282 MHz, $CDCl_3$) of **3x**

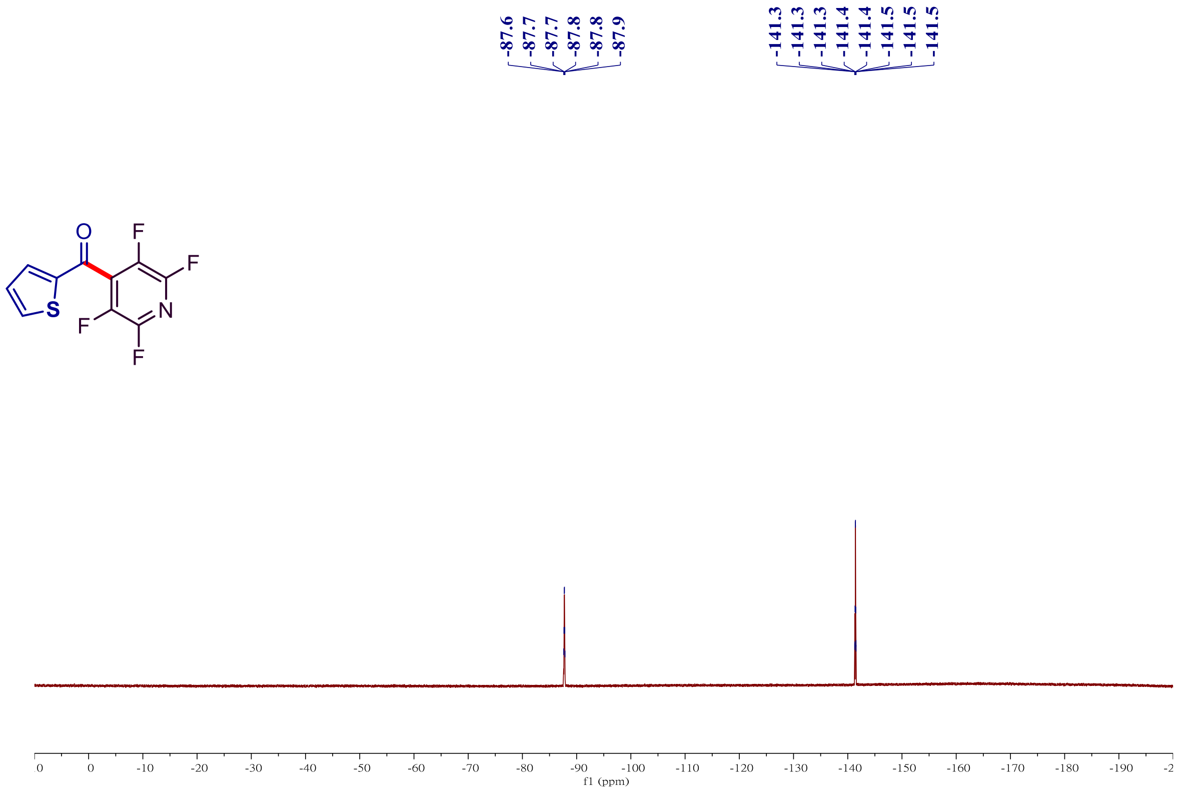

[Back to the contents](#)

^{1}H NMR (300 MHz, $CDCl_3$) of **3y**, See procedure

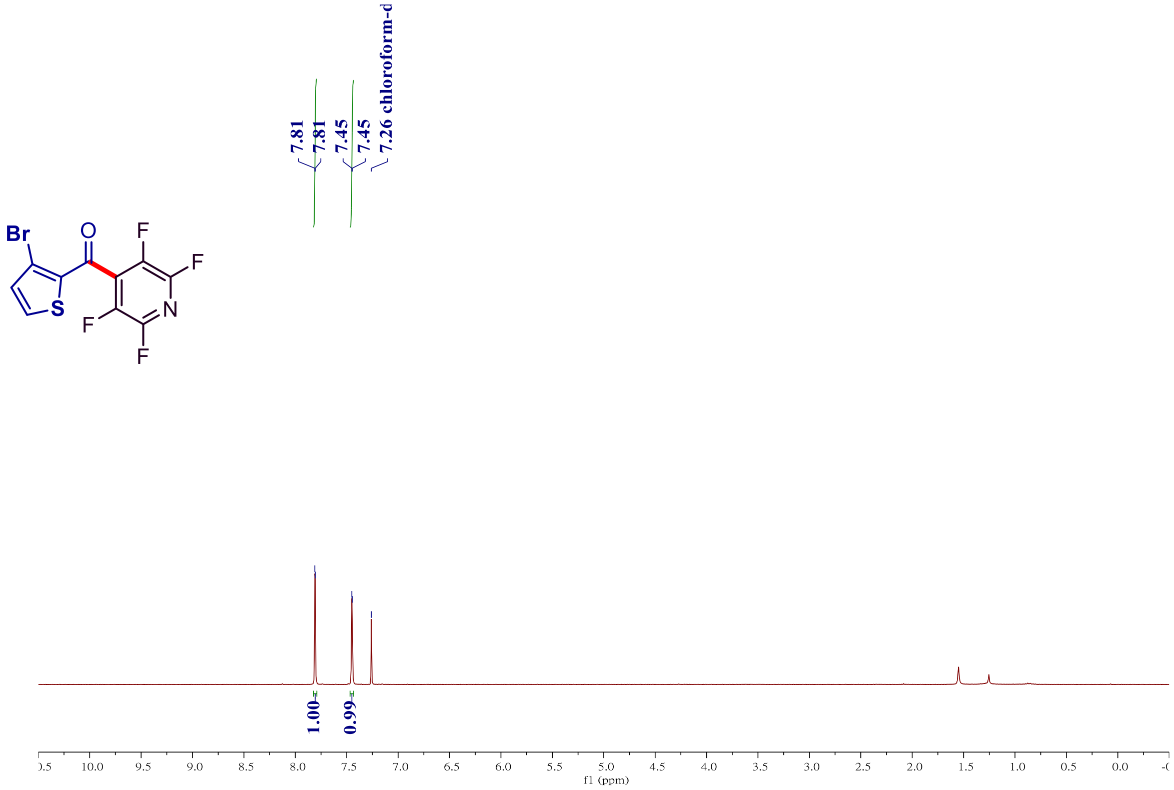

^{13}C NMR (101 MHz, $CDCl_3$) of **3y**

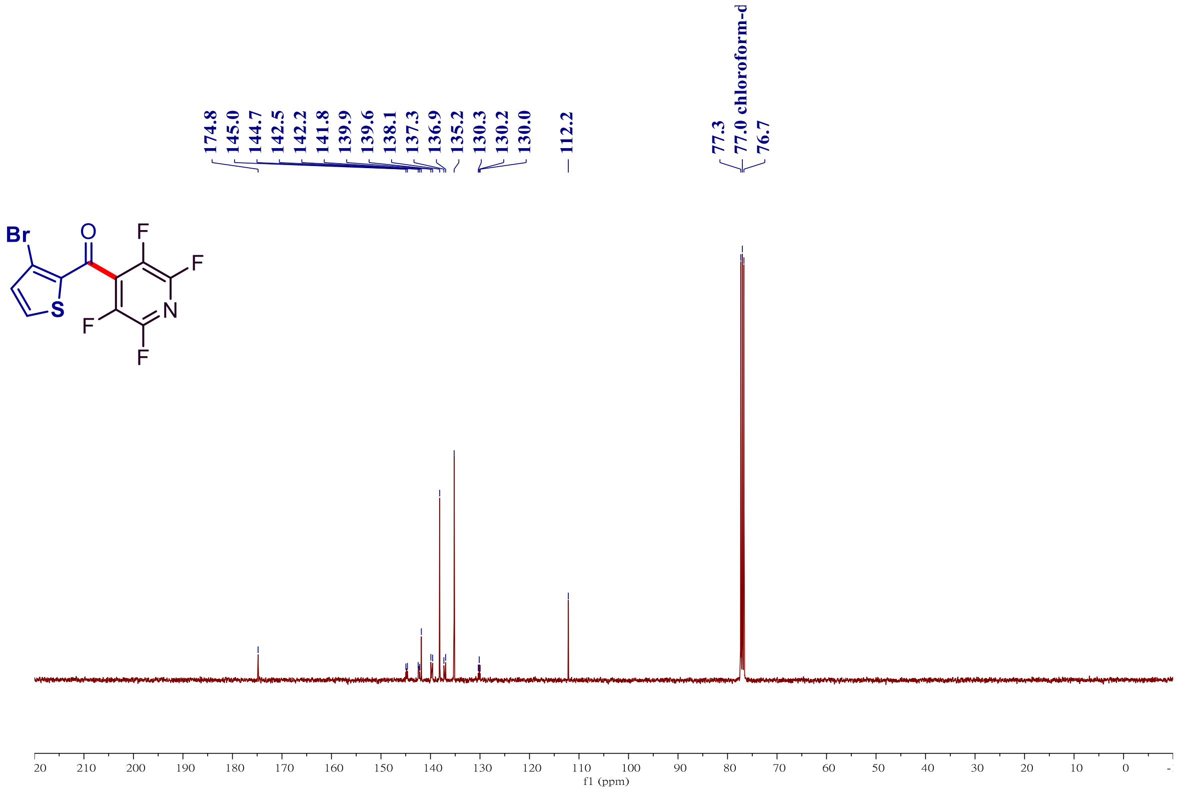

Back to the contents

^{19}F NMR (282 MHz, $CDCl_3$) of **3y**

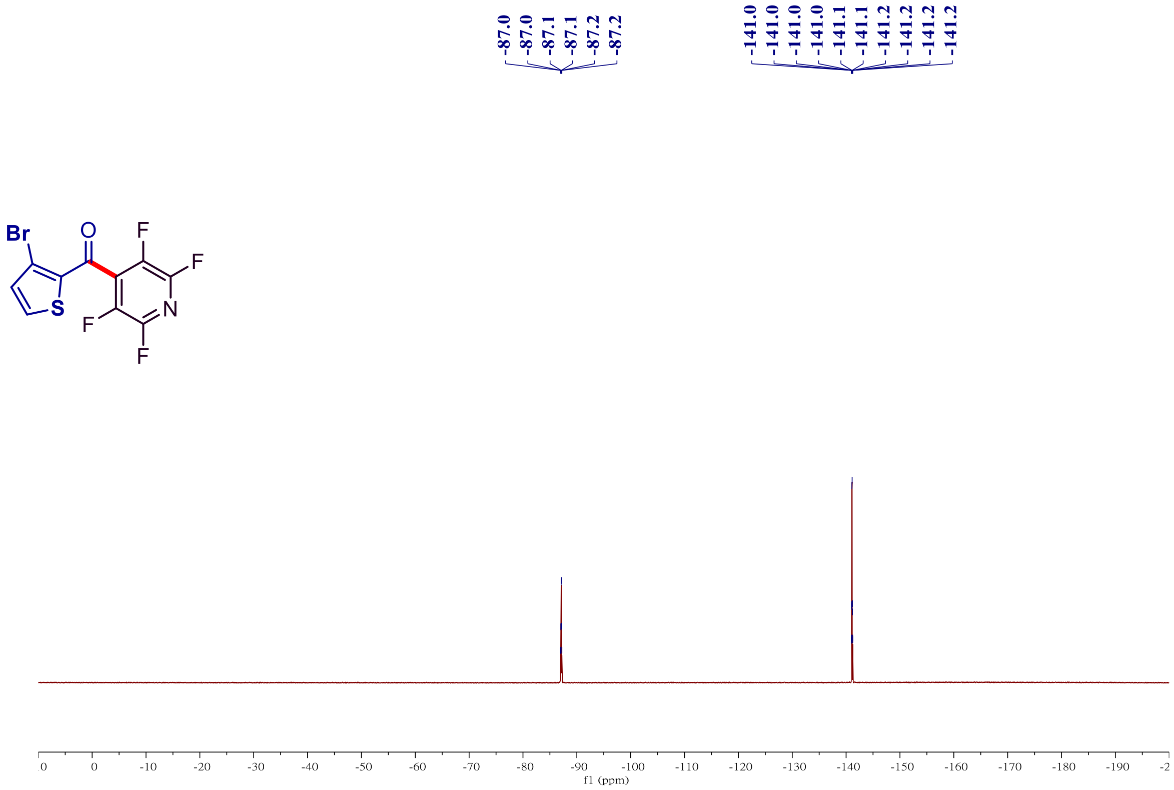

^{1}H NMR (400 MHz, $CDCl_3$) of **4a**, See procedure

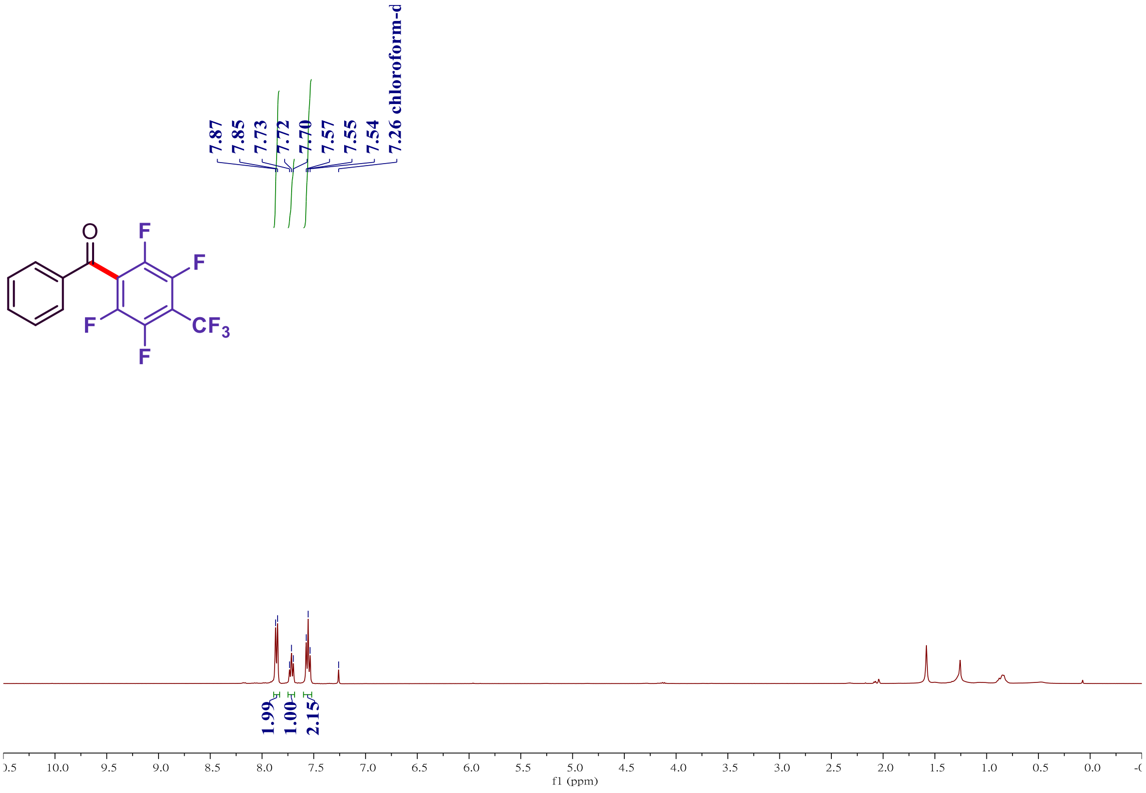

Back to the contents

^{13}C NMR (101 MHz, $CDCl_3$) of **4a**

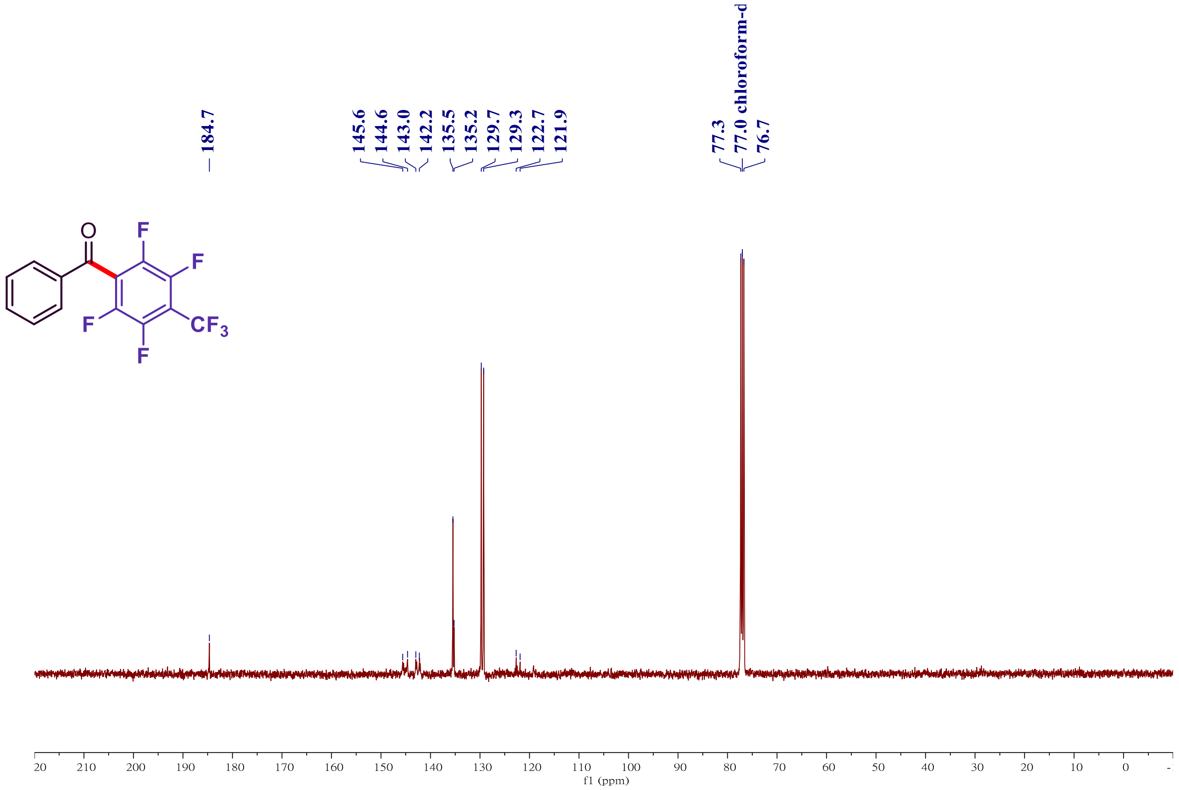

^{19}F NMR (376 MHz, $CDCl_3$) of **4a**

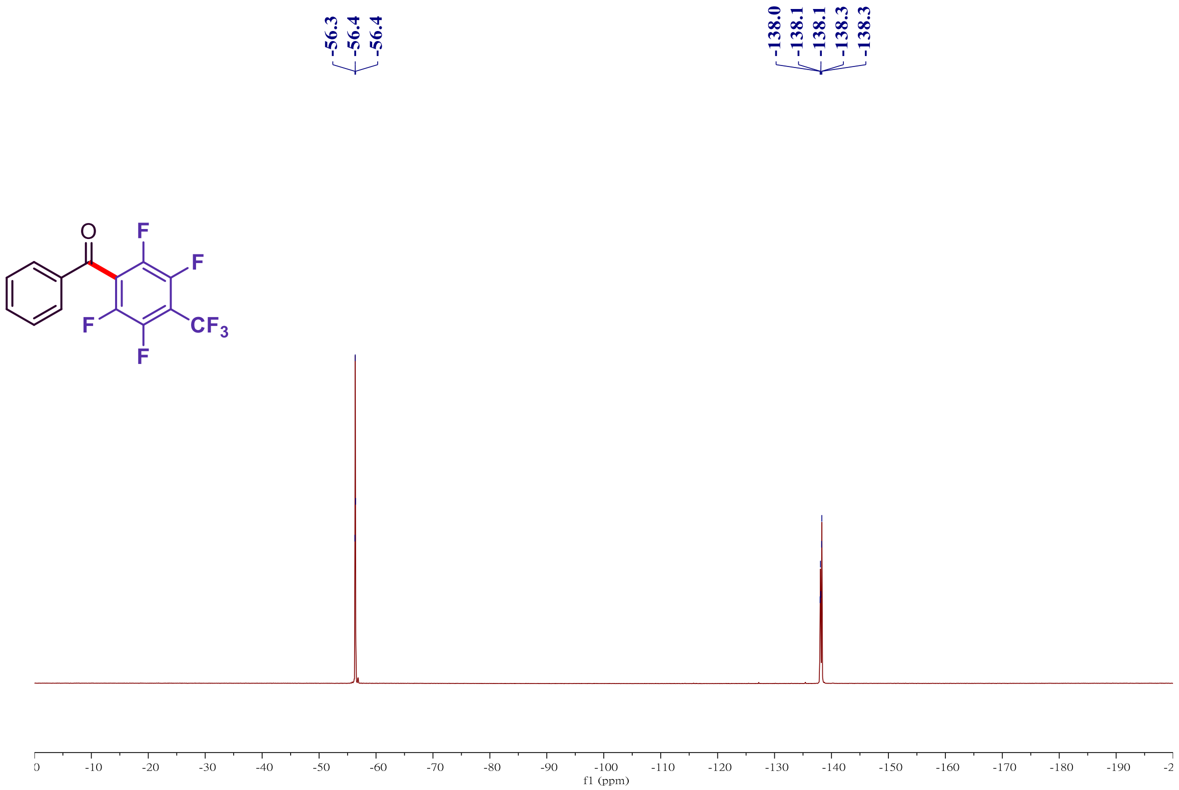

Back to the contents

^{1}H NMR (300 MHz, $CDCl_3$) of **4b**, See procedure

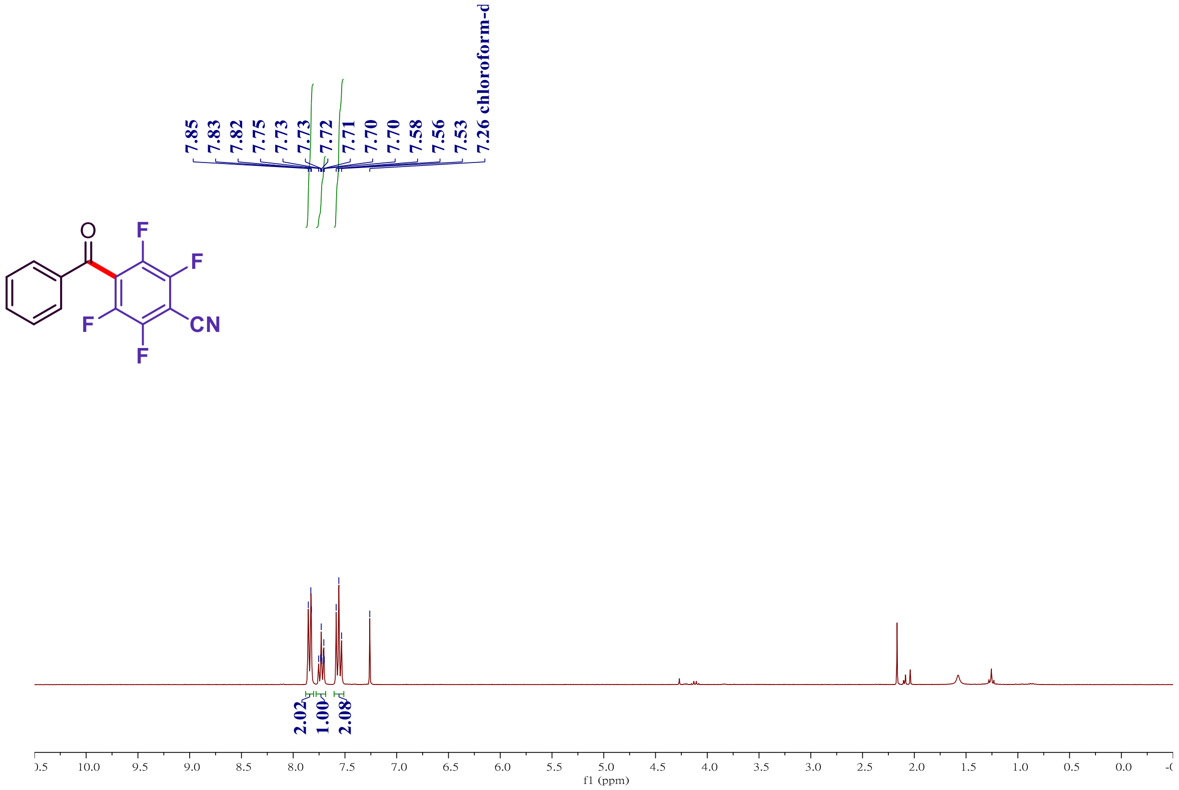

^{13}C NMR (101 MHz, $CDCl_3$) of **4b**

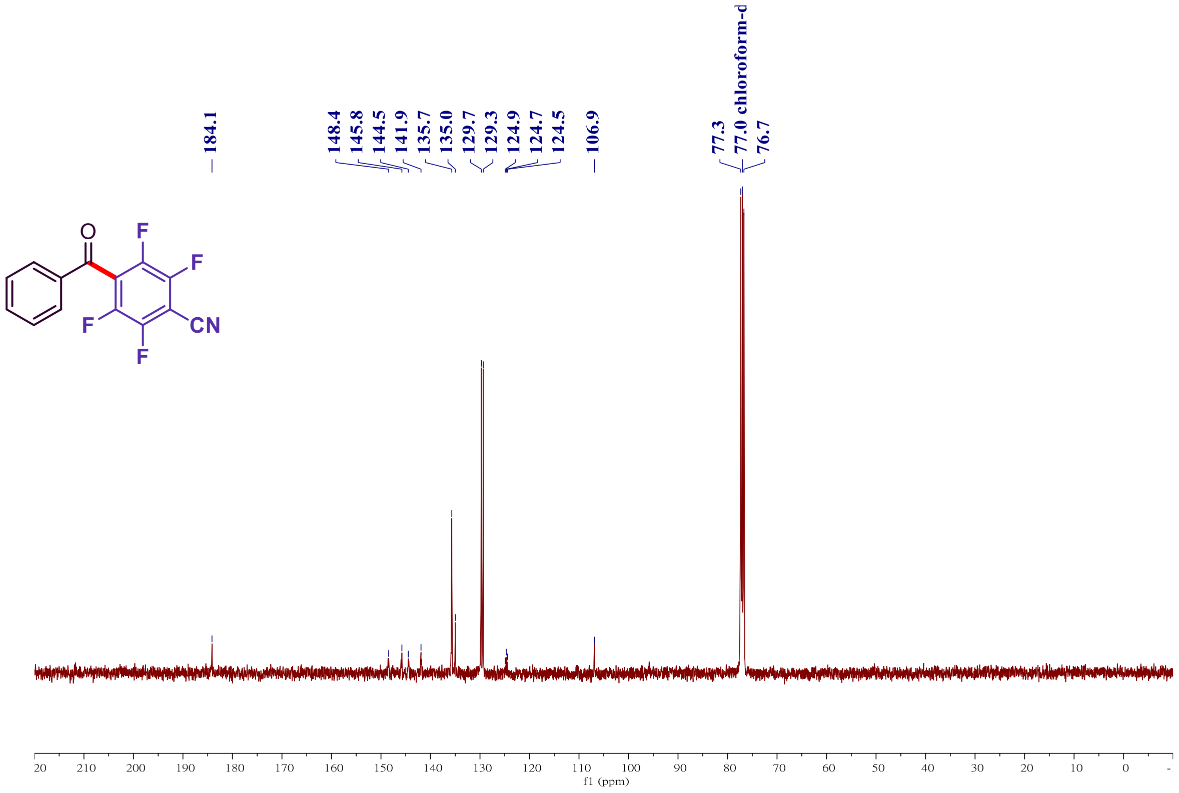

[Back to the contents](#)

^{19}F NMR (376 MHz, $CDCl_3$) of **4b**

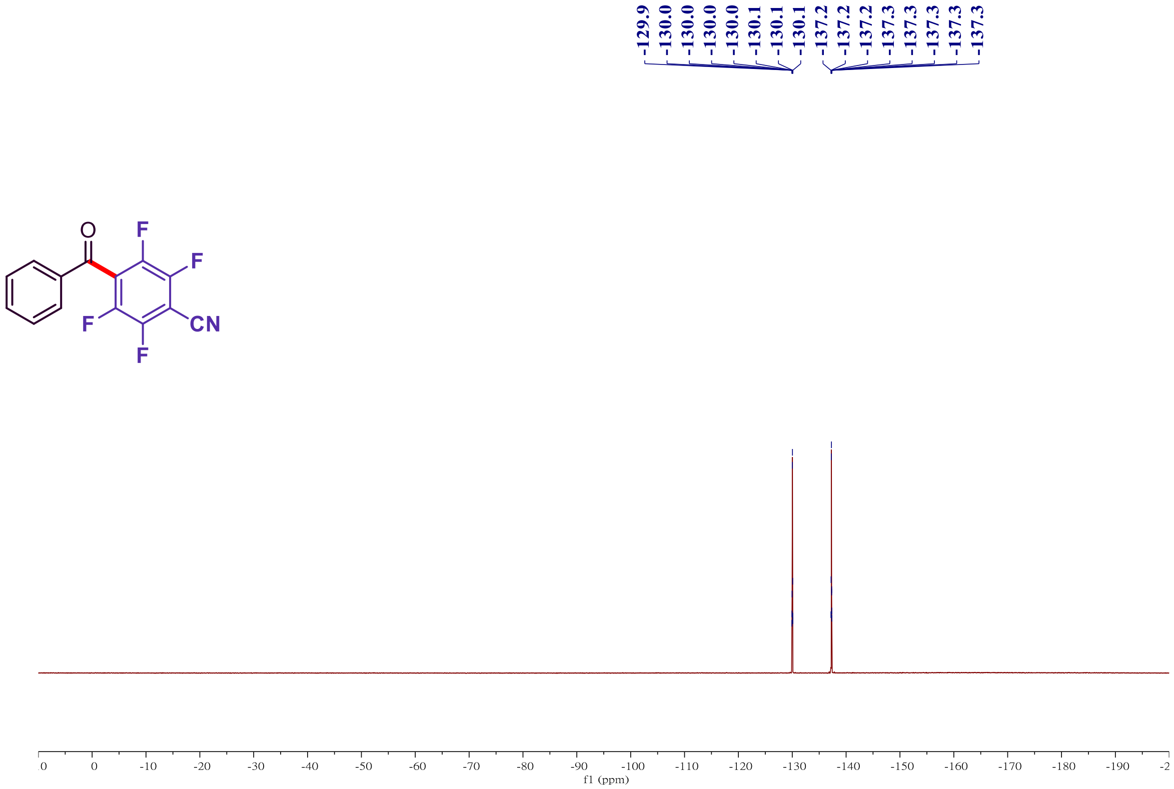

^{1}H NMR (400 MHz, $CDCl_3$) of **4c**, [See procedure](#)

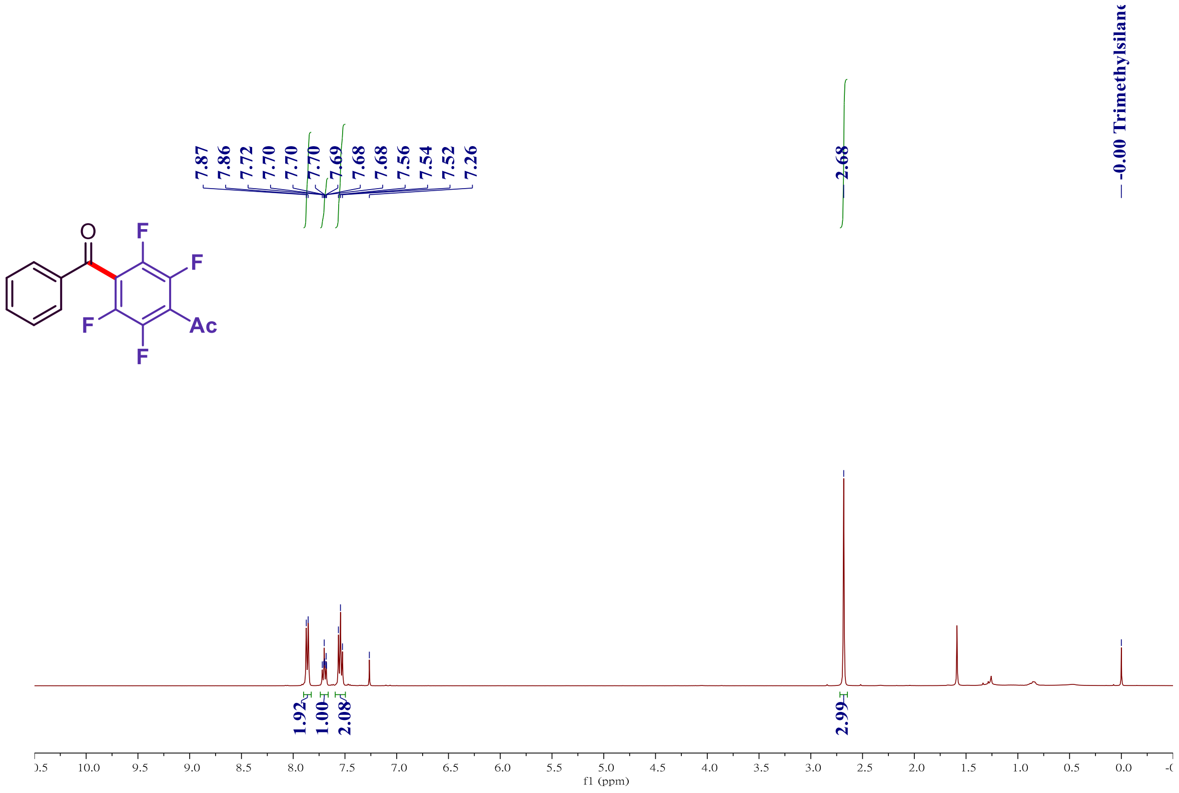

Back to the contents

^{13}**C NMR** (101 MHz, $CDCl_3$) of **4c**

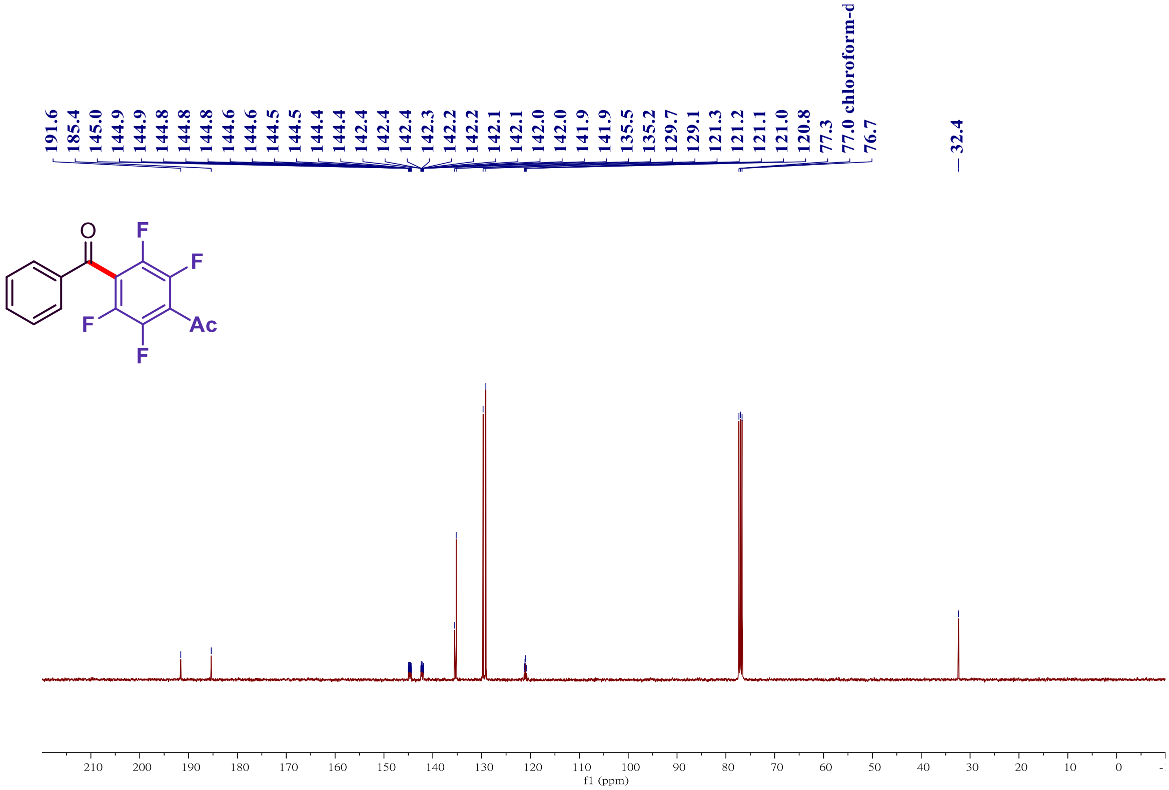

^{19}**F NMR** (376 MHz, $CDCl_3$) of **4c**

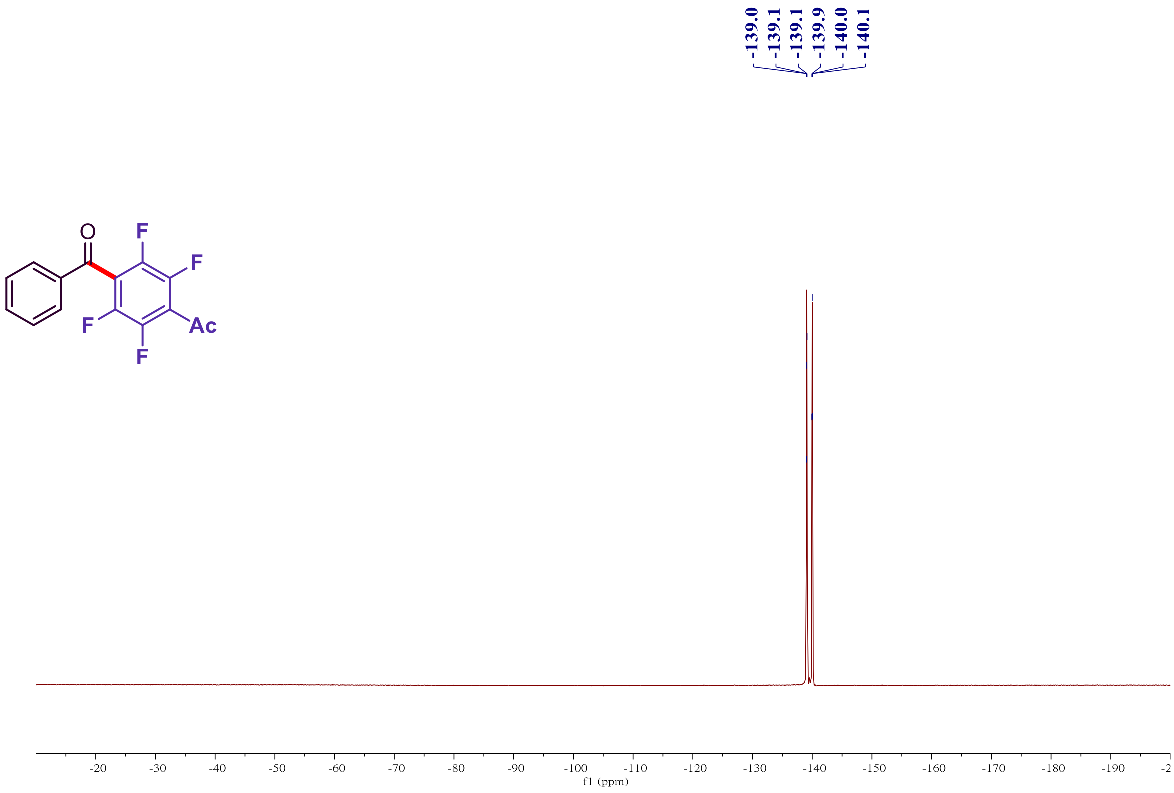

Back to the contents

^{1}H NMR (300 MHz, $CDCl_3$) of **4d**, See procedure

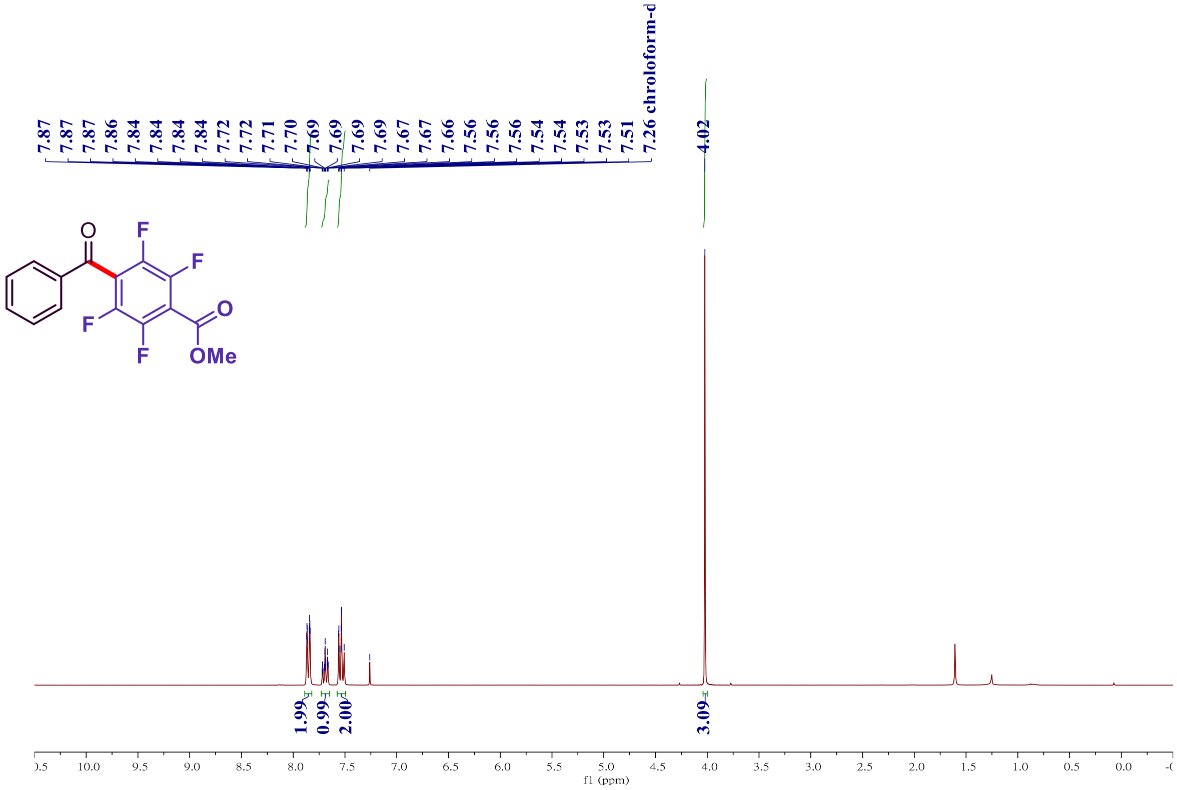

^{13}C NMR (101 MHz, $CDCl_3$) of **4d**

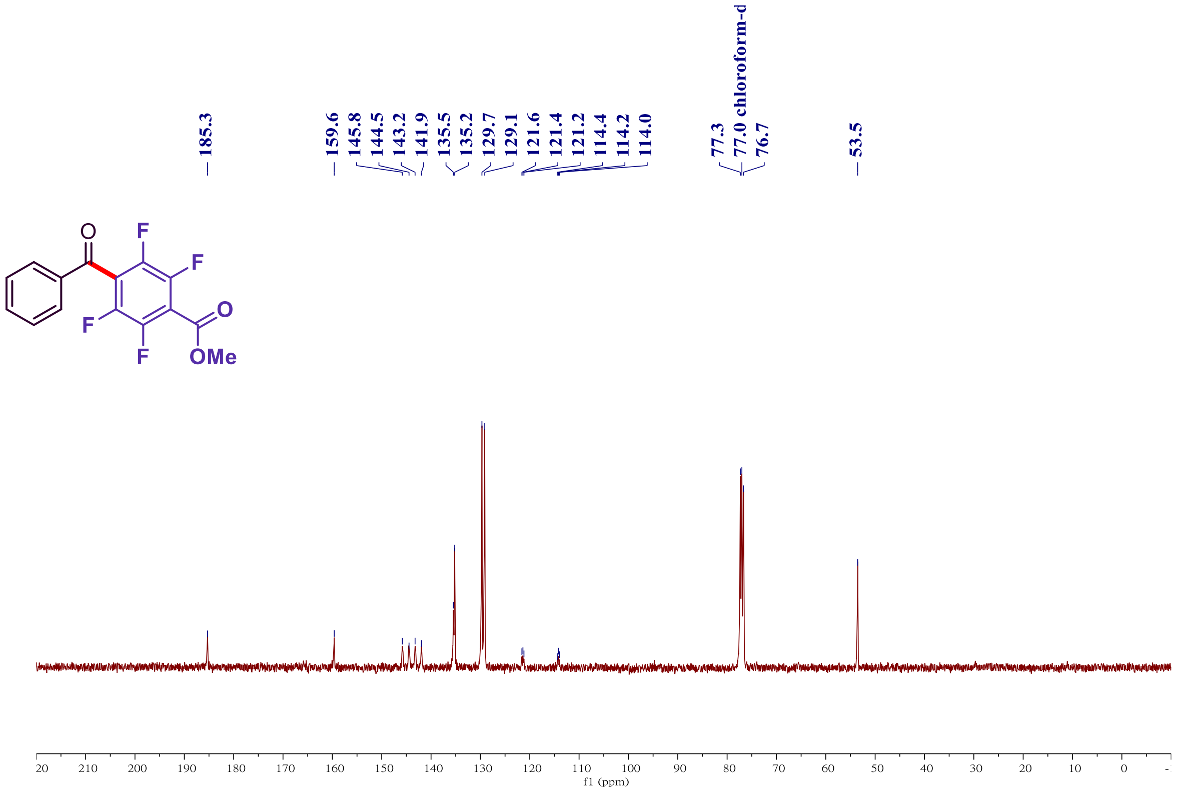

[Back to the contents](#)

^{19}F NMR (282 MHz, $CDCl_3$) of **4d**

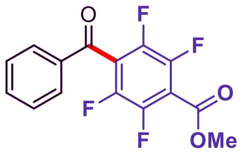

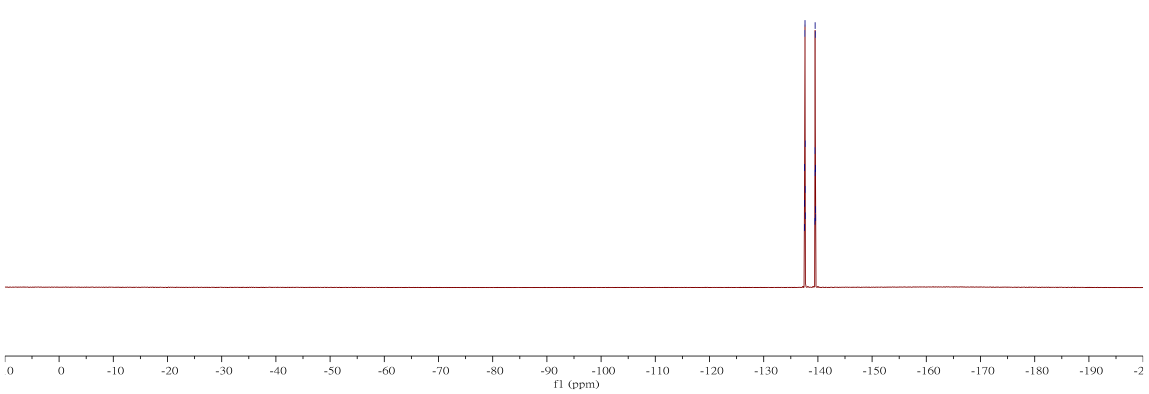

^{1}H NMR (300 MHz, $CDCl_3$) of **4e**, See procedure

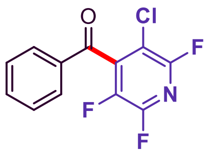

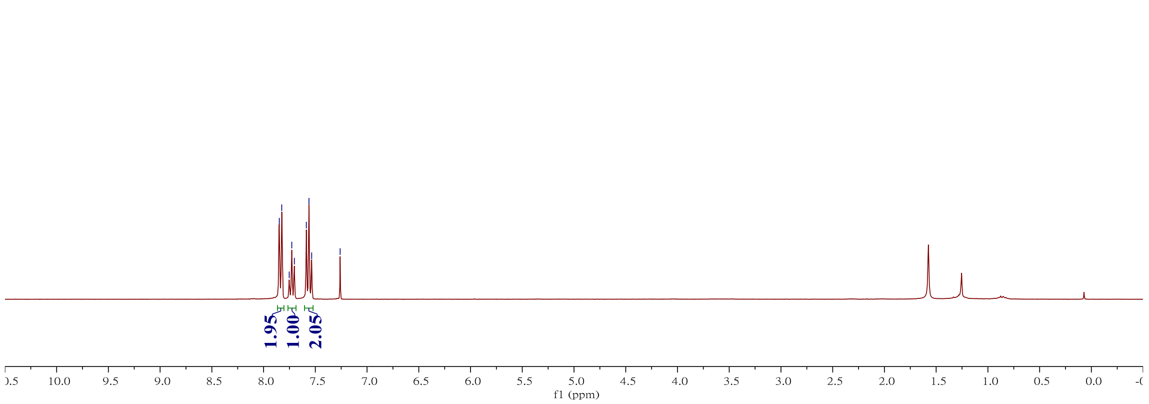

Back to the contents

^{13}C NMR (101 MHz, $CDCl_3$) of **4e**

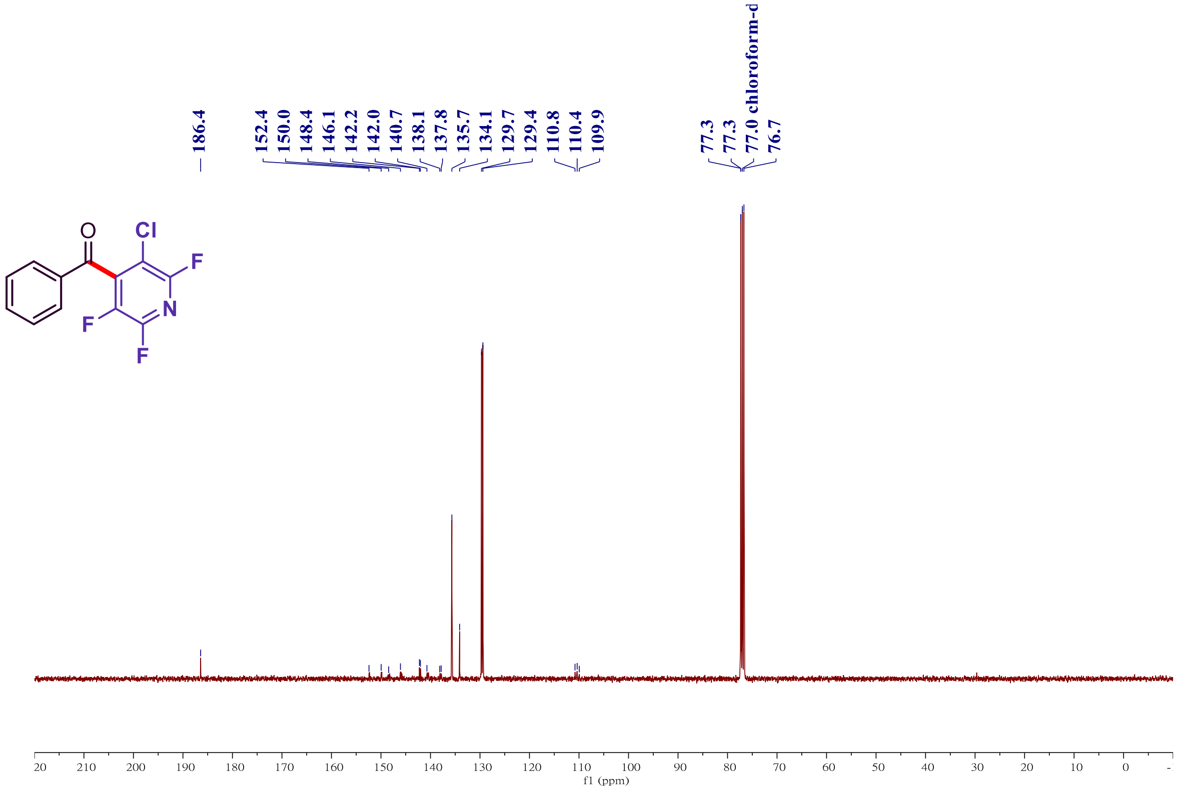

^{19}F NMR (282 MHz, $CDCl_3$) of **4e**

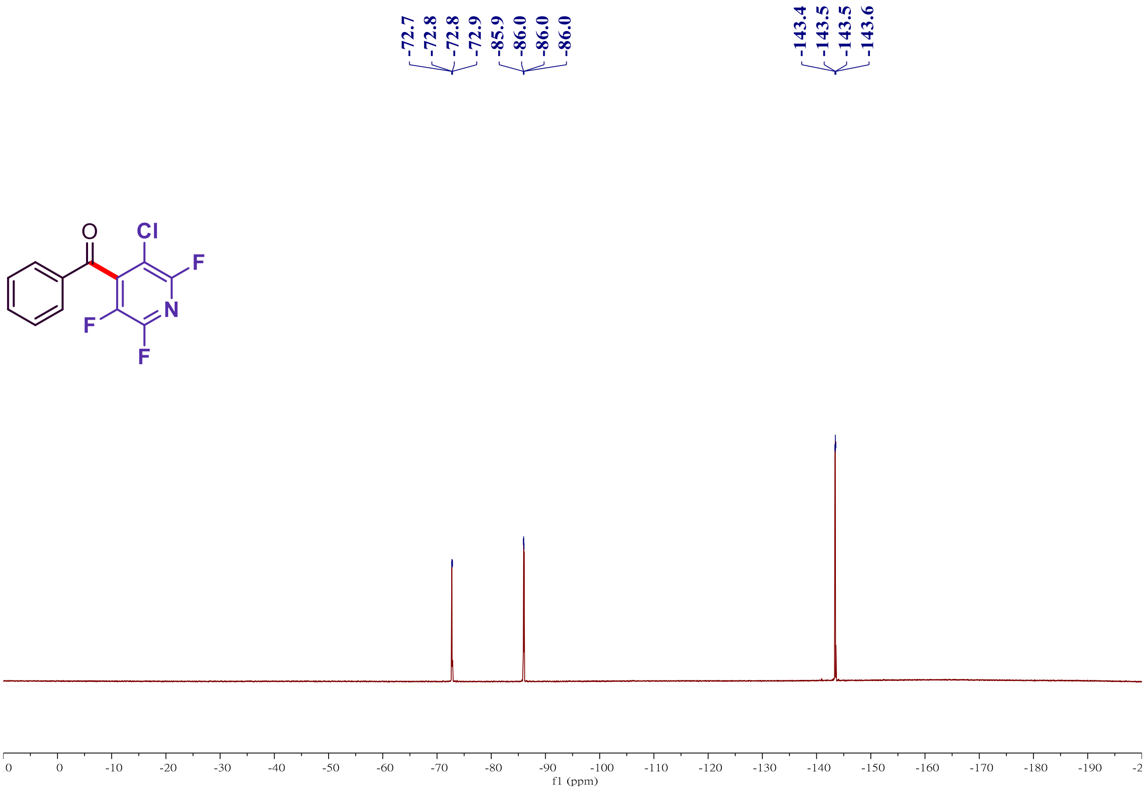

[Back to the contents]

^{1}H NMR (300 MHz, $CDCl_3$) of **4f**, See procedure

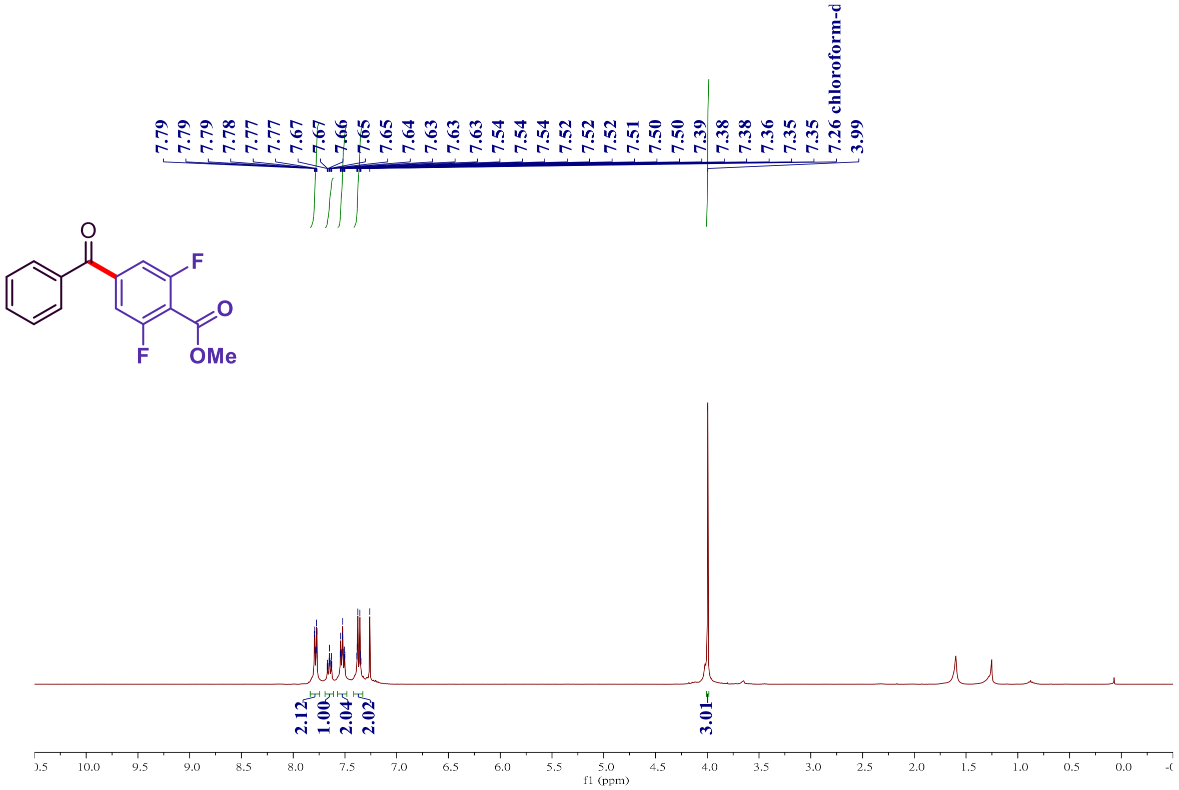

^{13}C NMR (101 MHz, $CDCl_3$) of **4f**

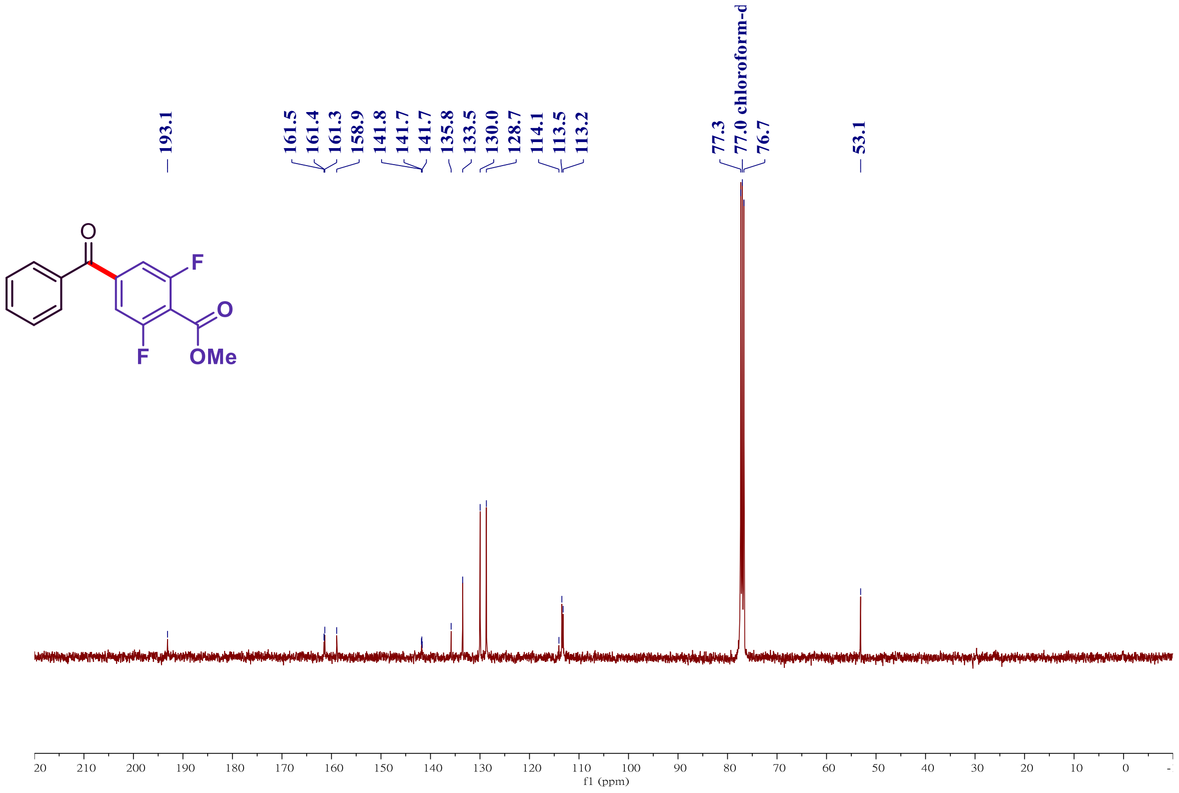

Back to the contents

^{19}F NMR (282 MHz, $CDCl_3$) of **4f**

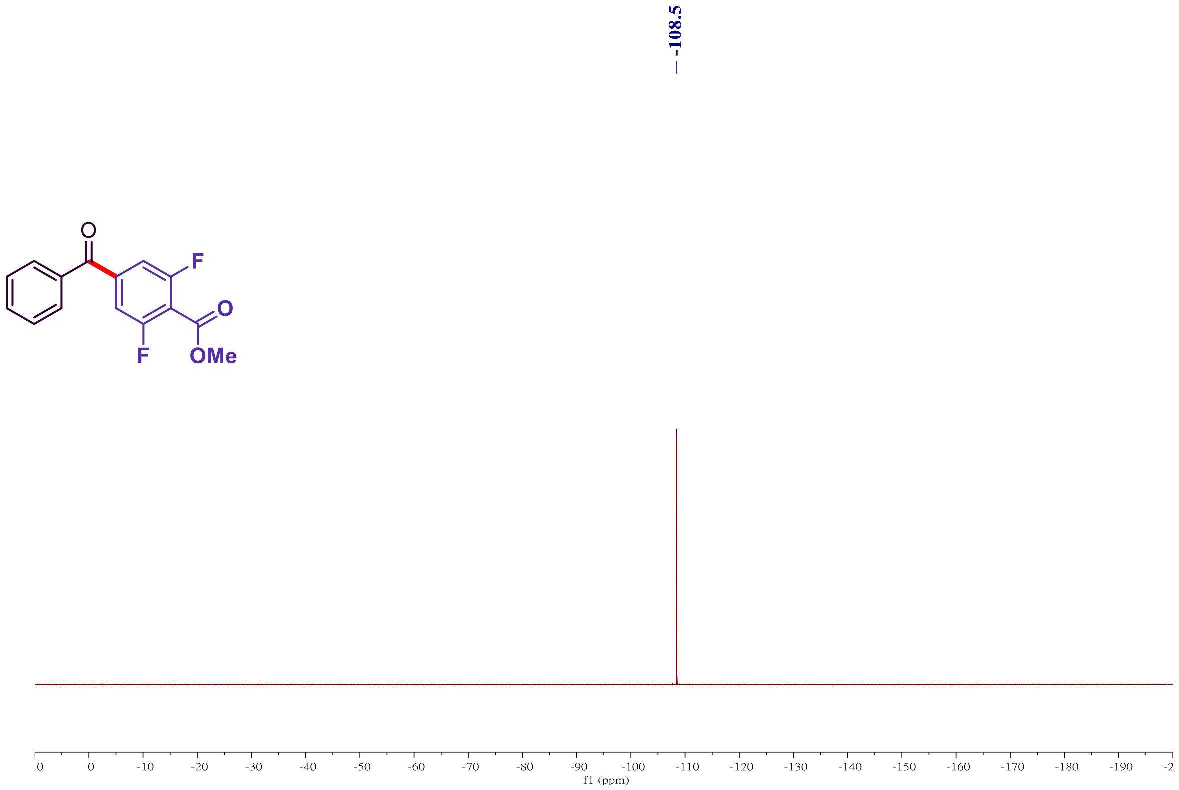

Substrate scopes of biorelevant molecules

^{1}H NMR (300 MHz, $CDCl_3$) of **5a**, See procedure

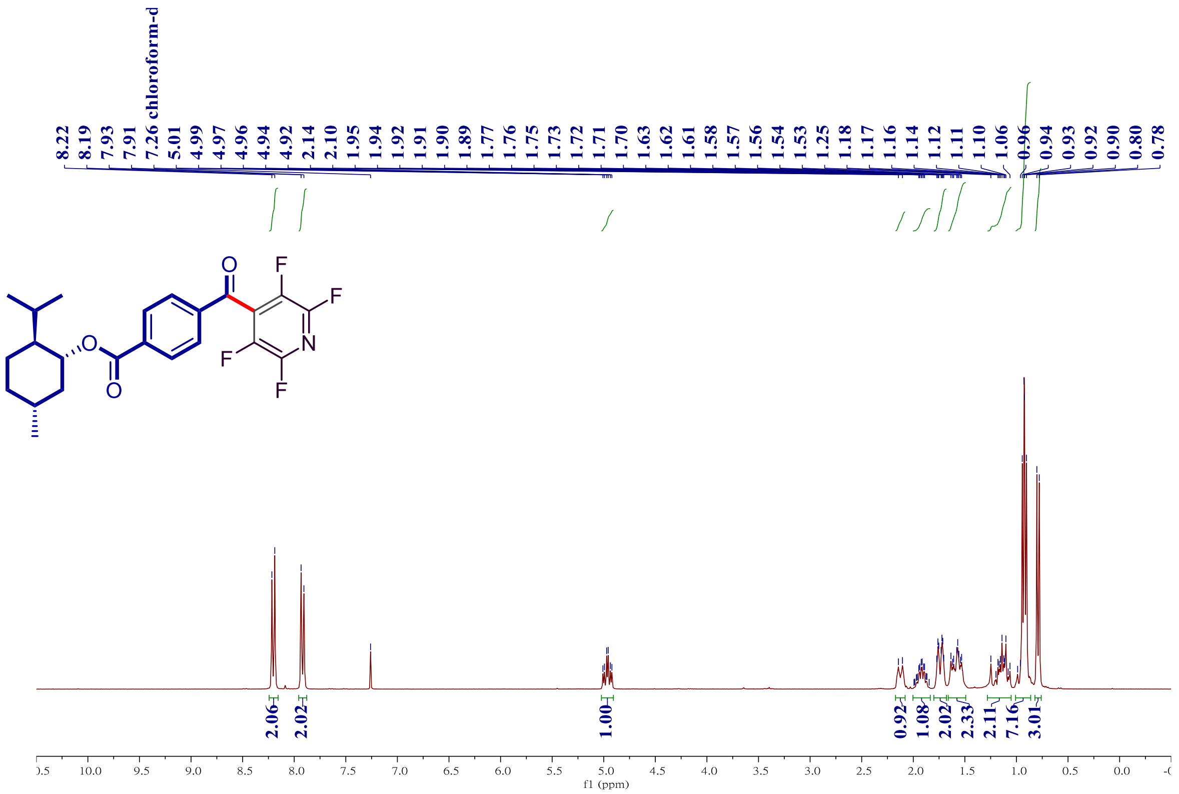

Back to the contents

^{13}C NMR (101 MHz, $CDCl_3$) of **5a**

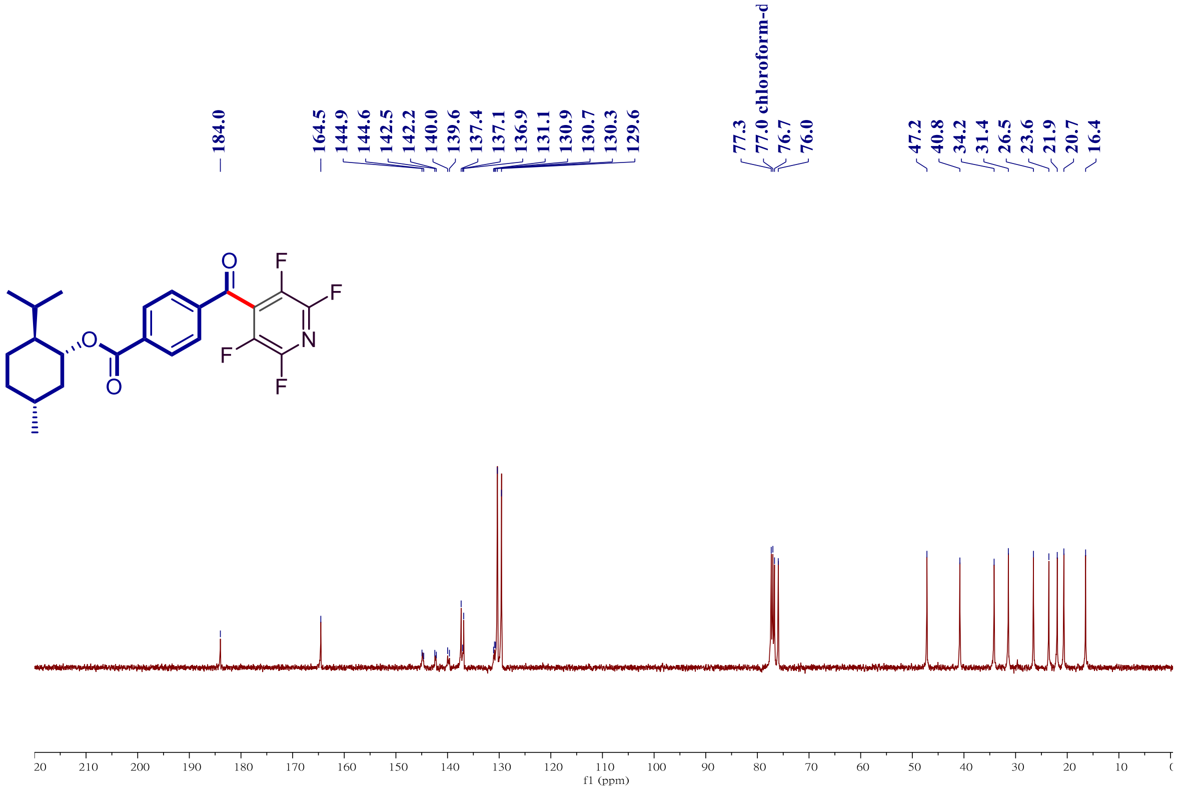

^{19}F NMR (282 MHz, $CDCl_3$) of **5a**

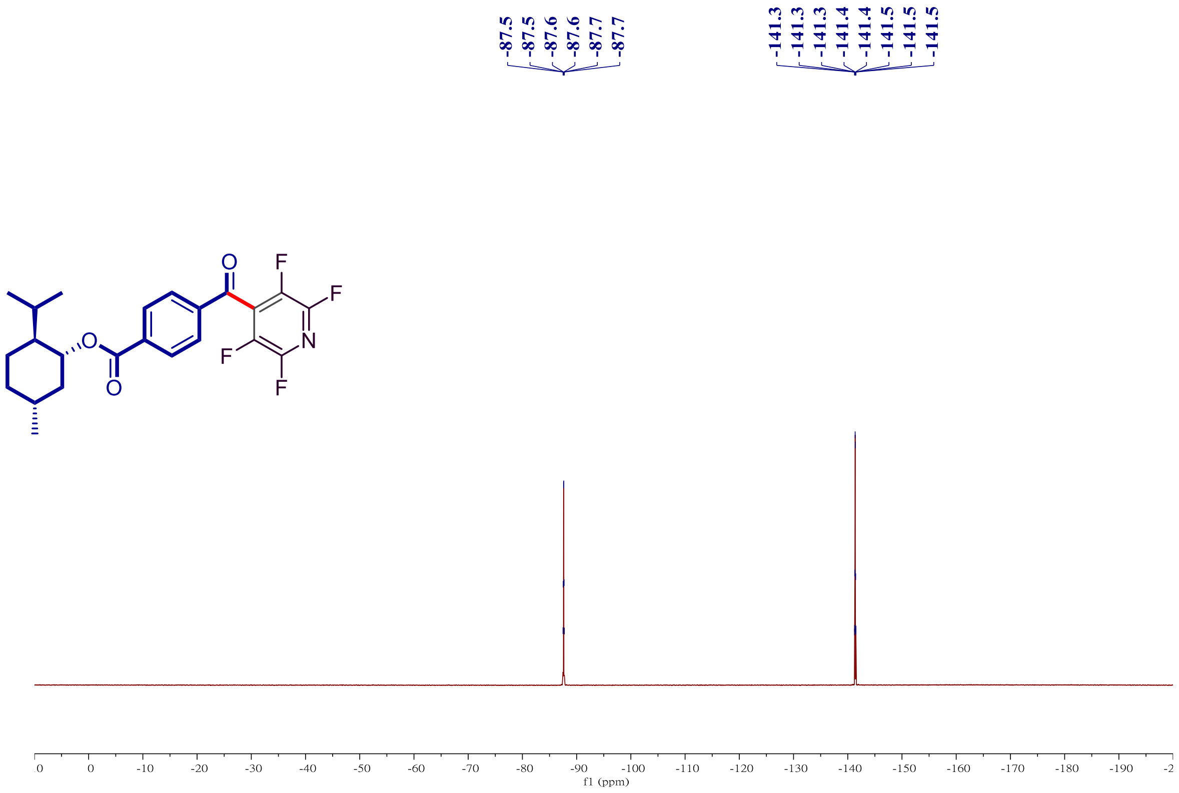

[Back to the contents]

^{1}H NMR (300 MHz, $CDCl_3$) of **5b**, See procedure

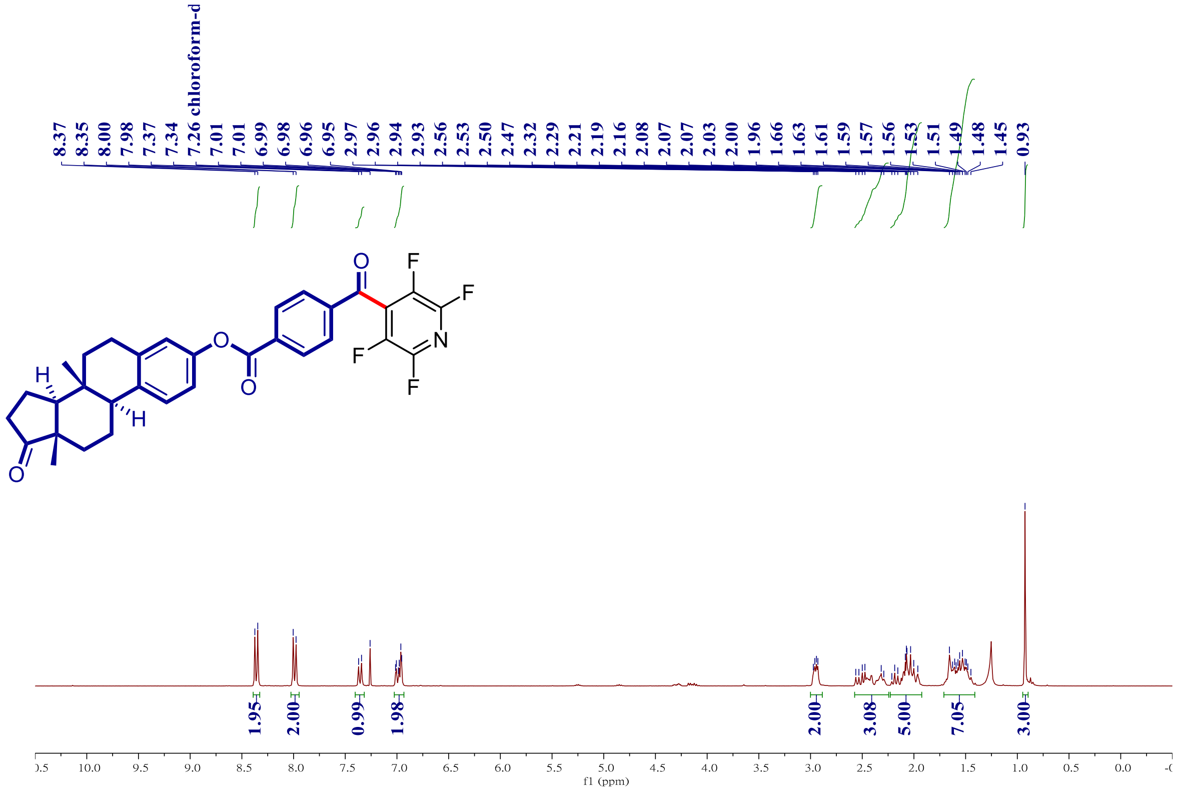

^{13}C NMR (101 MHz, $CDCl_3$) of **5b**

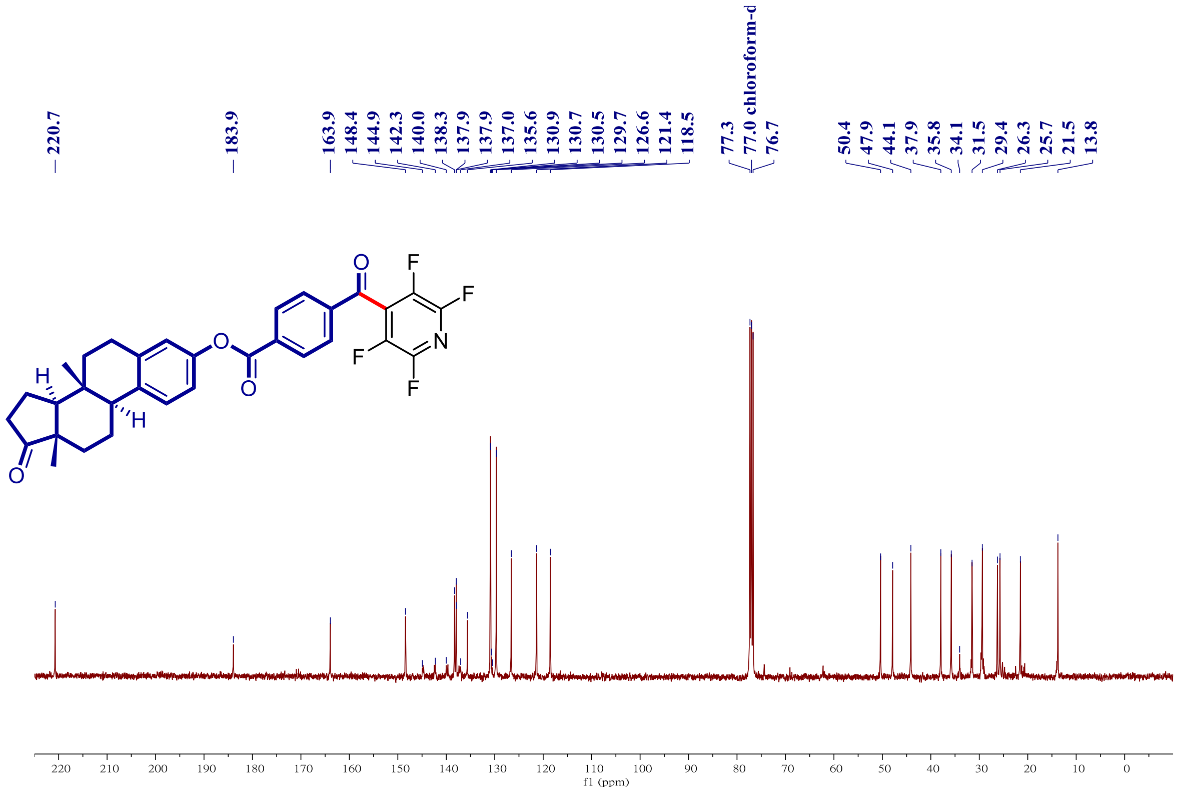

Back to the contents

^{19}F NMR (282 MHz, $CDCl_3$) of **5b**

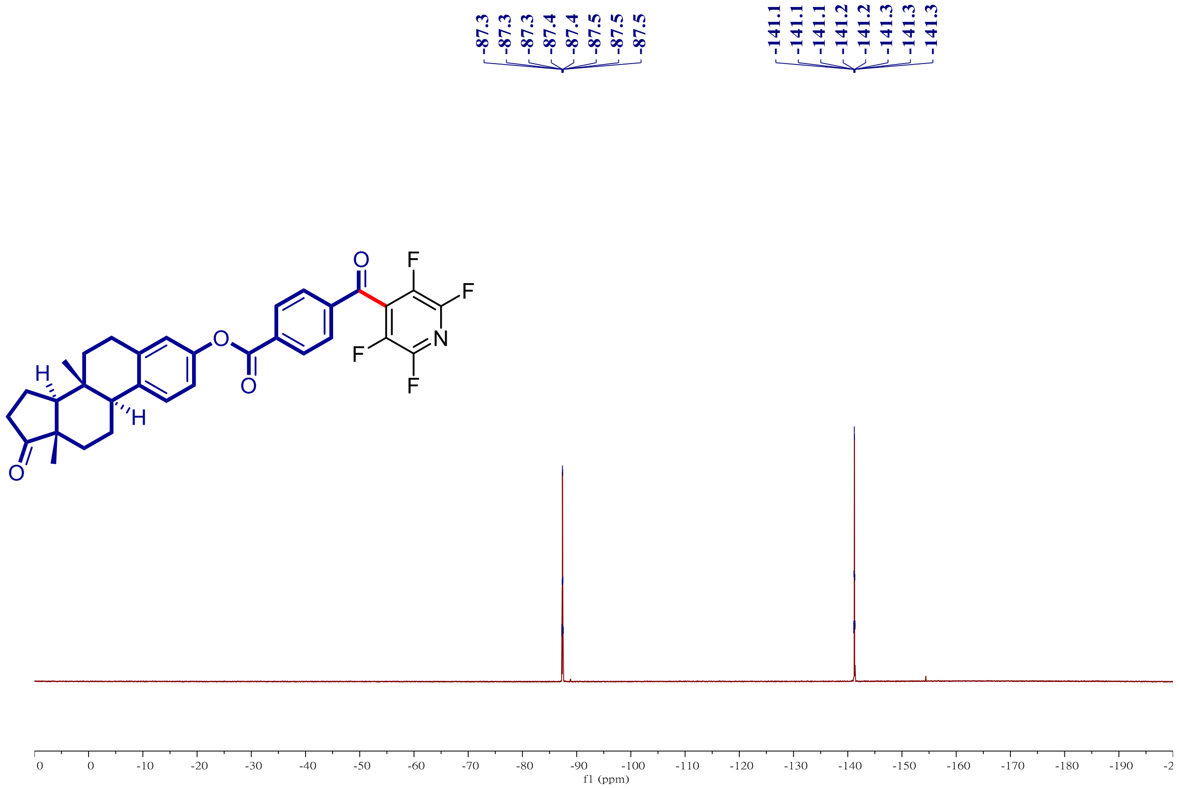

^{1}H NMR (400 MHz, $CDCl_3$) of **5c**, See procedure

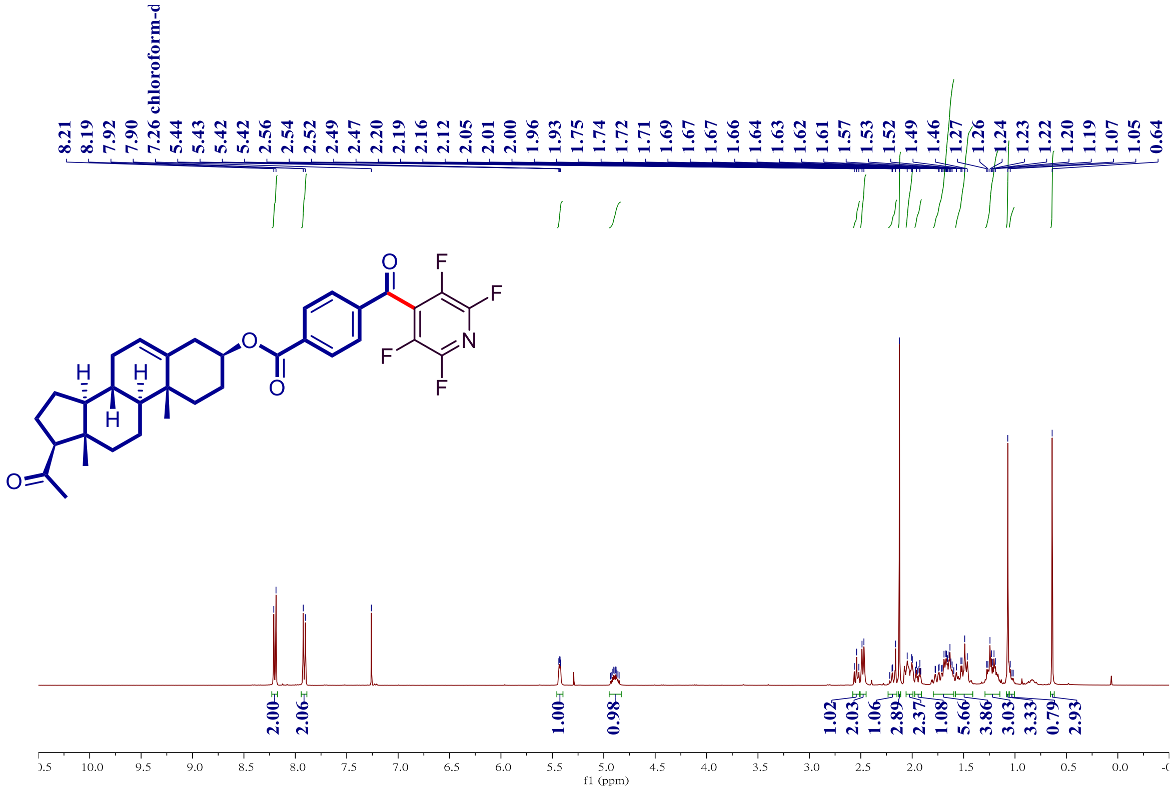

Back to the contents

^{13}C NMR (101 MHz, $CDCl_3$) of **5c**

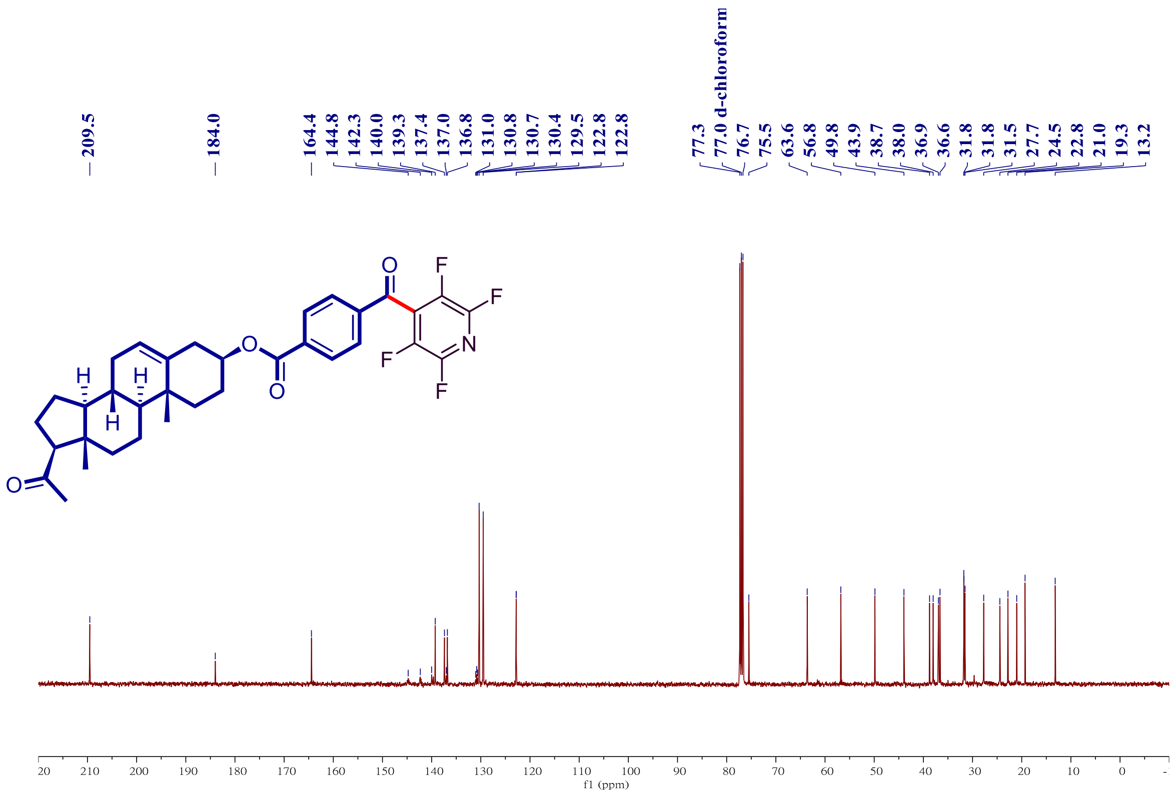

^{19}F NMR (282 MHz, $CDCl_3$) of **5c**

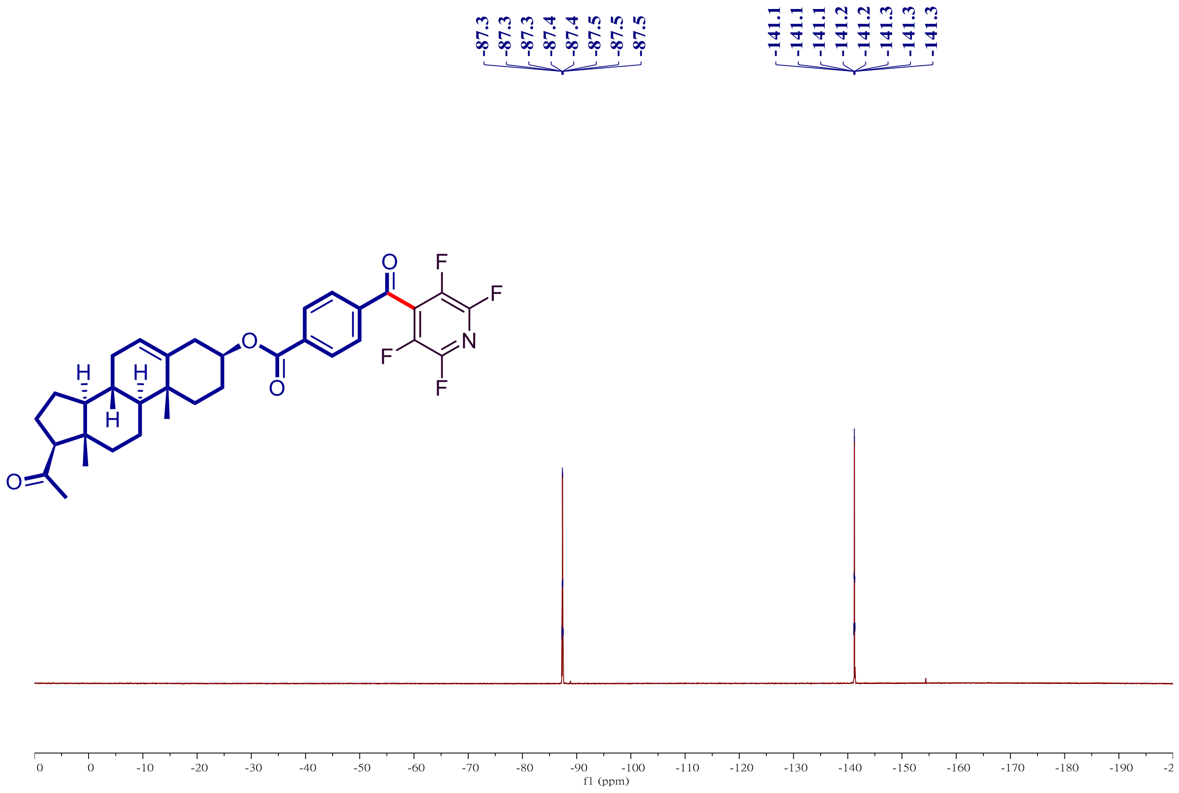

Back to the contents

^{1}H NMR (300 MHz, $CDCl_3$) of **5d**, See procedure

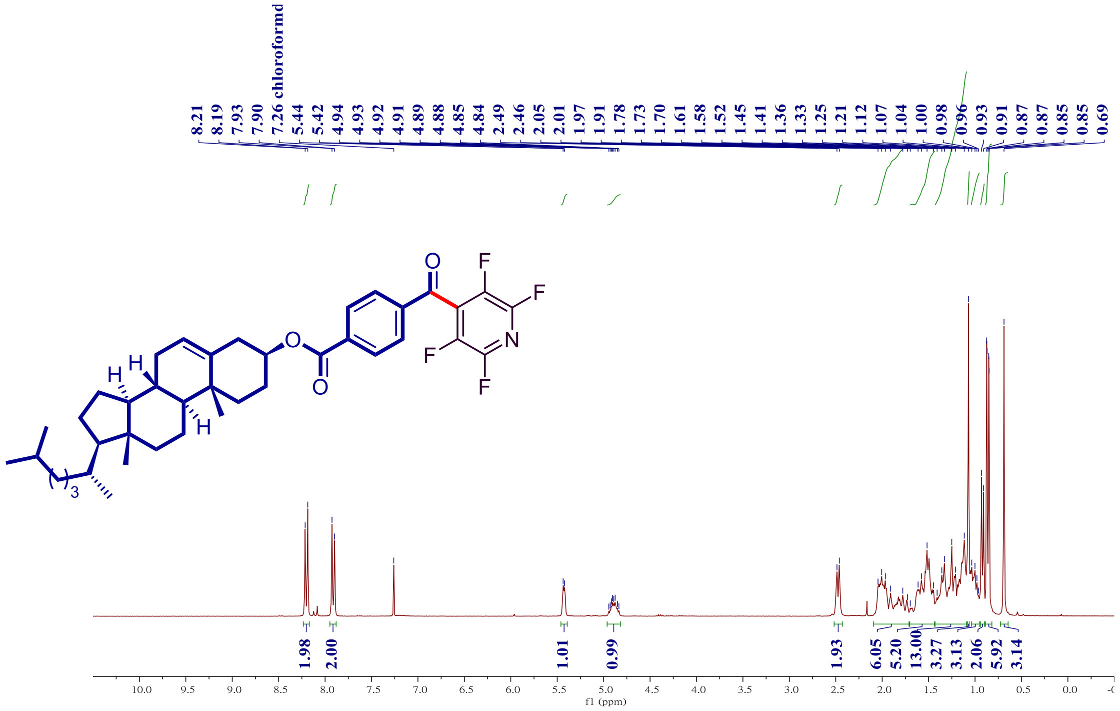

^{13}C NMR (101 MHz, $CDCl_3$) of **5d**

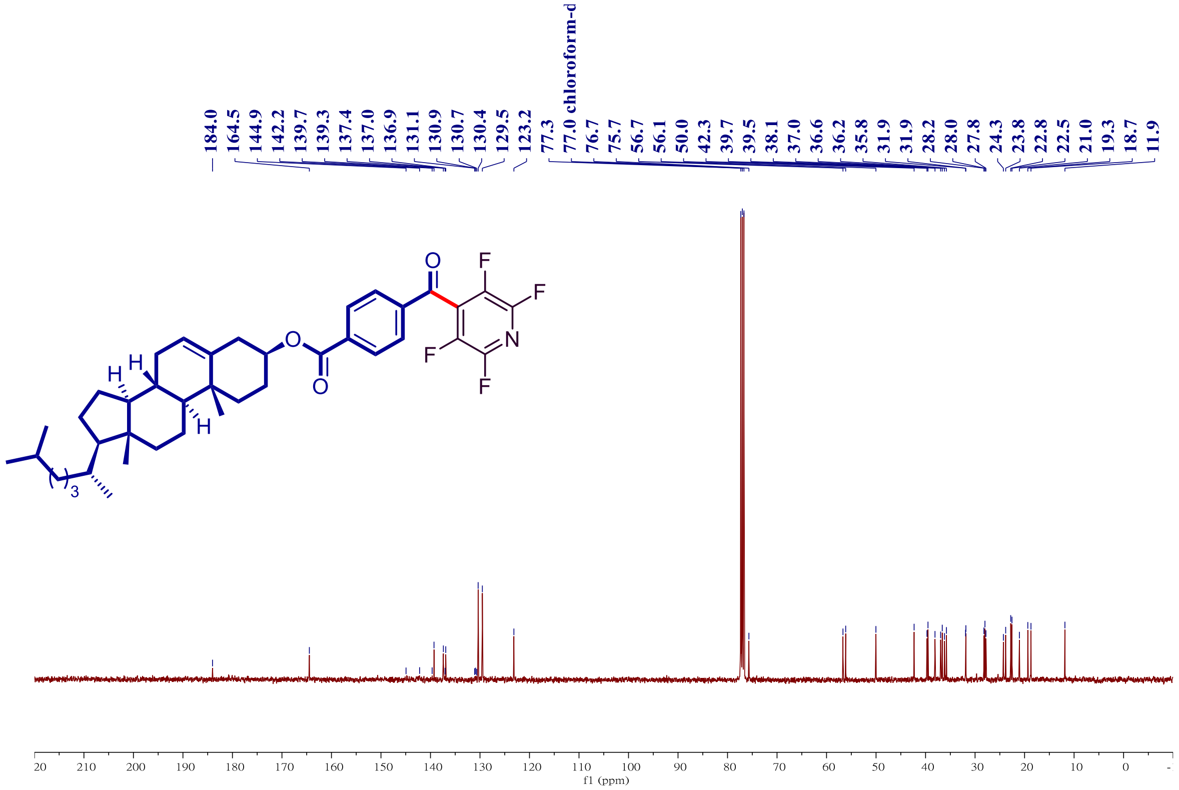

Back to the contents

^{19}F NMR (282 MHz, $CDCl_3$) of **5d**

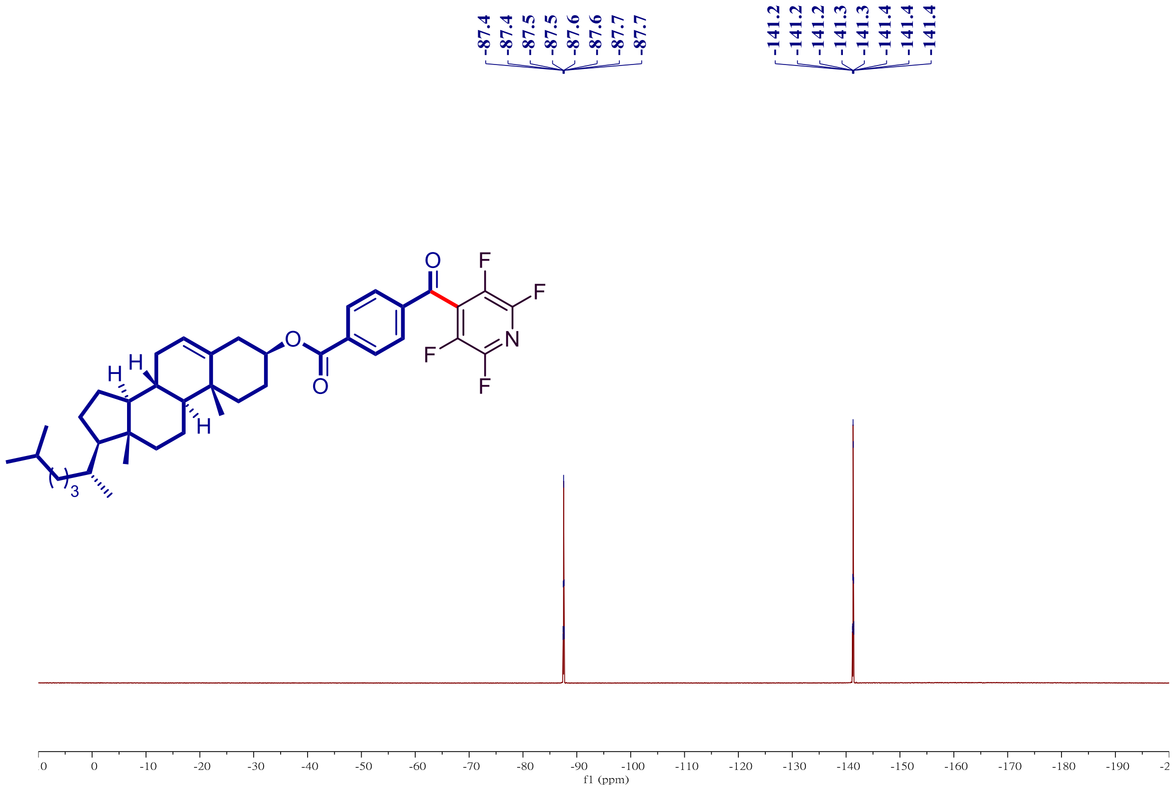

^{1}H NMR (300 MHz, $CDCl_3$) of **5e**, See procedure

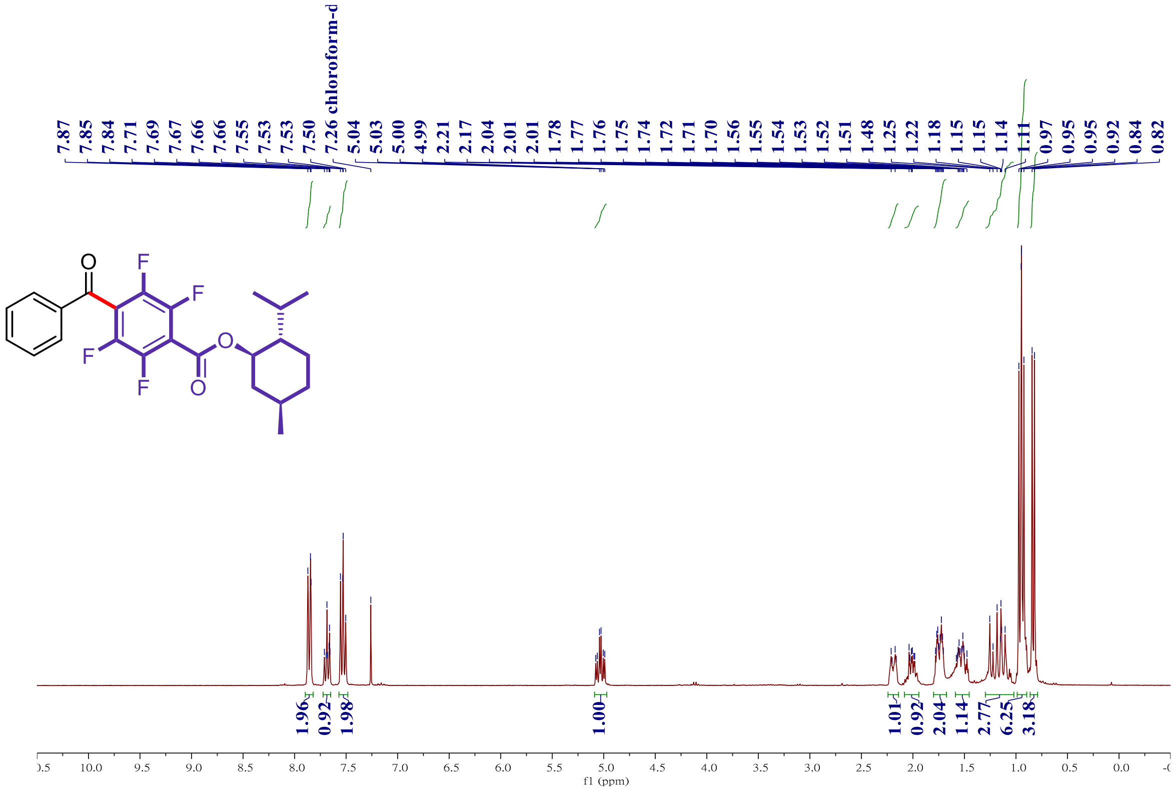

Back to the contents

^{13}C NMR (101 MHz, $CDCl_3$) of **5e**

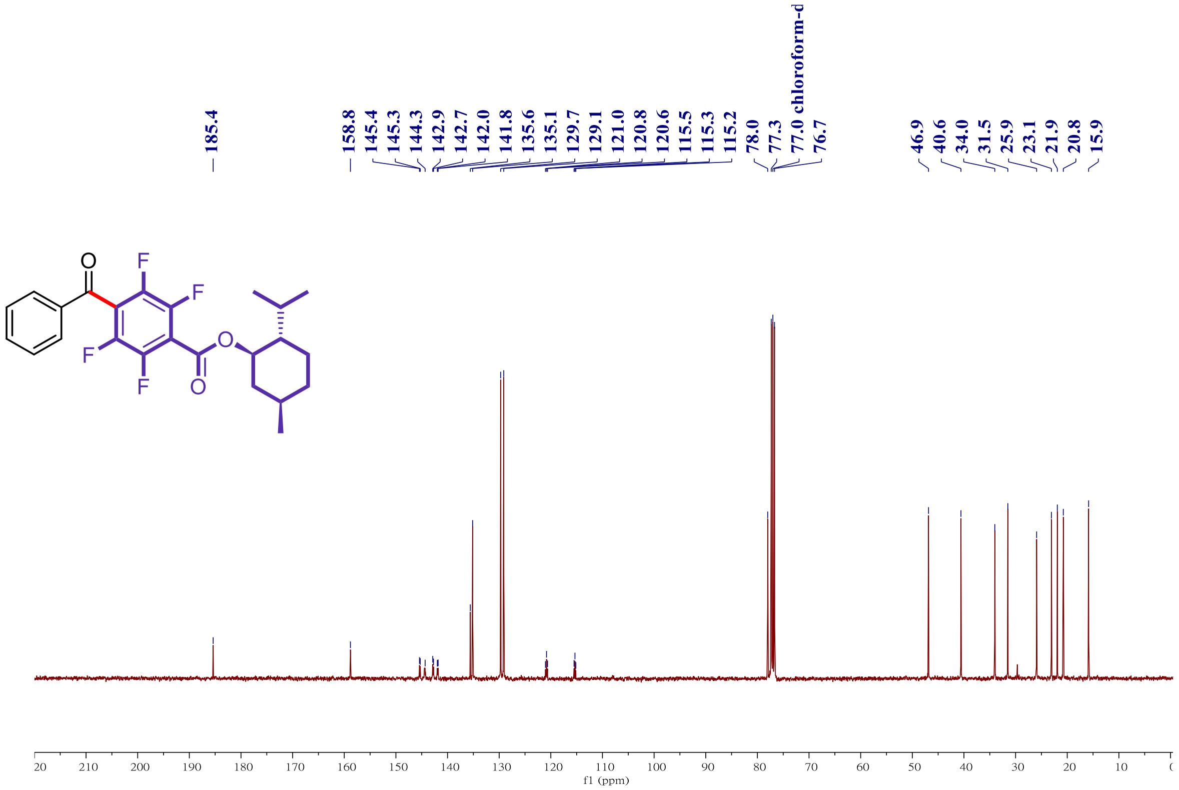

^{19}F NMR (282 MHz, $CDCl_3$) of **5e**

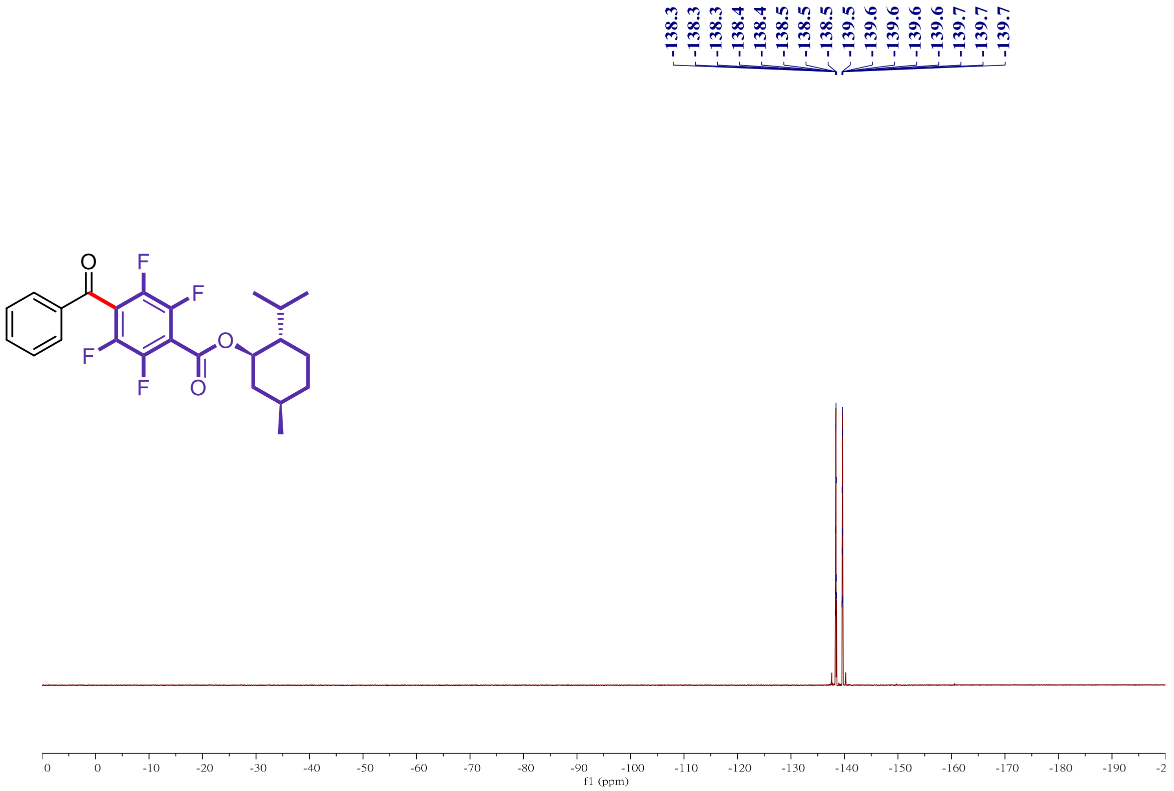

[Back to the contents](#)

^{1}H NMR (300 MHz, $CDCl_3$) of **5f**, See procedure

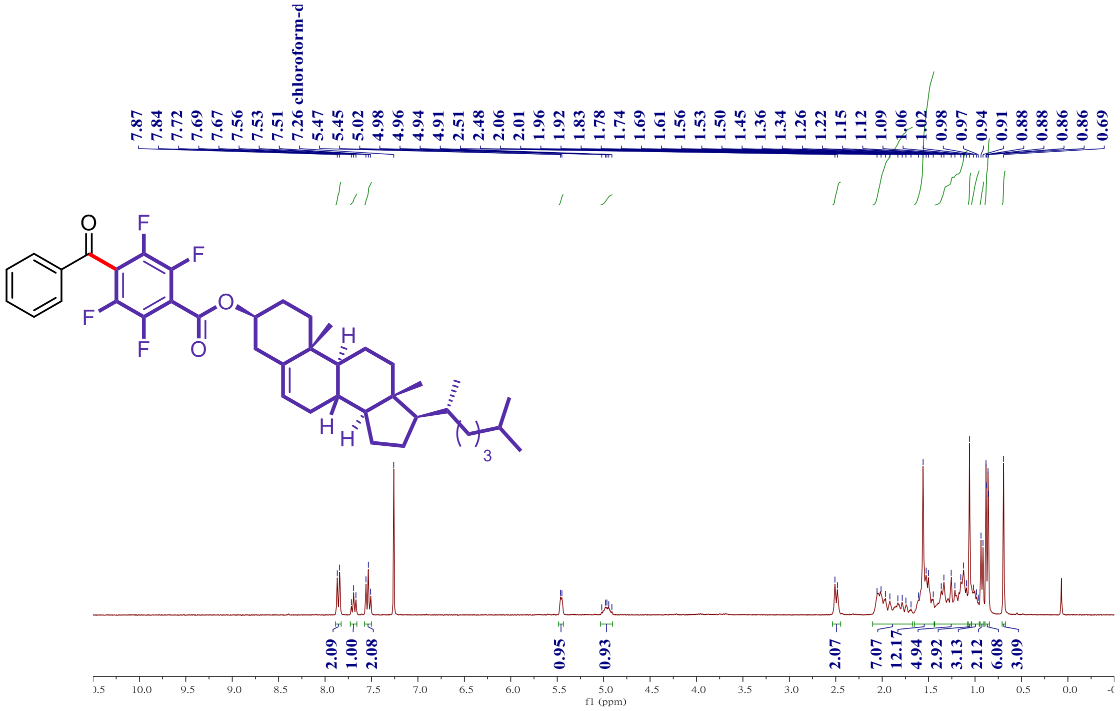

^{13}C NMR (101 MHz, $CDCl_3$) of **5f**

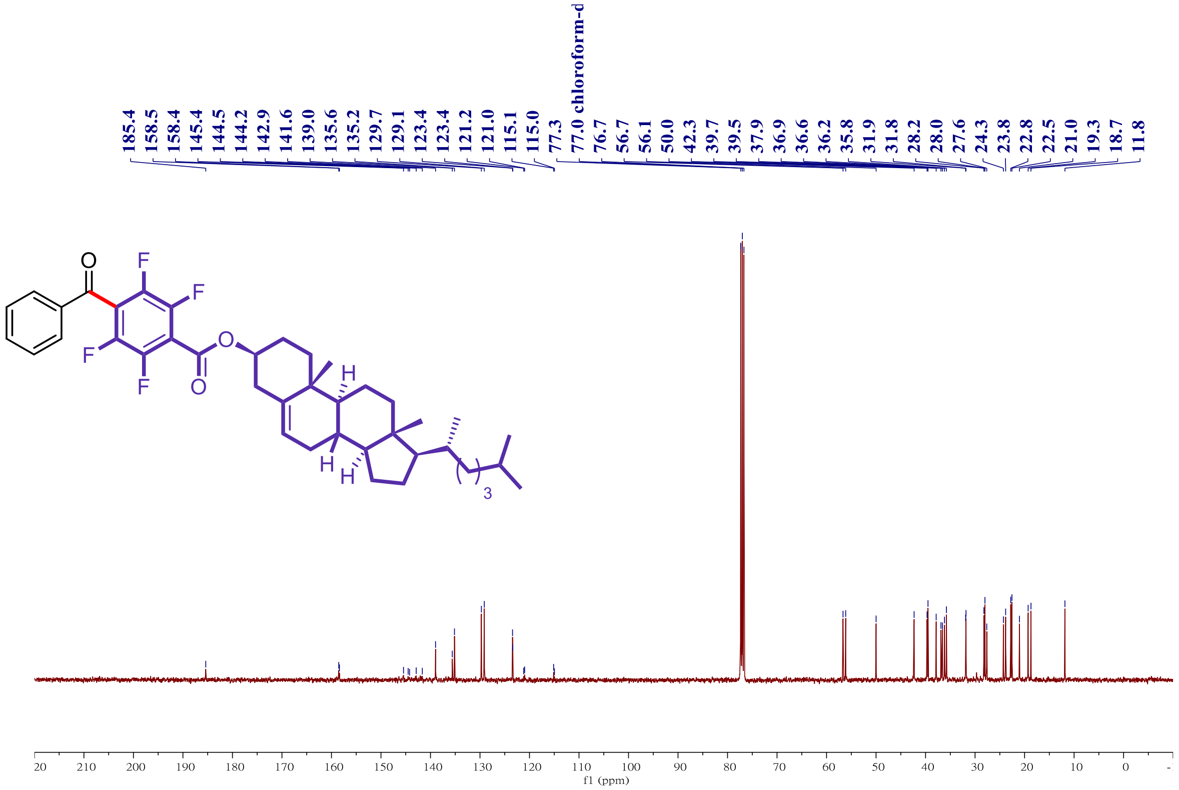

[Back to the contents](#)

^{19}F NMR (282 MHz, $CDCl_3$) of **5f**

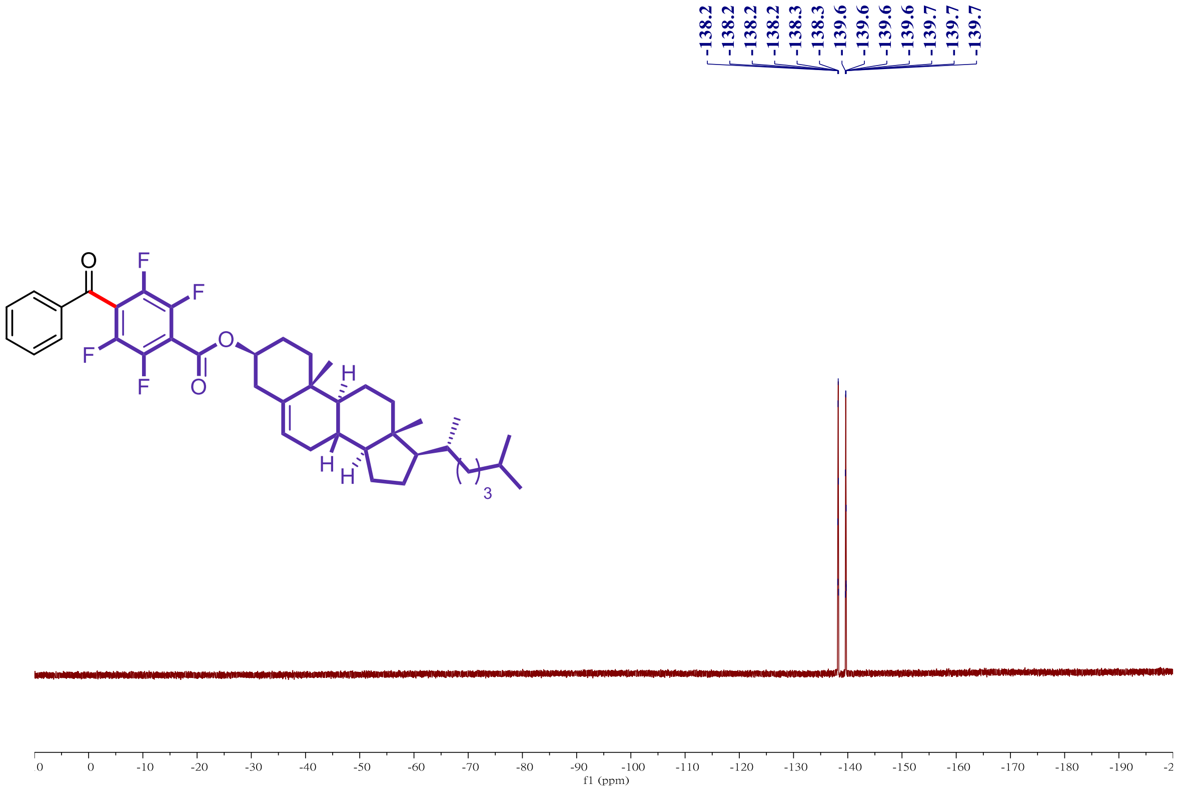

^{1}H NMR (300 MHz, $CDCl_3$) of **5g**, [See procedure](#)

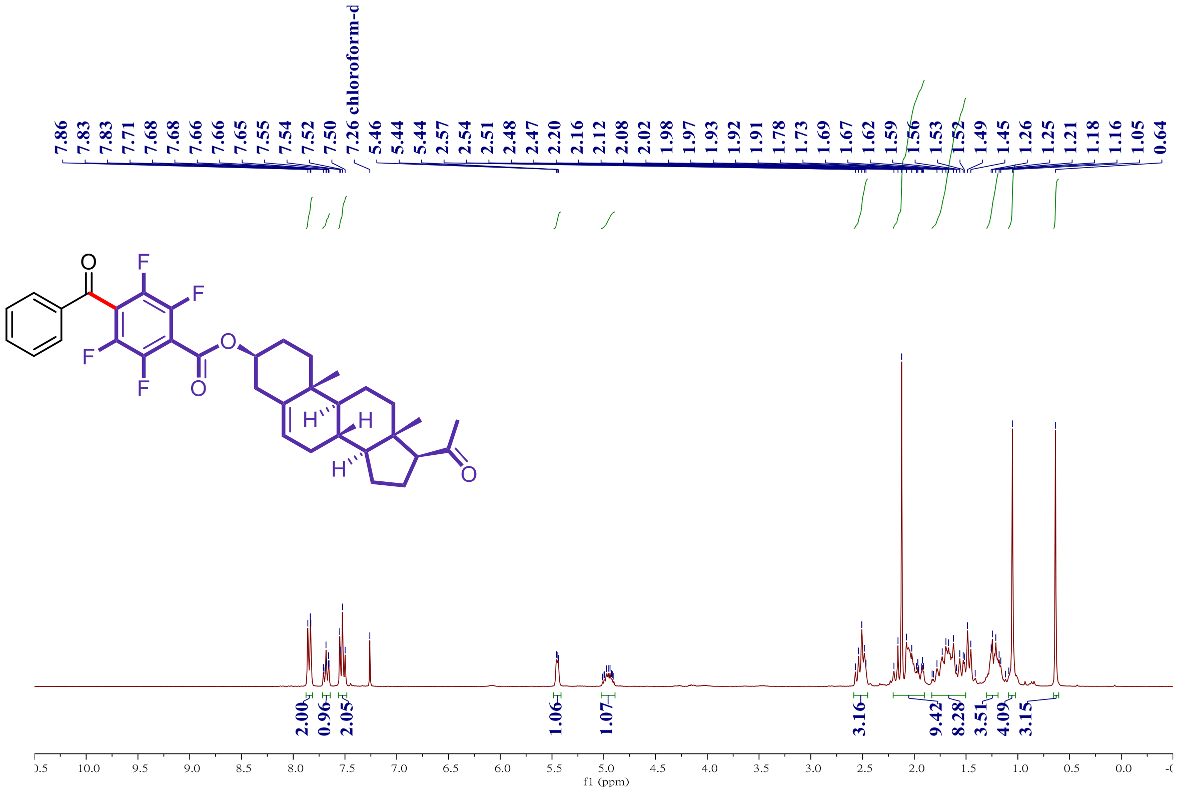

Back to the contents

^{13}C NMR (101 MHz, $CDCl_3$) of **5g**

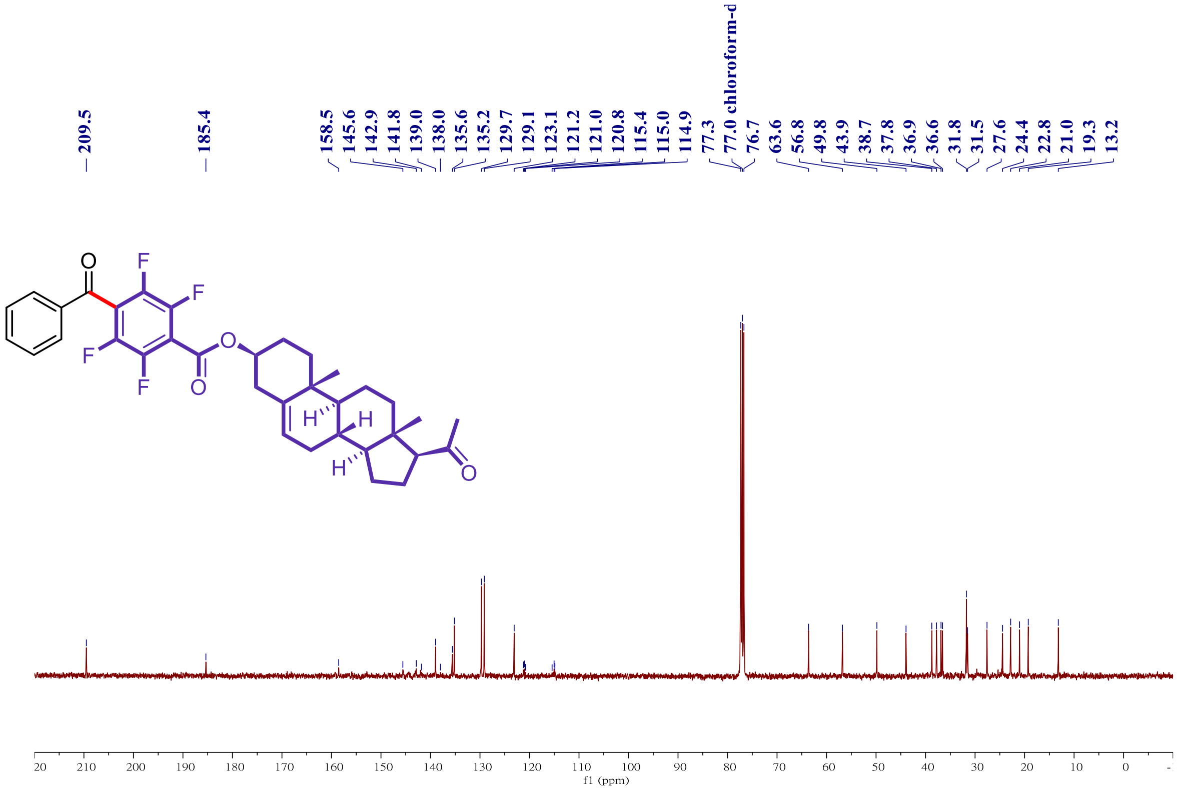

^{19}F NMR (282 MHz, $CDCl_3$) of **5g**

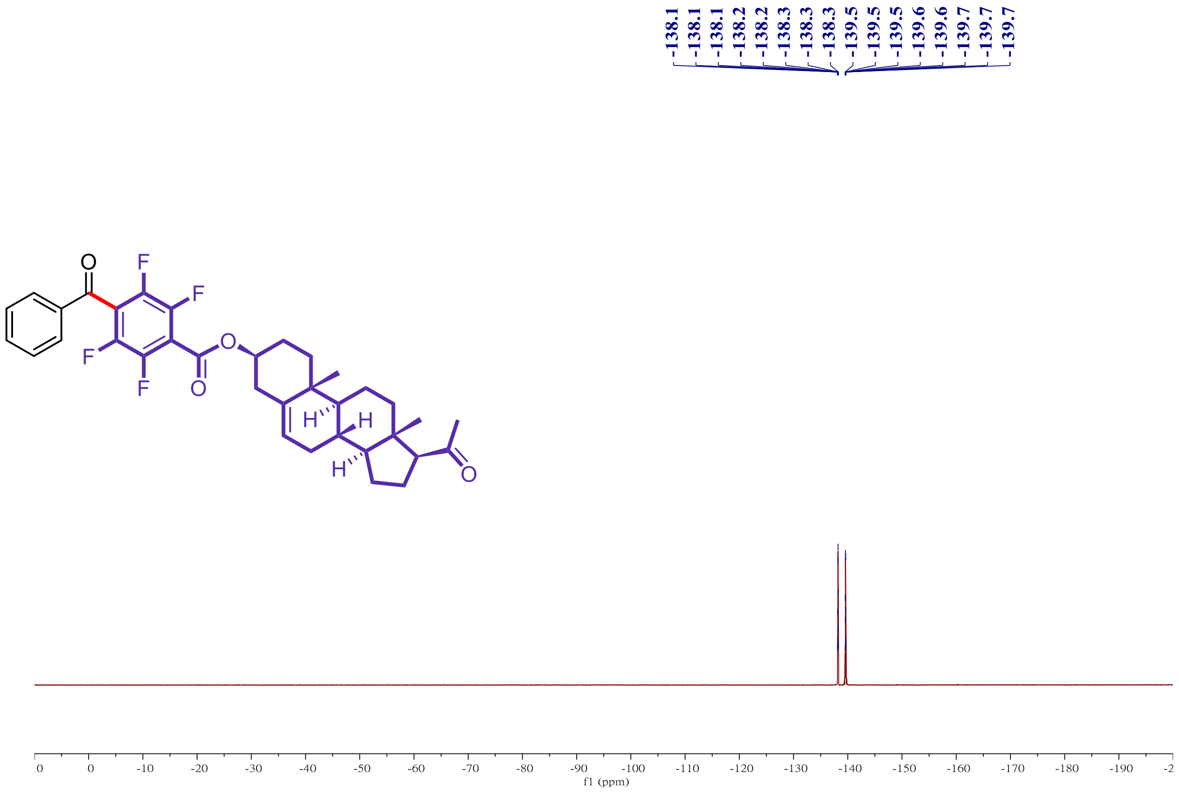

[Back to the contents]

^{1}H NMR (300 MHz, $CDCl_3$) of **6**, See procedure

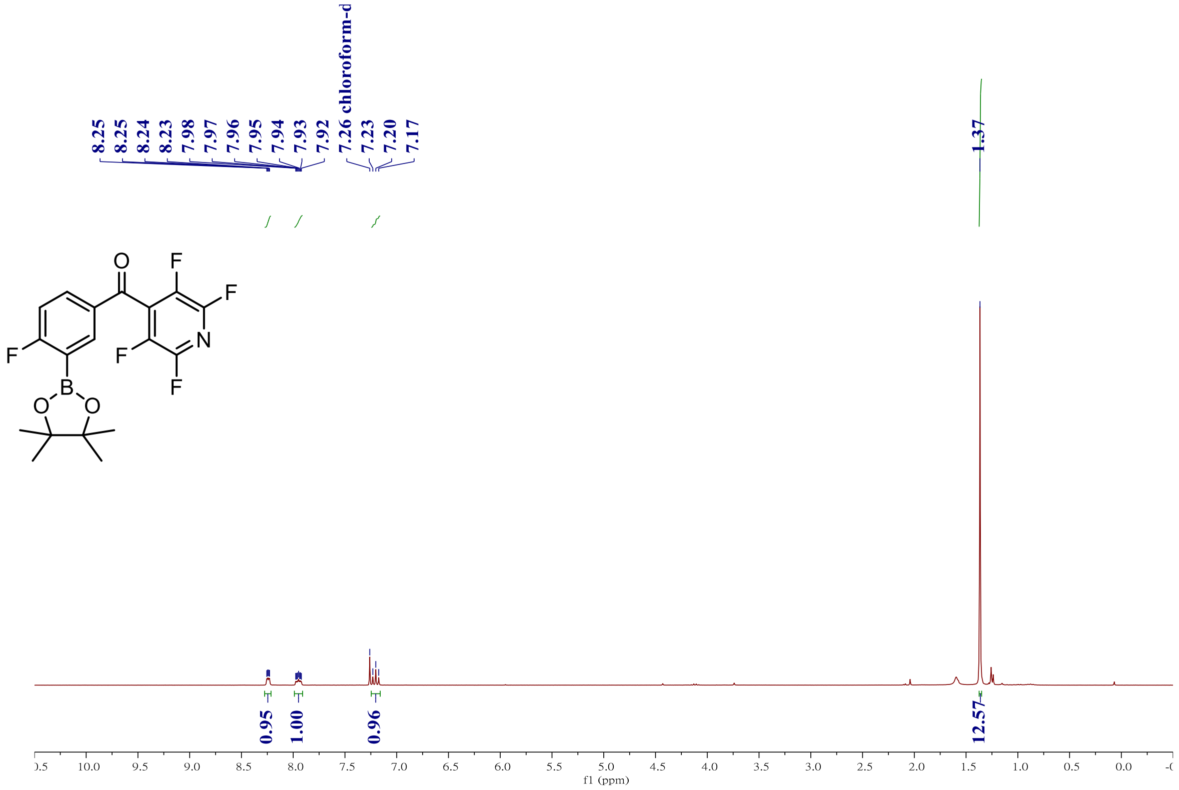

^{13}C NMR (101 MHz, $CDCl_3$) of **6**

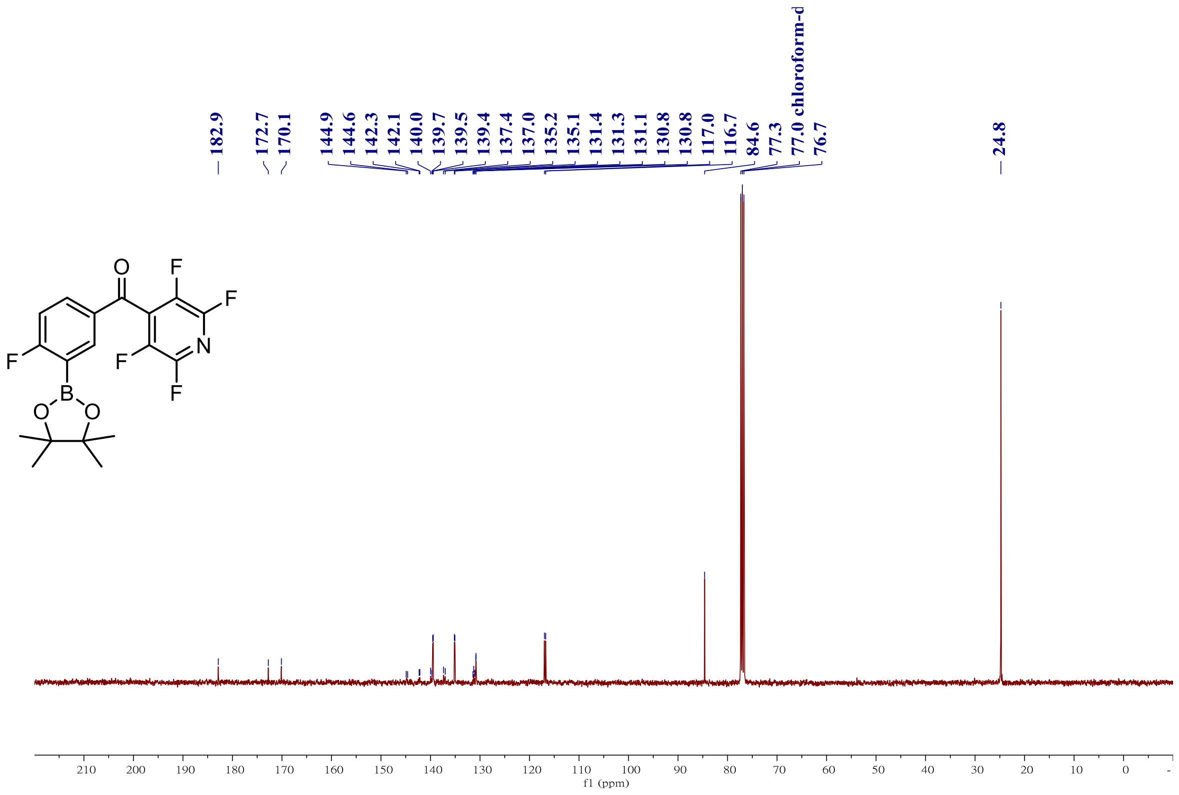

[Back to the contents](#)

^{19}F NMR (282 MHz, $CDCl_3$) of **6**

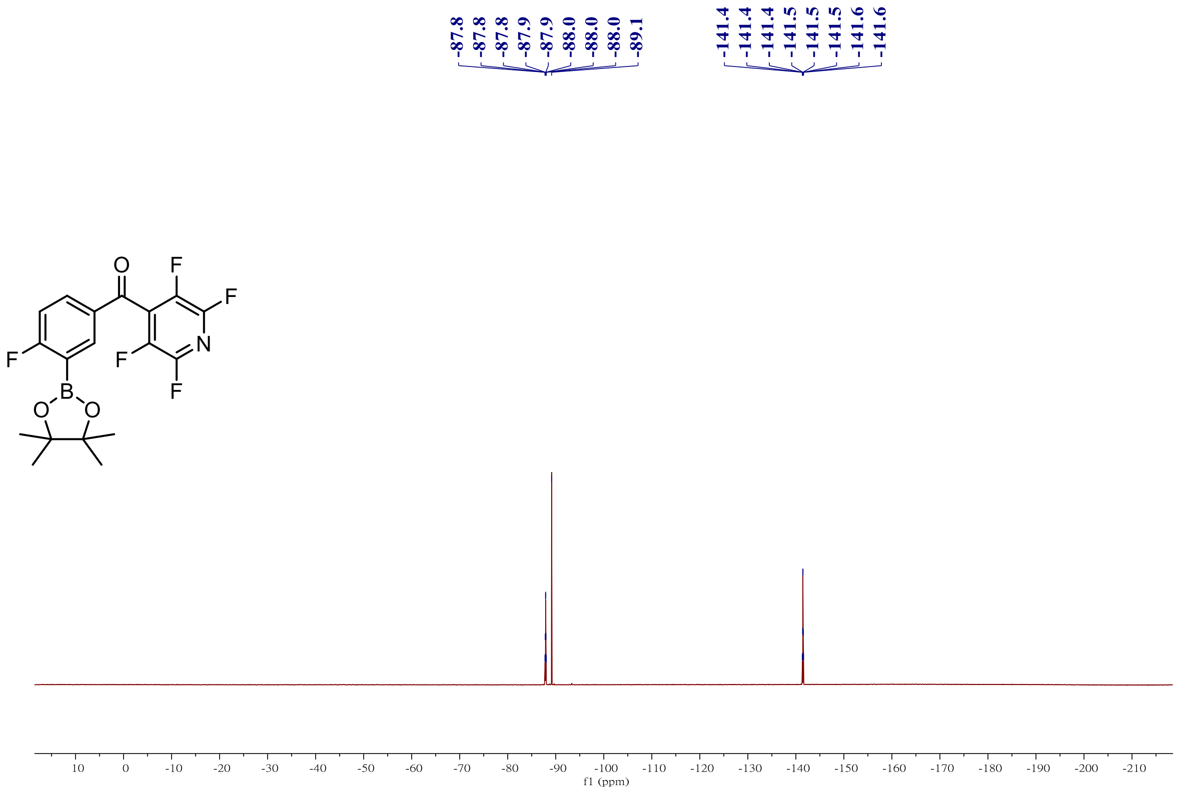

^{1}H NMR (300 MHz, DMSO-d_6) of **7**, [See procedure](#)

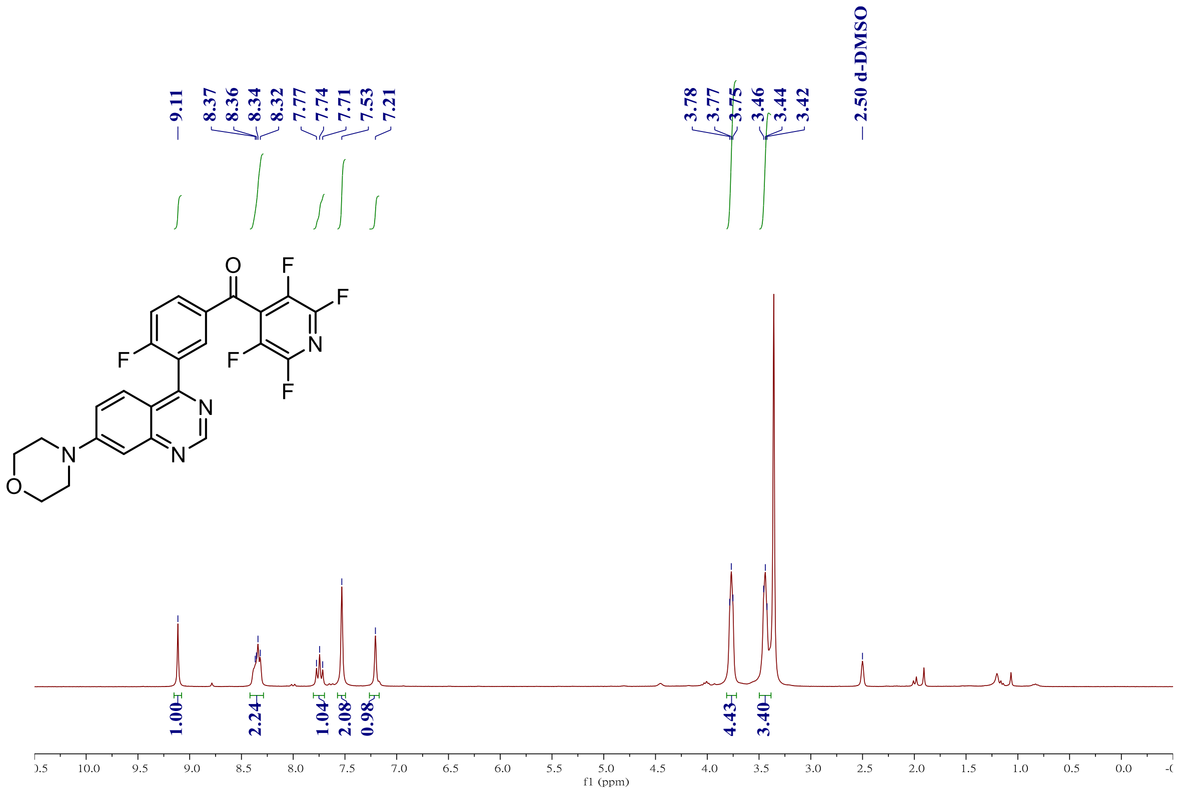

^{13}C NMR (101 MHz, DMSO-d_6) of **7**

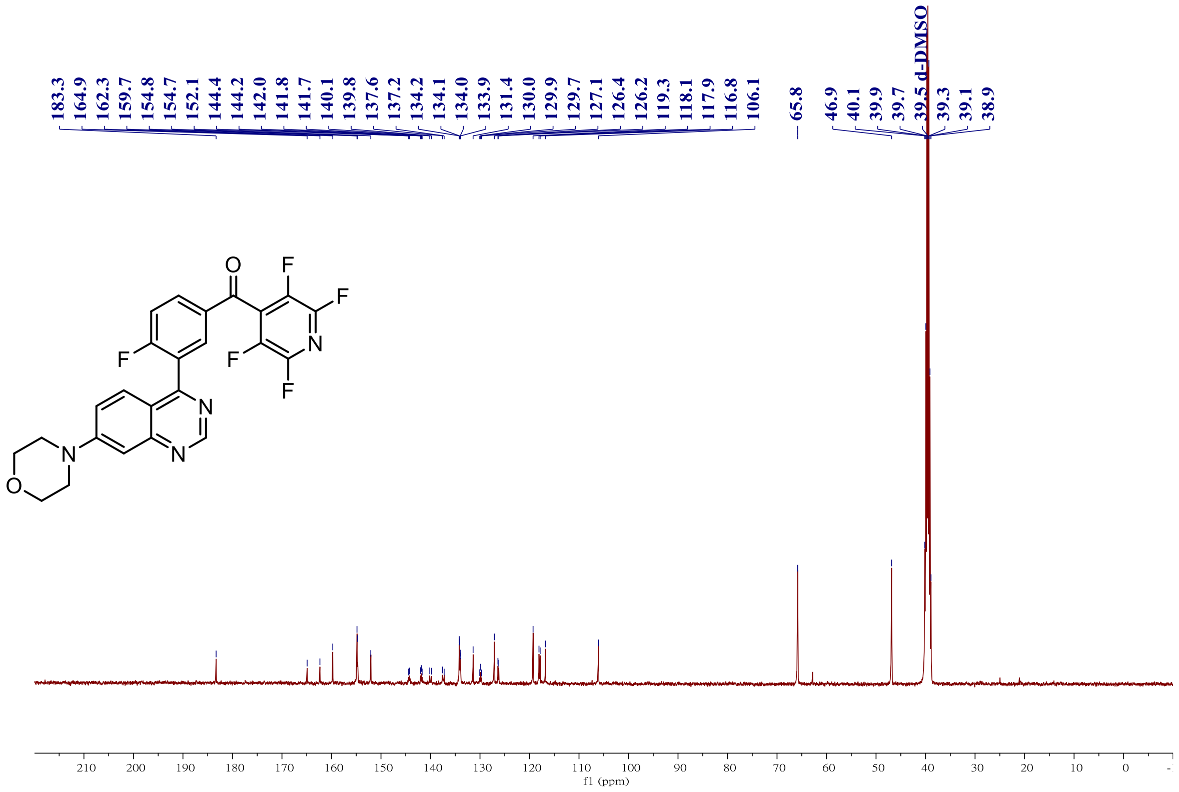

^{19}F NMR (282 MHz, DMSO-d_6) of **7**

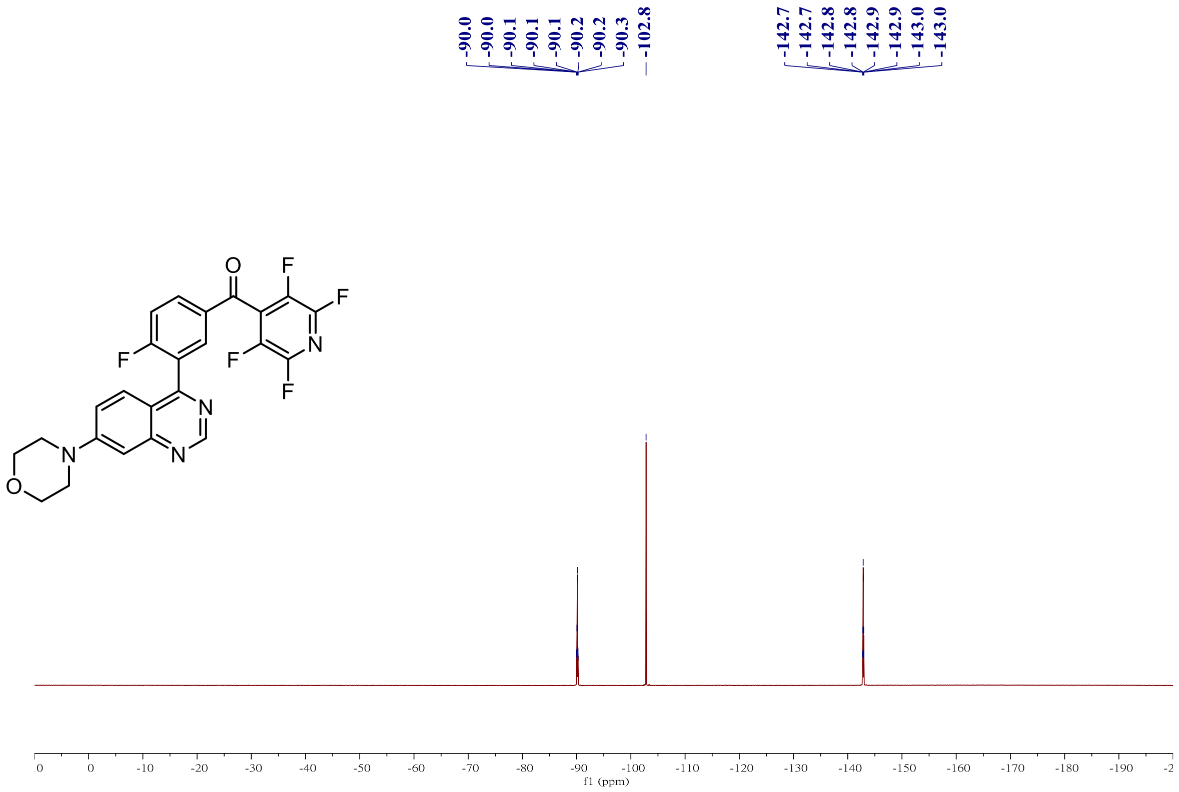

[Back to the contents]

^{1}H NMR (300 MHz, DMSO-d_6) of **PF-D1**, See procedure

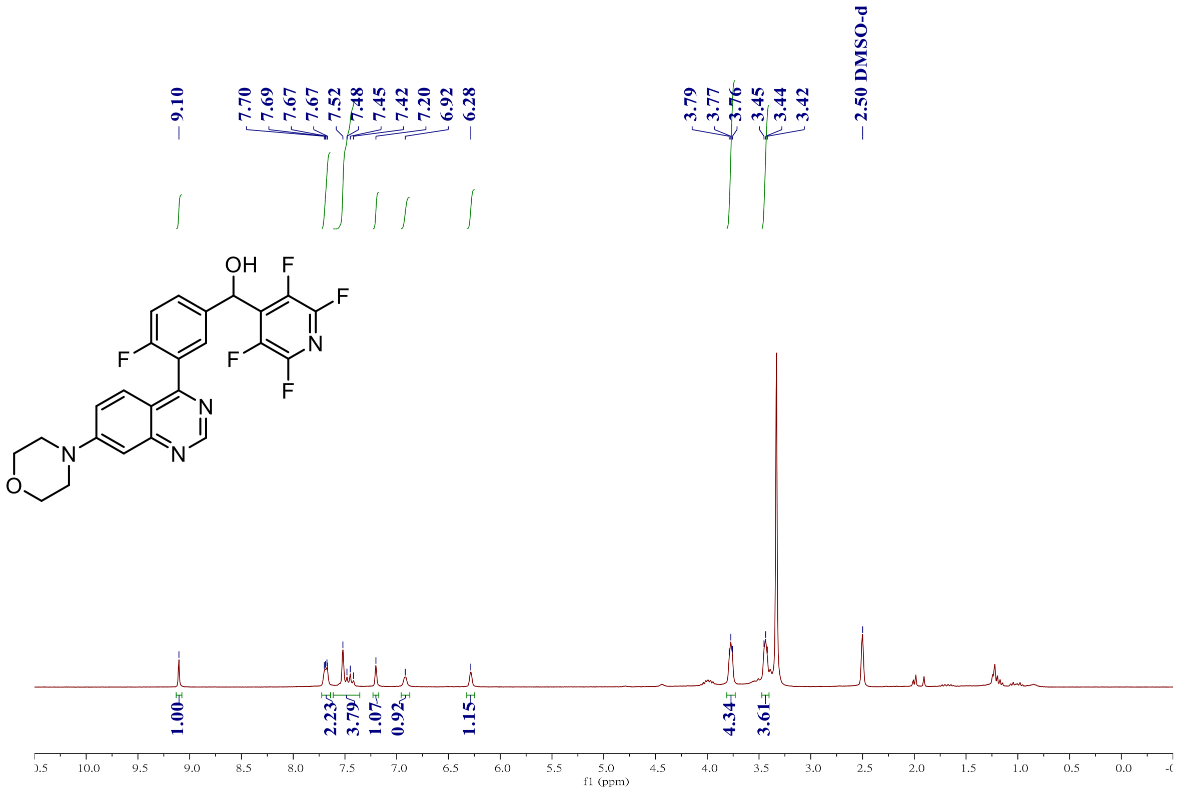

^{13}C NMR (101 MHz, DMSO-d_6) of **PF-D1**

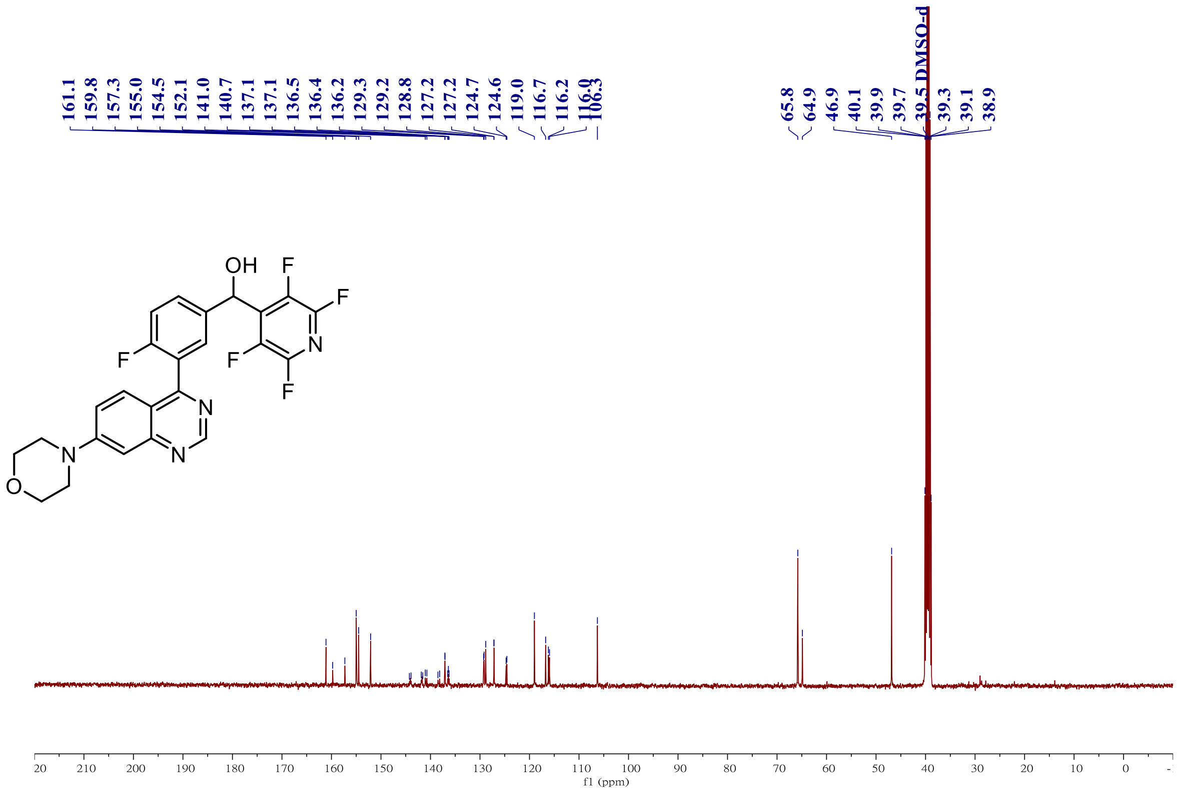

Back to the contents

^{19}F NMR (282 MHz, DMSO-d_6) of **PF-D1**

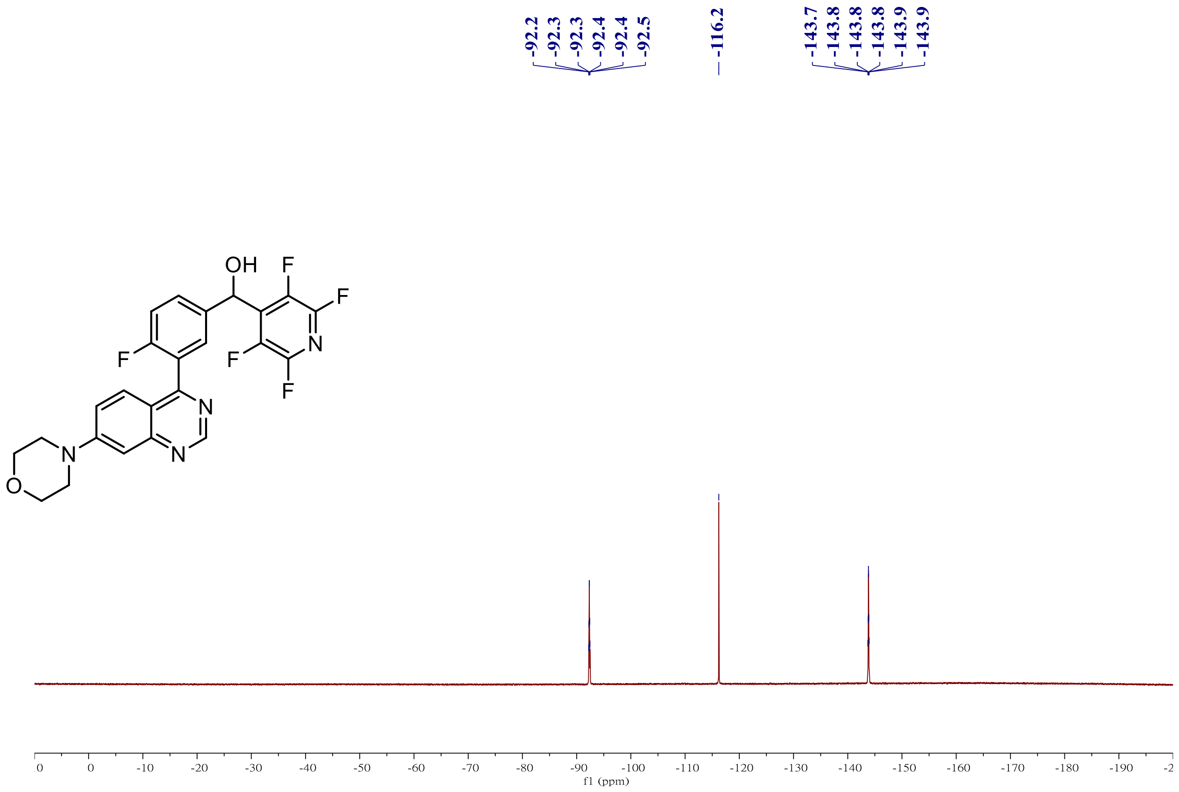

Supplement: Supplementary file 2 — ol4c00677_si_002.pdf [file ol4c00677_si_002.pdf]
