# Supplementary material for: FluoroFusion: NHC-Catalyzed Nucleophilic Aromatic Substitution Reaction Unveils Functional Perfluorinated Diarylmethanones
Source: Org Lett. 2024 Mar 8;26(11):2338–42. doi: 10.1021/acs.orglett.4c00677 (PMC10964231; doi:10.1021/acs.orglett.4c00677)
Supplement: Supplementary file 1 — ol4c00677_si_001.zip [file ol4c00677_si_001.zip › NHC SNAr OL/作者資料.docx]

| **Name** | **E-mail** |  |
| --- | --- | --- |
| Chenglin Chan | eric04011203@gmail.com |  |
| Shaochi Lee | shaochi.lee@kaust.edu.sa |  |
| Peishan Lin | anna96044chem@gmail.com |  |
| Radyn Vanessa Phaz P.Tapales | d102630006@student.nsysu.edu.tw |  |
| JiaSyuan Li | syaun0713@gmail.com |  |
| ChunAn Lai | a0976387096@gmail.com |  |
| Jyhtsung Lee | jtlee@faculty.nsysu.edu.tw |  |
| Chienhung Li | chli@kmu.edu.tw |  |
| HsuanHung Liao | hsuan-hung.liao@mail.nsysu.edu.tw |  |
