# Supplementary material for: FluoroFusion: NHC-Catalyzed Nucleophilic Aromatic Substitution Reaction Unveils Functional Perfluorinated Diarylmethanones
Source: Org Lett. 2024 Mar 8;26(11):2338–42. doi: 10.1021/acs.orglett.4c00677 (PMC10964231; doi:10.1021/acs.orglett.4c00677)
Supplement: Supplementary file 1 — ol4c00677_si_001.zip [file ol4c00677_si_001.zip › NHC SNAr OL/推薦審稿人.docx]

| Tülay Yildiz | Turkey | CF_3_, NHC |  |
| --- | --- | --- | --- |
|  |  |  |  |
| Sensuke Ogoshi | Japan | SNAr |  |
| Masaya Sawamura | Japan | SNAr |  |
| Makoto Yasuda | Japan | F |  |
| Chung-Yang Huang | Japan (Taiwan) | F, NHC |  |
|  |  |  |  |
| Hanyong Bae | Korea | NHC base cat |  |
| Sung You Hong | Korea | F |  |
|  |  |  |  |
| Renzo Luisi | Italy | F |  |
|  |  |  |  |
| Alastair J. J. Lennox | England | SNAr |  |
|  |  |  |  |
| Hang Shi | China | SNAr |  |
| Wei shu | China | NHC photo |  |
| Liang-Qiu Lu | China | Cu cat diazole |  |
| Wangqing Kong | China | F |  |
| 何林 | China | NHC |  |
| Gavin Chit Tsui | China | F |  |
|  |  |  |  |
|  |  |  |  |
|  |  |  |  |
|  |  |  |  |
