# Supplementary material for: FluoroFusion: NHC-Catalyzed Nucleophilic Aromatic Substitution Reaction Unveils Functional Perfluorinated Diarylmethanones
Source: Org Lett. 2024 Mar 8;26(11):2338–42. doi: 10.1021/acs.orglett.4c00677 (PMC10964231; doi:10.1021/acs.orglett.4c00677)
Supplement: Supplementary file 1 — ol4c00677_si_001.zip [file ol4c00677_si_001.zip › NHC SNAr OL/Cover Letter-Professor Kozlowski v2(OL).docx]

**Hsuan-Hung Liao, Dr.rer.nat.**

Associate Professor, Department of Chemistry
National Sun Yat-sen University

70 Lien-hai Rd., Kaohsiung 80424, Taiwan, ROC

Email：hsuan-hung.liao@mail.nsysu.edu.tw

Tel：+886(07)525-2000 ext.3947

20 February 2024

*Professor Marisa C. Kozlowski*

*Editor-in-Chief*

*Organic Letters*

*Department of Chemistry*

*University of Pennsylvania*

*United States*

Dear Professor Kozlowski,

I am enclosing herewith our manuscript, entitled "**FluoroFusion: NHC-Catalyzed Nucleophilic Aromatic Substitution Reaction Unveils Functional Perfluorinated Diarylmethanones**," for your consideration for publication in *Organic Letters.* The manuscript, with the manuscript ID cs-2024-002364, was previously submitted to ACS Catalysis and received a rejected submission decision. However, we have taken into account the comments and suggestions of the previous reviewers and made significant modifications to the manuscript. Moreover, we just want to emphasize that our manuscript focuses on the development of a catalytic protocol that is mild, facile, and metal-free for the synthesis of functional perfluorinated diarylmethanones, which have emerged as compounds of paramount significance due to their unique properties and versatile applications in pharmaceuticals, agrochemicals, and materials science.

The following comments from the previous reviewers were copied and pasted below, and our responses were immediately inserted as we see fit (written in blue font).

------ Comments ------

Reviewer 1:

The manuscript by Prof. Liao and co-workers described a reaction to synthesize a polyfluorinated diarylmethanones through NHC-catalyzed nucleophilic aromatic substitution reaction.
NHC-catalyzed umpolung strategy using aldehydes is well-known, there are no methods for synthesizing polyfluorinated ketone compounds via metal-free reaction condition from aldehydes is known. The synthetic route to ketones from aldehydes often involves reacting compounds such as aryl boronic acid, aryl-MgBr, alkyl-MgBr, or halogen-substituted benzene with aldehydes. The reaction conditions frequently include metals, such as starting materials or a palladium catalyst, and there are scarce publications on approaching polyfluorinated diarylmethanones. The authors have developed a protocol that allows direct access to polyfluorinated diarylmethanones under mild and metal-free reaction conditions. Additionally, a suitable strategy for the synthesis of a bioisosteric analog for a drug candidate was proposed using this synthetic method, and its potential use as electrolyte additives in lithium-ion batteries was confirmed. Overall, this reviewer feels the manuscript is acceptable in ACS Catalysis after minor revision.

*Response: Thank you, Reviewer 1, for your positive feedback and for recognising that our manuscript meets the standards of ACS Catalysis.*

Only a few issues should be addressed.

1. Compound 4a was obtained in 92% yield when reacted on a 0.1 mmol scale, but only 66% yield was obtained when reacted on a gram scale. Is there a reason?

*Response: We made a change in the new version of the manuscript - the compound previously known as* ***4a*** *is now called compound* ***3a*** *for better clarity.*

*To address this query of reviewer 1, we did comprehensive TLC monitoring, and the results indicated a successful transformation of the reaction. However, the observed reduction in yields can be attributed to challenges encountered during the solvent removal step. The unique structural features of perfluoroaromatic compounds, characterized by their lower boiling points, present a significant obstacle in the large-scale solvent removal process. Specifically, the extended vacuuming required, given that we aren’t able to use heat due to the distinctive structure of perfluoroaromatic compounds, may inadvertently result in prolonged exposure. This extended duration in the vacuum increases the likelihood of azeotropic interactions, leading to the partial carryover of compound* ***3a*** *by the solvent and subsequent decrease in overall yield. In contrast, the scaled-up synthesis of compound* ***3s****, which incorporates heavy atoms such as Br in its structure, exhibited consistent results in the large-scale process. Notably, there were no significant variations, and the yield increased from 66% for 0.1mmol to 71% for the gram scale.*

2. In the substrate scope part, most types of aryl aldehyde were used only compounds with EWG group. Additionally, even in the perfluoroarens scope, only EWG-substituted substrates are applicable. Did authors try using different EDG-substituted substrates? If it didn't work out, some comments should be added.

*Response: In our catalytic system, we utilized the EDG group in both aryl aldehyde and perfluoroarenes (compounds* ***1a'*** *and* ***2c'****). However, we observed the absence of the desired product in both cases.* *In the case of perfluoroarenes with EDG being an unsuccessful substrate can be based on the fact that in S_N_Ar-type reactions, electron-withdrawing groups (EWG) are required to increase the electron deficiency, and adding EDG diminishes this effect. Hence, the reaction did not proceed as expected. Conversely, employing aryl aldehyde with EDG not only resulted in the absence of the desired product but also failed to produce the benzoin condensation product (this was confirmed by TLC and GC-MS data). The generation of the Breslow intermediate failed due to the increased electron density of benzaldehydes. This increased electron density of benzaldehydes caused the N-heterocyclic carbene (NHC) attack to fail.*

*The discussion with comprehensive data and specifics on these findings is in the supplementary information, Table S2.8.*

Reviewer 2:

I am surprised: this reaction was already published by the Suzuki group and the authors should have been able to easily find this. Suzuki has used the same NHC as catalyst... The following text is from a review (<https://www.ncbi.nlm.nih.gov/pmc/articles/PMC3820033/>):

Suzuki and co-workers achieve aromatic substitution of fluoroarenes with a variety of aldehydes in good yields [91, 92]. Imidazolilydene carbene formed from 143 catalyzes the reaction between 4-methoxybenzaldehyde 22a and 4-fluoronitrobezene 141 to provide ketone 142 in 77% yield (Scheme 20). Replacement of the nitro group with cyano or benzoyl results in low yields of the corresponding ketones. The authors propose formation of the acyl anion equivalent and subsequent addition to the aromatic ring by a Stetter-like process forming XXVIII, followed by loss of fluoride anion to form XXIX.
91. Suzuki Y, Toyota T, Imada F, Sato M, Miyashita A. Chem Comm. 2003:1314.
92. Suzuki Y, Ota S, Fukuta Y, Ueda Y, Sato M. J Org Chem. 2008;73:2420.

*Response: We extend our gratitude to Reviewer 2 for their valuable insights into the Suzuki group's research. We acknowledge the oversight of their relevant publications in our initial manuscript and upon careful consideration of the Suzuki group's work, particularly their notable achievements in the aromatic substitution of 4-nitrofluorobenzenes with aromatic aldehydes, we have now included references to Suzuki et al.'s publications in our revised manuscript [Ref.11]. Nonetheless, we want to reiterate that our study mainly focuses on perfluorinated diarylmethanones. We are particularly interested in the development of a mild, facile, and metal-free methodology for the synthesis of* ***functional perfluorinated diarylmethanones*** *as these moieties hold crucial significance in pharmaceuticals, materials science, and agrochemicals. In fact, we were successful in translating this to practical application through the synthesis of the bioisosteric analog of a drug candidate and were able to utilize these coveted ketones as electrolyte additives in lithium-ion batteries (LIBs), specifically enhancing the cycling stability and maintaining the efficiency of LIBs.*

*In terms of chemistry for the synthesis of these ketones, the traditional synthetic approaches have proven challenging, relying on stoichiometric quantities of intricate metallic reagents [Ref. 9a and 9b]. While Suzuki’s group has made notable strides in developing the NHC-S_N_Ar reaction, their primary focus centers on optimizing N-heterocyclic carbenes (NHCs) as catalysts for the nucleophilic aroylation of 4-nitrofluorobenzenes. However, their preference for substrates with nitro groups attached to fluorobenzenes limits the structural diversity attainable within their methodology. In contrast, our approach offers enhanced versatility, accommodating a wider array of substrates, including modifications of biorelevant molecules, as demonstrated in our substrate scope. Furthermore, as previously mentioned, we further highlight the versatility of our approach through the diverse and impactful applications through the synthesis of bioisosteric analogues for drug candidates and the potential utilization of these compounds as electrolyte additives in LIB*

*We believe our research presents a promising pathway for synthesizing perfluorinated diarylmethanones, carrying implications of significance for both academic inquiry and industrial applications. With confidence, we anticipate that our findings will resonate with the discerning readership of Organic Letters and contribute meaningfully to the ongoing discourse in the field.*

------ End of comments ------

We have thoroughly incorporated the feedback provided by the reviewers and thank them for their remarks. We are confident that the revised manuscript we have submitted to *Organic Letter* is significantly improved.

Thank you for considering our submission. We eagerly await your feedback and are available for any further discussions or clarifications.

Yours sincerely,

Hsuan-Hung, Liao
